# Dynamic Expression of Long Non-Coding RNAs (lncRNAs) in Adult Zebrafish

**DOI:** 10.1371/journal.pone.0083616

**Published:** 2013-12-31

**Authors:** Kriti Kaushik, Vincent Elvin Leonard, Shamsudheen KV, Mukesh Kumar Lalwani, Saakshi Jalali, Ashok Patowary, Adita Joshi, Vinod Scaria, Sridhar Sivasubbu

**Affiliations:** 1 Genomics and Molecular Medicine, CSIR Institute of Genomics and Integrative Biology, Delhi, India; 2 G.N. Ramachandran Knowledge Center for Genome Informatics, CSIR Institute of Genomics and Integrative Biology, Delhi, India; 3 Academy of Scientific and Innovative Research (AcSIR), Anusandhan Bhavan, New Delhi, India; Medical College of Wisconsin, United States of America

## Abstract

Long non-coding RNAs (lncRNA) represent an assorted class of transcripts having little or no protein coding capacity and have recently gained importance for their function as regulators of gene expression. Molecular studies on lncRNA have uncovered multifaceted interactions with protein coding genes. It has been suggested that lncRNAs are an additional layer of regulatory switches involved in gene regulation during development and disease. LncRNAs expressing in specific tissues or cell types during adult stages can have potential roles in form, function, maintenance and repair of tissues and organs. We used RNA sequencing followed by computational analysis to identify tissue restricted lncRNA transcript signatures from five different tissues of adult zebrafish. The present study reports 442 predicted lncRNA transcripts from adult zebrafish tissues out of which 419 were novel lncRNA transcripts. Of these, 77 lncRNAs show predominant tissue restricted expression across the five major tissues investigated. Adult zebrafish brain expressed the largest number of tissue restricted lncRNA transcripts followed by cardiovascular tissue. We also validated the tissue restricted expression of a subset of lncRNAs using independent methods. Our data constitute a useful genomic resource towards understanding the expression of lncRNAs in various tissues in adult zebrafish. Our study is thus a starting point and opens a way towards discovering new molecular interactions of gene expression within the specific adult tissues in the context of maintenance of organ form and function.

## Introduction

The enormous success of major genome sequencing projects in this century was soon consumed with greater challenge of discovering and functionally annotating transcripts encoded by the genome. Thousands of novel RNA transcripts were uncovered by systematic sequencing of full-length cDNA libraries in eukaryotes [1], [2]. These studies estimated that over 70–75% of the eukaryotic genome encoded for transcripts of diverse nature [3]. Many of these transcripts did not have an obvious potential to encode for protein and were popularly called non-coding RNAs (ncRNAs). Genome-wide large-scale projects such as FANTOM 3 uncovered incomprehensible nature of the non-coding RNA transcription by detecting ∼35,000 non-coding RNA transcripts from ∼10,000 distinct loci in the mouse genome [1]. In human cells, genome-wide transcriptome mapping as part of the ENCODE project annotated about 18,400 non-coding RNAs including tRNA, rRNA, HYPERLINK “http://en.wikipedia.org/wiki/MicroRNA”microRNA and other non-coding RNA genes [3], [4].

The non-coding RNAs (ncRNA) are broadly classified into long and small ncRNAs depending upon length of the transcript and have been implicated in regulating expression of key genes involved in the maintenance of biological processes [5]–[7]. At least four classes of regulatory small ncRNAs have been described including short interfering RNAs (siRNA), small nucleolar RNA (snoRNA), piwi-interacting RNAs (piRNAs) and microRNAs (miRNAs) [5]. Among the small ncRNAs, miRNAs are the most well studied, phylogenetically conserved and are found to be indispensable for the development and functioning of an organism [6].

Long non-coding RNAs (LncRNAs) have emerged as a major class of novel regulating transcripts, which are ≥200 nucleotides and display spatio-temporal expression suggesting precise function [8]. In contrast to small ncRNAs, lncRNAs form an enigmatic class of transcripts, which regardless of having characteristic mRNA signatures such as 5′-capping, splicing, and poly-adenylation are not functionally well annotated [9]–[11]. Xist and H19 were amongst the earliest discovered lncRNAs using conventional gene discovery methods [12]–[14]. Subsequently, several other lncRNAs have been discovered [7]. The Allen Brain Atlas has documented 849 lncRNAs within the mouse brain, similarly ∼1,600 long intervening non-coding RNA (lincRNAs) have been identified in mouse cell types using epigenetic marks and ∼3,300 lincRNAs have been discovered in human cell types [15]–[17]. The importance of long non-coding RNA transcription is underscored by the fact that the human genome has four times more lncRNA sequences represented than the protein coding transcripts [18]. Projects like GENECODE (http://www.gencodegenes.org) and NONCODE (http://www.noncode.org) have focused on identification and annotation of lncRNAs. At least 9,640 human lncRNA loci, representing ∼15,512 transcripts have been reported by GENCODE 7 and over 11,000 lncRNAs were identified in the mouse genome by the FANTOM consortium [19], [20].

Several model organisms including zebrafish have been explicitly used for deciphering the functional role of lncRNAs [6], [20]–[25]. Zebrafish has emerged as an excellent vertebrate model organism for studies focusing on discovery and biology of non-coding RNA transcription in developing embryos as well as adult tissues [26]–[28]. The functional roles and interactions of small and long ncRNA transcriptome have been well studied in developing zebrafish embryos, worms and flies [29]–[33]. A recent study identified 550 lincRNAs in three developmental stages of zebrafish by using chromatin marks, RNA sequencing and Poly (A) site mapping. Conserved lincRNAs such as Cyrano (linc-oip5) and megamind (linc-birc6) have been documented to have specific function during zebrafish brain morphogenesis and eye development respectively [25]. An independent study also identified 1,133 long non-coding transcripts originating from diverse genomic loci through transcriptome sequencing of eight developmental stages of zebrafish. Furthermore, the study also documented tissue-specific expression and sub-cellular localization patterns of long non-coding RNA transcripts [24]. Collectively, these studies suggest that lncRNAs may have spatial and temporal expression with potentially important roles during embryogenesis in zebrafish. However, relatively less is known about lncRNAs and their biological functions in adult tissues of zebrafish. Deciphering the repertoire and expression profiles of lncRNAs in adult tissues of zebrafish would enable better understanding of gene regulation within individual tissues types.

In this study, we report a compendium of lncRNAs expressed in five major tissue types of adult zebrafish. In complement to the recent studies in zebrafish that focused on identification of lncRNAs across narrow windows of early developmental time points [24], [25], we have analyzed and compiled the lncRNA transcriptome within functional tissues in adult zebrafish. Using RNA sequencing of five tissue types of adult zebrafish viz heart, brain, liver, muscle and blood followed by multi-filter computational analysis pipeline, we predicted 442 putative lncRNA transcripts including 419 novel lncRNA transcripts. Further, analysis of 419 putative novel lncRNAs revealed 77 high confidence unique tissue restricted lncRNA transcripts in adult zebrafish. The dynamic expression of these lncRNAs among the five tissues was also investigated. A subset of lncRNAs was validated for their expression in the tissues and these transcripts displayed predominant tissue restricted expression in both zebrafish embryos and adult tissues. The identification of tissue restricted lncRNAs in zebrafish opens up the avenues to explore and characterize their unique roles in organ maintenance and the study has implications to discover new molecular interactions of gene expression within the specific adult tissues.

## Results

### Sequence data generation and mapping

Poly-A RNA was obtained from total RNA for five tissues viz, heart, liver, muscle, brain and blood of adult zebrafish and RNA sequence reads were generated using sequencing-by-synthesis method [34]. Approximately, 193 million raw paired-end sequence reads of 51 base pairs (bp) were obtained from five tissue libraries. Sequence reads were aligned to the zebrafish reference genome (Ensembl Zv9 build; hereafter called as Zv9). Approximately, 171 million sequencing reads (88.66%) were successfully mapped back to the reference genome (Table 1). These mapped reads were processed further for analysis.

**Table 1:** RNA-sequencing data production and alignment results for tissue-specific Poly (A) reads.

	Heart	Liver	Muscle	Brain	Blood	Total
**Raw Reads**	43,928,174	50,627,322	34,505,562	29,973,480	34,161,882	193,196,420
**Mapped Reads**	38,076,909(86.6%)	43,416,137(85.7%)	32,421,815(94%)	27,347,501 (91.2%)	30,024,662 (87.8%)	171,287,024 (88.66%)

The total number of sequence reads obtained from the five zebrafish tissues using RNA sequencing is described. Mapped reads represent all transcripts that aligned back to the zebrafish reference genome (Zv9).

### Tissue restricted lncRNA identification

**Figure 1:**
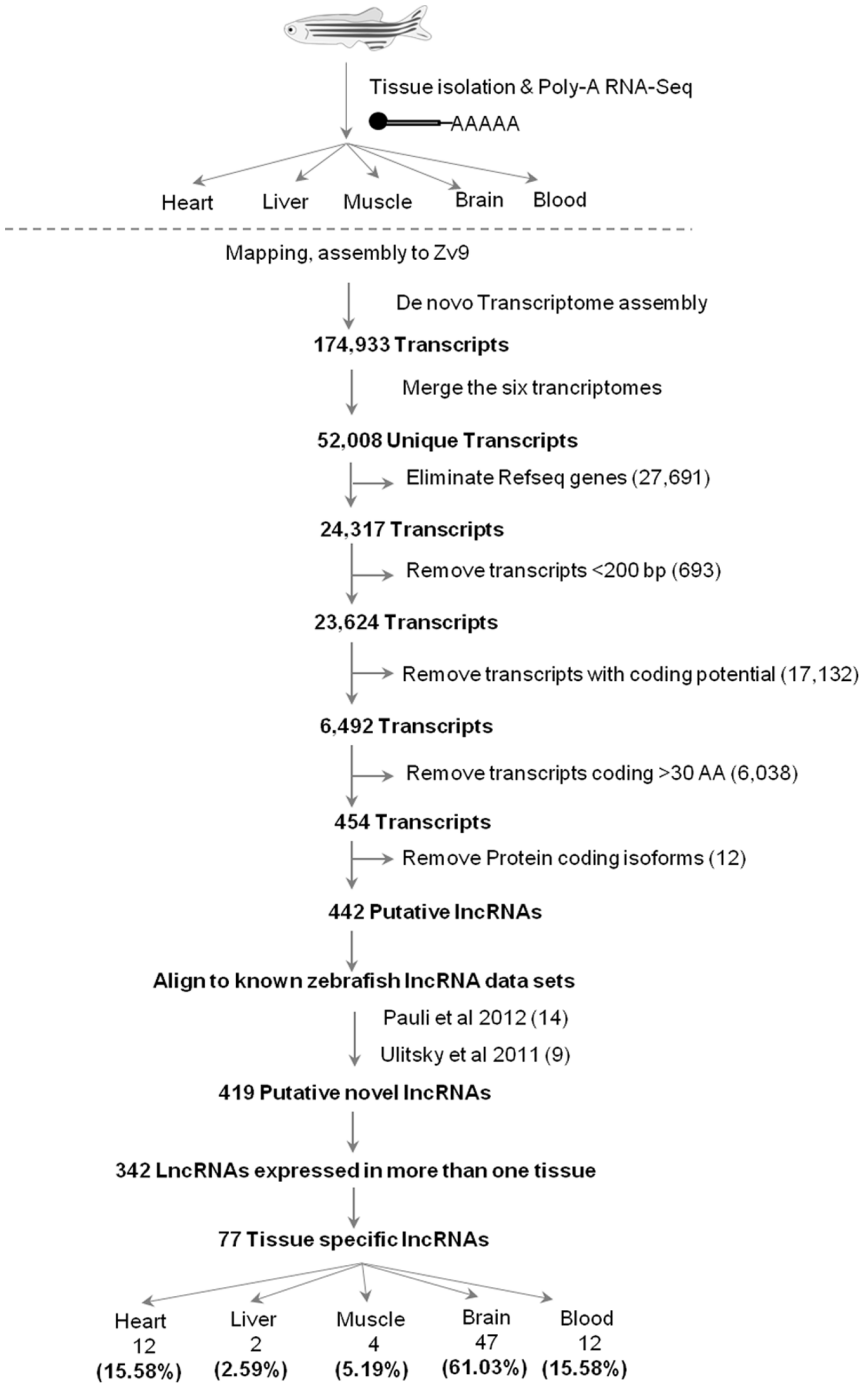
Overview of RNA-seq and analysis pipeline for identification of tissue specific lncRNA. Outline of computational pipeline and systematic workflow for discovering tissue specific long non-coding RNAs. Refer to text for description.

The sequencing reads that mapped to the zebrafish reference genome were analyzed by a custom designed computational pipeline to catalogue high confidence tissue restricted lncRNA transcripts. Details of the computational analysis pipeline are provided in the methods section. The sequencing reads corresponding to the individual tissue libraries were subjected to a reference based transcriptome assembly. This transcriptome assembly in total predicted 174,933 transcript loci from the five tissues. The transcriptomes of the five tissues were further merged together to yield a common dataset of 52,008 unique transcript loci (Figure 1). From this core dataset of 52,008 uniquely predicted transcripts, 27,691 transcripts overlapping with Refseq genes were removed. The remaining 24,317 transcript loci were filtered based on their length and 693 loci that were less than 200 bp were removed, as these could represent potential small RNA loci in the genome. The remaining 23,624 predicted transcript loci were evaluated for their coding potential [35]. Of the 23,624 predicted transcripts, 17,132 transcripts had a positive coding potential score, thus representing potential protein coding transcript loci and were removed from further analysis. The 6,492 transcripts with negative coding potential score were retained, as these would represent putative non-coding transcripts. These remaining 6,492 putative non-coding transcript loci were subjected to an independent open reading frame (ORF) prediction in all six frames [36]. Based on the ORF prediction, 6,038 transcript loci that could potentially code for thirty or more amino acids were removed from the analysis, as these would represent potential small peptides [20]. This resulted in a total of 454 non-coding transcript loci. Of the set, 12 transcripts that showed partial overlap with predicted protein coding gene isoforms were removed from further analysis. The remaining 442 predicted transcript loci represent potential lncRNAs identified from the zebrafish tissues. The 442 predicted lncRNAs were analyzed for overlaps with previously known lncRNA loci in zebrafish [24], [25]. We found that 23 lncRNA loci derived from our analysis overlapped with the previous studies. Thus from this study, we identified 419 potential novel lncRNAs (Table S1).

**Figure 2:**
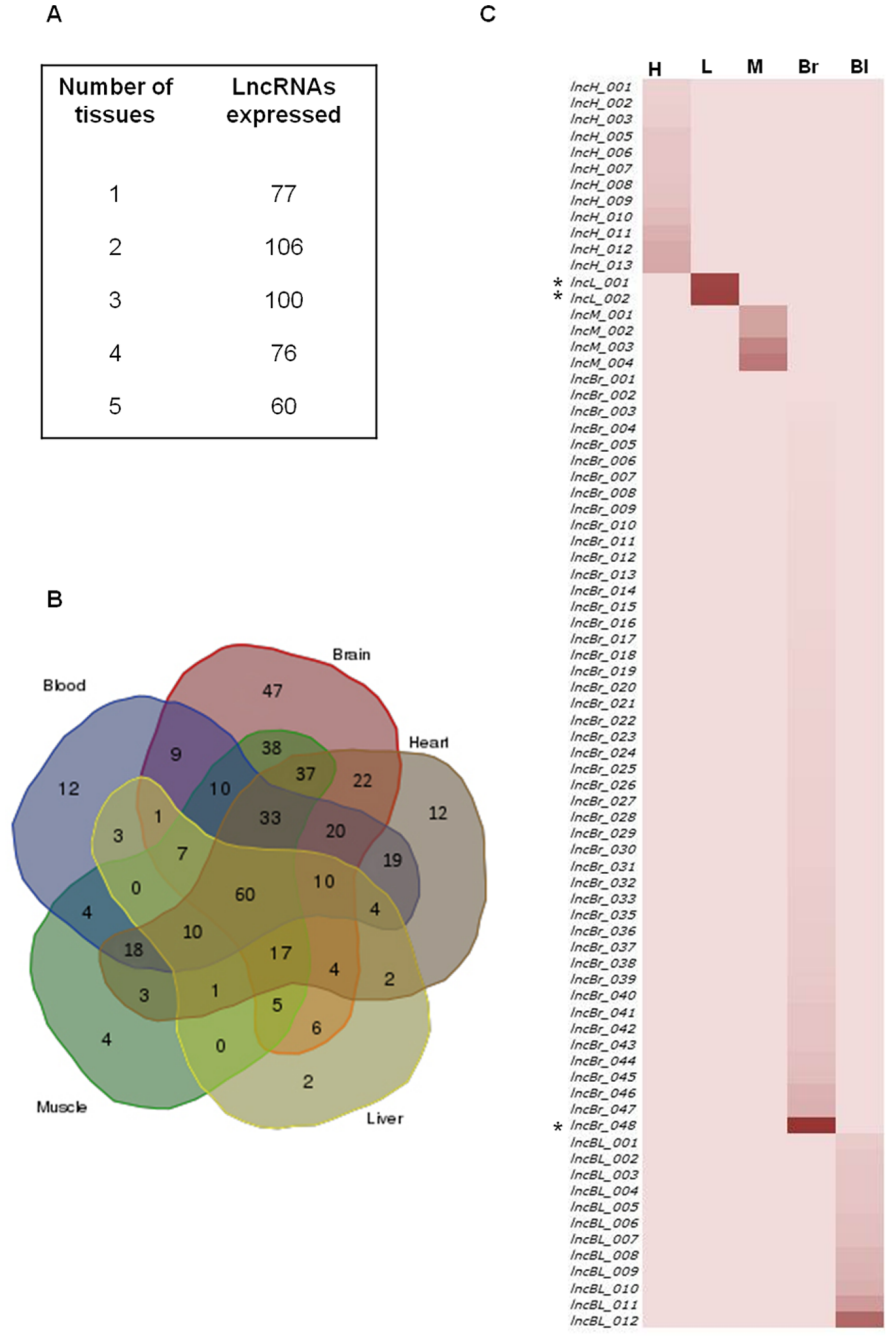
Tissue-wise distribution of predicted novel lncRNAs. Distribution of 419 putative novel lncRNAs across five tissues. The table depicts the number of putative lncRNAs that are expressed either in single or multiple tissues. A. Venn diagram representing 419 putative lncRNAs across five tissues. The overlapping expression profiles of predicted long non-coding RNA transcripts is depicted in different colours across five tissues viz; brain (red), liver (yellow), muscle (green), blood (blue), heart (grey). B. Differential expression of unique tissue restricted lncRNA transcripts. Heat maps of 77 lncRNA transcripts across the five tissues viz heart (H), liver (L), muscle (M), brain (Br) and blood (Bl) are represented. Each individual heat map represents the number of lncRNA transcripts predicted for the corresponding tissue type and its expression levels in the parent tissue versus other tissues based on the FPKM values. Asterisk (*) indicates lncRNA transcripts with highest FPKM values. The colour key represents the FPKM values in the range of 0 for transcripts with the least expression to 12.5 for those with the highest expression.

Of the 419 potential novel lncRNAs, we found that 342 lncRNAs were expressed in more than one tissue investigated in this study (Figure 2A, 2B). The remaining 77 lncRNA displayed putative restricted expression to a single tissue and were labeled as “tissue restricted lncRNAs” (Figure 2C, Table S2). Among the five tissues, brain tissue expressed the maximum number of lncRNAs (47) followed by heart tissue (12) and blood tissue (12). Muscle tissue (4) and liver tissue (2) had relatively low number of lncRNAs. Brain as a tissue accounted for 61%, followed by cardiovascular tissues such as heart and blood, which together accounted for 31% of the putative novel lncRNAs. Liver and muscle represented 3–5% of the total collection (Figure 1).

### Expression profile of tissue specific lncRNome

As a part of this study we identified 419 putative novel lncRNAs from five zebrafish tissues, of which 77 putative lncRNA show tissue restricted differential expression (Figure 1). We have performed detailed expression analysis of 419 potential novel lncRNAs using Fragments Per Kilo base of exons per Million fragments generated (FPKM) scores derived from the RNA sequencing data in order to examine distribution of these lncRNAs across five tissues of zebrafish. Approximately, 50% of the transcripts were expressed in 2–3 tissues and 15% were expressed in all the five tissues (Figure 2A). A Venn diagram representing the overlapping expression of all 419 transcripts in five tissues is shown (Figure 2B), suggesting their dynamic expression across five tissues. We have also observed that amongst the 77 tissue restricted lncRNA, transcripts *lncL_001*, *lncL_002* (Liver) and transcript *lncBr_048* (Brain) show the maximum expression (Figure 2C). Diverse expression patterns of lncRNAs were observed in all the tissues investigated (Figure 2 and Figure S1). In summary, we found that majority of the putative lncRNAs transcripts were expressed in more than one tissue type of adult zebrafish (Figure 2A, 2B and Figure S1) and approximately 17% of the putative novel lncRNA show tissue restricted expression pattern (Figure 2C).

### Expression of embryonic lncRNA transcripts in adult tissues of zebrafish

**Figure 3:**
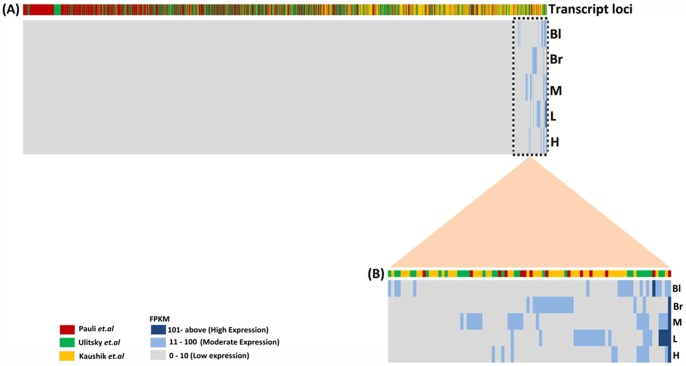
Distribution of embryonic lncRNA transcripts in adult tissues of zebrafish. A. Clustered heat maps of 2,266 lncRNA transcripts obtained from Pauli et(H), liver (L), muscle (M), brain (Br) and blood (Bl) are represented. The color key represents the FPKM values in which grey color indicates the range from 0 to 10, light blue indicates the range from 11 to 100 and dark blue indicates 101 and above FPKM values for those with the highest expression. B. Enlarged section of the heat map depicting differential expression profile of 90 lncRNA transcripts expression across five tissues.

Previously, two groups had identified 1,133 and 691 lncRNA transcripts respectively, originating from diverse genomic loci from different developmental stages of zebrafish [24], [25]. We coupled the lncRNA transcripts identified from the previous studies with those obtained from the current study to yield a total of 2,266 lncRNA transcripts. The respective FPKM values of the 2,266 lncRNA transcripts were analyzed in the transcriptome dataset obtained from the five tissues of adult zebrafish. The FPKM values for the 2,266 lncRNA transcripts across the five tissues of adult zebrafish are provided in Table S3. The analysis revealed that 1,228 embryonic lncRNAs (547 lncRNAs from Ulitsky et al. (2011) [25] and 681 from Pauli et al. (2012) [24]) were present in the transcriptome dataset obtained from the five tissues of adult zebrafish. The clustered heat map of 2,266 lncRNA transcripts based on their FPKM value revealed that embryonic lncRNA transcripts are differentially expressed across the adult tissues investigated (Figure 3A, 3B). Further analysis revealed that the embryonic lncRNA transcripts are predominantly expressed in relatively low levels in the adult tissues investigated (Figure 3, Table S3). In summary, our analysis showed that embryonic lncRNA transcripts were present as RNA transcripts in the transcriptome dataset obtained from the five tissues of adult zebrafish. However, these were not considered as lncRNA transcripts based on the computational analysis used in this study (summarized in Figure 1).

### *In vivo* validation of predicted lncRNAs

**Figure 4:**
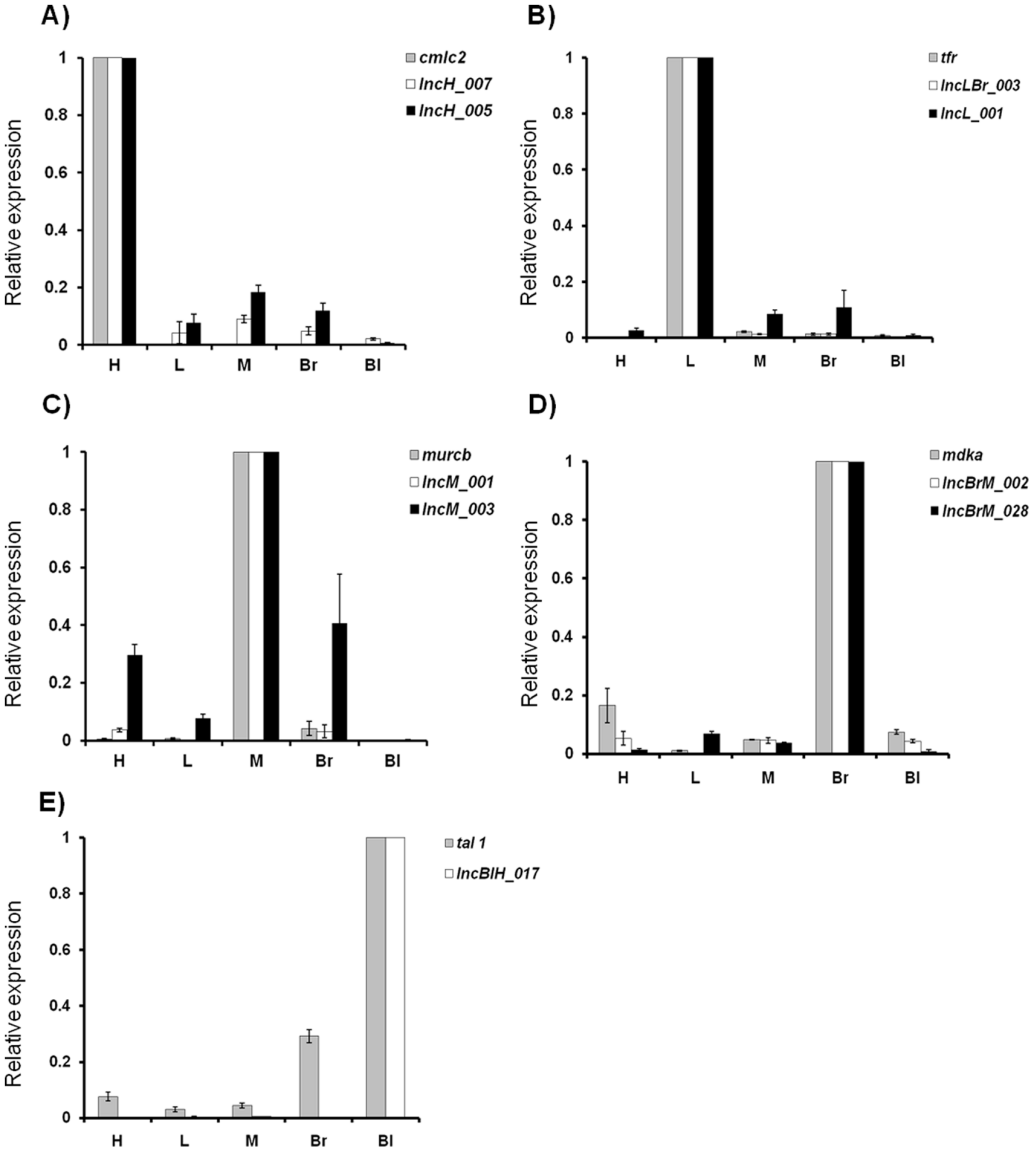
Real time assay for putative tissue restricted lncRNAs. Expression of candidate lncRNA transcripts was analyzed by semi quantitative RT-PCR in A) heart; B) liver; C) muscle; D) brain and E) blood tissues. A tissue specific protein coding marker gene viz *cmlc2* (heart); *tfr* (liver); *mdka* (brain); *murcb* (muscle) and *tal1* (blood) was used as standard control. See text for details on selection of protein coding marker genes. LncRNA transcripts investigated for a particular tissue type showed relatively predominant expression in the specific tissue when compared with other tissues.

A subset of predicted tissue restricted lncRNAs was chosen for validation using real time polymerase chain reaction (RT-PCR) and whole mount *in situ* hybridization (WISH). A known protein coding gene that displays exclusive expression in each of the investigated tissues was selected and used for determining the purity of the isolated RNA, in addition to being an experimental control. Regulatory myosin light chain (*cmlc2*), which expresses in cardiomyocytes [37], was chosen as a protein coding gene marker for the heart tissue and the expression for putative lncRNA transcripts was evaluated. In this study *cmlc2 was* primarily expressed in the heart tissue and its expression in the other four tissues was not detected. Putative lncRNAs, *lncH_005* and *lncH_007* showed predominant expression in the heart tissue with trace expression in tissues such as liver, muscle, brain and blood (Figure 4A). We selected *transferrin* receptor coding gene *tfr,* which expresses mainly in the hepatocytes as the protein coding gene marker for liver tissue [38]. The *tfr* transcripts expressed only in the liver tissue and the putative lncRNAs, *lncL_001* and *lncLBr_003* revealed prevalent expression in liver tissue. The lncRNA *lncLBr_003* was detected in comparatively small amounts in muscle and brain tissues (Figure 4B). Muscle-related coiled-coil protein b (*murcb*) expression was seen mainly in the muscle tissue along with minimum detection in the brain (Figure 4C). Putative muscle restricted lncRNA, *lncM_001* showed restricted expression in the muscle only whereas *lncM_003* had moderate expression in the brain and heart tissues also (Figure 4C). *Midkine a* (*mdka*), a protein coding gene that uniquely expresses in brain tissue [39], was chosen to evaluate relative expression of putative brain specific lncRNA transcripts. *LncBrM_002* and *lncBrM_028* show predominant expression in the brain with trace expression in other tissue types (Figure 4D). T cell acut*e lymphocytic leukemia protein* 1 (*tal 1*) was used as protein coding marker and displayed predominant expression in blood tissue with minimal expression in the brain (Figure 4E). The transcript *lncHBl_017* was found to express specifically in the blood tissue and its expression was absent in the other tissues investigated.

**Figure 5:**
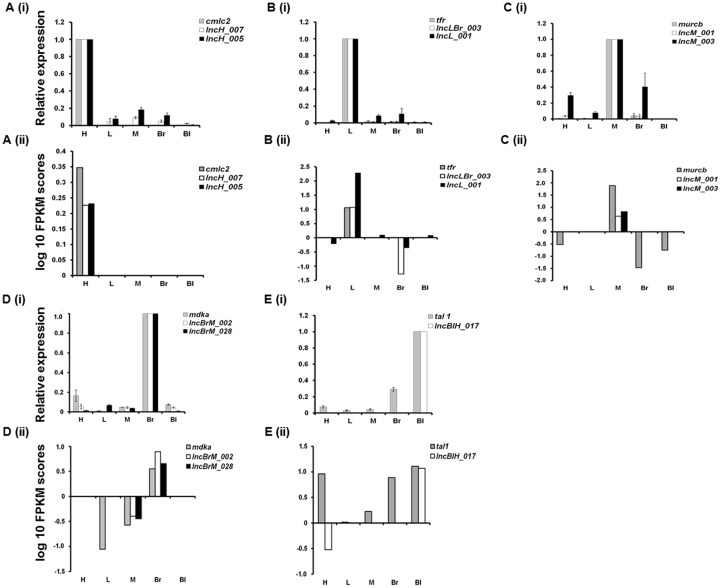
FPKM values are consistent with lncRNA expression. Expression of lncRNAs validated via RT-PCR for each tissue is compared with their corresponding FPKM values obtained from RNA sequencing. By and large, tissue specificity of the lncRNA transcripts as reflected by FPKM values shows reasonable overlap with their relative expression profiles across tissues obtained from RT-PCR assay. A(i), A(ii) Heart; B(i), B(ii) Liver; C(i), C(ii) Muscle; D(i), D(ii) Brain; and E(i), E(ii) Blood tissues.

We further compared the RNA sequencing derived FPKM values of predicted lncRNAs transcripts with the fold change values of RT-PCR assay in order to evaluate the reproducibility of the tissue restricted lncRNA expression (Figure 5). Analysis showed good concordance between RT-PCR data and FPKM score (Figure 5). This suggests that the trends of tissue restricted lncRNA expression were similar in RNA sequencing and RT-PCR assays. In summary our RT-PCR assay reproduced the relative transcript abundance of predicted tissue restricted lncRNAs similar to that observed by RNA sequencing.

**Figure 6:**
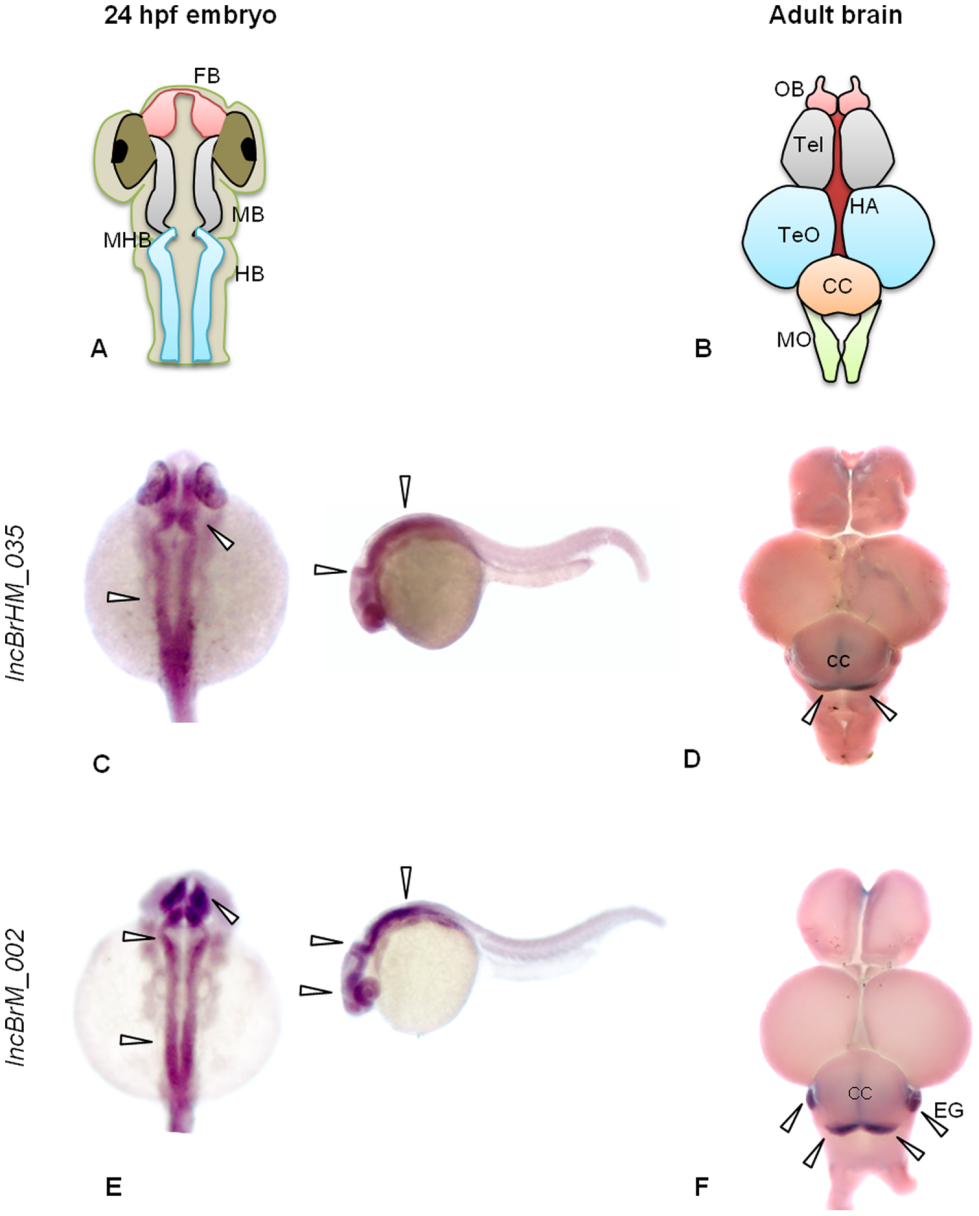
LncRNAs show tissue restricted expression patterns. Whole mount *in situ* hybridization of lncRNA transcripts. Shown are images with probes specific to the two indicated brain restricted lncRNAs. Arrow heads indicate the expression domains. A and B Anatomical cartoons of 24 hpf developing zebrafish embryo and adult zebrafish brain. C and D Expression of lncRNA transcript *lncBrHM_035.* (C) Dorsal view (anterior up) and lateral view (anterior to the left) showing expression in mid-hind brain boundary and hind brain of 24hpf zebrafish embryos. (D) Dorsal view (anterior up) of the adult zebrafish brain showing expression in regions of cerebellar crest (CC). E and F Expression of lncRNA transcript *lncBrM_002.* (E) Dorsal view (anterior up) and lateral view (anterior to the left) showing expression in fore-brain (FB), mid-hind brain boundary (MHB) and hind brain (HB) of 24hpf zebrafish embryos. (F) Dorsal view (anterior up) of the adult zebrafish brain showing expression in the regions of CC and a localized signal in eminentia granularis (EG). MB, mid brain; OB, olfactory bulb; Tel, telencephalon; Ha, habenula; Teo, optic tectum; MO, medulla oblongata.

To further verify whether the predicted lncRNA transcripts were predominantly expressed and localized in the specific tissues, we performed whole mount RNA *in situ* hybridization (WISH) for two brain restricted lncRNAs, *lncBrHM_035* and *lncBrM_002* in adult brain organ as well as developing embryos (Figure 6). Prior to examining the expression of lncRNA using WISH, we performed 3′ RACE (Rapid Amplification of cDNA Ends) of lncRNA transcripts *lncBrHM_035* and *lncBrM_002,* in order to confirm the directionality of the lncRNA transcript in the genome (data not shown).

*lncBrHM_035* transcript displayed distinct localization in the eye, mid and hind brain of 24hpf zebrafish embryos (Figure 6C) and was found to be expressing explicitly in cerebellum of adult zebrafish brain (Figure 6D). Another brain restricted lncRNA transcript, *lncBrM_002* could be detected in mid and hind-brain of 24hpf zebrafish embryos (Figure 6E) and showed restricted expression in cerebellum and EG (eminentia granularis) of adult zebrafish brain (Figure 6F). The WISH data revealed that the predicted tissue restricted lncRNA expressed in adult organs and displayed slightly overlapping expression profiles in developing organs during early embryogenesis. In summary, we have used three independent approaches, namely RNA sequencing, RT-PCR and WISH for determining the expression of putative lncRNAs across five tissues. Collectively, the results of the assays suggest that the predicted lncRNAs display defined tissue restricted boundaries of expression.

## Discussion

Non-coding RNAs have been documented to display a high degree of specificity in their domain of expression. A number of studies have shown tissue-restricted expression for short non-coding RNA such as microRNAs [40]–[43]. Recently, we reported that expression of miR-142a-3p was restricted to the vasculature endothelium and has a role in developmental angiogenesis in zebrafish [32]. In contrast to rich literature on the tissue specific expression domain and function of miRNAs, evidence for tissue restricted expression for long non- coding RNA is still formative. Studies have described tissue and cell type specific, spatio-temporal regulated expression of the lncRNA transcripts, suggesting putative functional roles [15], [44], [45]. Studies on the lncRNA expression indicate that brain as a tissue expresses the largest repertoire of lncRNA transcripts and displays conserved expression within specific domains across amniotes [46], [47]. *Evf2*, a long non-coding RNA, transcribed from an ultra-conserved genomic region, displays explicit expression in mouse brain and regulates activity of Dlx homeodomain genes across vertebrates [48]. LncRNAs such as Gomafu show distinct localization within sub-cellular compartments (nuclear) in neurons [49]. Another study found that a neural specific lncRNA, *CASK regulatory gene (CRG)* in *Drosophila* participates in locomotor and climbing activity [50]. LncRNAs are also known to express as pairs with protein coding genes and co-localize at genomic level in developing brain [51]. LncRNA such as tie-1AS are known to express specifically in vascular endothelium and regulate the tie-1 coding transcript [52]. The roles of lncRNAs such as braveheart, Fendrr and LINCRNA-EPS have been documented in early cardiovascular lineage commitment, heart development and erythroid differentiation respectively [23], [53], [54]. Apart from directly interacting with protein coding genes, lncRNAs also act as a decoy of miRNA as in the case of linc-MD1, a muscle specific lncRNA [55].

Majority of the literature pertaining to lncRNA in zebrafish is primarily focused on describing functional roles during early developmental stages. However, information regarding their expression profile and biological role in adult organ function and maintenance is limited. This study describes the lncRNA expression landscape from tissues of diverse function in an adult zebrafish. Next generation high throughput sequencing technology was used to capture the polyadenylated transcripts, which were then subjected to a computational analysis pipeline leading to the identification of putative novel lncRNAs from five tissues derived from adult zebrafish. A total of 52,008 transcripts were reconstructed from our RNA sequencing data. A similar number of transcripts 56,535 were reported by Pauli and co-workers in their description of zebrafish embryonic transcriptome. Of 52,008 transcripts identified in our study, 27,691 transcripts corresponded to the RefSeq transcripts and were removed from analysis. The remaining 24, 317 transcripts were subjected to the computational analysis for identification of putative lncRNAs (Figure 1).

In this study we identified 442 putative lncRNAs with high confidence from five major tissues of adult zebrafish. Of these, 14 lncRNA transcripts overlapped with those identified from zebrafish developing embryos [24]. We also noticed that only 9 transcripts in our dataset overlapped with the lincRNA dataset of developing zebrafish embryos reported by Ulitsky and co-workers [25]. Reasons for the minimal overlap in lncRNA transcripts between the previous studies and the present work could be attributed to the stringent computational analysis used in this study, which filtered out a large portion of embryonic lncRNAs that are otherwise present as RNA transcripts in the transcriptome dataset obtained from the five tissues of adult zebrafish. We have also examined the overlap of lncRNA transcripts after modifying the ORF cut off from 30 amino acid to 100 amino acid as used by Pauli and co-workers. When the ORF cut off was set to 100 amino acid, the total number of lncRNA transcripts increased from 442 to 6,214. In addition, the overlap of the lncRNA transcripts with the previous studies also increased from 9 to 176 in case of Ulitsky et al.,2011 and 14 to 197 in case of Pauli et al.,2012 (Table S4). However, it is well known that the higher ORF length could potentially add to the false positive predictions of lncRNA transcripts [56]. Therefore, to avoid false predictions, we have followed stringent criteria of 30 amino acid cut off in our study. Furthermore, we have used a non-stranded RNA sequencing approach in our study and this limits the number of lncRNA transcripts that could be predicted. Lastly, we have investigated transcriptome from adult tissues of zebrafish, which is known to harbor distinctly different transcriptome repertoire from embryonic stages [57]–[59].

Out of the 442 lncRNA transcripts predicted from this study, 419 lncRNAs were classified as putative novel as these have not been reported before. Of the 419 putative novel lncRNAs, 342 lncRNAs were found to be expressed in more than one tissue investigated, suggesting that these lncRNAs transcripts may be important regulators of protein coding genes that may be required for maintenance of the corresponding organs/tissues. The remaining 77-lncRNA transcripts were predicted to have predominant expression restricted to one single zebrafish tissue investigated. The expression of individual lncRNA transcripts varies widely in the tissues investigated. All the five tissues have different subsets of uniquely restricted lncRNA transcripts with almost no expression elsewhere. The expression profiles of lncRNA transcripts derived from the RNA sequencing and RT-PCR for the five tissues indicate a good concordance. In addition, the WISH assay showed the unique and non- overlapping expression domains of the two brain restricted lncRNA transcripts *lncBrHM_035* and *lncBrM_002* in adult brain, which clearly suggests that lncRNA transcripts within a single organ (brain) may have discrete localization patterns that might signify restricted functional activity.

The present study is not without caveats; firstly, we have applied a non-stranded RNA sequencing approach, which limits the number of lncRNA transcripts that could be predicted. Secondly, we have not investigated the chromatin marks flanking the predicted lncRNA transcript loci, which could have revealed additional information on transcript loci. Thirdly, we sequenced only the poly (A) containing RNA transcripts in our study, which prevented the identification of lncRNA transcripts that are devoid of a poly (A) tail [60]. Nevertheless, this catalogue of tissue restricted lncRNA transcripts will be useful for exploring the role of non-protein coding transcriptome in maintenance and repair of tissues. The predominant tissue restricted expression of the lncRNA transcripts may suggest specific functional roles in each tissue type. We speculate that the lncRNA transcripts identified in this study may also help to better understand the recently identified functional interactions amongst mRNA, miRNA and lncRNA [22] in a broader context of processes such as tissue maintenance, repair and regeneration. The strategy outlined here for identifying putative novel lncRNA transcripts can be employed as a methodology for prioritizing and understanding biologically significant of non-coding RNA transcripts. Further, this methodology could be readily applied to a large number of tissue specific fluorescent zebrafish lines for identification of functionally significant non-coding RNA transcripts in specific biological pathways.

## Materials and Methods

### Ethics Statement

Fish experiments were performed in strict accordance with the recommendations and guidelines laid down by the CSIR Institute of Genomics and Integrative Biology, India. The protocol was approved by the Institutional Animal Ethics Committee (IAEC) of the CSIR Institute of Genomics and Integrative Biology, India. All efforts were made to minimize animal suffering.

### RNA isolation

Adult wild type zebrafish were maintained at CSIR-Institute of Genomics and Integrative Biology as per standard practices described [61]. Tissue isolation was performed by anaesthetizing an adult zebrafish by treatment with Tricaine (Sigma, USA). Individual tissues viz heart, liver, muscle, brain and blood were dissected out and utmost care was taken to ward off contamination to obtain pure homogenous samples for each tissue type. The tissues were washed in PBS several times to clean up any debris. The tissue samples were homogenized in Trizol (Invitrogen, USA). RNA isolation from the homogenized tissue samples was carried out using RNeasy kit (Qiagen, USA) as previously described [32].

### Next generation sequencing and data generation

Approximately, 5–10 µg of RNA isolated from the individual tissues was used to capture poly-(A) RNA using Sera-Mag oligo (dT) magnetic beads. The captured poly-(A) RNA was fragmented into small pieces of size ranging from 200–500 bp. This size selected RNA was used for cDNA synthesis followed by second strand synthesis using reverse transcriptase and DNA polymerase I respectively. The overhangs at cDNA ends were repaired to blunt ends with the 3′ to 5′ exo-nuclease activity of Klenow enzyme and synthesis activity of T4 DNA Polymerase. To the blunt ends, single “A” base overhang was added by Klenow (3′ to 5′ Exo minus) activity to facilitate specific pairing with manufacturer specified paired end adaptor with a single “T” base overhang. This was followed by the adaptor ligation to the generated cDNA. These ligated A-tail products were run on a 2% agarose gel and fragments corresponding to 300 bp size were purified and selectively enriched by PCR using adaptor specific primers. Quality of the purified library was verified by agarose gel electrophoresis and the concentrations were measured using Qubit (Life Technologies, USA). The RNA libraries were amplified on the Genome Analyzer IIx (GAIIx) flow cell to generate clusters using Illumina's cBot cluster generation system as per manufacturer specified protocols. Genome Analyzer IIx (GA IIx) sequencing platform from Illumina, USA, was used for sequencing of the RNA libraries. The clusters were sequenced in the GAIIx using sequencing-by-synthesis methodology [34]. High resolution images were captured after every cycle and processed for base calling using Illumina Pipeline software (v1.9). Reads that passed the initial threshold values for quality filter were only used for further analysis. The study accession number (SRA) is PRJNA207719 (SRR891495, SRR891504, SRR891510, SRR891511, SRR891512).

### Assembly of the tissue restricted lncRNome

The RNA sequencing reads were aligned independently to the zebrafish genome (Zv9) using Bowtie and TopHat (v2.0.3) software (http://tophat.cbcb.umd.edu/). Short read aligner Bowtie was used to align the reads to the exons. These aligned reads were processed by TopHat for demarcating splice junctions between the exons. Further, the mapped reads were assembled into transcripts using Cufflinks software (http://cufflinks.cbcb.umd.edu/), which calculates a transcript's relative abundance based on the number of reads supporting the transcript, using a reference annotation file. The Cufflink assembler generates the output in the form of FPKM (Fragments Per Kilo base of exons per Million fragments generated) values. The value of FPKM score is directly proportional to the relative abundance of a transcript in a given sample. Transcriptome assembly corresponding to each of the five tissue types was generated. Following this Cuffmerge script (http://cufflinks.cbcb.umd.edu/manual.html#cuffmerge) was used to merge transcriptome data from all the five tissue samples and to filter out reads representing sequencing artifacts owing to the use of random hexamer primers. Next, all the Refseq genes were eliminated and the remaining transcripts formed the corpus of data that was used for downstream analysis. Any lncRNAs that overlapped with Refseq genes were also removed from further analysis. In the next step, transcripts with a length of more than 200 bp were selected and were checked for their coding potential using Coding Potential Calculator software (http://cpc.cbi.pku.edu.cn/), which distinguishes coding and non-coding transcripts with high accuracy [35]. Coding Potential Calculator applies sequence based features to predict the protein-coding potential of transcripts, and has been widely used to discover long non-coding RNAs [62]. Transcripts with a negative score correspond to a non-coding transcript. Transcripts with a score of <−1 score were selected for further analysis. Further, the selected transcripts were checked for open reading frame prediction (ORF) by Getorf software (http://emboss.sourceforge.net/apps/cvs/emboss/apps/getorf.html). The transcripts with an ORF length of less than thirty amino acids (as defined for lncRNA) were chosen. The final predicted long non-coding RNAs were aligned back to previously known datasets for developmental stages in zebrafish [24], [25]. At this point we also checked for matches to any protein coding isoforms. Those transcripts that had any match with protein coding isoforms were removed from further analysis. The remaining corpus of transcriptome was screened for tissue specific expression using Cuffdiff software (http://cufflinks.cbcb.umd.edu/manual.html#cuffdiff). Cuffdiff software determines the differential expression of transcripts in various tissues. The resulting transcripts were classified as putative tissue restricted lncRNA. The genomic co-ordinates of the identified lncRNA transcripts (BED file) are given in Table S5.

### Quantitative Real-Time PCR (QRT-PCR) assay

RNA was isolated from the tissues dissected from the adult zebrafish, using RNeasy kit (Qiagen) according to manufacturer's instructions cDNA was prepared from 1µg of RNA using Superscript II (Invitrogen, USA). Quantitative Real Time Polymerase Chain Reaction [63] (qRT-PCR) was carried out using Sybr Green mix (Roche, Germany) for detection in Light cycler LC 480 (Roche)**.** The lncRNAs for each tissue were selected based on their FPKM values. Protein coding genes that expressed predominantly in specific tissue types were analyzed in parallel for ensuring purity of the isolated tissues. These protein coding genes were selected on the basis of the *in-situ* data and publicly available gene expression profiles. *Regulatory myosin light chain* (*cmlc2*), *muscle-related coiled-coil protein b* (*murcb*), *midkine a* (*mdka*), *transferin* (*tfr*), and *T-cell acute lymphocytic leukemia protein 1* (*tal 1*) were chosen as protein coding gene markers for heart, muscle, brain, liver and blood respectively. The sequences of primers for the protein coding genes and predicted lncRNAs are given in the Table S6.

### Whole mount *In Situ* hybridization (WISH)

Paraformaldehyde-fixed embryos were processed for *in situ* hybridization according to standard zebrafish protocols (http://zfin.org/ZFIN/Methods/ThisseProtocol.html) [64]. The brain specific lncRNA sequences were amplified from cDNA by PCR using primers (Table S6) and cloned into Topo TA vector (Invitrogen, USA). The *lncRNA* clones were linearized with NotI and digoxygenin (DIG) labeled *in situ* probes were generated by in vitro transcription with SP6 or T7 polymerases using DIG RNA Labeling kit (Roche, Germany).

## Supporting Information

Figure S1 **Differential expression of lncRNA transcripts identified in adult zebrafish tissues.** Heat maps of 442 lncRNA transcripts across the five tissues viz heart (H), liver (L), muscle (M), brain (Br) and blood (Bl) are represented. Each individual heat map represents the number of lncRNA transcripts predicted for the corresponding tissue type and its expression levels in the parent tissue vs. other tissues based on the FPKM values. The colour key represents the FPKM values in the range of 0 for transcripts with the least expression to 196 for those with the highest expression.
(TIF)

Table S1 **A dataset of 419 putative lncRNAs that are predicted to express in five tissues of adult zebrafish.**
(DOCX)

Table S2 **A dataset of 77 putative lncRNAs that are predicted to have predominant expression restricted to particular tissue type investigated.**
(DOCX)

Table S3 **FPKM values of 2,266 lncRNA transcripts across the five tissues of adult zebrafish (Transcript ID with prefix “U” indicates data from Ulitsky et** **al. (2011) and Transcript ID with prefix “P” indicates data from Pauli et** **al. (2012)).**
(DOCX)

Table S4 **Comparison of lncRNA transcripts between the present study and previous studies (Ulitsky et** **al., 2011 and Pauli et** **al., 2012) generated by using ORF cut off set to 100 amino acids.**
(DOCX)

Table S5 **Genomic co-ordinates of the 442 lncRNA transcripts identified in this study.**
(DOCX)

Table S6 **List of oligo sequences used in the study.**
(DOCX)
