# Supplementary figures and images for: Dynamic Expression of Long Non-Coding RNAs (lncRNAs) in Adult Zebrafish
Source: PLoS One. 2013 Dec 31;8(12):e83616. doi: 10.1371/journal.pone.0083616 (PMC3877055; doi:10.1371/journal.pone.0083616)

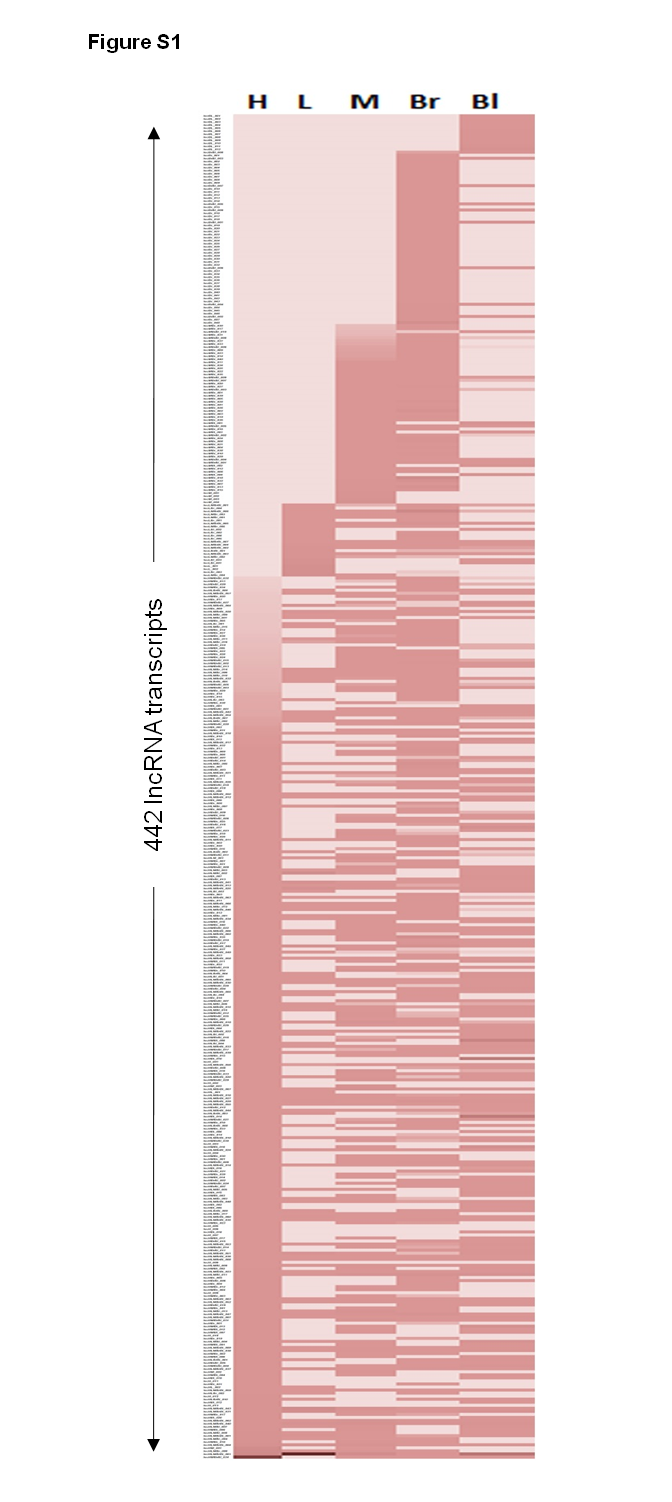

Supplement: Figure S1 — Differential expression of lncRNA transcripts identified in adult zebrafish tissues. Heat maps of 442 lncRNA transcripts across the five tissues viz heart (H), liver (L), muscle (M), brain (Br) and blood (Bl) are represented. Each individual heat map represents the number of lncRNA transcripts predicted for the corresponding tissue type and its expression levels in the parent tissue vs. other tissues based on the FPKM values. The colour key represents the FPKM values in the range of 0 for transcripts with the least expression to 196 for those with the highest expression. (TIF) [file pone.0083616.s001.tif]
