# Supplementary material for: Dynamic Expression of Long Non-Coding RNAs (lncRNAs) in Adult Zebrafish
Source: PLoS One. 2013 Dec 31;8(12):e83616. doi: 10.1371/journal.pone.0083616 (PMC3877055; doi:10.1371/journal.pone.0083616)
Supplement: Table S1 — A dataset of 419 putative lncRNAs that are predicted to express in five tissues of adult zebrafish. (DOCX) [file pone.0083616.s002.docx]

**Table S1:**   A dataset of 419 putative lncRNAs that are predicted to express in five tissues of adult zebrafish.

| **S.No.** | **LncRNA ID** | **Sequence Description** |
| --- | --- | --- |
| 1 | lncH_001 | ATGGAGTTGAGTAGTCTCATGCGAGACTCCAACAAACAAGGCTTTTAACATCTAACGCTGCTACCTGAACCTGCTGTTTCTTAGCTTTTGTCTAAAGTGATCAACACCCTGCCATGTCCTTCCCTGATCCCTCTGTTTCTCTTGCACTTGCTTACATGTTGGCTTAGACTACAGACACAAGCCGTCCAATCCTCCCAAGCTTCTGTCCCCACCTTCTTCTTACATGTCTACCCACACTCCCTCCACACCCTAGCATCCACTGCTTCTGCCCTTTACCCCTTCCTCTGTTTCCCTACTTTACCCATTTCCTTTTTGGTACCCCAACCCCCAACAACCGTTCA |
| 2 | lncH_002 | ACACGCTCAAGCAATACACACATGGTTTGTGTGTGTTTGTGTGTGTGTGTGTGTGTGTGTGTGTGAAAACTGCTGGAGAGAACAATGCTTGCAGAAGAAGCGGCGCTTTACAAAGCCTGTAGATCAGAGTGAGGCAGTGATGGCTGTCAGCAGGCCTGTAGACGGAGATAAGCTCCACTGTCACCGCATTCACTCTCTATTAGATTAGCCAGACAAACCAACTACCACAGCAAAACGTACCCGCTAATCGAAGAATTCAGGCCTTCCCTTGTCCTTAACCCTCGTGTACTTAATATAGAGGATGAGTCAGCTCAAACG |
| 3 | lncH_003 | CTGATTCAATATCTGCGAGAGATTATGGCTGATTATTTAGCTAAATGGACCACACACGGAAAGACGAGTCATCTCACACGCGCTAATCAATTCAAGATACGCTAGCTATTAGCACCCAGTCGAAAACCCGATCGCACTCGCTCGTATATTCAAGCTTACTGTACAGTCCAGTCCACAACAGAAGAACAGACACATCTGCTTTCTTCACTCGTCTTTCTCCTCCCCGCAGCTCAAGCTTTCACTCGCTTTCTCTCTCTGTAGTGGTTTGAGAAAGTTCCAGACATTCCTGAAAGGATTCCGCTCGGCAGCAGCCAGTC |
| 4 | lncH_005 | GTAAAACCTCAAGGGAACCAGGGTTGTATTTGCAAATAATATTTATCTTGTGCTCCTCTTATTTGTCAGATTACCGTGTGTGAATCAAAAATGATGTGCAAGGTCACCCAAAACACAAGTATTTAGGCAGTATTAATAGTCAGTTTAATATTAGGTTAGGTTAGTTTATCCCACCTTAAGCAAGTTTGATTTGCACAGGTGACAGTTTTGTTGACAGTTACACATACAGTAGAAGAACTGAA |
| 5 | lncH_006 | AGATGTTCCCCTGTAATTTACTCACTGAACTTCAATGAAGAGGTTTTTACTAGTTTAAAATGTCAGTTTCTTTTGGAGAACTGGGCTGTTGCTGTGAGACTGTAAAATCAGTTGGGACCAGACTGGAGATGGGACCTTGACTATGAGAGAATGACTATTTGAATAGTTTTAATGTCTAACTTGATTGGGAATGGGACCTATTCTTTGTAAAATAGTTAAAAATACTCAATCTTGAAGAAAAA |
| 6 | lncH_007 | TTAAAATGTAAGTTTAGTCAATGCGGCGTAATTGTAAAGCGCCATCTGTAGGTCAACACTTAAACTGCATCTAACTCAACATTCAACAGGCTGCATTGCAGCATGTTTTTAATCAATTGTTTAATCTTTTAATGAACCACTCCAGCTTCTAAATTCACCCAATATGCTTTTATTAAAAAGAAAAATTATCTATTTTCTGTTTGTAAACCACAAATAATGGCAAACGGTTAATAAATTAAGCATGCTCTCACCCGAGGGTACACAATAGCCTTATTGGTTTCAATCAAGGCTGAATGTGAAGCAAATATTTAGACTGTTGATTTGATATGATGTGAATATGATGTTGTTTTATTAACTAATCTCTCTCCACGGACAGCGACGCGCACACATTTTAATCACAGCAAATCACAATTCAATATCGCGTGCGAGCCACAGCTGCCTGACAAAATAAATGAATAATTTAGCATAAAATAACGTTCAGGGGTGATTAAAATTCAAAACAACAGTAAGAGTTTGGTACCAAGATGCCCT |
| 7 | lncH_008 | AGACACTGGGTTTCATCCACTAATAATTGCATATGTTTTTCATATTTATATGCACATTTGAACCTCACAAAAACTGTCCGCCATCTTCACAACACTTCTTCTGTTAGTGAATTTGATTAGTTCTGAGGGGGTGTCCTCGTGCAGTAATTTGCATAAGACACGCCCACAGCAGCTCCATAGACAGTGTTCAGCATATATAAGTACACGCCTCACAAATCTCTCTTTAAATTAATATTTT |
| 8 | lncH_009 | GCAAGATATGTATTAAATGATATAAAGCACAGAAGATTTTTGTGACTTTGTGAATTGTGGATTTTTCAGTTCCATGAAAATATGTGAATATTTATGCATGCTGTTTTTAGTTATGTGGAGGCGAAATCTGGCCTAGTTACAGCTTGTGTCACTTAAAGCAATATTTTACCATACAAATGAGAGTTCTGTCATCATGTACTTGCTGTTCTAGACCTAATGCTCAGCATATATAAGTACACCCCATGCAAATCCATCTTTTAAATTCATGTTTTAATCAGAAGCTATACAATATTTAATTTGTACATATACGTTATATTTGTCAGAACTGAAGCCAAATCTGGAGCTTATCTAACAAAATAACTCACGATTAACGGTCAAAAAAAACTAGTACAGTGCACCC |
| 9 | lncH_010 | AGTACGAAAGGTTTACATTTAAATTTGCTCTTACACAAAAATGACAAAATGAGGAGAAAGGTAACACAAATGCCTATTGCAGTAATGCCACCTACTGGACATAGTGGATGTTGACACAAAAGCCTGGGTTGTACTGAAATAATTCCTCAAAATGACCTGACCTAAAAAGGAAAGACAAATAAATGTTAGTAAAAACAATGTCAATCACATAAAAACTAAATAAGCAATTCTGTAAATG |
| 10 | lncH_011 | CAAAAATCAGAGGATAACTGAGTATCTGCTGAAGTGTTTCCAGAAACGCTGCAGACATCTGAGGCTGAGAATAAATAACATTCAACTTTCGAATTCTAGTAGTGAGGAAATGTCACAATTATTTCTAATTTCACTAGATTTCCAGCATTCACTGAGCTCCTGAGAGCTTCCCATTCTTTCACAAATGTTTGAAGATTTTCT |
| 11 | lncH_012 | AAGAAAACAGCCCAGTCAATTGCAATTCTCATTTTGATTTGCTTTTTAGTCAACCATGAGTTTGGGATGCTGTTTTTGCTTTGCACTTCTTGTTTCTGCACATTTGACAAGGGAGTAATGAGTACAAAGGAGTAAATGAAACTTGGCCGTTTACAGCAGGCCCCTTGACTCCGCCCCCTTGTCAAATCAGGGCCACAAAGTGAAACGCGCAGAAACGTGAAGTGCAAAGTGAAAACAGCATCACAAACTCAAGATTGAATAAATGCAAATATTTTAGTTTATTTTTGCTCTGC |
| 12 | lncH_013 | AAACAAAATCTACTGATTGAAAGGAACCTGTTTTTCTAGGGAGGTTTAAAGATGAGCAGTAAAGACCAGGAAGATGGCGCCACCCAGTGTCTGAAAGCTCAGAGGTCTAGGAGGAGAAACGGGATCACATGAATCATCATCACATCATTTTTGTGACACTGTACAATGATTAACATTTTTACATTAGTGTGAGCGTTGGGGTAAGCA |
| 13 | lncL_001 | GCTAGATACATTGTTCTCCATGAAATAAAAAGCTGACATTATTTTCTGTCCCCTTATGCCTTTACCAAAGGCAAGTCTAAAATAAAACAAATAACTAGCTTAGGTTACCTCATGTACTTCATTCCAAAACCTCTAAAGCCACAGGATAACTTTCTGTGAGAAACAGTCCACATTCAATCATTATTCACTGATAATGAAGATAAGCTGCAGCACAGTTTAGGATTAGCAGTGCACTA |
| 14 | lncL_002 | CTGTGTTGTGCGTTAATATAGTGTGGGACATCAGCGGGATTAGGAGTATTTGGTCTTAAATCGGGGTAGTGTGAACGGTCCCTCCCCAGCAATGGGTTAAAGCGGCATTACAGCTTGTAAGATGGGTGTCCCGCAGATCTCCCGGGAGACCAGCTCCACTTGGGTGTTCCAGCACTGAGCACAGACCATTTAGATGACACGGAGGTGTTCTGATGCCATCTTC |
| 15 | lncM_001 | AACTGACCTACAATTTTATCCCAAAATAAACAAGAAAATTGAGAATACATCAAACAAAGACAATCATCACATTGTCCTTTAATCTAAAATATTTAATTTTCACTTTTATTTACAACCTATTAAATCTTTAACCCACTTTCCACTAATTTAGAAAAACAAGATCAAACTATACAACACAAATGTTAATTAACACGCCGCTGCTTTACTAATCCTTTAAATATGAACTTGGGCAAGAAAAATC |
| 16 | lncM_002 | GCAAACACTGACAATTACTTTCCTGAATGTAGTTTACAGACTTAACTTACATTTAATACTTTTACACAGACTTTTATGACTTGTGTAAGTATTTTATTTTATCAGTCAGTTCATTCAGTGGTGGAGTCTGTCTGATTTAATTGTGTAGCACTTTTGATATTTTTTCCTCCCCAAGCGTGCAATATTATTATTTGAGAACAAATCTCTTTGGAATTTTATATCTATGTATACAGTCAAGCCTGCAATTATACATACCCTGTA |
| 17 | lncM_003 | CCCTCCTCAACCGCCCTGCTCCCCTGTCCGCTGTTTACTTTCACTTTCTTCCGCGACTCCCTAACGCTTCACTTTTTGTCCGTGACACCCCTCCGCCATCCGCCGCACCAGTCCGCGCGGCACCTCTTCATCCTCGGGTAACATGCCGGTGCCGTAATCCCGATCTGTTCCGGGACCTCGCAATTCACAAATTAAGCACTGCGCACACCCTAATGTGACATGGAAGTCAAGGGGGATCAAAGTAGT |
| 18 | lncM_004 | GAGCTCTTGAGCATTTCCCCATTTTGCTGCAGGACAATCCCATCATCCTCTAATCAAGCCTGTGGTTTAATCTCAATCCCGTCTCCATCCTCGTGACGCAGACTCTGGACAATTGAAGGATGTTCAAACACTTCGGCATCCACTTGAAAGTCCCGACCGCTCCTGCCAGATCTAAGTGCCGTCTTAAGACTGTAAAGACATTGATGGAATAGTTT |
| 19 | lncBr_001 | GTAGCAGCGCACTGTGGCTGTACTGTGTATTGGCACTGGTGGGAGGCATGCCTTAGTGTTTTAGTGGCAGGCGTGTCTTAGAATTTTAGCCAATGAAACTGTGCAGTGGACGGAGCTACACAACATTGTATATAGATGTTTAATATTCAACCGTGTGGAATTTTATCCAATGAGATAGCACAGTAGGTGGAGCTACACAACATTGTATATAGATGTTTAATATTCAACCGTGTGGAATTTTATCCAATGAGATAGCACAGTAGGTGGAGCTACACAACATTGTATATAGATGTTTAATATTCAACCGTGTGGAATTTTATCCAATGAGATAGCACAGTAGGTGGAGCTACACAACATTGTATATAGATGTTTAATATTCAACCGTGTGGAATTTTATCCAATGAGATAGCACAGTAGGTGGAGCTACACAGCATTGTATATAGATGTTTAATATTCAACCGTGTGGAATTTTATCCAATGAGATAGCACAGTAGGTGGAGCTACACAACATTGTATATAGATGTTTAATATTTAACCATGTGGACTTTTAGCCAATGACACTGCATAGTGGGCGGAGCTACACAGCACTGGATATAGATGTTTAATATTCAACCATGTGAAATTTTAGACCAATTGGAGTTCATAGTGGGCGGAGCTACATAGCAAGATATAATTTTGGAGCAATGAGATTGCACAGTGGGTGGAGCTACACAGCAATTTATGTATAGGTGTTTAATATTCAAC |
| 20 | lncBr_002 | TCCCAAGTCATTTTTTCCCCTCATATTTTATTAACAGAAGTTATTGATTCCAGCTGCACATATATGCACATATAACCAGTCAATCTGCTTGTTAAATATATAAAAATCTATCCCAACAATGGTGAGTTTTCTTCTGTTGTTTCTGTTTCCATCCAGGAGATCGATCAAAATCGGATTAAATCATTTGTGTTTACGCTTCAGCCATTGATGTAAACATGTAAATTCATTACCAATCCTTTATATTTGCCAACAACAGGATTAAACTGCAATAGGCAAAGGCAGCTAGATTAGATCCCCAATCAAAATCTCTAGCACAAAAAAAGGAGGGACTAATTTTGCCCGGAGAAAAGGTCTGTTTTGATGAATGGTCCCAATCAGCAGAAGCAGAATCTGGAAGTTGTGGAGGGATTATTGAATTTTAAGCCTTTGTAGAATTTTAATTAACTTTCTCTTTGGCCAGAAAACGGTTTTCCCTCAGTCGGTGCGGTTCAATAATCCAGCCACCCATGTAATAAGATGTTCATTACGAAATTGTACTTTTCACGCTTCGCTTTTATTCAGAGCAACTTCTGTCTGTTAGAGTGAGACTTGAAGCTAAAGAGAATATCAGATATACCTGGGCAAAGGTACAATAATTGGTCATTTTTTATTTTTACTGGTCAAAATAATTGGCCTCCTGCAAGACAGTTGGATTCTTGCATAGTTCTACATGGAATGACTATTGGGTCATTTAATGGGATCCTTTGAGATTTGGGATAAAACTAAATAAAATAAATACTGCTATTAGGTATATATTATCATACGAAATTTTGAAGTGATGGCATAATTAAAATGTACAAAGATTGAATCAAATTTTACATTTTAAAAGTGCATGTGTGGTGGG |
| 21 | lncBr_003 | CACACAGGGCTGAAATTCAGTTGTAGTAATGTGCATTTCTGTCTAAAATCACAACAGACTGGGGCATCTTACCATTGAAAGCAGACCAGACCAATCAGAGCAGAATAGAACCGTCTGACCAATCAGAAGAAAGCAAACCAATCAGACCAAAGAACAACTGTCTGACCAATCAGAACAAAGTAAACCAATCAGAGCACATTTGAACCTTCTGACCAATCAGATTAGAGCAGAGCCACCTGACCAATCAGTACAGAGTAAACCAATTAAAGCAGAGTAGAACCTTCGACCAATCAGAGCAGAGTAGAACCGTCTGACCAGTCAAAACAGAGTAGTCATCTGACCAATCAGAATAAGCCAATAAGAGCAAAGTATAACTGTCTGACCAATCAGAACAAAGTAAACGAATCAGAGCAGAGTAGAACCGTCTGACCAATCAGAACAGAGTAAACGAATCAGAGCAGAGTAGAACCATCTGACCAGTCAGTACAAAATAAACCTATGAGAGTAGAGTAGAGCCTCTGGCCAATCAGAGCAGAGTAGAACCGTCTGACCAATCAGAACAGAGTAAACCATTCAGAGCAGAGTAGAACCGTCCGACCAGTCAGAACAGAACAAGGTAGAGCTTTCTGACCAATCAGAGCAGAGTAGAATCATCTGACCAATCAGAGCAGAGAATAACTGTCTGACCAATCAGAACAACGATCAGAGAAGAGTAGAGTTAACCAATCAGAGCAGAGCCATCTGACCATTCAGTACAGAGTAAACCAATGAGAGCAGAGTATAACCGTCTGAC |
| 22 | lncBr_004 | GGATTACTTTTAAAAGTAACTTTCCCCAACACTAAAGGTACATATATAACATAATATATATATATATAAAGGTTTTGTGTGTGTGTGTGTGTGTGTGTGTGTTTCTCAGCTCACCCTTTCTTTAAAAGTGTGTAAAACAGGCTATTCCTCATTAGAAACAGCCTTATCAAAGCTCCTGCCTGCTCTGATACTGCTCAATATTCATCAGTCCAGAACTTCGCTTTCCTTTTAAAAATCACACAGCTCTCTCTCTCTCTCGTAGCATTGAATATGCAGGCTTCCTGATGAGGTTGCGTAATGATTATTTAATGCTGGCATAATTGCTTAAGGGCCTGCTTGTGCTCCTGTACTTACTGGCATGATACATCAGCATCATTACCAGAGCGATTGGCCCCTGATCACTTGCCTCTGATCATTGCCATCCATAGGCTTCAACAGGAAGCCACGACACACAGTGAGCTTCGTGTGTTAAACCGGACTGACGGCTGCGAGAAGATGAATCATGTATATGCACGCGAGATATGCACTTTCAAGTTCATCCGCTGGGTTTCTGGAGAGCGTGTGGTGCTAGAGAGTTATCG |
| 23 | lncBr_005 | TTTTGATTGTTGATATAAATCACTTATTGAGTCTGTCCTTACTTTTAATATTGTAGCCTGGTTTAATTCCCTCTCTGTTAAAAGCACAAATTAACTTTTAAGTTTGGTAAACTTAACAAGTAAAATAATTGGTGAGAGACAAACACCTCTCATTGATTTATTTATAGCAGCGATAGAAAGAAAAACCTCCACTATTGTGGAAGACTCTTTACATGGTTCCTTTAAGTTACTTCCGTCTGGTAGACGATATAAAGTCCCTTTAGCAAAAAAGGCTAACTACAAGAAGACATTTATTCCCATTGCCATTGTTTTTTTAAATAGGATTTTTAAAATAATTGTGTAAGTGTATTTATGTTTTGTTGTTGTGTGTGAGCCCAAAGACAATTTTTTAACCTCTGTGGGATAGACAATGACGCTTATTTGAATTGAAAAGTTGTTGGAGAAGAGATCAATGCCCCATAATGCAATACAAGAGCAAAAATAAAAGGAATAAAACCCATGAATCACAAAATACGGAAAACTGACAAAGTATGGATATTTTTCACAGTGTAAAGTTTATTTCAGATAATTGTGACCTGTGACCTTCAGTTCTTCGTTTCCTTATTTTGGGATTCACAAGTGTCATTCGTTATTTACTGTTATAAATTGC |
| 24 | lncBr_006 | ACCGGTTTTCTTAAGCAACTATTACTCAAAAGTTATGTTTTCAAAAGAAGACAACAATTTTGAGGAGAAACGTGTCGCATTTGCACATTTAACAGCTTAATTCAACCCAAAATCTGTTAGTCACACCGCATTCAAAAAACAAATGTGGAGCTACAGATACTTCATCTACAAATCCACAAAACAAGCAAGTCACATCAAAATAACCATAACTATAAATAGATAAATTAACAGGATAGATTTTGTGCAAAATCTGTTTCAGTAGCACAAATTTATGTTTTTATTGTATGTTTTTATACAGAATGGTTATGTGCGTATGATATGTAATGTTTTTGGTTAAGATGGTGTGTGTAAATGGTACAGACGTATTTAAGCATACAAAAGGTAATATCTGTGTGTAAGAACACACCAAATACAAAC |
| 25 | lncBr_007 | TGAATATTACCATCCCTAAATGACATTTGTTTACAACAAATTAGTTTTACTGTGTCCGTTAACTTAAATGAGCCTGTTTACACACACCAAATATTCAGAGGCCTTGTGAAATGTTGTATGGACTGATTTGCAGATGAAGACTGGCAGTCTGTAAGGCTGTGTGTGAAATCAAAGTCAGCTGCATCACTGCACACACACTCTCCGTGACTGAACACACACAGCACTTGAACACAACAGACAGACAAGCCTGAAATCTACTAGTTCAGATGATGCAACAAAGAGATACAAATATGTACTACAGTGCTTAGACACAGTAGGAATAGAGCAACGAGTTGGAGGAAAATAGTTTCTGCTACTTTCTTTTATAAGAACACTCCTTTTTTTGGAAATAGGCTAATTTTACAAGTAATATCATTGTAGCCATCATACGGCAGCAAAGTTCCTTGATTATTACGGCAATATTAGTGTATAGTTCCTAGCAATATCGACCTGGACAATAAGAACTTTTCATTTTCCG |
| 26 | lncBr_008 | GCACAACAATCTGCCTAGGTCAGTGTTCTCAGCAGTAAATACATGGAGAACTTTTAAACAAATGTTACTTTAAGGAAAATATTTAAACCATTATGGTAGATAGAGTTAAAAATCACTTGTTTAAATAAAAGTTTAAAGTTTTATCGAGATGGATTGTTGGTGATTGTATTGTATTTTGGCCAGATTGGGAAGCAATGAACAATGGAAAATTAAGTCCTGTTAAAAAAAGATATAAGACCTACAACACAATATTTCAGTAAATTTAAGACTTTTTAAGGCCTAACATTTTGATCTTGGAATTTAAGACATTTTAAGGCTTTTTAAGATCCTGCGGAAACCCTGATTTAACCTTTCTTAAGATAATTTGCAATCAATTACCTGTGCTAAATATGTATTTTTCTCTTGTCAATTTAAGGAAATATAATTTCCAGTAAAAATATATTTATAATAATTTTAAATACTTTTCCAGACTCTTACCAAGGTTGTCTTTACAAATATATTTACAGTATTAAAAAATATTACTTAAACTAAAAG |
| 27 | lncBr_009 | AACATTCTAGGCTACTTTTTAACAGCAGATGGTGCTCCAGGCTAGTTTTTAACAGCAAACAACACTGTAGGCTAGTTTTTTAACAGCAAATGGCGCTTTAGGCGAGTTTTTAGCAGCAGTCAGTGCACTTTTAGGCTAGTTTCTAACAGCAGATGGCGCTCTAGGCTAGTTTTCAACAGCAAACAACACTATAGGAAAGTTTTTAATAGCAGATGGTACTCTAGGCTTGATTTTAGCAGCAGACAGTGCGCTTTAGGCTAGTTTTTAACAGCAGATAATACTCTAGGCTAGTTTTTAGCAGCAGACAGTGCGCTTTAGGCTATTTTGCAGCAGATGCTGCTCTAGCCTAGTTTTAAGATGCAGACGGTACTTTAGCCTAGCTTTTAACAGCAGATGACACTCTAGGCTAGTTTTTAACAGCAGATGGTGCTATAGGCTAGTTTTTATCAGTAGAAGATACTGTAGATGCTTTACTTGACTGTGATTCAAAACTGTGCTAAAGATTGTCATGCTTAGGACTTAACCAGGTTTTT |
| 28 | lncBr_010 | GTTTCTGTCTCCTGTTCCAATTTAGTATATAATGCTCTTAAAACCAATACAAGGCCAATCCTGCTTGTGGAGGGCCATTGTCCTGGATACTTTAGCTCCTACCCTTACTAAACACACCTGAACCAGCCAACCAGCTGAATCAGCTAATCAAGGTTTTACTTGGCTTACTAGAAACTTCCAGACAAATGTATTGAAGTGAGCTAAACTCTGCAGGACAGTGAAACTCCAGGATTAGGTTTCGACATCCCTGGTTTAAACAATTAACTTTTTAATGTTCAGGTATATTACATGTTATCTCTACTTTAAAGTAGAGCTAACATAGGTAGGCTCTGTCAATCCAGCAATTGAAAGAACTTTGGATGCTGGCAATAAATGAACATATCACTAAAAGTTCAAGAAGAGTTAAGATGGAATGTATTGCTTTAAAATAGTTCCAAATAAAAGTATAAATATTCTGTGCTCAACTTAGGAAATATATTATCAAAAACTAATTGAGAATAACACCTAATATGATCAAAGATTATTTGTTTGAGTTTTTAAGAGAGAAATCAGAACACGTTTCTCATAATTTATAGTAGTATG |
| 29 | lncBr_011 | GTTGGTTTTATAAAGACTTGCATGATGGTTCCAAACACAATCAATAAACAGTGTGAAGTATAACAGTAATGTACAGTAAAATGATCACACAATAAGTTATTTTAACAGTTTTCCAGTGCTTTCATGCAATGCTAAAGAAACAACAGCAAAAGATCACAGTGATATCTATTTATACATTCATCAAACAAACGCTCAGTTTCTGCTTTGCAAGTATACGGTAGGAATCCCGTTAGCAACTTTGTTTCAAATGAACGCCAGGCAGCAGCACAAACTCACAGATAAACATCTGAGCACTGTAAACACTTCATAAATTGATAAATTCAGTGTTTTTATCTGAATATCTGCACATATTCACATATTTTTTTGTCACATATGCCATTTTGTTGTTGTTTTTGGACAAAATCTCAAAAATATGGGTTCTAAATAT |
| 30 | lncBr_012 | CAGATTTCATCTGACATATCCCATCCTCTTTTTGATTGATTTCAACCACTTCCCTCAGGATCTTGCGACTTGCTCTTTTTGGCAAGGACCAACAGACTTAAATTCTCATTTGTTCCTTGTGCTACTGATTTGCTGAATTTAATGGGAGGAGTCAGTGTTGAGTCTGAGTCTATTAATGTTGCTTAGTGTTTGTCTGAAGTGTGTGGTGTGGTGTGTATATATATGTTGTTAATCTAATGCAATGCAAAACAAATCGCCCCAAAAGGGTGCAATAAAGTTTACATTAACCGTAACAAAGCAGCGACACAGGTGAGGCTTAAGATGCTGTTTACCCCAAGCAGAACATGAGGATAAATATATCAGAAGTCAGAATTATTCGCCCCCTGTTAATTTTTCCCCCCAATTTCTGTTTAACGGAGATCAGATTTCTTTA |
| 31 | lncBr_013 | TAGAAAGGTAAGTAGACGTTTTTTAGAAGTGTGTAGTGAAAGTGCTTTTGGTCAGGAGGTTTTTGCGGTTTGTGTTGCTTTGGGTTTGAGTGTGCGATGTTGAGAGGGCAATGAGGGATGTTACTAGAAGGACAAAAGTTGGGGTTCAATCTTAAAGGGATAGGTCTTTTAAATATGTACTGACCCTCAGGCCATTGAAGATGACTTTTTTCTTTAGTAGAACACAATTAGTTTGCATAATGGTAGTCGGTCATCAATTGTTTTAGTAATTAAAAATAAAAACATCCAAATAAGTCTGAATCAATCCTCGTGACTCTTGATGATACACTGAGGTCTAATTGGTTAAAAAGCGAAATGGTTGCTCTGTGTAAGAAATTAAATGCTATTTACAATGGTAAAATCTAGCCCTTGGTTGCTTGATGACTAGGGTTGCGTATTAAAAACCGG |
| 32 | lncBr_014 | CTAGTGGTTTTAAAGCAACAGGTTTACTCATTTATTTTAAGTCAACTAGTGATTTTAAAGCAACAGGTTTACTCATTTATTTTAAGTCAACTAGTGGTTTTAAAGCAACAGGTTTACTCATTTATTTTAAGTCAACTAGTGATTTTAAAGCAACAGGTTTACTCATTTATTTTAAACAAAGTCCAGTTCGCAAACACCACATGTTTACAGACGGCTCTCCTGTGTCCGTCGTGCATATTAAATCACACTGAAGAAGTAGTTTAACAGCTCGCCTTTAAATATTTGTTTGATTATCGAAATCAATGCGCATAATGTTATGGTTCTTTTAGGAAGAAGTCATATAATTCAACAGTAAAACTCCTAACATCATTCATATCTACAGATTTAAAAGTGGAATTATTATGGATGAGACCAAATTGTGATTAATTATCATTTGTTGCAATGTAAGTTAAGAGTAAATGTGAAAGTTGAGGGCCACTGGATGGCAGCAGCAAATCATTCTGCATGATGCTTTTATTTTATGTACATTCATTTCTGTTTAATTTAGTGAGTACTTCTTTAAATTTAATGCACTTAAATCAAATCGTAGTCTTTA |
| 33 | lncBr_015 | GCAGTAAAAAGTTTTTTTTTTAAGTCAACCAAAACGCTTTAAAATAAACGGGTTTACTCACTTTTTATAAAATTAAATTAAATGAATAACTTTTTACAATGCAGTGCCTTTGTGATCAGCTTTGGGTTTAAAGGAATAGATGACCCAGAAATTAAAATTTGCTGTTAATTTATTGACCCTCGGGTCAGTCAAGATGTAGGTGACTTTTTCTTCAGTCAGATTTTTTGCTGAAACTGTGATTCATAACATGAATGATAAAACAAAAGTAATATCCACTTATTTGGATGTGCATTAAAGGTGATTTAAATAATCATTTGATTATTGTCAAAATCCACAGCTACTCATTTAGTTTTACCTGTACACTGTAAAACCCAAC |
| 34 | lncBr_016 | GCACTGCAGTACATTTCGTTACAACTATTGATAAAAATAAACACAACATATTCTATCAACGTTCACCATTTATAAACTCACCTATCATAGCTTTATTCTGTGACGCATAACTATGAGCACCAAATCTAAAAACTTGCACAGTCACTTGCTCACCAAACGAAAATATCGTAAACTATTAGCCTAAATCTTGATAATGCTACCATTAACCTGCTTTCTGGAAAACAAGCTTTTGTTCACTAAGGATTTAAAAAAGATAATACGGTAACACTAAACAATACGGTAAACACTTTACAATAAGGTTCATTAGTTAATGCATTTACTAACATGAACTAATCATGAACAACACATGTACAGAATTTATTAATCATAATTGAACATTTACTAGTACATTATTAACATCCAAGTCCATGCTTGTTAAC |
| 35 | lncBr_017 | TGTAAAAGTAATTATTATTATCAGTCAATATAAGTGATAATCGGTATTAGCCAATAAAAGCAATTATCGGTATCAGCTAATAAAAAGCAATTATCGGTATCAGCTAATGAAAGCAATTATCAGTATCAGCCAATAAAAGCAATTATCGGTATCAGCCAATAAAAGCAATTATCGGTATCTGCTAATGAAAGCAATTATCAGTATCAGCCAATAAAAGCAATTATCGGTATAAGCTAATGAAAAAGCAATTATCAGCTACAGCCAATAAATGTAATAATTGGTATCGGCCAATAAAAGCAAAAATCGGTTACAGCAAATAAAAGTAATTATCAGTACCGGATAATAAAAGCAATTATCAGTATTGACCTGTAA |
| 36 | lncBr_018 | GCGAGTATTCAAAGGGTATTTAAAAGAACTAGGGGAAATATCCTTGAACAGTCTTAAACTAAACTAAATGTACATGAAAATTCTGAATAAATTTTAGATTTGAAGAGAGATTTTGAGATTTTTTTTTTTTGTTGTTGTTGTTTGTTTGTTTTTAGAATATGCAAATTGGCATTATTTGATGCAGAAATACAGCATGTGATTGAGCTATTGTATCTCCTAATGGATTTAGCAGTGTCCACTACTTTGGGTGCAGATGTCCCATTTTAAAGACTTTTTTTCTGTGTTGTTTGGTAGGGTAGAGTGAGTTTGGACTTGCTCAGCTGTAGTGTGGACATGCAATAATGTTTTTTTTTTCAATCTATTTAAAGTGTAA |
| 37 | lncBr_019 | GCTTTTAATTCATGAAACAATCATCATTAATTAGTCACTAATTAATATTGGTCATGTTTATGACAGATTTATGACCAGTTCTGTAATTTCGGTAATGTCAAGTTGTTGTTACAAAGACAATATGTGATGTATTTGATTAAGTCAAGTTGTCATAACAAAGACATCTCAAGCAATGTCCCCTTTTGATTAAAAATCACATTACTGAGTGAATGACATACATTAGATTTCCTTCATATTCATGACATTTGAACTTATGGACACACCCTTCAACTAAAGTTTTACTTTTACCATTATGTCTTATCAAATGTATTATATATCTGTCTG |
| 38 | lncBr_020 | CAGAACACGCTGGATGAACGCAGCGGTGCCGCAATCAGCGAAATCCCGCATTCTCCATTCACATCCACACCGCGTCTGCTGCTGCCTCACATAAACAGAAGGCTATAGCATTTGTCTTAGACAGCAACTGACAGCATACAGCTGCAAGAACACAGCTGCAAGAACCGCATCGCTTGGACCTCACAGAGGTTTGATCGCATTTTAAGCTCCAATTCAATCTGGAGATCGTTTGGTTTTAAACCGAGCGCACGCACGATGGTTCATCTGCATCCCCGCCGAAGAAAACTGGTTTGGCTGTTGCCCGGTCTGCTTGGAGTCTGG |
| 39 | lncBr_021 | GTTAGCAGCGTCGCCATGGAAACGACTCCCTCACCGAGATCACGCCTCATCAAGGGCTGATCAGGGGCAGCTGCTGCTCGTTGGGAGGAGAGTATTTAACAGACGCCGTCCTCACAGACGACGAGCACGATGACAGCAGGCTACACGCTGAGTTCTCTCCTTCCACTGGGCACCCTGATCAGCACTTCACCCTGCACATTCCTTGGACACTGAATTCACCGCCCTGCACATCCTAGCACACTTATCCTTTGCTCCACTCCCTCAATAAAACACCCTACAGGGATTTATATCGCCTTTCAGTGTCC |
| 40 | lncBr_022 | GTTTGATCAATTTGTTTTGAAGACCTCTCTCGAAAGTGGAAAACACTTATTTTCTCTCTTTACCTTTGCAGTGGATTTTCTTTCAGGCGGCATGTGGTGTTTAAGTCAGAGGTCAGATCTCTCCTGCTGCCCGTCCAGTGAGGTCACAGCATCAGACTGCTACGCCTGACTGTCTTCTGGAGCCACCATCCATAATCTAAAGAACCCAGGGAAGAGGCCAAAAACCTGTCAGTACGACACCGGCTTCAAACTGCGAAGCTGAGGATGCCATAGGGAACAAACTAAGGCAACCGAGCAAATATGATATTTATACAAATTTTCAGATTAAAAGCAAG |
| 41 | lncBr_023 | CTGGTCATCTTTATTCTAATGCTTTAAAATAAGTCCCTTCTGCTCACTTCAAGACTTTATTTATTTGATCTTTAACATTATCAACATCTTGACAGCACCGCAGAACACGTCAGCAACACCCTAACAACCCTCTACAACACCCTAGCAACCATCCAAAACAGGAACAACCATGTGGAACAGGCTATGGACGCCCTAGCAGCATGAGCACACGCCAGCAGTGCATCTACAGGGGAAATACACGCGATGTTTAAACTAAACAAGCGTCATTAGAGTGAATGAAGCTGTCAGACTGAGGTGTTCATCTGCTTACAGTGGAGAGACAGCTCTACTGCTGATACACGGCAGACAGAACACACTACTGCCCCAGAGTGTGTGTGAACGGGAGAGAGAGTGAGTGAGTGAGTGTGTGTGTGTGTGTGTGTGTGTGTGTGTGTGTGTGTGTGTGTGTGTATGTCTGTGAGATTGTGAGTATGTGTGTGTGTGTATATGAAAGTGGATGCAAGTGTGTGTGTGTGTGTG |
| 42 | lncBr_024 | GGACAGTGCAGCGTGGTGTATCCCTGGCTCAGTACCATGTCTTAAACAGTGATGATATTTTCATCTATATGGGCAGTGTGTGGACGACCACTCCCTGTCACATGACATACTGATCCAGTGCAATCAATTTTCCCCAACAAGTAATTCAATCCACCTTTTGTACAATTTTAGTCAGAAATTCACAAAGAAAACATGAAGCACTGTAGACTTTCAGTTTTCGTAAACCTTTGATCAAACTCGAAAACAACTCGCACTCGTAGCAGTGCAGTGTGGCTGTATATTGGCACTGGTGGGAGGCGTGAGTTGACTCGAGGCTGCAGGCC |
| 43 | lncBr_025 | AGAAAAACGGGCGACTAGAGAGGAAGGCAAAATAAAGTGGAGAGAGCGAAGAATTAATGAAAGAGGCAAAGTGAGGGGGGATTTTAGGAGGTCTATGGATTCTGCTCTGGACTTTGGATTTTAAGCCGGATCGAACCTTGTGCTGAGCCTCATTGGAGGGCGTCCTGCGGATGCACTAGTGCGCGTGCGAGGGGTCTCTCTCTGCCCGCTGGGGAGGCGTGCCGCACAGACGGACCGCCGGCGTGCGCGTGTGTGTGTACGTGTCTCTGCGTGCCGTTTCCCCTTATGTCCTTCTTTTGGTGGCTGTGTGTGCACCAGGGCCC |
| 44 | lncBr_026 | CGTGCCTAGACGATTCAGAGTGTCATCTTCAACTTCTCCGCACGTTCACACATACTGTACATGTGTGTGTGGACAGCGACGGTAAATGAATGCTGCCCGGAGCGCGCACGAACGCGGTATGACGCCAAAAAAAAAGCTGTCTAGGTAGGCAGCTCACTAGATTGTCTGACGTCTTTTCCAGCCACGTTGACAGTCCCGTGGTAATAGAAACAAACAATAAGGATCCCGGCGCTGTTTCCTACATTGGCAGAAAGCTGGTTCGTGTTGTAGTTGTCGCGGCGCAGTCGGTTGTACGCAGCCGCTGCGTCCTCCCTCTCATGCCGATTGAGGATGTGAGCGCAGCCGCCGAGATGCTGGATCGCGGTGAGACCTGAAGCAGCCTAGAG |
| 45 | lncBr_027 | GCGCTGTGTAAGTGTGCGCGCGCGTTCCAGGGATGACGATGACGGTGTTTGGGGAGCTGTGGGTGTCTTTAAGAGCTTCTTTCTGCTCACTGCTGTCAGGATAGAGAGGCACGCGCCACCGAAGTACTCACGAGAGCAGAGAGACGCGAGAGTCCTGTTGGAAATACGTTTATCTTCATACACATCTAGGATATACTGTCTTCGGGAAACGTGGAGAACATGCAGTTGCCAAAGTCACGCGCTTTCGCTGTCAGTCTGACGCTTCTATCCGTCCTCAGCTCGGCAATGTGCGGCAGTCAG |
| 46 | lncBr_028 | GTTCAATCAATCAATCAATCAATCAATCAATATACACTACATCACTATTGAGCTAGATTAGATCCTGCTCTGTAGTATGAGCTAATTTAGTTCCCCAAATGGCGTTCCTAGAACTAAATTGTTCCCACTTCCAATACACACCAAAGAGCGGGTATTTCTGCCTATAGTTTGTAAATAGGTTCCTTGGTTGGGAAAGTCCTATAAAACTAAAATTATTTTCTTAACTGCTGAAGCACAAACACATCTGACAGTAGTTATAGGAACTATGAAAATATTCCTTCATTAGGTTTAATAGAA |
| 47 | lncBr_029 | CAAGAGGGCATTTAAGAAGAATCTTTCTTGGAAAGATGAGGCCGTTTCGGTGATACACAGCTATAACTCTCCTCCGTTCTGAAATCAGTGTTGGGTTTCTCGATGATGGAGCACCAATCACAAAGCCATTGAAGTCCTATTTCTGCTGGAGTCAAGCTTTAAGTTGAACAGCTACTAGGAAATACTGCAAACTTCAACACTGACAGGTGAAAATAAAAATATGTTCTGGCGCACTCTTGAAAATCCCATTGGAAATCAGTTAGGAATGATCCCGTAATCCAACAGCTTCCTTAATTTCCACGTGTGTCCACCCAAAATCCAAACGTGCACTGTGGAATCCTCAAAATTGATGATGTTAATCAAAAGTGGAAGCCATCATTATCCCAATCTCTCCCGCCTCGTTGTGCCTTCTTGACCTGCACGGCTCAGATCATGCGAGTTCATGTTGACCTCAGT |
| 48 | lncBr_030 | CTCAGATGAGCAGCTCTGGATATACACAAGGATTGTGTGTCTTGCCGATCAGGGGACGCCTGTGGATCATGTTTCCGTGAATACTGACAGAAATAAAGCGTATTTCACGTATCTGCTCTCTCACCGTGTCAGTCTGTATGCATTTGCGCGCGCACTGGGAGTTTCCGTCAATCTACGGATGAGACACCGCTGGAAGTCAGAGCTTCTGTGGCACAACTTTTCTGATTTATTCGTCTCAGAGGATCGGCAATTTTGTAAAACG |
| 49 | lncBr_031 | GTGTACTTATATATGCTGAGCACTCTATATATAAAATCAAATTAGTGATCATTTATTACATATATTATACTGTCAAAATAAGTGCACCCGTGTTCTTGTTCTTTGTCTTAATCTTTGTATTTATCATAATGTTTTTTGCTGTGTTAAACATTAAGTCACTGCAGTCTTCCTTGAGAGCATTTATTCTTATTTATATGTATGCCCAAAATTAGCTTGCCAAGTAGTACCTGACAAGTCTTAAATGCATGTCCATTTTGGCAGGTAACGTTATTTGGAATAGCAGAACAATTTATGTTTTTATTTAAAC |
| 50 | lncBr_032 | CTTTTCTTCTTTTTGCAATAAATGGTTTTATAAAGCTACACCACATAAAACAGAAGTCAAGATCCATTACGTTTGTAATATTTTTGTTATGCAACAATGTTGATACACATTTCCATGTACTGTATGTAAAGTTACGGCAACAGTGGAATTACTCAGTGATTCACAGACAAGTGTATGGTTTCCTGATCACCCTGTTTATTAGACTTGATTATTCTTTCATGGCTTGATTGATTTCGTTGTTGAGTTTGTTTGTCATATAATGAGTGAATCACTAAGTGCTTTTCTTTTCCTTATATATGCCATTTTTTTGTTAGATTTGTCC |
| 51 | lncBr_033 | CCAAGCTTTTTTTTAAAGTAACTGATTTCAGTCATAAACAGAAGATGCGCTGCTTAAATTAAACAGATTTTTAACTAATCAATTTGTATTCATTCATGTTTTCAATTGACAAAATACTTTTTTTAGACACATTTTAACTAATCAAGAACAAATATATTGTGGTAAATGTAAAATTTTTGCAGTGCAGACATTGTAGACAGCATTCATTGTTAGACTTTATGTTAAGGTGTCTTTGTTAAATTGTAACAATAGATGTCTCGTCATCTTTGCCACCCAAATGTACACAAGGATTTATTTCTTTTGAAACTTGCTGCATTTTTCCACCATGTTTAGATTGACAGTATATTATCGGCCTTGATAATCATCTGTTTTTAAACCGATTATTGGTCAATATATATCAGTGAATGCTTCATTAAAAGTGCTTTAATACAGTTGCTGTGTTCATTATTGTACACAGTCATTGATTATAGTGTTACCTGCAAGTGAGGCGTAAAAAATTTCTCATGTCAGTTTCTATTGTCATAATTATTATGTGATAAATAACAAGTCATATTTGGTGCTTCTTTTCCTTTAACAAGTGCTTTCTTCAAATAATAATGATAATAAAATATTC |
| 52 | lncBr_035 | CTTTTTCTTGATCCATCTGCTAGAGCCACAAAACCTCAAAAAAGTGCCAAAGAAACCTATTGAGGTGGATATGCAAGCCCCCTGGGCCATTTCCAGGTGTCGAAACGTTGAAATTCCACTGCCCACGAACAGCCTATTTCATACATTGGGATAGATTGTCTGTGGGATGTCCATATCTCAGCTTCTGGACACAATAGAGAGCCCAAACCTGCGTTTCTGTGCTAGTCTGGACACGCTCTGTCTAGGG |
| 53 | lncBr_036 | AAAAAATAACCTGTCCATTGATGTATGGAAGATTTGTGAGGTAAAACTATTTGGGTTTTAAAAAAAAAATCATGGACTATTGCTTAAACATAACTGGATAACGTGTTCTGGATCACATTTGTCACAATAATAAAAACTTGAATATTCACGTGAAGCTGAGGTTAAGATTTGAGAATAATTTTAGACTATATACTGTAAATCTGACCTTTCTTACCCTTGTGAAAAAGAAATGTACTTATAGTATATGTACTGGATGTACTTTAGTGTACTTCAAAAATGAGAGTATACTTTCAATTAACCCAATTAGCCCGCCTTTGAATTTTTTTTCTTTTTATAATGATTCTCAAATTATGTTTTAACAGAGCAAGG |
| 54 | lncBr_037 | GCTGAACAAGATGGCTGTGCGTCGAGTAACGGGCGGCTAAAAAAACGGCGCTAATAATCGCACAAAACCCGAATAACAGCGCATTATATTGCAAGAGGGGGATTGAACCATCCCCGGGTTGAATTAGCTGGAAGATTCGGAATAGAAGCTGTAGTATTTTTTTGTATTTTTGGGGTTGTAGCTTTGGTAACTGGAAAAGGGGAGGTTTCTCAACGCATAAAATCCACTAAAAACGTTTTAACAGCAGTTTATTTTTGTAAAATAACAGAAAAGCGCGCGGAAAATATAAATAAAGAGATGTCACTGCTCTGTGTTGGA |
| 55 | lncBr_038 | CTTTGCCCTAACTGGACTTGTGGATGAGCAGTGCTCTAGTCCCGCTCCTCAACTCAACTAATAAATGACTGGAAATAGTTATAAGGCGCTGTCGCTCGCGTTATGACAGACAAAACTACTCTGCCATGTCTCTGTCTGTGCGGAATACATAAAGGCTCTGCCTTGAATTAATCACTCGCTCTTTAATGGATCTTTGAGGATCGCGCTTGACGCAGTGAGGAGCTGCAGCTCTTTGTATTCCGAGGCCAAGAGGAACCTTGTGCTTTTTTTGCCCCCGTCCGGTATCTACTGCCTCTTAGAAAATGTCAAGGGGGTACATGGTGACATAGGGACCCTGGTTTGGTTACGGAGAGACTCTTGCTTCGCTCTGCGAAAGAGTTCAAGACTACAGCGGCACAGATGCGGTGCGTTTCTTTTCAACCAAGCGTCAACAAAGATCAGTTCAAGGGGAGATTCCGAGCATCATTAGTTCGGT |
| 56 | lncBr_039 | TGAATATTTTGTCAATTCAAATGACTAGCGGCTTCGACCTGGAAACAGTATTTCATACGTCACGGCTTAACAAGCGGATGGGTTTACTCAGCAATGTTTACTAATCACTTTTCTGGATTTTGACTGTGGTTTGCATATCTTAGAATGCTCAGCACTAAACATTAAAGCTATTTAGATGAGCCTGGTCACTCGCCAGATGTTGCTTTTACAGCTGGTTAGAACAAAAAACTCAATGTAATCATGCGTCACATCACGTCTGGGTAGGACACCGTGTGTTTTATGCAGTTGTTTTTGTATCTCGGTTGCATGTGTGTTGCATTTCTG |
| 57 | lncBr_040 | ATGGCAGAGTTTTCACTTTGGCTGAACTATCCTTTTAATTCTCAATACTTGCATTTAATACTTGTACTTATAAATGTTTAGGGATTCTTTAAATGCCTTATAGTTTCACTGAAATCCAGTTCACACATGTGAAAGCAAGGGTAACACTTTACAAAAAGGTGAACATTCGTTAACGTAATGATAATAAATCATGTTCCCGAAGCCATTATTAGTTCCAGTGCACTCGCCAGTGACGTCTTCATATCATTTAATAAAC |
| 58 | lncBr_041 | CTTGTGTTTGTGTGTGTGTGTGTGTGTGTGTGTGTGTGTGTGTGTGTGTGTGTGTGTGTGTGTTTGCTTTCAGACAATGAAATTGGAGGAGGTGTAGTGACCTCTCATCAGTCGTCTAACATTGAGGACCACGGGGAAAACAGAGGTCTGAACCAAAGACTAATTCAACCACCAGTTACATCAGATGGAGGTGGATGTAGCAGATGAGAAGCGCCATTGCACTCGCTCGAAAGTTCCCCTGGAGCCGGCCATTCAAGAGCTGTT |
| 59 | lncBr_042 | ATATTTTAAGAGCCGAGAGTGAGCCGAATACTACAGGGATTATATCAAAAGCAAGACTTCGACATCTGGGGGCGACGCGTCAAACTCACGCGGTGCTGAAAGCGGCGGCGTTCGCTACAATATGAGCTGTTTTGAGCCTCTGATCTGATGAACGTGCGTGAGGGGACTGTTAATGTGCCTTTAAAAATACGGAACCATTAGGCGATGTGTGTGAACCAGAGGAAAGAGCCTCAGGTGAATAGAGGATTTGTCAGAGAGGCGTGTGAGCGTGTCTGCTTAAAGCAGTCCTCAG |
| 60 | lncBr_043 | CACACACACACACGCACGCACGCACGCACGCACGCACACACACACACACACACACACACACACACACTCTTCCTCTGGTATTAATGCTTCCTAAACCAACAGTACTGTACGCTTTCAGTTCTTCAATGATGAGTTCAGAGTTTGTTTTAAAGCAGCGGTTGTAGATTTGTAAACGTGTGTGTTCTGTGTTTAGTCTGATTTGAAGAGCTCGTAGTCGTCTCTCTGGACCAGCTGAATGTGTG |
| 61 | lncBr_044 | TATTTATTCATTTCCCTTCAGCTTAGTCCCTTATTTTTCCAGGGGTTGCCACAGCGGAATGAACTGCCAACTATTCCGGTGTTGTGACATTTTATCCTACCCATCAGATTATGTTGAGTATACCAACACTGTATTTGAATAGTTCTGAGGTTGGGGTTAGGGATTAGGTAGGTTAGGGCAAGATTACAGCTTCATATCACTCTACTCCACATTCAAATTTACGTTGGTAGCAAAATTTGATGGGTAACAAATTGAGTAATCAAAACGTGCTACCTACTTTTAGAACAGGAGGTAGGACAATTTGACAAGC |
| 62 | lncBr_045 | CTTCAAAGCCAACTCATAAGGCAAACCCCGGCCGGGGCGAGAGAGGAAAAGAGTCGATCGGTCCGTGCCCACCACAGCGATGCGGGAACCACTGACCTCCGGCTGCTGATGGTCAGAGCACAGTCAACAGGTGAGTAAGTAAGTGGCGGCACTCAGAACATGCAACGATGAATACAGGAGGGATGATCGACAACCAGATCGAGACTAGACGAGACAAATGAGGATGAGATCTGATGTAGAGGAAAGACTAATACACGGATTGCTAACGCACAGAGTAACAATAATCTGACAACAGGAGGAGAAATAAGGGAGGTAGATATAGGAGTGTCA |
| 63 | lncBr_046 | CAAAATTTCTGGAGGTTAAAATTTCGCCAAGAACTTTCTAGAGATTTTTAATAAGGTTTACATCAGGACTCTGGGTCTGCCATTTCATTATTCAGCTTAAGGAGCTGCTTTACATGTTTTGCTGTGTGACAGTGCTTTCATTGTCCTGCATGAAAATGCTGTCTGACTGTGTGATGAACACAGGGAAGAAAACACACTACCCAAG |
| 64 | lncBr_047 | GTTTTGAAGAATGCATCCGTGTCCGAATCATTGGGTCCGTCCTCCGGCGCTTCAGCAGAATTCATGTTTTCCTCTTGTATCTCACAGCTCAAAATAACAAGAAATATATCCAAATTCAGCGCTCCATCATCTCAGTGTGTCTGTTTTTGACGGCACAGCCCTCAGTCAGCGCGACAAAGCACAGCTCTGCATTGCCTTCTATTGTCTCCAG |
| 65 | lncBr_048 | TGTGTGTGAAAACTGCCAGTTTTTATAAACAATAATGATAATTTTTGTAATTAGTTAACTTTTTTTAAGAAAATGTTTATACTTGGCTGTGATGGCTATGATCTCAACACCTTAGAACATAGATATTAACATTTGATGTTGCCTATGCTGTTGTTGTCTATTGGCGTAGATATATACACTAGATATCGCATTTGGATCCTGATCATGC |
| 66 | lncBL_001 | GTTTAACGTGGGCTTTCTTGTTTAAAGAGTCACTGATACTTTGTTATATACAACATTATGTCAGTCCCTAATAGGGCAGAGTGGTGACAGTTGCAACACTTTTTTTTGCATTTGCTTCAGTTTCTTAGAGACAGTTATTGTGGAAACCCAAACTGTTAATACTGTTAAGATAACACATCTTTTGGACACACATTGCTATAATATGTGTACATATCATTAGTGTAGTCCTTCATATACACACGTATACTCAGTATGCTTACCTTTTCCACAAGCCGTTTGGGTATTACCACTTCTGAGGATGAATAATTGAGTGTACCCCTTGGATATTAGTCAAACATTTG |
| 67 | lncBL_002 | GTTTCACTCCAAGAAGGCCAAACAACACATTCAAAAACCCCATAGAAAGTGTCGCACCGAATGAACAACACAGGAATGAACCGAGCGCATAGACTGACAATTGACAGTCAAATTCGTTGGGCAGAAATTTAAATGCGAAAGCTCCTATTGTGACACCAGGAGCCGCGTAACATGCGAAAAGCACATCTGACCACGTCCTGATGCTGATAATCGACCCAAAAACCAGAGCTGAAGTCAAGAGTAATGCGAA |
| 68 | lncBL_003 | AGTGTACATTCATAGCAATGCAATGTATAACGGTGTAAAAATACTGATCAGTTTCATATCAATGCAGAGTGGCACGTAATAGAAACAGACCATCTAAAATCTGTTATTCCCTGAAGGTTTAGTCTAGAAATAGCTTTTTTTAGTTGTTCCCTGCAGTGGTCTGGAGAAGGTCAGTCTGCTTCACTTTCTTTCTGCCTCCATTCGTTCATCCAGATTTTTGACGTTCTTCAGGAACGTCATGACGTCGGTTGCCTCGTATTCCTCACATGTTCTGCATTCGCACTTTCGGTTCGGGCAAGGCTT |
| 69 | lncBL_004 | CGCGTGTCCTCAAATCTGCCTCTGAAACACAATGGGATCACAATCACTGCCTCCTTTGGGACAGCAGAGAACAAACTACACAGACGACAACAACAGAATTTAGGTCATTACAGAGGCATATCGACTCCTGCTGAATCAGCTGCAGCTTAAAAGAACAAATAGATGTAGACAGAAGAGTAACGGGCCCTGAGGTTTGACTGCATTACGTTGTTGTGTGTAATGGGAGAGTGTGTGTTGGACCGCTATGAAGAAGATGGGTTTCAGAGTTGTGAAGGGTTTGACAACTGGGCCTCATTTAGC |
| 70 | lncBL_005 | GATTGTCCTCAGGTGCGATGTGTTGAGGTTTGCACTGGACAGCAGAGAGGAGAGCTCAGTCTACAGCAGGGAGATCTGATCAATGTCATACAGAAAACTACTGATGGGTTCCTGGAGGGCCGTAGAGTACAGGATGGACAGCGGGGCTGGTTCTCGGCCTCATGTGTAGTAGAGATCACTAATGAACATGTACAGAGACGTCACCTCCGTCAGAGGTATCATGTCCTACAGACGGCCACACGCCTGCTGAAACAGCGCAACAGAGGCCTTGAGCAAACAACCACAAAATGCCTAAAATGAGAGACC |
| 71 | lncBL_006 | TCTGCTCCATCACTGCATTTTTCTTCAAGAACAGGAATATCTTGCGTAATCTTGCAGTAGTACCATCCACTGTCATTGGTCTTTGTAGAATTAATCTTGAGTATTTGCCAAGTGTACACTTTTTCTTTTATCCTGCTAGGAAGAGAGCCACAGTCTAATGTTGGTGTCTTGCTGTAATGCCAGCTCACTTTGTGGTTTTTGCTTGGCTCAAAAGTGCAGTTGATTTTGACTGATGCTCCTTCACAAACATTAATTTGTT |
| 72 | lncBL_007 | CCCCCTTTAGAAATCTTTAGTGTGCATTTACTGAGTGATTAAGAGTTAAGAGCGTTTGAATCCACAGATACCGAGGCAGGTAAACACCAAGTCAATGACGTACAAACCCAAGTTAACATAAGTCATGAACGTGACCACAAACTGGATGCTCCATACGCAGCTGTTCCCAGGACAGTCTTTAGGCCGCGGGTTTCCTCTGAAGCTGTAGATCGGCCACAGGATTGCTGCCGAA |
| 73 | lncBL_008 | TGATATTAAAGTGGTGTTTACACCATTTCTGCATTTTGAAATCTCGGCAACTGGAGCTGGAGCTGGAGGTTTGTAGTACTCAGCATGTTGTTGCAGTGTTTACAGTGCTGATCCTATACTTCTGGGTTTCTTCCCACCCAGCCTTGCTTTCGTGTTTTAAAATGGCGGGCTCATGAAATAAGCGGCGGATGTATGATAGGGCCCAGAGTATGTGTGAAACAGGCGCA |
| 74 | lncBL_009 | TCAATATGTGAAAAAAGATGGAGAGTGCTGTGGGTCGTGTGTACAAGTGGCCTGTATTTATGATGCTCCAGACAAAACCACACATGTCCTCCAGGAAGGACTGAGGTACAATTTCACATGCTTGAATGTCACGTGTCTGAAAAGGAATGACGTGTTCACGATCAAGGAGAGTTATAAAAAATGCCCTTCCTTTAATCAAGACAACTGTGTGGAAGTAAG |
| 75 | lncBL_010 | TTTTAATTCAGCTGTACATGTTTGCCCTATAGGAAGAGCATATGAAAGGCTGAAGAAGCCTGAAAGTGTCCTCTATTTGAGTGAAGCTCGGGTGTTGATGAAGTTCAGTTGATCAAGGATAATGTTGTGCTGGAACTGCCGATATCCGGTGACATTGAAGACGGGTTGTTCTCCAGGAATTTATATCCATCTTTAAACAGAACCTTGAGGATGTCCAGAACGACCAA |
| 76 | lncBL_011 | GGGCAGAAGAAAAAAAATACAAAAGCATGAGACAGAATAAATACGTACAGAACTTAAAATGATATCGACATGCAGCTGAAATCAAGAAAGCAAAGAAACATGGGATTTAGAGTGCATACTGTATGTAGTGTTCAACATGGGCATTACAGCCAAGGGTTGTGACAGGACGTCTGTAGATGTCTGACAGAAATAAAGTCTAACAAGTGAAACTGCTGATAGTGTTTGTTGAGTTATTTAAAGATCCTCTGTTGAGATTTTG |
| 77 | lncBL_012 | TCTGGGTTTGTTTTACTTGAGCTTTTGACTTCACAGCATTAGTTTTCCTCTTGTTGTATGTTGTGATTGTATGTTATGGCGTGTGTAACCACTAGATGGCACCAGAGGTTTGGAAAGGGTTATGTGAGATGAGAGAGTGCTAGAATAAGACAGTATCAGGCAGACTGAACTTGTAAATATAGAAGACACTCGTCCAACTCGTCGTCATTATTATAAGACGCAAACATGAGATGGTACCAGAGCATAGCAAGACAAATGCC |
| 78 | lncBrBl_001 | CTTAAACCAAAGATTTGAAACAATGCACAGAAGTGTAACGTTTTAAAGCTAATTGTTATAGTAGTCTAATTTTAACAGCTCGTGATATTCAATGTAATCACTCTTGAGACCTGCCAAATGTTGTCATTAACCCTTGTGTGCTGTTGGGGATAATTTCGAGTCTTAATTTGGCCACAACTTTCACCGTGTCAGCAAAATTTAATGCTTTTTGG |
| 79 | lncBrBl_002 | ACAGGAAGTGTTTTCAGACTGCTCATTTCTACATCGAGGACAGCACTTCGCCCAGGGTCATTTCCTCATTGCCACTGCTAAACCCTACAGGTGAACATGAACCCCTGCCAGTAAAGCAATTTGTGAATCATGTGACAGAGCTTCACAACACCAGCTCGTTTTCCAGAGAGTTCGAGGAAGTTCAAACGTGCACCGTGGACCTCGG |
| 80 | lncBrBl_003 | TGTTTGTAAAGGAAGACCTTCGACAGGAAGTCAGACGTGGGTCTCCGGAGGCAACAGCTCTGCTACATTTACCACCTGCAAGTGTAGCAACCAAGTGCAGTCATGTCCGGCACAGGGTTACGATCCACCTCGCAAAAGATGTCCATCATCCCAGATCGTCTATAACCTTACGGGCGTCAACATTGAAAACTACCTGCTGGCTACTGCCAATAACTTCATCAGAGACAG |
| 81 | lncBrBl_004 | TACAGTCTGCGGCTGTTTTCTCTTTGTCTTTTATCCCGTCTGTGTTTTCTGCGCATCCTCATCCTGCCCGCACCATCAGCAGCGCTGCATCACATCCTTATCGCTTCACCGTCGGATTTAATCCGTACTAATGTATTCAAAAATCGTCCCCGTCGCCGTTTTTGCTCGCATCCATGTGCTCAGCAAACAATCGCTGCTCGTGAGGCTTTGATATTCGTTCTGGTGGCTCCACTAATCATGTCAGACTGGAAGAGAGTCTCATTGGCCAGCA |
| 82 | lncBrBl_005 | ATGTAGTAAAAATAATTTGCATAAAAATTGGAAACATCGAAATGTCAATTATCAAGTACAAATTTATTGTAGGTCTTTCCTTAAACCTCATACAAGGTTGAATACACCACAAGATGAACCGTTTCAGTGGTGTAGGTAGTAACGTGATCGGCACAAATAATTGGCATATTACTTGATTGAATGGGTTTGTGTACAGCTTTTGCCGAGCTTGTTCTAAAGGTGGAACTGCATGATGAGTAGGGCTGTGTGATTTGAGGAAAATATTGATATTAATTGCAGATTTTGTGATTGCACTTTGATTTAGTATTTAATTCAAGCTGATTTTAAGCTTCTGCTCAATAGCCTGTAAGCTCTGGCAACAATTCTG |
| 83 | lncBrBl_006 | GAAACACTATCGTATTTACTATAGTTTTTATATTGTGGCCTACTACCCAGCACTAGTGAATCTTGTACATGTCATCAGCACCTTTTAATAGTCATTTTCTCTGTGTAACACTGCAGTGTGTTTGTGTGCGAGGTGTTTCGTAAATGACGGTTTACTCGTTATAACGTGCAAATGAATGTGTGTATTTGTTCACCTTACCGACCCAGATTGCTCCTTCATGTGGTCATCGCCGCACTTTCTGTATCTTGG |
| 84 | lncBrBl_007 | CGGGAGTCTCCCATATTTTAGACTCATCTTCCGCCACCCTCACGTTTTGTTATTTCTTCCGGAAAACTCCCGTAATTTCAACCATACCACCCTCCTGGTCCTCAGCATTTTGGTACGGTTTGTGAAACCCCTGGGTCGTCAACAATGGTATAGGATACGATTGCAGCGGCCGCCCAAATCACACATCATAGTACAGTTACTAACACCACAGTACACAGTACCCAGTTCTCAACAGTCAACAGTGTTCTTCTGACACCTCACTCCCAAACCAAACCCTCAGGTATCCCATATTTTCATGGATTAATGTTGGCAGGTATGCCATCAACC |
| 85 | lncBrBl_008 | GCGAGAGATACTTAACGAAGAGTGATGAGGCCAATAATTTGCTGATGTTGGAAACTGAGGAGATGAGACTGCCGCTAACAATACTGCAGCACTAGAGATTTAGACACACACCTACACACACCCACATGCAATTGCACAGGCAAAGAAATGATGCAAAATGATGATCTGCTGAGGATACGGCGGTAAATTTCTGTTCGGCTTTTAAAAGGATTTCTAGTGCTGATAGAAGAAGGATCCCGTTTGGATTGAGG |
| 86 | lncBrBl_009 | TGTTCTCGCTGCGGATTTCACCTCACCGCCGCAGTCGCGCTCACACTGCGGATGATGGGCACCTTTACAGGAATCCCAACTCTCTTACTCCACAAATACAGCCAGACGCCGTTTTAAAACACTCGATTCTGTGCGTCTTGGCACTGGGAAAAAATCCACTGCGTTTAATTTTCCCAGGAAAATAAGACAGACGAGCTGCAGAATCCCATTCCGGACTAAACTGATGCGCTGGTGACCGTTAAAACTGCCGCTCGCGCTATAGTTTCCATCATGACGCGCTGGTTGTCAGAATTTAAAGCGCGTTAAAACGCCAACCGGAATTCCGCGCGCAGGGAACGAGCGCGCCGTGTGCGCTGCAGTCAGAGCGGGCGTTTTGGAGTTTACTGGTTCGCGGCTGACTCCCAAGTATACCAGAAGAGAGGCG |
| 87 | lncHBl_002 | AAAATATCTACCACTTTATCAAGAAGGCATCAAAATCTTACCGAGCAAAGACAAGACCATATGCGATAAAGAACGCACGCACACACATACATAATAGTTTGTTTACTTTTCTCTCTCAGCCTTGCTTCATTTTTTTGTCATTCTCATTTCTTCTTTCCATCATCAGTGATATTAAAAGATATCACACCTTTGTACCAAGAAAGAGCTAGAATATATAATTTTA |
| 88 | lncHBl_003 | AAACAGCTGGCTGTTCAAAGTCCAGTCAAAGCCATGTGGAGGCTGCAGAAATGCAAGTCAGAGCGAACACCGTGACTGAAGCTGAGGACATCTGCTTAAAAGAACAGATCACAAACGGACATACATGAGAATTAATGGGTGGAAATGTGTTACAGCAACATGCTGTATATGGGACATCAAAAATCTGAACCACAATCTAGAACGTCATTAAATCCTGAAAAGTGACGGATTTCCT |
| 89 | lncHBl_004 | TGGAACATCAGGAGAGGACCACAACAAAATAATACACTGTCTTGCAAGATAAGTACAAAAGACATTGACATCCATAATATTTAGTTTTTCGTACAATGAAAGTCAGTGATTACAGGTTTCTAACATAACACATGGATTTACTCTCACTTATGCATAAAGTGAGTAAATAATGTGTAAATGTTCATTTTGGGCTGAACTATCACTGTAAATACTGTAGTGTAGTTTATATTGAATGCTTGATTCTGATTGGCTGATGATCGTTCAAGGTTTGCTGTTATTTTCAGATAAACGCACAGATAAAGTCGTTTGTGTGAGAAACTAATCTAACACATTTTCAGTTTGTGCAGAAAAACCTGACAAATGTCTGAAACAACATCTTTATTGTGGATGTAGGAGCTGTA |
| 90 | lncHBl_005 | TTCCCGTATGTGACCACGTGGTCCTTATCTTTCCAATTAGCTTTAAGATGAACATCTGTTAGGGGTAAGGGGCATGGGCTGTGTGATATATGACTGGAAAGCAGCAGGATGGTTTTCACACCTGTTGTAGTATGTGTGGTTTAGATTGAACTAGAATTGACCTCGTAAGCAGTACAACCCAGATAAAAGGATGAGCACACTTTGCTTCATTGTCTCACTCTGCAGAAACTGTTGTTGCTGTATGACTG |
| 91 | lncHBl_006 | CGAAGAGCAACTCTTTATCCAGTCAGTGCACAGAGGAAGAAAAGGTCCCAATGCCAGTGCAGGAGATGTGTGGGAAGACCGAGAAACTGGAGGATGTCTGAGAGAGGAGAAAGAAGACCACTGGTGCGTCTGAGTATTGAGTATTTACTCTAGTACAAACCTGCTGCTGCCATTTACTCTGCACTGAGGTGGATCATCCAGATTGTTACGAGAGAACAGAATGACGGTCCATCCTGAGAAATGCTGAGACGGAGAGAGATTTGAGCTCCCACTTCACATAAAGACAAACACAACTTTTCACATACAGCTCA |
| 92 | lncHBl_007 | TTCTGTGACAGAATGGCTGACTTGCTCAAACAAATTCTAACGCAATCACTGTGGGTCTTCTAAGTTTATGAGAAGTTAAGCAAAGCTGCGCACAAGCTTGAACAGACATTTACTTTTGGATTCGTCTGTAAGACAGCGCTGGAGGGGGAAGCATAAGGAAAACAGAAACACAAGGAGGGGAGGAAGATAAGTGTCAATCTGTTCAAACTTGTTTGGGAATTTGAGACGGGACGGAGGAGGGGACAAGAGAGAGAGAAACCGAGAGAAAGACTGAAAGGAACACTGA |
| 93 | lncHBl_008 | ATGTTTTATTCGGTGTAAGTAACCTGTGTGTCTTTGTTGTTACACTCTGAAGCTTTTGAATTGCACAAGAGAGACACTGAAACATCAGGGAAAACTCCAAATGCAGCACAGAGTGGTTCAGTAAATGTGGTGTTGAATGTGTGTAAGAGCTTGAGTGTGTGCGTGTCAGAGACGCTGTAGAAGGACAGAGTGCCGGCAGACCGGTCCAGATACACTCCTACTCTGTTAGAGTCAGATTTTGAATATTTATCTATATCTCTATTCGTATTTTTG |
| 94 | lncHBl_009 | CTGTGAAGAAATTTAGCAAAATTCACTGTAAATATATGGAAGCTCAAATTGTGAAGTGAGCCGCCTCGTCAGTGATGGCTTTACCCATTGTAACAGAATGAAATTTCTGTCACTTTTTTTTATCCTTAGAATAATGGAAATAAAAAATGCTGTTTAAATGGAACTACAGGGCACTAGAGGGCGCTGGTCAAACACCTGGAATGTATAATAGGTGAGTGGGTGAATGAAGAAGTAGATTCAATTGTGGTAGAGGGAAGACGTGGTTCCTTGGAGCTGCTTCAAACTGTTTGTATAGTGAAGTCCTAGAAGTGAACCCTTGCGTTTAAAGCTCAGCCTGTGAGTATGCTTACATTCTTTCTTCTTTCAATATGTTTGAAATTCTTATGTTTGTTTTTGGTGTTTTTTTTAATTTTGTTAGAG |
| 95 | lncHBl_010 | AGACTGAGTGCAGAGGATGTTTATTGACAAATGTGACAATGTAATAGGGGTTAGGTGCAGAACAGTTCAATGGTGAATGTATTGGGTAGTGCTGGTGAATCCCGGATAATGCAGCCAGAATCCAAGTAATGCCGGAAGGGTAGGAGACTGAGCAGTGGGCAGGGTTGGACAAAAACAAAGAACAGAGACAAGGCACACAGAACAGACAGGGGGAACAAAACAAAAC |
| 96 | lncHBl_011 | ATTTTGTGTTAGTTAACATACTTACAATAACTTGTAACTAGCGATATGGGTAGGGTTAGCAAGAGTCTTAGAGTCCTGGTCTCGTAGCAGAACCTTTGGCGTATGATTTTGGTATTTAAACTGCCTCTTTGCACAATAATCTAGATTAGTTTAGTTAAGTTTATTTCATATATTTGTACAGCAATGATTTACAACATGACATGCTTTGCCAGATTTAGCTAGAAAGCTAATTAGCATCTGTAGACCCTGGGCAGGATTTTGATTGTTACAAAATTAACCACA |
| 97 | lncHBl_012 | ATTCTGTCAGGTTTAATCTCTCCATCCGATGCCTGGAATACAGTGTTGCAGTCCTCAATACATGTCACCACATTTGCTCTGAGAGAATTACTGCTAATCACATCACCGCCTTGAAGACTAAACAGTTGATTTGGGCACTCTGGAAAAGAGGCTGCATAATGAACTGACACACATAGAGAGATGCTTACATTCTGCAGTCGGTTCAGGTGTTAA |
| 98 | lncHBl_013 | AGAAGATGATAGGAACACTTCTTCACCCATATACGAGAATACAGAAAGTACCCCGCTGATTCACAGTGACGACTCAAGTGAAGGAGTCATACAAAGACAAGAAGATGTACCAAGACATGAAGACAAACTAAGACAAGCAGACAAACTAAGACAAGATGCTTGTTGTTTTTGTGGGGTTTGCTTCTTCTTTTGTTGTGGTTGGCTTTGTGAATGTAAACCTTATA |
| 99 | lncHBl_014 | CCGGGCTCTAAATGCGAAAGAAAGACTAGTAAATGATAAAGCTAGATCATATTTTCACTTGTTTACACCTTGATTTTCTGATTCTGAGTGTGACCACAGCCATGATTATTGTGTAGTAGCATGATGTCGTGTTTTTGCTGAAGTGAATGTGAAGCTTAGTGCATCACAGCTGAAAACAGAAGGGATGCTAAGGTCATCCACAATGCTGTTAGCATACTAGCCAGCACCGGAGACTTGTTGG |
| 100 | lncHBl_015 | TTCGACAATACCTGTCCTCTCAGAGAAACAGGGGCACAGACAGTAATGGAGTAAACCTCTTACAGGAGGCTCGTCTTCATACAGCACACCTAATCATGATGTATTTACAGTAAACTAGCACATATTACAGCCTACACTGCTGGAACACACGTCAACAGCTGGACCAACCGGTTTATACACTGAGAGAAAGAGAAAGTGTGAAAATGAGAAGACTCTGCGGAGTGTTGAATCTGTTCATCTGTCTGATTTTATCCAGAT |
| 101 | lncHBl_016 | GGAAAAACAAAACTAAAAATACAGAAAACTGCCAGAACAGTACTCGAGGGGGACACACAAATGAGTCATTCTCTGCAAGTTTATTTTATGTAAATGCTACTGCACGCTTTCGTCTCCTCAAGGAAAATGCAGAGTACTAAAAGCATTGTTTAATCAGGCATACTACATCACACACATCATTTCAATCAGGCCTAAAAATTCAACACTCAATTGGTAGCTGCTTTGCTGACATTCTCAGTGGCACTGTGGTATTCAAAAACACATTCATAATAATGCAGCAATATCTGAC |
| 102 | lncHBl_017 | TATTTATTGAGTATTGCACATTTGTACAATTTGTGCAACATAGAAATTCCACATGTACTGGCTCTTTTCCACATCACTTTGCTATACTCACACTAGTTGACACCATCTTGGAAATAGAGATATTGTGTTTGTTTGATTAATCTGGCTGAAGGAATCAGATATATGTTGCTCTAAATATTTTTTTTGGGGGGGAAATGAGTTTGCAATACTAAATACAGTGGAGAAAACAAACAGGAATAGGTGAAT |
| 103 | lncHBl_018 | ACGTGCCACAGCAGCTGCTGGGGTCTTTAGGTGGGATATATATCTGGTTCTGTTGGATAGATAGCACAGTTAAGTTAGTAGGTGTTTTGTGCTTGACTTCTGAGTGTCTTTGTTCATGTGTTATACTCACAGGCTGGCAGCGTGCAGTGCAGTTAGTGCCAGTGGATCTGAGGACGTAGAAACCAGTGGCTGAGTCTAGTGTGTGTGTGCACTGTGTGGCGTAACACACACCCTGATCCCGG |
| 104 | lncHBl_019 | CAGCCAAGAACTGAGTGACTTCCTGAAAAACATTTTCTTTCTGGGTCTCCACATCCTCGCTTACCCACTAAAAGAACATCTGCATCTGGCCTGCTGACCCTTGATACAGCTTTTTAATGTTATTTGCAGGATAACATTGATCAGATTCTGATCAGCTTAACCCTTACACACAATAATTATCAAATATATAGATGTATTAAGCACGCAGTTATTGTTGGCAAGATGGTTCTAGGTCAAGCCTTACACACCCCCAGAGCGAAGGAGAGCAGCGACAATTCAG |
| 105 | lncHBl_020 | CAATTATTGTATTTACTTTGTCTTATGGTTTGTTTGTTTTTGTTTATTTAAATTTTTCTGGGAGGTGGTTGTCAGTATAATGATATACTGGTCAGCACCTATAACTAATGCTTAATCCTAAATGCCTTCATTTTGTGTGCAGTAGATAGTTAATACAACATGTTAACTGCATACAGGATAATCTGCATTCAGCAATTAATTTTACAATAATGATATGATTCCAATCCACAGCAATGCATGTTTGATACAGAAGAGTCAGTGAGG |
| 106 | lncLBl_001 | ATGAAGACTGATCTTAAGGATTTTGACTTTATTTGAGCATCTCTACAACCATTGACTATCACGACTGCATTTAAACCTGATCTTGTGTAGATATAATTGGTACAAAATGTTTATTGCAGTTGACTGACCACATGAGTAACAGATTTATTCACTGTGGCTGTACAAATTGCAATCTGTGACTGCATTAGACCATGGTTTAGTTTCTGTAAGTGCTAACTCTATTGTATGACGTAGATTGATTCAATTTGTGTTCATCTTTCATAATTCCTG |
| 107 | lncLBl_002 | AATAACTCTTTTGAGCCAGTCAGAATGCAGCACCCCATATGGATATCTGATGTTCAGTATGGCCATGTATGAGCTTATATACAAAATTATATCAAAAGCTTGAACATACAATTACATTAAGAGCAAGTGCTGTGTACAGGTTGAGCTTTATATACTGACAGAATTGTTCTTTAACTACAGTACCTGTCACAAGCACACATAGATCAGAAAAACAGGCTGAATAACCTGGAGCCACAACAGCAGTTCATAAACTGCCCATCCTTCAACTCCAAATCTGATCGCT |
| 108 | lncLBl_003 | AAAAGTACCAATAGAGACCCACAGGAACATTACGGTTTCAATTGAAACCAATACGATTATGACCATTGCAAAGTCTATTCTGACGCAAGGCAGTGTTATGATATGTTATGAAACCTTATAACACCTCTGAGGGCGTTTTGGCACACCGTTTGCCTTTACCTCCTTTGCACACAGTCAGCATACAGTTCCTTTGAGGGGCCGTTTCGAGAGTCACGTCCAAAATGAAACATTATTGCAGCAGCAGCAGTTTATAACATGTGCCTTTCTTAACTCTTGTGTATCCTCATGGCTATTTTTGGCCTTTTAGGTTTCGTGATCATTTTGACTGAGTTCATGCACTGAAAAGAAATGATTCATTGAATTTACACTTTTTTTTAAAAGGTAAGTGATTTAAAAC |
| 109 | lncMBl_001 | GTCTGATGTTGTGATCTACTTGGGACGTCAGACCAGAAATGGCTCAAACCCACATGAGATACCCAGGACAGTGATTCAAGTCATCAATCATCCTAAAGATAACAGTTTCACCAACAGTATAGCACTTGTCCAGCTCTCTTCCTCCGTGACTTTCACTGATTACATTAGGCCAGTGTGTCTGGCTGCTGCTGGTAGTGTGTTTGTTGACGGGACAGAGAGTTGGGTCACTGGCTGG |
| 110 | lncMBl_002 | GCCTTGTCAACATTCTGTTTAGTTTAGAAATATTACTTTGCTGCACACGCTGCATCGATACGTTATGTGATTTACTGTATGTACAACTTGTGGTGTGATGGTTAAAGTTATGGTTTGGATGTCAGAAGTTAGAAACCCTCAAGACCTGATTCCTAATAGGGATCGCTGCTGCAAACATTAACATTACCTATTTTACTAGCTCAATTTGGGTTTTCACGGTAG |
| 111 | lncMBl_003 | TTTGTGTTTACCTTAATAGTAATCTCAGCATAAACTGACTTTACTGTCAAAACAGGGAGAACTCTGAGCTTAGCTTAGCTTAGCATAGCTGTTGAGTTTTCTGAGTGTGAAGCATTGTTTTGGTAAGGAGTAAACTTTTTAGCGAATGTACATTGCTGTCATTTGTGTTTATTTTAATAGTAATCTCAAAATATGCTGATATGACTGTCAAAACATTGCTAATTTGACCCCGTATGAAAGCAAAATGTTGCTAATTTGACCCAGTATGGGAGCAGGTATGGCACAAGATAACCTAAAG |
| 112 | lncMBl_004 | AGCAGAATGATGTTCATATGACTTCACATGAGAGTGAGGTCAATCCACAAAGAATAGTTCAGGTTTCATTGTGACGAGCCAACGTTTCGTTCTTCTTTATACAATGACATTATGATCAACCACAAAGTATGAACAGAAACTAAACAGCACAATCAGCATGAGAATGTTATGATGAGCAGATAGAGTTGAAGTCAGAATTAT |
| 113 | lncHBr_001 | ATTTCACTAAAGTTTTAAATGGTATCATCGTCAATGTAGTTCAAAGTAAGGCTCAAGATAGAGTCCATCGTCCTACTTGACCGGAGATCAGATGTGGCTGTAAAGTGAAGAAGTAAAAATCACCCAGAGCCTTTAACAGAGCGGGGTCACGTCACTCGTAGGGAAAGTAATTGAAAAAAAATCGTCCTGGAAAAATTAGATGGATTATAGGTTCTGAATGTCGATTAATTGCCCAGCCCTAGCTGGTAGCCATTGACTTGATTATATGAACCACCAAGGACCCCAGTTT |
| 114 | lncHBr_002 | GGTGTGCTACGGTACGGTACGAGTCGGTACTTTCAATAGGTACCAAAAAGCAAGTTACCATAGGTACTAAACCGTTACTGTTACTCCTGCTGTTCTATTGACGCTCCCCTGCTGGATTACCTCTTTTATTCATTCATTCATTTTCCTTCGACTTAGTGTTTATTTCAGAGGTCACCACTGCGGAATGAACTGCCAACTATTTCAGCATATGTTTTATGCTGCAGATGCCTTTCCAACTGAAT |
| 115 | lncHBr_003 | GATTACATTTGACTAGATATTTTGCATGATATTAGTATTCAGCTTAAAGTCCAATGTAAAGGCTTAACTAGATTAATTAGGCAAGTCATTGGACAACAGTAGCCTCGGACAGTGCTTAATTTGTAAACTGCGAGGTCCTGGAACAGATCAGGGTAACCGATCCGGCATGTCACCAGAGGAGGGAGAGGTGTCCCGGATGAAAGAGAAGAGCG |
| 116 | lncHBr_004 | TATGTGTCCAGGTGAAAAACATCCCAGAAGTAAGAAGCAGCCTTGACTAGGTTCTTTGAAGTGCACAACAGCTGTGAAAAGTCTCATGCGCTGATAAGGACAGACTCTGTGTCACAGTGTACAAACAGCACGGCTGCGTTTACACACAAGATAAAGATAGACTGTGTACTTTCCTAAGAAAGCTACATAAGAATGTTACATGGGCACAACTTGGGACATT |
| 117 | lncHBr_005 | GTTTGGTGCTTTATTAGCTGATGGTGAATATTGTGTTTACCACTAGTGTCAGATATAGACACGGCGTTTGGTTCTGCAGCAGTCAGTGGATATCATCTGGAGTATTATACTGACGTTGTGTGTGTTTGCCATGATTATAATGATGCCTCCGTGAATGCTGGAGCTGATGGACGAGGCCAGTTGTGGCTTGAGATGCAATAAACAGAAATGCA |
| 118 | lncHBr_006 | TTTACATTGTCATTACTAGACACACCCCCTTTTATATTGCTATTATCATTACTAGACAAGCCCCTTTTTAACTAGACACGCCTGTTTTTATATCTCCAACATAATTTCTAGACACGCTGTTTTTTGATATCTCCATCTTGACACGCCCCTTGCTGCTGATTGGCTGCAAGTGTGTTTTGGGATTCTGTGGTTGTTCAAATATTGTTCAGTACGCCTTCAACACAGGATCCATCAGCATCTTAAATATGTTATTCCTTCACTTTTTATTTATTGATTCATTCGTGTGTGTGTGTGTGTGTGTGTGTGTGTGTGTGTGTGTGTGTGTGTGAAATGACATATTCTGGGCTCCACATTTAAAAGTGCAATTATAATATGGTCAAACGAGCCTTGCTGTGATTATTCTGTAATTATTTACT |
| 119 | lncHBr_007 | CACATACCGAGCTGTTTTGGCACTGCACGCTGGAGCTGTTGAATCTGAGCGCCGTGACTCTGTGACTGACTCCTTGAATGTGTAACACACACTCGTATCCGCGCTGCCCCGACTGAGGTTGTGGAAGGTTCCGAGCTTTCAGGGTGATGGGCTTCACCTCTCCTGCTGGGATCAGGATCTCCTCAGAGCGCACCAGTTGAGGGCA |
| 120 | lncHBr_008 | CTAGTCTGAAATAAGAACGAGAAACAGATCAAAGGCATCTATAGGACACACCAAGCTGACGGTTGGCCATTGGGCAGGTTTTGGGTTATCATTTTTTTGAGTGTACTGTACGTCTCGTCAGGCTTACGTCAGGGAAAGTTTGTCTGATCCAGCATGTAGAATCAGCAATCTGATTGGCTGTTCAGCAACAAATAAGAGCGTGAAGACAAAAATTAAAGTGACAAAACTAAGTCATGAGTGGGTTCAGACAGATTCTGCACACATTTGTTGCTATTTCTGCTGAGAATATTGTAAAAAATCTGCAGAATTAATGCGGATTGATTTAGG |
| 121 | lncHBr_009 | CATTGGTCTATCTACAACTCCACATAAGCTGTAGTTTTGAAGTTCTTCCGGTCATTCAGGTGACATCTTCTGTCTGGAGCATCAGGCCAATCTCCTGCTGTCCAGAATCAGGTGGTTTGTCTTGGTCGGGGATCTCAGCTGAGACCTGTCCACGATGGGTGACCCTACCGATAGCTGTGAAGTACCAGTGGCGTAGCTCTCAGCATCACTGATGCACACAAGCCCTCACAGCACGTCAAGCTGCAAACCGTGTGAGGGACCCTAACCTACTCTAACCCAACAGCCAACGCTAACAATCACTAATGTGCCAAAACCACTCAGAGGAGGCCAACAATGCGCAAGACACCACCTAGAAAACCTTGCTAAACACCTGCCAACATCCAGCAAACCATCCAAAAGAACCCAAGAGCCAAATATCACCAGCACTGACAATCAGCAAAACATCCAGCAATGTGATGAAAACTTGAATGAAATGTC |
| 122 | lncHBr_010 | ATCAACTCAACACAGATAGACTGTTCAGTGCCCCACCCCTCATTATTTTCTCTGGAAGGTAAGGGAGAGAGAAGGGGAAATCTGACAGGGACAGATACAGAACGGTACAGTCACACAGCACAGTGATGAGAGAATGACAAGAATGACAGCTATTATTCACGCCACAGGCAATCCAATCAGTCACAACGGCTCATGCAACTAAGGATCAAGTAAAGTTTTCAATAAAG |
| 123 | lncHBr_011 | TTATCTTTCATCTGGAAGGTGACGGCCATCACTTTGATCAGCTGTTTGCCGCTCTACATCCTCAAATACCTGCGCAGACGCTTTTCTCCTCCCAGTTACTCCAAACTGACCTCTTAAACCCTTCACAAAAGAACAAAAAACCCTAAAACTACTGGAGCTCCAGACCTCCGTTCTCCATCATACTAAAGTAACAGGTCAGTTTATGTGGTAAGGCAGGCAGTTTTTTTTTTCGTCTGAAATGAGCAGGTGAACATTAGCTTGAAGGCTGGACGCTTTTCTGTTGGAGTCCAGACGCTGTTAGCTATGAATCATGTCATTTCTGAAGTGAGTTTAAGTTTGGGTGAATCACTGGCCCCAAGGAAGACTAAACACACTGGCTAACTTTAAGGGGTGGACAGGAGACAACAG |
| 124 | lncHBr_012 | GACCCAGTCGAGGCTCAAACCAGCTAACTTCTTGCTGTGAGGCTATTATGCTACCCACTGCACCACCATGCTGCACCATTCTAGTTCATATAAATTAAAATATAATAGAAGCTAATGCTAAACAGCTAAAATGCTACATAACAGTCATTGTACCCACAAAAACAAAATGACATTGCTTTACAATGAATAGCATGCCTGAAAAATGTCTTATCTCTCTAGACATTTTTATACAATGATGAAAAAGTTCC |
| 125 | lncHBr_013 | CACCTTCATCATTTCTTTGAGTTTAAATCAGTCAAACTGTAATACCCTTTACACAGTGGTAATACTAAAAGTACTCAAGTGAGCACATTGTGGGTGTCATTTTTAATTTCAAGATTGTTCAGTTTGAAAATGAATACAAAAGAACAGTTTAAAGAATAAGCTCTTCAGCATCTACCATAATTTGTTAACTATCACAATGCATTCCTCAATTTTTAGTAAAT |
| 126 | lncHBr_014 | CTGAGGACTAACTAACTCTTTTAGACACAAGAACCTGTACTTTACCAGCGTTTCCTTGCTCGGAGTGTTTGGACAACACAGCAGACCTGCGCTACATTTGGACCACTGTTTGGCCGACTTTTAAAGAGGCGAATCCTCCAGTCTGGAGAGTCTCCACTTTAAGGACTCAGGCAAGACGTGAAGCAGTGGCTTCTATTAAGTCCTGTCCATCTGGCACTGCAGTTCCTGCTATTACTTTTAATTAGATGCAATACTGGTGAATTTCTGTCGGCTTTTTCCTCATTATTGTGACATTTCTCTTTTTTTATTGTCAATGGCACTTTATTGTAACCAAGTAGGGTACAAATGGGGACTGAAATATTAGACTTGTCAACAACAGTTCAGGCAAAAGAGGGGAAAAACATTCACAAAAGGAATGCGGC |
| 127 | lncHBr_015 | TGAGTGTGCTGTGATTGGCTGATTGATGTGACTGAGTGTGCTGTGATTGGCTGATTGAGGCGGGTCTACAAGTTGTGATTGTATGATTGAGGAGGGTCTACAGGTTGTGATTGGATGATTGAGGCTTGTCTACAGGTGGTGATTGGTTGATTAACATGTCTGCATGTGTTGTGATTGGCCGATTGAGGTGTATGTATGTTGTGATTGTATGTATGATCTCATTGTAATATGATTGGCTGATTGAGGTGTGTCTGCATGTTATGTTTGGATGATTGAGGTGTGTCTGTGTGTTGTGATTGGCTGATTGATATGTCTGATTGTACTGTGATTGGCTGATTGAGGTGTCTATGTGTGTAGTGATTGGCTGATTGAGGTGTGTCTGTGTGTTGTGATTGGATGATTGACGTGTGTCTGTGTGTTGTGAATGCCTGATTGAGGTGTGTCTGTGTGTTGTGATTGGCTGATTGATATGTCTGATTGTACTGTGATTGGCTGATTGAGGTGTCTATGTGTGTAGTGATTGGCTGATTAAGGTGTCTATGTGTGTAGTGATTGCCTGATTTAGGTGTTTCTGTGTTGTGATTGGCTGATTGATATGTCTGATTGT |
| 128 | lncHBr_016 | AGAACAGTCCCGTCTGAAAGAAGGTTACAGACACCCAGAGAGGCAGCAGGAGGAAAAGCCAAACACAACCTGACAGAGAGCCATCTGTGAATGAGAAAGACAAACAACAGCTCGTGTTTGGAGGTTTCTACCGACAACTAGATCTTGGCGAACAGGAGTTGCTGCTTTGAAAAACAGAAGGTGTGAAGAAGAACAGGCCTAGAGAGAAGCAAACAATGGTTACTAGGAGAATGTGAATGCCTTGCAGACTGGGACACTCAAAAACTGCTTATTTCCAGCTTCCACATATTTCCTAATG |
| 129 | lncHBr_017 | GTATCTTTATTGTTATTGTTGAAAATTTGTTACTTGGTGTGAACAGGCCTGTATTTTGATTTTGTACTTTCTTACTTATTGCTTTTGGATTTTTTTTAAAATCACAATTAGTATCCTTGTAATTGATTGATTGATTTTTTACAGCATTATGTTTTCATACACACGTGTGTATGCCCATGCAGTATAGTGATGAACTTGTGATGTGCCTTAGGAAATACCTTATGGATAGCAGTGTCCAATCAATCGATGTGACATAATTATTCAAGTCTCACAAAGTTGACGCATATAAAGATGCCTTTTGGTCAGAACTCTTTCAACAAATAACTATTTAAACTCTCCCAAGTTTAAGTGGAAAGATGGCAGCTTTATGACATTTTTAGCAAAAACACCCAACATTAGTCATTTAATAGAATTTAGTTAAAAGTTTGCACTTGTGCTTGTATTGTGAACTAAAGTTTGTTCATATAATAGTCAATAAAACCTTTCATTTCTC |
| 130 | lncHBr_018 | ATTATTACTTGGCTAAACACAAAAGTTGTCAAAAGTATTTGGAGAAAAACAACTACAATCAAAGAACTCTCTGGCTGTCAAGCGCAGAAGAAGATCCAATGTTTTGAGACTCTCAGAACTGTGACATGCATCGCATTCGAGACCCAAAAGCTGTAGCAAACTTCAAACTCCTCTTCCACTGTGCAAAGAACTTTTCTAGACAGAGTATATTTTGATTTCGGTGATCAGTTCAAAGTGCTTTTTTTGGGTTACCTTCGCATTAGCTGCCAACTATTT |
| 131 | lncHBr_019 | CATTTGTAAAGTACGTTCAAAATAATTCAAAATGCATTACCTAGGTCTACTTATGTGATTTCAGATGCATAAAAGTTGACCAAGTTGGCTAAGCGAGATGTCCTTTACTACAGCCAAGATCTTGGAGATTTTATGCTTTCTTAATCAAAGTATTTACTGAAACAGCATGGGGCTATTAACATGCCAAAACAATCTGAATTTAAATTGTGTTAATTTGAATTATTAACTGTTGGAATTTAATAAAAC |
| 132 | lncHBr_020 | TTTCCTTCCAACTATTCCAGCATGTTTTACGCAGCAGGTGCCCTTCCAGCCGCAACCCAGTACTAGAAAACACCCATGCAAACTGCAGCTATGTTTCAGTTCTGATAAATTAATGATGAATTATTATTTCTTTATTCAAAAGCGAGCACTTCCAAAGTTTCAGTGAACCAAAAAACAGTGGACCATGGTACTGTAATACTTTTTAATTGTCACTTAAATTAAGAATCAGCAGATGTTTAACTACAGGTCAAGAAAATTTCAACCAGACATAAAAACTTAACAAAATCGAAAACAAAAAAACTCATTCCAACAATTAAACGAGGGCAGATTAAGACACAAGGTAACAGAATTGTTCCTTTAGAGCAGATGATATGCTCCTGTTTGCAACATCAACACAAAAATAGTTTCAGCACAAAGGCAAGAACAAAACTTCAGCATGAAATAAAGTACAAGTAGCCGGGATCAGTGTTTCAGGAGTCTAGTACTACAGTTTTATGCTCTTCTAACACATCAAAAGCATGAAGTCACAAAGGAATATACTGCACCCATTTGATCAACAGTAGTTTAATGAGCAATAGTGCATTCTGGAACAA |
| 133 | lncHBr_021 | GTATGTGGCAAGTCGCTGCATAATAATAATCAAACGCTGTATATTGTTCAGAATTGAATTTGAACCTGTATTTTAATCTTAACACTGCAAGCGTTTGCACGTAACACTTTCACTTTTTATCCCAGTGCTTCACTCGGACTGACATGATTTTTATAGTTTGCACTGTATAGTTTGTACACCACAATACTCGACAGGTGTTTT |
| 134 | lncHBr_022 | GTTTAGACCAAAGTGAAAGCTGAGTAAATGATGCCAGTTTTCATTTCTGGGTGAATTATTCCTTTAAATCAGCAAGAAATAGCTCACTCTCAGTTGAGTATCATTGACTGATGTTTCTTCCTCCAGTGCGAGCAGCCTTGTTAAAAGCAGCCGTGTTGAAGTAGGTCAGATTAGGCGATGTGTGTGTGTTTTTTTTTTCTCCCAGAGTTTCACTCCTTATTAACTGCAATAGAAAGCAAGCCTCCTCTGCACTGGAGGATCTGATGAGGGAATGTGGGCGTCCACCAGCTCATCTTGCTAGAAAACACGCAACTTTGTTTATTTTTACACCATCCGTCTAGCTGTGCTTCCCCAGATTTAATGTTTTAATGAAACCTATCTATCACTCTTATGCGC |
| 135 | lncLBr_001 | AAAGAACAAACACATAAGATGGAAATTCTACCTCAGGAATATATATTGCGCAGCTCTTTGCTTGTTTCTGCTAACATTTGTGTTGGAGTTGTACAGTCAGTGACAGTGTGAGCCCATTTTAGGAAATCAATTAAAAGATTCCGTTATGGACTTTTAATGGGCATACCGACAAGGATATTAAAGACTAGTTGCTAATGGAAGACTGAGGTTTACTTGCACAGGTCCTACTAGCTGCCCTCTGTTACATGAAAAAGCAACAAAATGAATTCAGATTGAGCACCTGGATGGACCAATTTGTGTAAAAATCTAGTGCTGTGGGGCACTGAATGTCTTCACTTACATTCTATATTTTAATGTGACCCTGGACGACAGAAACCAGTCATAAGAGTCATTTTTTTGTAAATCCAGGTTTATACATCATCTGAAAGCCAAATAATTGAACTTTATTCATTTCATTCTTGTATAGTTTTTAGGGTAGGACAGTATTTGGCTGAGATACAGCTATTTGGAATCTTT |
| 136 | lncLBr_002 | GGAACTACTACTGCAAAGGGAATTTTTCCTTCACAAAGCAGGATATCATCTCTCACTGCCTGACGCAAATGTACATCCATCCTAAGAACATGTGGAGTTCCTGTCTGTCATTCTAATGAGGGATCTTTAGTGTGCTGGAGGAACACGCTCTGTGCTCGGACACCATTTGCCGGCCGCTTTCTTTCTGTGTGGATTTACATTAAACGACCAAGTGTACAGCACAAAGCTGCAGTCTGGATGGATTTGGGTCGAACTAAAATAATAATGTAT |
| 137 | lncLBr_003 | AAAAATGAAAACTCTATTGTTTATTACTCACCCTTTTGTCGTTCCAATCCCCTGAGATCCTGAGACCCTGAGATTGATAAAATCCAAGAGTTCTCTCATCCTTTAGCAGTGGTCCTGAGATTTACAAAGTCTAGAAATGAAACCAAAAACATTCTCCAAACATTCCCTGTGGCTTGGTTCAACTGTAATCAAGCAATAAATACGTCAATATGACTCTGTTGACCTGTATCCTCTCTCCTGCGTCAGGTCAGGGTGTTTGTATACTACAAGCAGTTTGACGTGTGGACACTAGGGTCAGCAGTGCAACATGCAAGCGTCATATTAGTAAATGCGCACCCTGATCTGACACACTGGTGATGAATAATGTTGTTTTTTACAGTTTTTTGGCATGCAAAAGTGTTCAGGGATC |
| 138 | lncLBr_004 | TCTGAACAGAAAAGAAGATATTTTGAAGCACTTCCATATTGGTGAAAAACAATTCAAGACAAATTCAATGGGTCCCAGCAATCAACTTTTCTTTGCAAAATATCTTCTTTTGTGTTCAACGGAACAAACAAGTGAACAATGAGTAAGTAGTGGATTTTTTTTTTTTGGATAAACTATCCCTTTTAGTGCTTAATCTGTAGTTATTAGTGCCTTTAAAAAGATACTATAACTTATTACATACTTGTATCTAATTAATATGAACTAAAAGTTATTTCATTAGTAATCCAAACAGTTGGGTTTGTCCACATTTGAGCCAACGTCGGGCTACAACTCAGAATTTTTAGAGTGTAGTCAAGTAATAATAAGCTTTTGTGACTAGTACCAGTTATATATACATATGATGATAATGTGTGATAAAC |
| 139 | lncLBr_005 | GTGACTTCTTGAGAAGTCCAAATTCTGGACCCTTCAAGGAGCTCATATAGTCGGTATTTGGAAAATCAACCACAGAGGAATGTTATAATGACACTGTTTAAAAAAAGGCGAGCAAGCCGAGGGGCCTTTTGTTCTGAGACAGCCTGAAAAGAAAATTCATTTCCTGTCTGACAGATTACAGGATTACTAGGGGGAGAAAAAAAAACGCTACAAAGGGTACATGAGAAGTCAAAAGTGTCAGTTTTGCT |
| 140 | lncLBr_006 | TGAACTCTCGCCTCATCCAGGTGTTCACAGACTTTCCTTGTGTCAGTGAGCAAGTGATGCCGCATCTTCCCTTAACATGCATCAGTCAGAGAAGAGAGAACTAAGGTAGAAATAACTCACTGAATCTCAGTCTGAGCCTCTCTCCATGTCCCGGGTCTGTCGTGCAGGGATGTTTGTGATGAGTTTCTGCAGTCGCTCACAGTAAACGCGCATCTGTGCGCAGGATGAGGCACACGCACAGTAATCTGACCTTCTGA |
| 141 | lncMBr_001 | GTTTGCTCATGAGAGAATGATAAATACAATTATTTTAATATACCAGGTTTGAACTATTGTACAATATTAAAGGGTTCCACAGTAAAACAGTGGCATGATTTCCCTGTAGACTTGACATATACTCTATGTACTTAATGTAAAAACATTGGTGCTTGTACACCAGTACATCTTGAACATTAATTTCATTGCATAATGACTGAGAAACATGTTCTCAATTTTTCATAATAATCATTCTGGATGATCAAATGAAGAGTAAGAATATAGCTTTTTGAATCACCCGTGCTTTTGTTCGCTTGCCATTATTGTTTCACAAAGCTTAATATTGTTGTAGTCACTCATTCACAAGACTCACAGAATGTTTTTAGTATTTGAACTATTCATATGAGATTTAATGTAAAATACAGTCATAATCCAAGGAGCTGCTGTTTATTACAGATCTAACAAACCAAAGTGTTTTGTTTCTCTGCATTTAATTTACTTTTATAGCTCTTTTATGTACAGCATCTGTGTGTTTTCTGCTGTTTGCACATTGTTTAATTGTGATCTGCTGCTTTGACTTCAGTTTCCGCTAGATGGAGATAAAAGAAACTGGTGTGGTGTCATTTAACAATTCTCATGGTGTATTTTAGATGCTGAATGGACTTATTAAACCAC |
| 142 | lncMBr_002 | CAGAAACTGACCTGAAACTGAGACCCAAGCCTTGCAGGAGGTTTATGCATGAGCACATTCTTAATGAAATGTATTTTAACACACTTTAACAAGCAGCAATGTGAGAAGCCGAGTTTTGTTTACGTAGTATTTTAGCCGGAGTGTTTTTCGGGTTTCTGATCTTGACTTCTTTTTGTGTGTGTGCCCGCGTGCTATTTCAAATACATAAATGCATTTTTTTGTTTGTGCATTTTTTGAACATTTCAGAAN |
| 143 | lncMBr_003 | ATTTATTGACTCTGGGATTTAATCTGTGATAAGCTGCTCTATGTGCAATCTTTGCAGCTGTTTTAAAGTAGGAACAAAATCTAATTATAGATTATCTTTTTTACAGTAAACAGTTCTGAGGATGGGGTGTGCTTAAGCAGTACAGACTTCCAGAGGGCATAAAAAGCATGTGCATTTTTTTATATTCGTCATCAGTTTAAATCATGTCACTGACTTTCTCAGCTCTAGTGAAACAACAACAACTAAAGCCATTCTTTGCTCCTTAGAGCTGAAAAAGACACTCAAATCAGTCAAATCAGATGTTTAACCCAGTAAATTAGTTAACCCTAATGGCATATGTGGGAACATCAAACATTTACATATAAAATGTTAGCAGCAAACAATACCAGGAAATATATTAATATATGTATATATTAGTAAGGCAGTGTTAGTAGTTTAAATGGACAAGTTAATATACACTGTATGGTGCGTTCTGTTCATGCACCAAACACAATGAAAATTTTACCTTATTAAATATATTTAATATACAGTTGAAGTCATAATTATTAGCGCCCCTATTTATTTATTTTCCACCAATGTCTGTTTAATGGAGAGAAGATTTTTTTTCAACACATTTCTAACCATGATAGTTTTAATAACTTATTTCTAATAACTGATTTATTTTATCTTTGCCATAATGACAGTACATAATATTTTACTTGACATTTTAAGACACTTCTATACAGCTTAAAGTGACATTTAAAGGCGTAACTAGGGCAGTTAGGTTACCTAGGCAGGTTAGGG |
| 144 | lncMBr_004 | TCTTGTGATAACATAAGGATTACAGTGTGAATGCAGTGTTGACCACTAGAAATATTATCTATATGGAGATGTTTTAACGCAGACGTTGTTTTAATGTTGAGAAACCCTCGTCTGGTTGTGTACGTATGTGCAAGAGACTGTTGGTTTTGAATGTTCTTGTGCTGTTTTTGTGATTATTAAAGTGAATATCTTCTATGACGGTCT |
| 145 | lncMBr_005 | GAGGAAAGCAAACTTTTATTTTGTAAGTAAAAACCACAAAAGGACACAAAAACCTAAACAATAACAATGAAAACGTCGGAATAAAGCTACATAAAATAACCACAAATATGAACTAAAATTTGAACACTTCAGAATAAATATCAGATAATTAAGCTCTGACTTCTGTGCCACGTTTTTCTTGCAAAACTGTCTTCAGCAGTGCATAGATTTTAATGGCTTTAATACTTTAATTTTAACAGCTTTGTTAAGAGAGATTTACAGCATAAGCCTCCTGTAATGGAAATAAGTAGCATTAAGAAAGTGAAGCTTTAAGCTTAACACAAAACAACCAAAGATGAACAAAGAGAATGGAATAATTAATTCCTCCAAAAATGTAAAATGTTAAATTAC |
| 146 | lncMBr_006 | CTACGGTAACGCAAGACGCAGACGGCGCTCTGCATAGGAGCTGTCCAGCGTCTCGGTGCTGAAACTTGCCCTGGCGGCTCTCAGCGAACAGCAAGAAGGTTTGGTATCAGTTCAAAATAAGGCTTAAACCCCCAGCAGCGAAGACGTTGCTGATTCCGATGCGTCAGTACATGGACAAAGGTCTGCAGATAAACAGAGGAAGAAGGGACGTGGCGAGAACGAAAGGGGGACAGAGAGACTGAGTAAGACTGTGGAGAAGT |
| 147 | lncMBr_007 | CATTTTCAGAAATAGTTTTTTATGATGAAAATGTTTCATCAACAATCAAAATTATTGTTAAAATCAATGTATTTTACATTGATACATACATTAGATAGTAATTGAGCTATGTTTTATTTTTCTTTTAGCTAAATATGGACAACCAGCAGTGGCATGTATTAAGGTTTCACCTAAAAATTATTCTGTAACCAGTGCCAAAACACTTGCAGTACATCTTTCAGGCTTAAAGTCCCCATGAAATACATTTTCCTTTATAAAGTAGAAATATCTCTATAAAACAGGATATTGAAAGTTAGCTAATAGCTAACTAGCTAGCATTTCATATTTATGTGATTGCAAAATATGATTGTTAAAAGCATCGTCTTCATA |
| 148 | lncMBr_009 | GAAATGTTTTAAAATGAATAAAAAAATGACAGGAGGGTGAATAATTTAGTCTTTATAATAGGCTAGTTAAAATAAATCCTTATAATTAAAGGTGGTAAACAAACACACCCATAACTTAAAGCCCCGCCCACTTTAAACCACCTCCACTATAGTTATGACACGCCCCTTAATGCTGATTGGCTGCAGGTGTTTTGAATCTTTTTAAAGCGTTTTCAGAAATTGTTTATTGCATTTTTGTGTATTACTGTGTTGAAGCAGTGTAATAATAAACAATCGGTTTGGAAAAAATATTCATATAATGCATAAATGTTTTATTTATAGCACAATTTTAAAAATTGAATATGTGATTTTCTAAAAATCAATCAAGATGAATATTAAAGAGATGTACTTGCCTCAAAATCTCTAAATATTACATAAAAATGCACAAAAATG |
| 149 | lncMBr_010 | CTCCAGTGCTCCTCATCCGCCAGTCGGTGCAGCAGAAGCGGGTCCGTCAGCATGGAGCTCCGGGCTGCATAACGCACACATCCAGGACACACCGCGCTCTCCTACGCACAGATCTGCGGGATTGATTGACTGACTCCCTGCCTGATTGATTGATTAGCAGCGGATTTCGCGCTCGTGTCGCTCCACCATGGGCTGCGTCACTGGAGATATTCGCCGACTTTCCGCGCTCCTCATCGGGCTGCAGGTCCTCTACACACACGGTCAGTGCTGCTCAC |
| 150 | lncMBr_011 | TGCCAGTCCCCGAGCAACAGGTCTCGGAATCGGTACCGCGGTGGCAGCGGCAGCACCTCCTTCTCCAGCTCCACCATAGTGAGCAGCACCCGAGAGCAACGCGGACCAGAAACTAAAACACTCGCACCGTTTATTCCTCCGCCGTGATCGTTTCAGGTGTTATAAAGTGTGCGCATCACTCAGTAACGGAGACGTGGATGGTCCACTAAAACAGCGGCGGCGGAGCAACTGGTGCCTCCCTGAGCTCTCCAAGCATTGACACGAGACACGCAGACATCTCCGCTGCTGACACCAGCTGTTTCACATCAGCCGTAACAGTTGCCAGTTATTGCTGCAGTGCATGATTTCACCCACTGTCCTGC |
| 151 | lncMBr_012 | CAATAAAATCAACATCTCGCTCATTAATCATCATCATATAGTGATTTCTGAATGATCATGTGACTCTGAAGACTGCAGTGGTGATGCAGGAAATTCAGATGTATATCACATAAATAAATTACAACTTACTATGTATTTACCTAGACAGCAGCTCCTTTAAATTGTAATAATATATCAGATTTTTGCAGATTTTCTGGGATTTTTAACACTTAATTCTCAGCCTCTGTGCGCAGGAGAGTCCTCTGCTGAAACACTGCGGTGTGTATTAATGCAGGAGTGCTGGTGTGAAATGCCTGTTGTTTCTCTATAATGAATGTCTCAGTGGAAACACACCCTCTGTCCCT |
| 152 | lncMBr_013 | GCAGCTGAAGAGGCAGGAGCGCTAGAATCACAGAAGACAGAATGAAGGCTGGACAGAGAAAGAGAGCGAGAGACTGAAAACACACGTGACGGACACACACACGCACTCACACACACGTCTGTTTCCACGGTGACAACATGGGGGCGGTTGTTGGCACACTGACCTTGCAGACGAGATCGAGACGACCGTCCAGAGGTAAGA |
| 153 | lncMBr_014 | CAACTAATCACTTTTAAACCCAGCTAATTTCTTCAAAGGCAGCATGATTACTCACTTCTTCAAAATAGTCAACTTATCACTTTAAAAACGACAGGTTTCAACACTTTTCCAAAGTAGAAGTCAACTAATTGCATTAAAAGTCAATTGATCACTTTGAAAGCAACTGCTTAACTCATCGTTTTAAGTAAAGTCAACAAATAGCTTTAAAAGCATATAGCCAACTAAAAGTCACCTAATCGTTCTAAGGGCAATCGCTTTAAAGGCAACTGGTTTATTCATTTTCTAAATTAAAGACAACAAATCACTCAAAAAGTCAACTAATCGCTCCTAGATTCAACTAACTGCTTTAAACAGAATGGGTTTAGTCACCTTTGTAAGTAAGCAAATCGCTTTAAAGACAACTAATCTTTCTTTAAAAGCA |
| 154 | lncMBr_015 | CAGTACGAACCTCAACCAATGTTTACATTTTGATCATACTACTAGTCAAAAGCCATTCAGTGCTCCGACATTGTTATACAAAGCAGGTTGTGCTTGAGATGATCTGGGTTTTCCCCTTCTAATCTCGATCCCATTCCTTATGCTTCTAGAAAGACAGTGCGGGGTGTAACTACACTGAAGGGATATTACTTTTCATTTTTAGGTAATTATTACTTCTACTTAAATACTGCACGTAAATTCAACAGTTCTGAGTAACAGTTCTGTAAGTTCCATCAATTCTGAGCAGAGTAACTGCATTTTGTTAGACAAATATTTTGCATAAAT |
| 155 | lncMBr_016 | TAGATAGATAGATAGATAAATAGATAGATAGATAGATAGATGTTTTTATACATAATTTTTACCATTTAGTCATCAAACATTGGTCATTTTTGAATGAAATATTGTTTATGAACCAAACGGTTGAATAATTAATTGACTATGAATGGATGGATAGATATTTTTATGCATTTTTCATTTTTAAAATAATTATAAATATATCCCTTGGTATGATGAGCATGTTTTCATTTTAAAGAACCTGTTGTATACAGTTAAAGGTTCTTGCTGACACCATTGATCCTATTGTAGAACTGTTATTTATAAGATTGTATCTGATTTGATCTTATCAATTAATCTGTGATTGTGTCTAATTTGTCGATGAATCATTCATTCTGTTCATTTGTTCAAAATCAGACTCATTGAGGGATGAAAAAGTGACTTCTCATGAGTGAGTCATTGTATTATTCACTCAACCATTTGGTTCAAACGCAGTATTTAAATCAGAAACGAACAACTTTTGATGACTAAATGGTAAAAATAATGTATAAACTCTATCTATCTATCTATCTATCTATCTATCTATCTA |
| 156 | lncMBr_017 | GCAATCCTGTAAACCCCTGGTATAGCATAATTTTATTTTCAGTATTTATTTATTTATTTAGATGCCTGGGCTCAAACTGAAGAGAGAAAATTAACTGGAGAAGATTTACACCTCAAGGAAGCCTCCACCGTTATCTGTTACAGACCAATGGTAGTTTGACGGAGTTTACCAGCCAGCGTGAACTTTTCCGAAAGAGTTGTTTGATCAAGCTCTTTTGAAATTCCAAAACTGCAAACTAAAGGGGTCGTACCATGGATGAAAGATTTTTGGTGCTTTTTGCCTTTATTAGGATAAAACAGAGAGGGGGGGATACGATTGGAAAAAGTCCATGAGCTGGGACTCGAACTCAGGTCACCTGAAGCACAATTGCACAATATGTCGCACTGCCCTAGGCTATCGGTACTGACATAAGTTGTTTTTTAGGAGTTTACGGACACCAGCGGCACCATCCAACACCTAGCTATGATTCAGGACTCTTAAAGTCCTGCCATGCCACAAAGCGACACCTGCAGTTCAATATTAAATAACATTATGACTGCGTACAAGTTGTGTGCATCTGTTAGGCCGGGGAATCTTTAGAGACTCATTAAAGTTACTATTTTCCACCTGCCATCCTAAAGGGGAGCTGTTATGCACGTAAAAATCAC |
| 157 | lncMBr_018 | GCGCAGACGGACGCGCGCAGATGCTCCGAGTGCGCGTTTGCAGAAGAGCTGTGGATGCGGAGGAAACTTGAGTGTGTGTGTGTGTGGCGCTGAAGATACCACACACAAACACACTTCGACTGCTGATAATGCCGCTGATTAAGAGCGGTGACGGATGAGAAGCAGTGCGCGCGCTCGTCACACACCGCAGACTCTGGCAGTGAATTGCAGAACTGTGTGTGTCAAATTACTGCTGCAGGACTGAAAATGTGG |
| 158 | lncMBr_019 | GTGAATTTCACTCATTTTTAATTCTTTATCCTCTTTGCTACCTCAATTCAAATTCAGGAATTGAATTGGAATTTGCAAGCCATCCTCAGTTCAATTCTGAATGGTGCACAACCCTGGTAAACTTAGTATCTTCTGTCTGCTTGGCGGTGTGGATCTCTGGAGGATTAAAACTGGAACATAGGCCACTTTTCTTTTAGCCGACTAGCAGGGTCTTTAATGAAATCCCCAGACAAGACCTTTTAGTTGGATTCTTTAAAGTTGTGAGTTGCCTGTGTAATATCAGACTGATTCAACATGTCTTTTTTTAATTTCCCTCCTTTCATGTTGTGTTTGACGTAAAGCTCTCTTTTGTTTCTAAATTAGAGATAATATCCTCTGTGTTTTGAGCAGAGCTGCTTAGAGATTTTAATGCTTACAATAAATTCTTTTGTACA |
| 159 | lncMBr_020 | ATTATGATGTTCACAGGCATGTGATTATTAAATAAATTATTCTAAGAAAATGTGTTATTTTCTTTTAGTTTGTTTTGGCATCCATTCAACTTGTAAATCAGCTGATGGTTTATGAACATTAATACCGACACAAACCCACACACAGACACACTCGCCCCGTCCCCTGCTGTTTAAATACTGCTGATCTTCTACAAAACCGGGAGTTCGGTAATCACTGCTCGTTTCGATTCGATTCACAGCAGGAGGGGGTGCATGGTTCGGATGGGAGGAGGGGATATGGGGTTTGACTGTATTACATCGCTCTGTGGGAACTATCTAATTCAGACCTCCACATAACTCTGCCTAAAAATCAATAGACTACAGTCCGGCAAGAGACACATGGAGTTAGAGAGACACCGAAAACGAGGAATAGAGAGAGAGTTGGGCGGACTGTAAAAG |
| 160 | lncMBr_021 | TAAATTCTCATTCCACTACAATAACTGCAACTGCTATATTCACTGTTTGATTAGATTACATGGGCTGGATTCACTGGTTAGATGAGCTAAAATGCAGCAAACACTGAGGACATCTAGTGGTTACAAGGGGTAATGCAGAAAGAAAAGCATAAAAACTAAACAAATCATTTTTATTAATCAGTGCAGACACAAATATAGTTATTGGTATATATAAACAACGGACTGAGGATATTGGCTCGTTACAACGGGGTATTTTAAAAATAGGTATGAAGTGCTAGTTTTGCGTTATTGAGTCTTTTCAATTCCATTATCATGACTGTGATTGTTATATTCACTGTTTGATTCGATTACACGAGCTGGATTCACTGGTCAGAGAAGCTAAAAATGGTGGACATCTGCTGGTTACAA |
| 161 | lncMBr_022 | TCAGCTTTGAAGAACGAAATGATCCTGGATCATGTCAAATCGTCAATATCCAAATCCAGCTAACTGAGTAATCCACGTACGAAGAACGGACCCCTGGTTCACAAAAGATCCTTTGTGTTGTCAATACACTCTGATATTAATAGAAAAATATATAGTTCAAATCTATAGCTGTAGTTTCAATTATAGTTGAAAAAGCTGCATTGTTTTGTGAGAAAATAAAAGAGTTACTCCTGCGAAATGCTGCATGTGTTCCTCAAAACCCCGAATTTATTTCATAGCTAACGTTTGAAAGAAATGAACACGATTAGGGAG |
| 162 | lncMBr_023 | GTGTAAACATATTTTTAAGCGTTCATGAAATTGGATTTTATTTGAATTTGATGACATCCTATTTAATAAACAGTGTGAATCTGATATATTCCAAAGATGTGCTGGTCTTTAACTCCTATTGAAGTCGTCCTCCATTCAGATATTAGTAATAGAAGATGCGTTTTGTTGGTTTTGATTTTCCTGAATTGATTTACGCTGCTACATTAGAGTCGTTTCACCCGGCCGTCCTGCTTAGCAGCTGGTGAAATGAATGTTTACTTTTTAGTTTTATTTGTAAATAATAATAAATAATGAGTATTGTATCATATTAAGTTCTTTTATATTGCACAGAAATGTTTAGTACATTGCGAAGTGGTCTCGTTGATCTCTGTGGGTTATGGTCTTATTTTTTTGTACAGCTTATGTTTTCTGATGACCTGCAGG |
| 163 | lncMBr_024 | TCTCTCCAGAGGAGTTTGAGTGTTTTGGATCGTCTTCTGCTCACACACTCCATATGGCTGCAGCTGTCAATCAACCCTGACTCCGCCCACCAAATCCTGCAGCGAGAGATAGTCGGGACTTTCCTGGTGTGTAAGTGCAGCGCCTCCCACAGGAAGGTTTTGTGTGTGCGGGTGCAGGAGGGAGGAGTGTCGCAATATCCTGTTCGTGAGGAAGATTC |
| 164 | lncMBr_025 | CTAATTTGTAACTTAAAAAGTCTTTTAGTTTGGAGTCTGTGCTTTTGGCTGTGGTGTAGAAACTTAAAAAGAGATAGTTCACCCAAAAATAAAAAACTACAATTAAATTACAATAATCAATAAACAATGACTTTTTTTAGCTCAACCTGTGGTCCTTGAGGATTCATAAAATGCAATTCCTCCATCCCCATTGTAAACTCTATCAATTCTATTTTGCCTTCTGAGTGCTGGTAGTTGTTAACTTACATTTTATGAATCACCAAAAACACAGTCTCAGTTGAAAACTTCACTGCTGCACAAAAGAAAAAAATCACCAACATCTAGATTGCACAAAAG |
| 165 | lncMBr_026 | TTTTAATTCTTTAGAAAAGTGTTGATACATACATATGTGAAGAAACTGAACGATTGAATGAAAACGAAGACTAAAAAATGGATTTTTGTTGTTTAATTCAAAACAGCATGGGCGGCTCCTAACATATCACTTTCATTAATGAAAACCAATGATTTGAGATGAAGCTTTCTCCAATGGGATGTCACTGCTGAAATGACATTGTGACTTTTTTTTTCTCCCAAGCTGTACGTCACAAAGCTAAATATACTAATAAAACCGTG |
| 166 | lncMBr_027 | CTGCCGAAGTTTATTTAAATTGATCTTCAGTTTTCCCAAATTGACTTTGATCAATCTAGTGTGGCGGAAGACTTGCATATATTATTGTTTTTTTCAGTTATCATAGCTTTCAAGTGTTACACAGTCCATTACTAGGTCAAACGTATGATTTCATCAACACTCACCCAGTGTTAAAATCAGTGTGAAAATCCTTGATTTAGTAAGTCCAGTTTATTGCTTCGATTTATACGGAATTACACTTATATTGAGCTTCAATTAACGGCACTCCCAGTTCTGAACTTAAAAGTCT |
| 167 | lncMBr_028 | CTCTGGTGAAGAGACAGCCGTTTACACGTAGAGAGCTGAGTTAAGCAGGTGCTGCTGTTTCATTCACACACTTCCAAAACCACTTGTGCTCCCGTCCAGCCGGAATGGGAGTAATATAGCACCTTTTACTTCCATCACTGCTACTACCATTAGAGGTCTCTGACCGATTTACTGCAGAGGGATGGACAGAAGGAGGTTTGTTGGAAGAGGAGCAGTTAAGAGGAGCAAATATATAGTGCCATGTTTCCTGTTTGTAGAGGTG |
| 168 | lncMBr_029 | GACTCACTCTGAGCTAATCATTTGAACTAGTGATTCATGAACTGGAGACTCACTCTGAGCTAATCATTTGAACTAGTGATTCATGAACTGGAGACTCACTCTGAGCTAATCATTTGAACTAGTGATTCATGAACTGGAGACTCACTCTGAGCTAATCATTTGAACTAGTGATTCATGAACCAGAGACTTAAGCTGCGTCCCAAATCACATACTTATGCACTATTCTAC |
| 169 | lncMBr_030 | CAGTTTGGTTTAGGGGTGGGGTTTGGTGCCACACCTCCTTTTAAAAATTTAACATTTTCATATGACTGAACTCATATAAATTTGTACAAAATTAGCCAAGATACTGACCATACGTAAAATAGTTACGTTCCATCGTGAAATCAGGCTGGGTTTTACTACAAATTACAACTACTTTTGTAGTTATGACAAAACATCCATGGTTTGCTTCATTGATTACAACATTATCTGCCGTAAATATGGATTATAATACAATAGTAATAGATACGTCAAACTGAAAACCGGGTTAATACACTTTTACGATAATAAAACTTGTATAACCATACTACACGATCATGGCATAGCAAAGTAATGTTTTGCTAAAGATTTTTAATTTGTTTAAATTGTTTGCTCATGCATAAAATGGAGATAGACAGGAAATCACCAGATATTAATTTATAAATAGTGTACTTGAGATATATTTTGAAGCTATACGTTTTGTGCAGAAATTTCTGCACCCTTATGATAATTAACAATTGCTTAATAATAAAAGTGTGCCCTCAGAAAAATTTAAGTCTTAACTGTTTGATTTAGTGGCTAATTCATACGAATTTGTACGGTTTTATTCGTACAATTTAGTACGATTTGCTCATTCCCCATTGATGGTTGGGTTCAGGGGCACGTTTTGG |
| 170 | lncMBr_032 | CCAGCCAGCAATGTTTATTTCACTGTTGTGCATCATGGCTAAAATATACAAATCAGTCGCTTCACAAGACATCTGTTAAACATTCACATCATTCACAGGTTAATTCAAGTGCACTTGAGGAAGACAAGCTTAAAGTGGACACAAAGACTAGTCCGTTTCTAGAATGACAAGTTAAACGAAACCACAGAAAGTGTCAGTAACTAGA |
| 171 | lncMBr_033 | GCAGGTTTCTTTTTCTAAATCAGTGTTTTTTTTTTTTAATCAGTGTGCAAATACATAGAATATTCATGAAGCTGTACATTCCAAAAATTCTGCAAGAACTTATTCCACTGCTGCAGCTTATTTAAACCTTGAGTATTTTCCACATCAAATACTCATATCTTCATGTTTCTTTTTTTTTACTTCTTCTTCACAGTGTGTTGATTGTAGTCTCGTTCTGGTGAATAGTTGTCCTTTTAAACTAAAGCAAAGATTTATACCATGTCCTTACCAGCACATCTCAAGCAATAACGTAAATTACTGCACAACGGGACTCAATATGGTAATAGCAAACTGAGATGAGAGCGCTGGATGGATGAAATTTGTATTTTATTTTATTTTTTTGTAAGCCAACCCTTAAAAGGATAGTTCACCCAAAAGTGCAAATTACCACATTATTTACTTTCCCTCTTGTGGTTTTATAAAATAAAT |
| 172 | lncMBr_034 | TATCTGTCATATTGCTTTTATTGTTACATTAGAATACAGACAAAAACAATGCTGCTGGTGAAACATTTGATTAGCTTTTGACAAATAACTAAACAAACTGGACTCTACTTCTGTATCAACATGTGTAAATCCAAATATAATCCTGCTTCTATTTCAGAAATCCGTACTTGTTACACCATGCTCCAGTAGACCCTTTATAAATTATTTTTAAAAGGTGAATCTGACTGGAAATTGTATCATTCAGAAATGTAATGTCTAATCAACAGTAAGAACTAATAAGCTCACCTGCTACATCTAATATGCTAATATTTATTAGTACTGTTATATTAGTATAATATTTA |
| 173 | lncMBr_035 | AAGCCGGAGAGAGACGCGCGAGGGGAACAGCGAGCGCGCTTACGAGACTGCGCCCATCACACCGAGATCCAGCAGCCCGAACAATGGCAGCATCCAGCACCTGATTTACAGATGGAGACGTGCTGGAGCTAAAGAGCGCCGCTTTTTAACGACACGGCCCTGCTGTCATCGGAGATGGAGCGATAGAGGAAAGAGAATCAGCGGGCTGTTCTCCATCAAGTGTGCCGCAAGCCTGCGTTTGAGTGAAGGTCTCGGCCGTTAGATCGCCCCCTGGGGGCTGGCTGCAGTACAAGTCATAAAGCCCGCCTCCTC |
| 174 | lncMBr_037 | TCGGCAGCGAATGCTTTGAGATTGAGTGAGTGTTTGAAGAGCTCAGACCTGCAGAACTCACAGGGCATCAGACTCCATGATGTCAGGGTTTGTGACTCGTCTGTAGAATCAGAGCTGCCATTCACAAAAAGGCCTGTTTTTAATAGTGTCAGATAGCTAGCAGACACTCCTGTAGCTTAGCTTCGTCCAGCGTGTTCTTAGTGTAACCTGAGTAGATCATAGCACAAGGTGAGGGGCGGGGTTTAACCTAGTGGGTGGAGTTATTTGTTAAAAGTGGGCGGGTTATTTAACAAGTGGGTGGGATATTTGAAGGATTGGCGGGGTTATTTGACATAAGTGGGCGGGGTGTTTGAGTGAGGAACAAGGTTATTTGACAGGTGGGTGGGGTGTTTGAGTGAAGGGCGGGGTTATTTGACAAGCAGGTGGGGTGTTCGGTTTAGGGGCAGGGTTATTTGATATAAGTGGGTGGAGTATTTAAGGGAGA |
| 175 | lncMBr_038 | TCTGACTCTTCATTCACTCGTGTTCTTGCCCTTAAAGAGAACAAATGTCAATACGAGATAATGGAGCAGGTGGACAACTTTCATTAGCGCTTTAATTTAATTTGCATCTTTATCTGAAGCTTTGATTGACAGTTTGACACTTGGCGGTGCGGCGCGCGCGCGGCTGCCCTCCGTGTTTCAGTGGACGTCCATCTCTCATTTGACCATTTTAAGACTCTTTAACATGCCGAACTGCTATGTGCCGCGCTGAAAACAGGACTGCTTTCGTCTGTCGGTAACCTCAGAATATAAAAGCGACAGTTTGGCGACATTTCATATCAGTTCTCTTCAGACGCGCGCAGAAATGGATCTTATGGGAATCTACCTGCTTCATCTCAGCTTTGCC |
| 176 | lncMBr_039 | AAGCCATAGGATATTATGAATGAGCTTCTTTTCGTTTATATCCACAACTTCTTGATGCTGAGCATCTTTTCCTCGAGCAGTGTGAAACTGTCCATACTCGTTTAAGCCAGAGCGTTTAGTGTTATCGAACTGTTCACGACGCTATTTATGAAACGCAATTATCTCTCTTGTTGTGGCCAAGTGTATAAGCAGTCAAGAGTTACATTAAAGTTCACACGGCAGTTTTATATAGTTAGATGAAGTGCGTGCTGTGAGCAGAGGAGCGCG |
| 177 | lncMBr_040 | CAGGGTTATTTAGCTTTATTGAATCTGCATATTCGGCATTCCCTTGAGTTCAGTTTCTGGCTCTCGAAACTACTTTTTTTTAAATCTTCGTCGTAAGTTCACACATTTCCAAGGAAAAGTAGAAACTAGTAAGAATCATACAAGAGGAGCATGTATTTCATTCAAGTGTTTACATAAGCATTTACATTGCCTTGCCAAAAGTAATCAGGCCTTGACCAATTCTGTCATGTTACTGGATTACACAGTCCCACACTGCAGTTTGTGTTCTGTTATGTTGCATTTCAAAGCCCCGAATCTAATTTATTAAAACAACATTGTTTTGACATTCCGCACATTTTAATATTTCTCAAATACTGCTATTTTTGTTGGATTTGCAGCACATCCGAAAAGTATTCATAGCGCTTCAC |
| 178 | lncMBr_041 | TTTTCATTTCAATGTTAGTTTTAATCACTTTGTTATTATCACTTTTACTCACTCCCAGGGCCATTTATATCAAAGTTGATATTTAGCCGCACCATGTTGGTGTTTCAGAACATCTCATTTTACGAGGTGGGTTGTTAGTACTTAACCCCCAACCTGAAGGTCCAAGACATACACAGATACACTATGTAATGCAATTTAGCTTGCCCACTTGTGGGAGAAACCGTAGCACCCAGAGGAAACCCACGCGAACATAGTGAGAACTCAGAAATGCCATCTGGCCCAGCAGGGGCTCGAACCAGCGACTTTCTTGCTGTGAGATGACAGCGATACCCACTGTGCCACCGATGTTGTTTATTTACAAACTTTTTAGTTTTAGCTTGC |
| 179 | lncHL_001 | TAACTCTCTACCCTTTGGCCATAACACAGAAGTGAGAGAGGAGCAAAGTTCTTCTGCATTGCACAAGCCCAAAGTTACAGATAACAGAGTGAGTACAGGTTAAACTTTGGGTGAATAAGTAATTGAATTTGCTTATTTTGCTCTTGTTCTCTATTGACAGACTACACTAGCATACTAATGACGATGCCTTGACCTTGACGTCTTTAATGGGAAGTCTATCAAGCATGCAGTGCCTCTGGAAAGAAGCAAAACAAGCTCTTATACTGCTGATCTTTAGTTATTAGGCATTCATAAAGTCAAGCCTCTTGGCCATAACAGGGTCTGAAGTGAAGA |
| 180 | lncHL_002 | GTCCAAAATACTTTCTTGCAGCGCACTGCGTATTCTGTTTATTGGCGTCCCCCACTGTTAGCGGTGTAGCATGCTAACTTCAAACTCTCTTAGAATCCATACCAGGCCGGGGCAAGATTTGGCGTTAGTCAGTCTCTGTGGTTAACCAGCCGTGGTTAAGCAAGCTTAGCTACGCGCTCGAAACAAGATCTGAAACACCCACACTAAATTGACCATAACAAAAATGGTTGTTTGCTAATATTCGCTCA |
| 181 | lncHM_001 | ATTAATCCTGACCTCTTCTCTGGAAATCTTTATCACTTGATGAATGAGAACACAGTATAATGATGATCACAGTGGTTTATTTATATGAAATACGTGAGTGCGAGTCCATAGTGTGACGCAGAGCACATAAAAGCAGCAGCATCAGTTGAGTAATAAGATAATCAGTCGGTATCAAGTAAGACACAAACCCCCTTTTGTTATCATGTTGTAATAATCTGTTGCTTTACTTTTATTTTAT |
| 182 | lncHM_002 | CAGCTTTTATTCTTGCTTCTTGGCATGTTTTTTACAAGAACAGGACAAACTTAATTTTAGCATTGTTCACTGTTGCAGATTAGGGCTCCACAATATATCAATTGAGCATCGATATCGAAGACATGCAATGTTGAGTTAGGATTATAGATGACCAGAAACTACAGGTCAAACCACACAGCATTTGTGGAGACACTGCAGAATTGAACCGTAATAAAGTGAAAAGTTATCATCTGCATTTGTTTTTAGGGATTATTATTTGTTTTCA |
| 183 | lncHM_003 | AGTCGCGACTCCACCCACTTCCAGAAACAACTCCACCCATACTAAAGTAGTGGCCCCACCTCCTCAAACAAAAACTCCACCTCACAAACAGACCACACCCCCTCGTACAGAAGAACCGCCCGTCAGAGAGGCCCCGCCCCCTCAGAAGCCATATACGGAGCGTGAGAGGAACGTGGATATCCTGAATCACCTTATTAACGACATCGAAGCGTTCGTTGGCAGACTGACG |
| 184 | lncHBrBl_001 | GGAGACCAAATTTTCTTCCATTTTGAGCTACAGCAGGTCTTCAACACTTGACCTTTTGGATTTGTGCTGACCTCTTTGTAGCCATTACTATTTTTTGCCTTTTCATTTGCCTATTTGTCCTTCAACCAACAGATGTAATTCAAGAACTTAATGTACACTTGCTGCCTGAATTATCGTACTTTCTCTTTTTCCTTCACTTTTGTCAATTGGTGAAGTTTGTCTCTCACCACGGTCACCACTGGCTTGCTTGGTTTGGGATATGTGGAGCTATGTAATGGTGGAGTTACTTTTCAGTCTTGGCTTTGTTTTGGATTTCAGCAGTGA |
| 185 | lncHBrBl_002 | TGCAGCTGTTTTATATTATTCTTGGCTGATAAGATGACATTCAGAAATTTTCGTTTGTTCAATTTTAATTGAACATACATATATTATTGTCAAGCATAATGGAAGCTGTTCTTCTAATTGGGCTCTTCAGTTGCACATCTGTAATCATGCATCCTACAGGGTGTGACTGAACATCATCTTAGCATATTATATGTTCATTCTGTGCAGTTAGTTATTGATTATAGAGGACCTGTGGATAATCAACTATGCTCAACCGCAGATCATATATTCACAGTATTAGAAGG |
| 186 | lncHBrBl_003 | GCGAATACAAAACTAAATTGAAATTGTGGTTTTTGAGAGGAAGGCAGGTCACAATTGAGTAAAAATGACAGAATTATTAGTTATAATCTCAGCAATATGATGAATTGTGAAAAGGTTAATAAACTTACAAGCCTGAAAACAATTGAAATTTGAGTTCCTTCCAACTTTCGGTTAAATTCAGTTACCTCTCCTCAAACTGAATCAGAGTTGACAGTCAGATCTGCATAACAACCTCAGAACTGTAATTTAATTTGGCAGATTTATTGCATTACTTTTTGATTACAATTCTGAGGTTGTTATGTAGATCTGACTGTCAATTCTGATTACATTTGCGGTGAGTTAAGTGAAGTTAACCCAAAGTTGGAAAGAACTCAAATTTGAATTATTTTCAGGGTTGTAACTT |
| 187 | lncHBrBl_004 | CTGCATTTATTTACTGACTTGAGCTCTATAAAATCAATAGAACAGTGAACAAACTGGCTGTGAATCAGTAGAAACAGTGATAAAGGCAGAACTGAACAGTGTCACAGTGGTCTGACATCATTAATAAAACAACAACTGCACTATTTCAGATTAAATATTCTCATTTAACAGCTCATCAAGACTTAAGTAATTGATTACAGGTGAGTTTTACCATTTTTCAATCCACTCAGCCAATCTCAACGTCTGACGGAGCACTTTTAGCTTAGCTTAGCATAGATAATGGAATCGGATTAGACCATTAG |
| 188 | lncHBrBl_005 | GACGAAGTGAGGTATTTTATTCCCGTTGCTGTTAGAAAATATTCACAACTGGTGTCATTTGCTCCCGTACAGCTGTCAGAAAGTAAACCAGCGGTCTTTGTAAAGGACGCGGAGGGGCTCAGTATCCAAATATCGCGAAGCAATGGGTCAGCATAGCGGAGACGCAGAAAGCCAGTTTGAATCCCAGCTGCGATGAGTAAACAT |
| 189 | lncHBrBl_006 | CTCTAATATACATCACGTCAACATGTACAACATGTAAATCAGTGGAGCTTGATGCGTCATCCACGTCTGGTGTGAACGCAGCATTACACATTGCTCACTCACAAGGGAGAGGGTCCTCTGGCTTTTGCTGAAGATTTACTGCTGATTACGTCACTGTTTAGCAGTAAATGTCAAGCAAAGGCCGGAGGACTCTCGCATTAATGCGAATTATACACAATACCTAAATATGACCTGAAGAAACCCAGGCAATATCAG |
| 190 | lncHBrBl_007 | TTTTTATATAGGGGTGATTTATTTATGCTGTGTACCACTGTATATACAGCATATTTGATGTGTATGCGTGTGTGTGTTTGTGTGTCTCTCTCAGGGTGCGGGCTCTAATGTGATCCGCTGCAGAAAAGACGGCAACTGGACCGGAAGTTTCCGCCTGTGTCCTCAGCTGAAGGGCCAGTGCTCTCTACCTCAAAACCTGCATCCCAGCATCCGCATCAGCTGCAAGAAAGGACACGGCATAGGTGCAAACTCTCACCTCAAACATATGCTAAAATAAACAGCGAATAAACAAATTAAGTTGAACGTTA |
| 191 | lncHBrBl_008 | CACGACTAGATACAAATATTTATTTAAATAGCTTCACAAGTAGACACTATACTCGACACACTTTTGTGTTCATCTGTTGCAACCCATCTGGGGTTAAAATACAGGCTAATTTGAATGGCTGATCTAGGAACTGTTGTATAGAGCAGTATTCTATCCACCGAAAGGCTGATAATCTAAGTCTGTTCTGAGATCAGAGTGACTAAAAGAGTGGAAGCAGATAGTCATATCTGAATGAACACTAATCTGGCATGAACAGAGCACTTTGAACACATGTATGCCGTAGATATATGGGCATCTGCCTCCTTCATTTTATCATACAAAACAAACGCCGGTTCTTTT |
| 192 | lncHBrBl_009 | CTGGCATCTTTTCAAGACTGTGGACTCCGCTGAACCTGGCAGCATATGCCGCCTGGCTTATCGCTCCCCCGCCGGGATCCATCCAAATAATCCAGCACTCAAAGGCACATCACTTGCGTTTCTCTCAAACGGCGATCTACGATGTGCATTAATCGACTTAATCTGTTTTAGCCAAGTTAGTCGACAACACAATAGATTTCCTTCTGGAAAGAGTCATGCTAATGCGAGAGGCTGAAGTTGAGCTGTGGGCGTTTGGTGTGCTCCTGTCTGCAGTTTTCAGATGTTGCGTTTTACGGTCTTGATATAACTGGATTAATCAGGACGGGGGAAAACACAAGCATCCGCCCAAAGATGCACTACACGGCTAATGCACAGCCTTCTGAATTAAACATGGCCTGGAA |
| 193 | lncHBrBl_010 | CTGCAGCGTTAGGACTGTTGAGCGCCCCCTGCTGGCCTCACTAACACCACTTTCTGCAGCAACCTATCTTTCCCATGTGTTCTCCCATCCAGGTACTGACCAGGCTCAGCCCTGCTTAGCTTCGGTGTGCGACCATGTGAGAGTTGCAGAGAGCGAGCTGCCAACCATGGTGTGTGTGAGCAGTGTGTGTGAGATCAGAGCAGTGTGTGTGAGATCAGGGTGGTGTGTGTGAGATCAGGGTGGTGTGTGTGTGTGATCAGGGTGGTGTGTGTGAGATCAGGGCGGTGTGTGTGAGATCAGGTGTTTATCATCTCTGTTTTGTCCGTTGTTGATGCATAAATCCGTGCTGATCATGTAGATTAGAGCATTTC |
| 194 | lncHBrBl_011 | ATTTGGTCATCATTTCTCAAGACCCAATTAACTGGTTTTTATATATATCTGTAATCATTCTTACTGCAACAGTCACCAGGTTGAGAGCCCACCAGACACGCTAGAATGCACTCTACACATCACATCTGCCACTGAACCGGACTACATATCCCATCATGCATCTCGCACACACACCAGTTTATTTTGACCTTGCTTTGCATTCCCTGCATGCTCCTGTTTTGACCTTTGCCTGACGAATCGCC |
| 195 | lncHBrBl_012 | GGTTAATGCGGTAAAGTTGCAATGTTTTTGCTGTCATCTAGCGGTTGATCATAATGTAACATTTACTTCCCCCATAGATTACGCTTGGTGTTTTGACAAAAGATTCATAATAATTTTGAAAGGGTGTTTCGCTTTTTCACTCCAGCGTTTGTTGTAGTTGTCATTTCAGTTATTCCTCTTCTCTTGTCTTGTGAGCTGTCGAGAGACACAATCCTAAACGCCGGTCTTCTCCATGACCGTGGCTCCAACCTCAGAGTAGCCGAAGTCGTCGAGTTGTTGCTGTGCAGCGGAGGCCTGACTGTTCTCTTTCGGCTTAATGAAGTCTGTGTAGACGCACGGAAGAGGAATCACTTTTTCGTTGTCTG |
| 196 | lncHBrBl_013 | GCTGTTTTCACTTTGCACTTTACGTGTCTGCCCATTTCACTGGCCTGATTTGACAAGGGGGCTGAGTCAAGGGAACCTGTGTGTTCACGGCAGGCCCAGACCTGTCACCATTTAAGCAACGTCATAAACTGGAGACACTGTTACACTGAGGTGGTCTTACAGCTCAGGCCTGCTGTAAACTTCCAAGTTCCCAACATAAGTGCAAAGTGTGAACAGCATCGCAA |
| 197 | lncHBrBl_014 | TGTCTTAATACCAGGTCTGAACAGGGCCTTTTTTCTTAAGCTGAAGTCAGCTTTTTTAAAATCAAAATTAAATCTAAGAATTATATCAAGATGCAATTTAAAACTACTCAAAACAAATGTAATGTGTAGTTTTATGGGAAATAAACTTTTTTTTTTCCATTATTATCCAAACGCAAGTTTCTATAAGCTCTGGAAAATAATCAAGTGACTGTTATCAGACCTTGTTGTTGCTATCCATGTTTGCCATCCCAAGATATTTTAGAAATCCAGACCTGTTGACGATGTATGTTTAGATTACATACATCTATACTAGAGTGATTTATTGTAGTGACGCAGAAGATTGGTCATATATGAACAGCATAAATCAAATGTCACGGTGTCAATAATACTGTATCATTAGGTACGTCTTGTACATTGTACACTTGTATTTCATTTAAGCACCGCTAGGCGTCACAGTGGCTTCATGAATGACTACTGACAAAGACGCAAATAAAGTGACAATAATCATGCGTAAACAAATAAAGTTTATATAAG |
| 198 | lncHBrBl_015 | TGTGCATGACGTTTAATAACATAATCATAAATACAATACAGTCTGGACTTCAGCACACATGCTCTAGTTGCAGATCCAGTAAACAGGATCAATAATAACCCATCTCCGCATCAGTGATTGATTGTCATCTCAGGCACTTGAGGAGAACAGCAGAAGTTAAATAAATCACCAATAAATGCATTCATATTCATATTTCACATTCTCTTATTGCTCGAGGATGTAACCCAGGTCTAAACGAGATAAATACATTGTATTTTAAC |
| 199 | lncHBrBl_016 | GGAGGTTTTGGGTAACTAATCTTTTTCACTGGTTTCCCATGCTCTCTGTCCAACCCATGTCTTTATTTATGGACATGCCACTTCCTGCAGGGTAGAAGCTAGTAATTATGCACATGAAAGTTCACAGACAGAGTAAGTGGTAAGTTTAAACCCAGATTTAGGGCCTGTCCCAGTTCTTACACTTTCAGTCTGTCCCAGGTCTAAAAACAGCAAATTCAGTGCCCTTAACACACACTAAATTAATCCACAAACTCTAATATTGCTTTAGGGCAGTATTTGAGGCTATTTAAAAAGTATTTCATCCATCGTCATGTTTGGGGAG |
| 200 | lncHBrBl_017 | TCATTTGCGCTCAGGATTCTCCGCTCGGCTTTCAGCTCTGGCTCTTCTGACGGACACACTGATCTGATTTAGGATGATCTGCACATTACCAACAACTCACAGCTCTGACAAAGAGTTGGAGGAGCAACAAAACACACGCTGACACACACACACACACTGACACACAGACGCAGTGAGCAGGGGTTTCTCTTATCGAGAGGGATTCCCTCACCCAGAGACACAGAGGTG |
| 201 | lncHBrBl_018 | GCGCGATCAACACTGATGTCAGAATCAGCACCAGCAGCCCTCGAACCCGCATCCTCTCAGCTGCTCACACACGCGCACACACTTCAGTAAGGCAGCACTCAGCTCAAAGCGCAAGTTCAGCGCTCACACACACACACACACACAGACACACACACACACTCTACAGTGCAGTACAGGACACGCTGCAGCATGGAGTCCGCCGTGCGCGAGTTCTCTGGGTCAGTGGAGGCTCTTCTGCTCTGTCGGACGGGGAACCCCTCACCTGCTGCTGCTCGC |
| 202 | lncHBrBl_019 | GTTGAATTTCAAGTCATGTGAAGCCATACGATGTCTTTATATGAAGAACGGAGTGATTTATGAGTCTCTAAAAGCTGAAAATGGCTTGCTAATTTCCTCTAATTAAAAAAAAAATAGCTGAAGTATATTAGCATGATGCAATTAGTTACGAGACACAAAGAGAGCCAATAGCATTCGACAACTGAAGAGGATGTAAGACGCTGGC |
| 203 | lncHBrBl_021 | CAATGTACTGCGACTATTTGTGCTGCCGAGAGTTTAAGAACCAAAATCCCAAATCTAGACTGACGAAATAAAACCTGCTGAATACAAAACATGAGAGTTGATATATGAGTGGTGCTAAAAAACAGTGATCGTATCTGAACTATCGCCGACACTGTATCCAGTGATAATCTCCAGGAATGTCATCAATATCAATACAATAAATAGTGAAAGGTTGTGTTTGGGTGAGAGCGAGAGTGATATTAAGCTCTCGGCGTCAAGTGCAGTTTAGCTGCCGAACAGGATTACAACA |
| 204 | lncLBrBl_001 | AGAAATTTAGTTTATGTCTCACATCTTGAACAAAGGGAGATTTAATAGAGTAATGCTATAGCTGGTGGGTAAACAAGTTAGCACAACAAAACCACACTAGCACACATTCAAGCCCCAAGCATTTTTTTTTTGTTTGTGCATGTGAAGTTTTTGAGATGTGACGTTATTTTAAATGCTGTTTTTGTACATTTTAGCTCATTTTACATTCTTTTTTATTACATAGTTCAACTAAAATTCCACTGGAAGTCTGTAATAATCTGTTTAATAACATTCCACTGTGGTGACCTCTATATGACCATATATCTCACCACAGATCAGTGTCTTTTTTAGTTTTTGTACGTTTTCTGTAGACAATTGATTAATCAATAGTAATTATGGAAAGCGCATATTCTTGTAGGTGCTACTTTATAGCATTTGGC |
| 205 | MBrBl_001 | CATTACGCGCAGAGAAACCACAGAGCACAGTCTGCGACAGCACAACGGAGAGCACCGCGAAATCTGTAAACGGACAGGATTTAAGGAATTAAGCCCCACTTTGGAGATCTGATCGCCTCTGGAGAGTATTTTGGTGGATTTTCTGCTTCTGGGGAGCTGCGAGGATGCGCGTATCGGTGCTGCTGCTGCTCTGCGCTCTTTACGCAGGAAACGCGCACAAACT |
| 206 | MBrBl_002 | ATTGTGTGGAAACCAGCATTTTTTTACAGTGCTGGGTCTTTTCCTCTTCGCAAAGAAACTTTGCAGAGATATCTGTTTGTTACTCATTTTGCTGGCTTGTGGGTTAAATTTTGGCACTCAAGTGACCGAGATGTAATCGAGCGAATGCGGTCACTTTTCAAAGCAAAAGATTTTTCAAAATAAAAGCTCAATCAGACTCAAATAATCACTAAAACATAAATAAAGATGTCTTTGGCAACCCGGTACCAATTGATACCCGGGGCCCTTGGGTTGGGAATCAATAGGTGACTTCTAGTAGGTGACAATAGGCTGCAATAGGTGACTTCTAAAATGAGCCTATTATTTTATAAAGTGGAGTGTTTCTTTAAAAACAAACTTAGCAGCACATCATGTATCCTTTAAAACTTGTTTTAATGCATTTCAAGTGCGCAGCAAAGTGTTAGCGGCCATTACTAAACCCAACACTGTTGATTCAGTTAACTGGTACTGTTTTCTAAATCTATTAAAAGTCTAATCAAATGTATGCTGAAGTTTAAAAGGTGATAGTAATGACAATTCTTGTGGTCCTGGTGATGGTGGCATTTTGAATCCCTTTTCTGTTAAGAGCTATAATCTTTGTAAGCTTGTGTAAGCCCTTTAATTCTCGTCTCAGTTGATTCTAAAATAAACTTTTTTGAAAGAAAAGCGAA |
| 207 | MBrBl_003 | GACAATCTGAACCAGGCCCTCAAGTTCCCAAAACTGCACACATTCTCCTTGATCCTTGCAACATATATTATCATTATATATTAATAATCCCAGGTCACAGAACTCACCACACACGTATAAAGCAATGCTTCAAGCTATCATAGGTTTCAAAACGGCACCACTGCAACTACATTGTGAATTTGTTTTTTAAAGTCGGATTACGGGTGCACATGGATTCAGCCTGAGGGATTGGTGCAAACTGCTGTTGCTTATAAAAACACAGTTCACCACAAAATGAACATTTACTCACTCTCAGGTTATTAAAAGCCTCTTATATTTCTTCTGATGAGTTTTGAATAATGTTGGTAACCACTGACCCATTATTTGAGCTATAATTCTAGCTCTGGGTTATGTTTTAACAGCTGATGGCGCTCTAGTGGCTAAA |
| 208 | MBrBl_004 | TGAATAATAGCCTTGCCCTAAAACACTGATAGCCCCACCTTAAACCATTTATAGCCTCCCCCTATGACTCATAGCCCGCCCTAAAACACTGATAGCCCCGCCCTATAGGACTGATAGCCTCACCCTAAACACAAATAACCCCACCCTAAAACACAATTATCCCCGTCCTACATGACTCACAGCCCCGCCCATTATGACTGATTGCTCTACCCTTAAACACTGATAGCCCCACCTTATAGGACTGATAGCGTCACCCTAAAACACAATTATCCCCACCCTATATGACTGATAGCCCTGCCCTAAAACACTGAAAGCCTCACCCTAAAACACAAATAGCCCCACCCTAAAACACAATTATCCCTGCCCTATATGACTGATAGCCCCGCCAAATAAGATTTATAGCCTTGCCCTAAAACACTGATAGCCTCCCGCTAAAACACAAATAACCCCGCCCTATATGACTGATAGCCCCGCCTTAAGCACTGAGAGTCTCACCCTATATTACTGATACCCCCACCCTAAAACACTGAAAGCCCTGTCCAATATGACTGATACACCTGCCCTATATGACTG |
| 209 | MBrBl_005 | GAGCTTGAGTGTGTGCGCACATATACACACTGGGGCTGCGTGCTGAAGTTGATTCTATGTTTTACACCTCTATATTTGACTTCCTCATGTACTGTATGTGAGTTTCATCAGCACCTGAGGTTTGCAACACTCTTCCAACTGCTCAGGTGTGTCACTTCTATTTATTATCCTACACCATGAAACTCGCTGTCGTTCTGCTGCTCTGTGGCTGCTGTGTGCTGTTTAAACACAAC |
| 210 | MBrBl_006 | GGTTTATGCACTTTTTGTAAAGACAATTAATCAATTTAAAAGCAACGAGTTTACTTACTTTATTAAGTAAAGTCAACTAATCGCTTTACAACCAACAAGGTTACTCACTTTTATCAAGTAAACTAATCACTTCGACAGCAACAACTTTACTCTCTTTTGTTGAGTCAAGTCAACTAATCACTTTAAAATCAATGGTGTACTCACCTTTATCATGTAAAGTTAACTAATCGCTTTTAAACTAACGAGTTAACTCACCTTATGAAGTAAAGTCAACTAATCGATTTAAAGCCAACAGGTTTACTCACTTTATTAACTGAGTCGACTAATCGATTTAAAAGCAACACGTTTAGTCACTTTTATAGCCAAATAATCGCTTTAAAACCAAGGAGTTTACTTACTTGTATTAAGTAAAGTCAACTAATCGCTTTAAAAGCAACAGGTGTACTCACTTATCAAGTAGTCGACTAATCCCTTTAGAAAGCACCAAGTTTACTCACTTTTATTGAGTAAAGTCAACAAATCGCTTCAAAATGAATGGGTTTACTCTTTATTAAGTAAACTAATTGCTTTAACAGCATTGGGTTTACTCACTTTTATTAAGTAAAGTCAACTAATCGCTTTAAAATCAACAAGTTTACTCACTTTTATTAAGTAAAGTCAACTAATCGCTTTAAAACCAATGGGTTTACTCTTTTTTTATGAAGTAAACGAATTGCTTTAACAGCAACAACTTTACTCTCTTTTGTTGAGTCAAGTCAACTAATCACTTTAAAATCAATGGTGTACTCACCTTTATCATGTAAAGTCAACTAATCGCTTTTAAACTAACGAGTTAACTCACCTTATGAAGTAAAGTCAACTAATCGATTTAAAAGCCAACAGGTTTACTCACTTTATTAACTGAGTCGACTAATCGATTTAAAAGCAACACGTTTAGTCACTTTTATAGCCAAATAATTGCTTTAAAACCAAGGAGTTTACTTACTTGTATTAACTAATCACTTTAAAAGCAACAGGTGTACTCACTTATCAAGTAGTCAACTAATCCCTTTAGAAAGCACCAAGTTTACTCACTTTTATTGAGTAAAGTCAACTAATCGCTATAAAAGCAACAAGTTTATTCACTTTTATTGAGTAAAGTCAACTAATCGCTTTAAAATCAACAAGTTTACTCACTTTTATTAAGTAAAGTCAACTAATCACTTTAAAACCAATGGGGTTACTCTTTTTTTTTATGAAGTAAACGAATCGCTTTAACAGCAATGGGTTTATTCACTTTTATTAAGTAAAGTCAACTAATCGCTTTGAAATCAACAATTTT |
| 211 | MBrBl_007 | GCTAAATGAGCATATTAGGATCATTTTTAGGTCACATGATACTGAAGAGTGTAATAATGAGACTACTGATGCTAAACGTTCGTCTTTGATATTATAGTAATAAGTATAGTTATTTGTAATCAATTTGTTTTGGTAAAACTTTAGTTAAAGGATCAACTCTCACCTTTAAGTCACTCTATAGTGCCTGAGTACCATTATTAAGATGTTGGCTGTTAACTTGTGCATAAGCTAAAAGTCATAATTAATGGTTGGTTACCTTCCTGCTGCGTTCAGGTCAAATATGACCAATTTACAAGTTGAAACACTCATAGGTATTGTGTTTTACATCTGATTGCCTCAAGGCCTTATAATATCCTCCACAGTATGCACTTAATCATGTAAAAGTGATGATCACCTCTTTCTTTGAATTCTGTTTGTTTTAATTGTCCATGTTACACATATGGTGTTCCCGG |
| 212 | MBrBl_008 | TTAGGATGTGAGCGTAACTCAAAATAAACAAATGCAAAATAAAGATTGAATACAAGTGTATGATGGGAAGTTTGGATCACTTGGTCTTAATTAAAATCTTACACATTGAGTCCCTGGCATAATTCACTACTGCTGTCCCTGTGAACTAGACAAACCTACATTGCAGCCATGAATATATAAGAGGCAGCCAAGATTTACTCATTGTTTACCAGGGTCTCAAGTTAGCTCCACTCATCTCACCTGTCATCTTTTGAGTCCTCAAGTAATTCACTGCCTTTGTCCTCACGCCACATCTCCATAAATTAAAATGTTACGTGGGCTCTTCCGGTTTAATAATGGAAAATATTAGTACATCAGTCTTAAGTCCTCACATTTTCAGTCATCCATTGAGCAATTAATAGCACTATAGGCCAAATATTAAACTCGAAGGGTCAATTGTTGGAGTTTTGGTTATTTGCTTTTTGAATTGCTTACTTGGGACCATTGGAGCTGCCATTTAAGAACTATCTTAGCATTCTACACTGCAAAAAATGC |
| 213 | MBrBl_009 | ACTAGTTAGATTAATACAGCAAATCATCAGAGAATAGTAGTTTGCTCTGTAGCCAAGTGAAAAAAACAAATCTTAAGGGGGCTACTAATATTCACCCTCGCCGGTTAAAATTTTTATTCCTTAAAATGTATTCTTTATTAAATGCTGTGAAATAAGTCATTATTATTTGGGGAAAATAATACAAATTTTCACAGGAGGGCTAATAATGGTGTCTTTTTTACATGTATACTAACGCTTCATAAAGTATTAGTAGATTGTTAGGTTTGGGTTGGTAGAATAAGTCGACGTAATAATGTGAAGGACAACCAAATTAAAGTGTGATATTAAGCAGACCATTTTTTAAAGGCAGAGTTCACCCCAAAAATGTACTCCTGAAATTGTTTCTCTCATCTGTTTAACATAAAAGAAGATACTTTAAAGATACCCAAAAACCGACAACCATTGCCTTTTTTTTTGAGAATTTTTTTTTTCTGCCGT |
| 214 | MBrBl_010 | ATAAATTGAAGCTTAACTAGCTAGCCAGTTAATCCAGTTTCATGGTATCTAAAGCCTGGGATTGGTACAAACTAAACTTGAACCTAAACCAATTTATCCAGCTTCATTGTACCTAAAACCCAAGATTAATGCAAACTAAACTGAATCTTAACCAAATTATCCAGCTTTGTGGTATCTAAAACCCAAGATTAGTGCACACTAAACTGCAACTTATCCAGGTTAATGGTACCTAAAACCTAGAATTGGTGCAAAATAAACTGAAACTTAACTGGTTAGCCAGTTAATCCGGCTTTATCGTACCTAAAATCCAGGATCAAAATCAAAGATCAAAACATAACCTTTCTGGACGGAAATGTAAAATATGAAACATTCGCTCGCTGTAAGTGTATAATCATCTTATTTAATCCCACTATACGACAGAATTATGCATGCTTAAACTCTAGTGTGACTGCGGCATAAGTGTTATCCCTGCAAAAAGGCTTTTATCGCCCCCTGTTGGTTCGGCGTGCCCTTAACATGCATCACTGTGCAGTTTTGCCATTTTATGTGGACGGAGACTGCTTATTTCTTTAACGTAAGTGTGAAAAACTGTTTATAAGAATACCCATGTACATGTGGACATTGCCT |
| 215 | lncHLBl_001 | CTTTAATGAAGTGCTTGCAGCAGTTGAACAAAGCACAGATGTGAACAGAGGTGAAAGGGGTCAGATCAGACAGAAACTGCATGAGTTATACCGACAGATCCTACGGTATAGCAGCAAAGGTCTATTGTCTGGCTCCTTGTGCAATGTTCCGCTTCCCAATCCGGAGATGAGTTTCCATCTGTGGTGGGAGGAAGAGGAGCTCTGTAA |
| 216 | lncHLBl_002 | TACTACGATAAATGTTTGTTAATAGATAGAAAAAGTTTACTCTGACTCTGCTAAACCCAAGTGTACTGTCGTGGACTTTGTGCCTGTTATACTTTACTAAACATCTTCATTGAGATCTTCAGCGTGTCCTAATCAATGCCATGTTGTGCCATGCATTATACTCCAGGTCTGTTAAATGTTTAACCGCAAAGAGAAAGAAATTCTCTGTCTAATAACTGCATCACTGACTGCTGGCAAATGTCTTAAATATAATTAAGACAAGATTATATTAAAATGTAATGTTGAAATACAAAACTTGATCAATGTTATGTAGTAACT |
| 217 | lncHLBl_003 | GGAAGTTAATGCTGAATGCACAGTGGAGGCCAGTAGTGACTGTATTGTACTCCCAGAGCAAGATGCTAATAGTTGTTTGGTCATAATCCAAGGGCAGGATGGGCTAAGTTCAGTGGCAGAGACGGTAGAAATTGAGACAGTGGTATGATGGTAGGATTTTCCATTTAAAATATGATCTATTCACATTATGTCACACAAAAAGGAAGAGAAATCGGA |
| 218 | lncHLBl_004 | AGATGATGTCACCAGAATTTCACTGATGATCCAGAGTAAACAATAAACCCAGGATAGAGAGTCTGGGTGAATGTGGTCTGGACTGAGTGGATGAGGCTCATTGTGTCTTTATAGATATTATAGAAGGTCAGAGTTCCTGCACTGTGATCCACAAACACTCCTATTCTCCTGCTGATTGACTTTACTGGGAGACGAATCTGTGTGTTATTGTGTCTGAATGAGCAACTGGAGGGAGAGAAACTCAAACTCCAGGACTGATCATTACGTCCAAACCAACACTCTTCACTGTACCCCTTTCTTATGATGCTCTTATATGACACTGATATAGACACATAATCTCCACTCCAGTCAATCTCCCAGTAACAGCGTCCACACACACTCTCTCTGCACAACACCTGAGGATGATCCTCAAATCTGTCTGGATGATCAGGATACGACTGTTTCTCTTCCACTTTTGTCACCTTTCTGTCGTCC |
| 219 | lncHMBl_001 | TGCCCAAAAGTATTGAAAGCCTCATGGGAAAAAGCATGTTTAGCCAGTAGATGCCATTCCATTGCCATTGTCGAATGTGTTTCAAAACAAATTGTGATATTTATTATCTGTGTTCATTTGGTTGAACATTTATGTTTAAATGACAACTATCTTGAGGCTGCTGGCTATGCATGTTGCCTGATTGTGAATTATTACATGCTGTATGTATTATGTTTGGTATTGTCGCAGGAGAGCTTTTGACCTAAATCGACAATGCTGATGCAGCCATGTTTGTATTCTGAGGTGAAT |
| 220 | lncHMBl_002 | TGAAATACTGACATCAACTTTTATGATGCAGTTTACACACACACACACACACACACATATGAATCATCCTCTCACTTTTACAAACAAACATGCAAATTAACGACGAGTGAAAACATGTTGAACGATCCTAAACGATCTCCTGAATTCTGTGTGTGTGTTTGAAGAGTGAAGAGGCAGTGGTCTGTGACTGGGTGGAGAAGAGTATGGAAGGAGAGGTGACACAGATGTGGTGACTCAGCACACACACGCACACACTGACTCACAATCACAACCAGGAATCTGTTTATCTGCAAACCGCTTTGGATATTAATTCTTCTCACTCCTACATATTCTGCTGCCTTTCATTTGTTTAGTTCATTTAGTTAATTGTTCATTTTGTTAATTGTTAATTAC |
| 221 | lncHMBl_003 | TTAGAGTAAATCTGAACTGAAGAAATCTTTGCTTGCTCTCACCGTGGAGTCTGTCATTATGGTCAAAGGACGAGTCATCCGTAGACCAGAGGGACAGGAGAATAAGGTTGATATGCACGTTTATATGTATATAAAGAGAAAAGTCTTGTAGCCTTATGTTTTATGTAAAATACATGTAAAAGTGTTATTTTTTTCTTTATTAGAACATATCCACTGGAGAAATTGAAGTCTGTGCTGAGAGCATTGAAGTGTTGAATACCTCCCGAAAGCTGCCGTTTGAGATCAAG |
| 222 | lncHMBl_004 | CTGATATAAAAAGGCTTGTATAAACCATTACAAAGAGCATCAAATAACGAAAAGTATGAATACACGGAGATTATAAGCCTGTATATGATAACAAAAAGATGAAAGCGCTCTACACATCATGGTAGAAGATGGTAGTGAAGCTGAGCTAAAGTGCAGCAGAGACTCCAAACGAGGCGTCAGAAAGACTGAGGTAGCCGATACATGATGACTACACCAGACCTGCTTCCACCACAGGACGACTAAACAGGGCGTTCATAACAGAGATAGATCACCCAAAAACGAAAATTCT |
| 223 | lncHMBl_005 | TGATTTTCTTCCTTCAGTAGAACATTAAAGAAGATTTTTAGCTCAAACTGCTTCTAGGCCAACCAAAAATGAACACCCGTGGCTTGTGATGACTCAGTAGTCTTGATAAAAGTGAAGTAATCAGTCTGTGCGTGAAGCTGAATATTATTAATAACACTACTGCCTTTAAAGGTGCACTAAGCGATATGTGAAAAAACTCTCTTGACCACAGTGACGGACTCCCAATACACACTTGCAGCCGTTCAGCAGTAAGGGGCGTGTTTTATAACTTGTCTTGAGATCTTGGGATTTTTAAAAGGGGGTGAGGCCTTGTTGAGTTGTGCGTTTTTAAATATTTGGCAAAAATCATTTGGTGCACCT |
| 224 | lncHMBl_006 | GCAAGATTTGCCTCAAAATTTCTGAATTTTGAGGTGAGTTCTATATATTGTGATGAAGTAAGGGATTCAATCCCATGAGATTTGCTACCTTTCAAACCTGAGACAATATTGGCTATTAAGTTGCCCAGAATGTACAGTATGTGTGGGTATTGAAATTGTCTTTCAGATGGACCTTTGTTTATACTGTATGCTATCACACTAATAACTGGTATTTAGGAAAACACTCTTGCTTGCATGATATTAC |
| 225 | lncHMBl_007 | TCTGTGCATTATTGTTTTGAGATGTTTTTAAGAGTAATTGATGTGTGTTGGATCAGATTAGTCGACTCTTCAACACTTAAAGTTACTCCAGACTGAGTTTATCTACTAAATGTGACTAATAGTGTGTCATTTCCCCAGTTCTAGTGGAGCTCTATCAGCTCTGTGCCTCTGATATGAGAGAATCATTCAGTCTGTCAGAGCTTCAGCCTCCACTAACCCACTATTGATTTATTTTACTCCTGGGCCTGGTTTTTCAAAAGTAATCTACTAGGATTTTGGATAAGGGATTAGATCAAATCTTG |
| 226 | lncHMBl_008 | ATTGCTTCAAGCAATTGGTGCTGCTGGATTGTCTGCCGCAGCTAAAATTGTTTTTGGCGCCAAAGGGGCTCTGTAGTGCAGGTGCTGCAGGTGAAAAGCCGGCCTGAGAGACGGTTCATCTGTGCCTGACAAACCCTTCACTACATGATATTAACTTTTAGCTAATTTACGGCCATAAAAGTAGACACTACTAACTACTTTTAAATGTG |
| 227 | lncHMBl_009 | TCCACGAGTCAGCTCCACCTTTACTCCTCTGTCTCTCTCTGTCCAGCTGGCATCCTCTTCATACACAGCCGTTTTTTCTGCTACTTTTCTCCGCTTTACAGCGTCTCAGTGCTGCAGCGGCTCTCGCGGTGCTTTCTGAATGCTTTTCGGGCTCTAAGATTGAATACAAACAGTGTGCTAAAGACTGCTAGGGGAAACTGGTTAACTTGAG |
| 228 | lncHMBl_010 | GCATTACATAGCCTTGCAGATCTGGCATTTAGGCTGTGTGGTATTTCAAAATCACGACCTTAAAAATCTATTTGTAAAATGTAAATCTAACTCCCACTAAGACAACACTCATCGGAAACTCTGCCATCATACGCCATAGGAGCTAACTCAGTCACTGTTTTCTAAAGTAATTCAGCGTTGACTGTCAGGAAGAAGTTTTTCACTCATCATAGCACACGTGCTGCTGCTGATGTGACAACAAAAGTGATTTGATTTGAAAGAAGTTCACTGGTTGCAGCGCGGGCATGGTTTTGTACGTAATGGCTGCAAATGGGACACTTGCAGGCACTCTTCTAGAGCCTAAAATGAGAAATAGGACTATGGACCTTTGCAAGTGAGAGACAAACCTTTAAAGTCTGC |
| 229 | lncHMBl_011 | ATTTACACAAGTAAATAAACAGAATCACTGATGGGCAAAAAATATGTGGAATTCTGCACGCGCAGATTCTGTGTGGGCCTAGCTATAATGTATGTTACATAATGAATATATTGTTTAATTAAGGCTATAGTGTGCACAGAGGGTTAAAATAACTTCAGACTGAGTATTTGTGTGACCCAATGCCAGTTGTGCAGAATCTGGAGATAATTGGAATATAATGTGTGTTTGTGCAGTATGATGTAGCCTATGTTAAATAATTGATATATTGTTTAAGCTTTTAGGGTAAAC |
| 230 | lncHMBl_012 | AATTACACTGAAATCTGATGTACATAGCATCAAGCATAAACAAACACCAGACACATTTTTTACACAGCTTCAGCCTTTGAGAAGTTACATTCACACACAATTACACAGAGTCAGTTATTTGTGAATATTTGCATCATGTTATTGATTGTACAGATTATGCTTGTATGGCACTAAACAGCACATAAACTGGCTAAATATATTCATTAACCAAGGGTTGAAC |
| 231 | lncHMBl_014 | AACAGGAAACTAAAAGAAGTTGACAATAAACGCCTTTTTTGCTCTCAATCATGTACTAAACTCCTTGACATGTAATCTGTCAATAGTTTGTAGCAGAGATAATGCCGGCTGCCACGAGTGAGGTTGATTGGCAGCTTGGCTTGGCCCCTCCCTCCTGTGGTTTGACGGGGGGTAGGTGAGAGAATACAGAGCCGGCCCCTTCCTCGCCCGCTCCTCCCTGCCTCTCTCTCTCTCTATCTCTATCTCACTCTCTCTCTTGCTCTTGGTCTACGGTTCTGCTCCTGTACACACGCACACACACCAAGC |
| 232 | lncHMBl_015 | CTTTTTTTCTGAATCAAAAAATAATGAAAAAATATCCTATTTCACATCTCACGCACGGACCTGCTTGGACAACACTGGAATACATCAAATCCGGTCGTCTGGTCGCATACGGTTAAGTCGCAGGAGTTCAAATATTTCAATGTGTCCGCAGCTCAAATCAGATCTGAATTTTCCGCATAAGAAGATGATGGGAAGTCCCGGATTACTCTCTATTGGAAATGATTGACTTCCGGACTGTCGCTTGTCATATGCAGTGGAAAGGCGGCTTTAAAAGGAATGGGAGATGAATGGTTTAATTCACGTAGTGCTCAAAACACACCCATAACTCATTAGGAGTATAAGCACAACCCTGTTTGACCATGCGCCAGGGAGCAAACCATATTTTCAGACACACCCTCAATGCTTTTGCAACATGCACTTTAGACTTAAAGCCCTA |
| 233 | lncHMBl_016 | GAGCTGTACAAAAAGAGTAAAGTACCTTTTTCCAAAATATTGCGATGGAAACATACAAAATTTTGTACAAATAATATAAGAGTCTTAGCTGTACAATAAATTAAACATTTTTTTAAAGTTGCAGATAAAGGGAACAGTCACCGTAGCTTAAACATTCTCTTATTTACCATAACATCAAAAATAAATCACCCAAAACTCTGCGTGCAATCGCTCACACGCACACACTTACACATAAACAAGAGGTATTAGTCATCAGAAGATCCTGAACGTAATCCTGCTGGGTCAAAATATACTTCATTCTTTAAAATATATTACAAACACCTCGTCAATTTCTTATTTTTGAGGCTCTCGCAGGGCTCAGGAGAACCGAATATAGCATCTGGTAAATTATTATTCATTTAAATCAGCAATGCTCAAACTAGGGCCCGCGGGCCAAAGTTGGCCCATGGTAACCTTTGATTTGGCCTGCCACTGCCTCTGAGAAGGGAGAGAGAATGATGGGAAAGGTTGAGGCAAAACAGATTGCTAATACTAATTTAACATAACTTTTTGCTTGTTTGTTTAATTTTCGAGCTACAAAAAAGCTAACTAAAATTAAATGTTTCAGTTAAATGTTCT |
| 234 | lncHMBl_017 | TCTGAAATCATGTGCTTTGAGTTGCTTTTGCACTGTTTAGTTTAGCGCTGCTTTAAGCTCATAGCATATTTACACTTTTAGATGTACACGAGGGTCTGACACACAAAAATGAACTGCTTTTCAATCCTCTGGTGTCAACACTGAACCGGCCTGTTATTTCAGTTTTTGGAAAGAAACTAAGACTGAACAAAAGCAACACAAGACAACACTGAAAATGGCAAAAATAGCCATTGACAAGTG |
| 235 | lncHMBl_018 | AACCAGTTTATTGCTCTCATATTGAAATGGCAGCACACATTATAATGGCAACTTAAACAGATAATTTGTTAAAACAGGGCAAAGACCTTATCATTGCTTTGCAGACAGTTGTAAACATAAGCCTTCATTCCTCTTGATGTAGTTTACACAGATTTATGCCAGTGAAGGATAAAAAATTATTTAAAGTTGAAAAAAAAGCTATACACCTAAAATACCATGCATGAAATCCCCAATACTTAAACTAAACGATTTCCTAAACACA |
| 236 | lncHMBl_019 | GAACAACTGAATCGATTCACAGGTGTGAAAGCACCCTATATGTTCCTTCACATCATTAGCCACAAGTGTGATCAGTTTTGAGTTATGTTCAAGAGGTGACACCTGTGCATTAAATGTCTTTGACTTTTGTCACCTGTGCTCACCATTATGCAGCACTGGTACATCACAATGAAAATGTGTTGGCAAGTTGAGTCTAAACAGGTAAAAAGTACGCATGGTTTTGCCAATTGATTCAGTGGGAATCAGTGGGTTCAGTCAACTGAGCATTTGGTTCGGACAATACGGTTTATTGTTTTAGCAGTTGAGAAGAACTGTAACAATTGTACAGCCTTAGTGATTGTAACAAAATTTTGTAAAAG |
| 237 | lncHLBr_001 | ATCCCTTTAAATTCTTTAACGTCTTTTATTCTTTTGATTGTTCAACTGAACTAAATTAGGGGTTTGTTTCCCAAAAAACTAATGACAAATAACTAGCATAATATAATGTTATCATATTTGGCTCGCATAGCTTTTGGGAAATTCTCCTCTAACTAATCTCTATCAACATTGGCTCTAAAGCCATCTCAACATGCTGAATTAGCCGTACTGCCACAGACATGAACACACACTTATGGAACAATAGACAAAACTAGCATATTTAACCTTGGCTTTTTGGCATCTTGCTGCTAAATCCTTCTATTAACCTATAGCCAGATAACAGCTTTTGCCCACTGGGGTCAACTTGACATTATTTACCAAACCTTTAGAATGTTATTTTCAAAACGGTTTGTGTCAAGTAGTTGATGTCTTGTATTGATAACTTAAATGTCTCTTTAAATAAAC |
| 238 | lncHLBr_002 | CTTTAATACTTGAGTTAGTGTTACCAAACAGTCTTCGAATAAAATTTAAGATATTTTCAGTAGTAGACATGTCCGAAATACTATCTTGCAGTGCACTATGTATTCTGTTTATTGGCGTACTATGCTGTTAGTGACGTAGCATGCTAACTTCAAACTCTCTTAGAATCTATACCAGGTCAGGTCAAGATTTAACGTTAGTCTGTGGTTAACCAGCCCTGGTTAAGCAAGCTTAGCAACAAAATCTGAAGAACACACACTAGATTAAGCATAACAATAGTTGTTTGCTAATATTTGCTCAGGAGTAGGTTTATCTGTAAGGATTGCAAGCTTTTGTAAATGGAAAAACAAGCCAAGAAACTGCGGTTAGCCTACTTAATTAATGTGGGTAATACTATAATACCAATGTGGATAATAAAAAATTGTCAGTAGTAGACATGTCTGAAATACTTTCTTGCAGTGCACTATGTATTCTGTTTATTGGCGTCCCACACTGTTAGCGGCGTAGCATGCTAACTTCAAACTCTCTTAGAATCCATACCAGGCCAGGGCAAGATTTGGCGTTAGTCAGTCTCTGTGGTTAACCAGCCCTGGTTAAGCTAGCTTAGCTACGCGCTCGAAACAAGATCTGAAACACCCACACTAAATTGACCATAACAATAATGTTTGTTTGCTAATATTCGCTCAGGAGTATTTTTTTTCTGTGAGGATTGCAAGCTATTTTAAATTCAAAGAAGTCAAG |
| 239 | lncHLBr_003 | CCTCAATGACACGTCAATATATGTACATTACCAAACAAACAAGAGGTTTATGTAAATAACGTGAACAATTTGGATACAGTGGGGAAACTGATCTATATTAAGTACTTAAACGCAAGTCAAACTGACTATTGGTCAAGGCGACAGCATCAAAATCACAAAAATGTACAATTTGCAAGGTAAGACACCAACTAATTTAGTGGAAAAATTCATGTATTTCTCTCCATTGTTACAAAAGCTACACAAGTGAGTTACAGAAAACTGAAAAGTGTTGCGAGTTAAAAGGTGTTGTTCTGCCAAAATTGAAATGATTACCTCATATCATACTAATTAAAATATTTAACAGTCACACATTAGAAAAATGTGCCTTGGTTTACATTTTTGAGAACCGAGGAATGTGTTCGCGGGCACATTTGGCTGAATTGTATGCTATGGGGCTGTACAGCTGACTGCTTACCTCCGTATGTAATGCTTTTCCAGTGACTGGTTTACTCAGTAGCTTACCGCGAATACTCCTAAAATTGGGAGGAGGAGCACAGCGAAGCAGACCAGGGTTCGAGGTCGATGAAGAACAATTCCAGAAATCAAGAAAAAAATAAA |
| 240 | lncHLBr_004 | GACTTTTAAAAACAAATTCATTTGTCTATCCTCCAAAAACAATCCAATCTTTGAACCAGTTGCTCATTCCATAGCGCCCATATGTATTTATTCATCTCTCTGCTGTGTTTTTTCCCCCTTTTACATTCACTGCCGAGGTCTTAAACTTAACACGTAATTACACAATGGTGAGCCCTGCCTGTGATAAGCTTTTGAATGAATGAGTAGCCGCTGAGACTAAATTGCTCTCGCTCTCCTGCTCCCTCCTGTCGCGTCTGGCCGTCAGGTCAGGGTTTGAGGCGAGTTGGCAGTGCGGAGAGGACCAGGCCTGGCCCCGTGCCTCAGTGCCTGCGGGACCCTACCCAGACTGCCACGGCCCCTCTGGCCTTCACAATGAATAGAGGTCTTCAGACACCAGTGCACTGAACTGAGGCAGCCTTTTCAGCAAACAATCCTATCTGCTTCCTCTCCACTCCAGTCCTCCTGCAACTTCACTCAACTCGTCCAGTTTGTCAGGGGGGCTTATTTTTGTTGTTTTATTTCCATGTGTTCGGGAGGAAAAAAAAAAAAAGAAGAAGAGCTAGAATGCTGGAAATGGCCCCGAAAAAAACACTGAG |
| 241 | lncHMBr_001 | GCCTAACAAAAATGAAGCGTTTAATAAGTAAATAAAGTGAATGCAACACTTCACAATGGAGTTTAAGGAGACATTTTTTAGGTTTTTGTAGAAGGAAACTAAGCTGCAGTATTGTGACTGACAGCTCTTGAGCCTGTGGCAGAACTGGAGGAAATGTTCTTGACAGATCAGCAAGCTGCAGCAGGGAAGGGGTTTGACCTCAAGCCCACTACTTGAAGAAAACGAAAGAAAGTGTCAGTACTTTCTGCTCCATATCAGTGGAGTGTTTCTGCTCATCTTCAAAGGCCTTTGTGATGAGTCCTGTCACCAAGACGATGCTTTATTTATCAAAGTAAACACATTCTCTAACGTTCTTTAAATTCTTATTGGATGGTTAAATCATGGCAAACATATGTCATATGTAAAAAGCAACATAGATGTAAAATATTTATACATTTAGTCATTTAGCAGATGCATTTTTCAAAGCGACTTACAATTAAGGAGGAATTCCTATTCAGTGTTTTTTGTATGGTCTGATAAACAATATCAACATTATGTACACTGACCTACTACTTAATGTGATAGTTTAACCAAAATTAAACATTTAAGCAGTACAACATTGAAGAAGATTTTTAGCTGAAATTGTGGTTCCAGGTTATTCATAAAATGTAAGTCAATAGTTACAGGCACTTTGTGAGTTCATACAGGTAAGCATACAGGTAAAACAAAATTAATAATCATGGCTACTGATGGTTCATTGAGACGTCTGTAATTTCAACAATTTACACGCATTAGAAAGCTTACACATTCTATAAAGTCATGACACACATTAAAGGAGTGGTCCCCAACCATACCCTATTTTAAACTTAAGTTAAAG |
| 242 | lncHMBr_002 | TTCGACCCTTTGTGGTATTGCTCAGCCGACACGTCAAACAGACATGGGTGTTCCTACCAAATTTAAATAGTTTAACCCAACTACTGACATCCAAACACACACACAAAAAAAGCTGGGTTGTTCTAACTCAGTGCTGGGTCAGATATAGACAAAAAACAGAAATGCGCGCACATCTAGAAAATCTTGAAGGCAGATGCTCGATCGAAAAACAAACATTTGTAGCAAAAGATGCACAGAAAATCGAC |
| 243 | lncHMBr_003 | GCGGGATGTCTCCCACAAAGATGAACACTTGGGACAAAAGCACCAACTCAGCCAACCGCATAGATCTGTCAGCAGTATAATACATTCAGATCCAAAAGAGCGCAGGAGGACAACAGCACAGCGCTTCCTGTAGAAAAACACGTGCCTTGCAGTATTGCATCGTAAACAGACTCCAAATGATGAAATAGTTACTTGCAGCTAATTGTAGAATAGTGCACTCACAACACAAAGTTCTCTGATGTCATTTCCTCCTGCTGATGATCTCTTTATTGTGAACTGGTTGAAGACACAAGGCCCGTTG |
| 244 | lncHMBr_004 | AGGGGGCAAATAATTCTGACTTCAACTGTATATTTATGTAGGAATATACTAGGAGTCATGTGCAAAATGAGTGAGACTCTCTCAAAACAGACAACCAACCCAACAGCAGACTTGACAACGCTCTGTTAACATGTTAAACAGCTTGTGGTACAGGTCATAACTCCTGCTTTCTCCATCTAAATGAACAGAATGTGGAAATATAAATGTA |
| 245 | lncHMBr_006 | TCCACCAGGTTTTAATTACAACCAGTATACAAAAATAATGTGACAGGACAGAAAAAGCCAAACCAAACTGGGAGTTGGAATGTACACGCTTTTGTTTCCGCTCCAAATAAAACCCAACACACACGTTTAGAGGAGTTGTTCAAACAATTCAACGTTAATTCTCTCACCCTCAGGCCATGAAAGATGTATGTGACTTTTATTTCTTTAGCAAAACATTGAAGATGATTTTTAGCTGACTTTTAGTTGGCTTATCATAAAAAACAAGTCAATTGTAACCAAAGGAAGAGAAAATGGGGCAATGCAAAATTAATACTTGTGTGCTCCTGAT |
| 246 | lncHMBr_007 | CTTGGTTTGATGTTTTGTTGTTTAATGCAGTATTCAGATATTTACAAAGACCCGTTCAAACCAAGAACCATACGATATCAATAAGTAAGTAGTTCTTAATATTTAGAAATAGCATGAACAATGTAATATTGTTGCACAAAATGAAAATTGTCAGCCAATCAGAGTGCACTTGAAGACTCTTGCGTTTATGACAGGCAAAATAACTTGATTGTGCACTTTTGTTAAGCAAAATGTAATCATGCACTTATGCGAATCCTCTTAATAATACTGGGTTGTCTTATCAGTCTAAGAAAGGCTGGGTTATTTTAACCCAAACTAAATAACATGTGGACTAGCCTAAACATTGGGTTAAATTAGGTCCTATTAATTTATTTTCTTTAAATGAACCCAGCAGTTGGGTTAGTCCCTATTATAGCCAGAATTGCATAAAAAATGCATTTTGTAACCCAGTTAAATTTGTCAATTTTTTTACTTTTTTAAATCTTTTTTTCATCATGGTTATAGTTTTTGGTGTGAATGGGTCTTAAAAGGACAGTTTACCCAAATTAACATTTCTT |
| 247 | lncHMBr_008 | TTTTTTGCAGTGTAGCTCTTGGATGGAGATAACAGCACTCACACTGAGAGATACAGAGAGTAATATGAAACCTGAAGTCTCGAAATGCACTGACTCAATGAAATAAATTAAGTTTATAGTTCGGTTTATGTCTTTTCAAGGTAAATAAGCACAGAAGATTGACATTGTGCACTATTGAAGACGCACTGTGATACTTTTTTCTTCAGTATTGTTTAGCACCTTCTCTTTAATGGCATTTTTTTAAGGACCCACACTGCAAACATGATTTTCTTACTTAGATTTTGTCTTGTTTCTAGTCCAAATACCTAAAAATTCTTTCATAAAGAAGCACTTTCAAG |
| 248 | lncHMBr_009 | TGTGTTTTTATTTGAATTATTATCTGCAGTGCCGTTATTAGCATTAATATTAGCATCAGGTGAACATCAAGTGAGCATGCATTATTTACAGTAAGCCTCCGTCATCTACTGTATATGCAAATATTACATTATACACACATATTCACAGCAGGATGATCCTCCTGAAATACAGTCAGAAATTCACTGGGATGTAAATTATGATCGATTATTGATGATGGTGATGGGAAAAAAGTGAAGATATGGATTAATTGGAAGAGTTAAACTTTATTTTAGTTTATTGTCTGTGTTTGGTGAATTTATAGACATATTATGAACTCTTTGGCTTGTCATTATCTTTTC |
| 249 | lncHMBr_010 | CTTTCCTTTAATTTCTAAAAGTAGTTACTGATGGAACAAACGCAGAAACAGTAGCAGGTATGAGCATTTCTACAGAGAGAGAATAAGTTGTGCAGTGAAATCAGTGTAAGCGCTGAAGGCTTTTACTGTGCGTTATTCATATGTAACATTCAGTGCAAACCCAAATCAACGAAATACTGAAGGACAGTGACAGCAAAACATATAGAAAAATATCATATCTGTCACTTTAAACATTAAACCTGGGAGTTCTTTAATGTGTAACACCGTCCAGAGCTCTGGACTAACACTGAAACAATAATAATAATGTTATTAATAATAATAGGATGAATAACTCATCACCATTAACAATAACAGGAGGCAGAAAGCAGCTCAAAAATAAATAAAATCTCTTTCATGTTCGGTGATTCTGTCTTGTTTTCACAGTACAAA |
| 250 | lncHMBr_011 | CGTCATTTTACTTTTGCTGTTGGAAAAAAACTATAAAATAATGTTTTTTTTTTACCATGCATATTTACTGCCCTTCAAGCATAACTTAGCAAATGTATAGGAAACCTCCTGATAAGAACAAATGAAAGCAGCAAAAACACTAGATCAAAAAGTGCCATGGGTTTATACAGTCCAGTATATTCATAATGAATATTAATTTATACTTGCTTTGAGGAGGCCATGTAGCTTTTTTTTAACTGTACATTTGTGTTTTAAACCAATTTTGTAAATATTTAAGTTACGGTTGTTGTTTTAGGGTCCACAGGGTCTAAAGTCTTAATGTCTTAAATCTCAAAAGAAAAATTTTAGGCCTTAAATCTACTGAAATATGGTGCTGTATGTCTTAAATCTTTTGTTAACAGGTCTTAATTTTCCTTTGTTCATGTATAGCTACACAATCTATTAACACCGATACAATCATCAACAATCC |
| 251 | lncHMBr_012 | GCGTTCTACAGGTTTGTGGTGATGCTGCAGTTTTGATTTGGTCATTTTTTGTGGTGTTTTATTTCTGCTGCCTCTAAAATGTTGTGTTTAGATGAATAAAAATGACTGTGAAGCGCACTAGGTTGTGTTTGAATAGCAATCTAAGCATGGCATGTCTTGGTTTGTGCTTCTGGGCTTGATTTCTTGCTCAAATAAATCTGGGTGTCCT |
| 252 | lncHMBr_013 | AAGAATTCAAATCATCCAAACATGATGTGAAAACGGAATTAGGGCCCGTTTTGGTACGGTACGCTACAGTTCGGTATGCTTTTATGTCAGTTTCCACTGTCAAAGGAACATCTGTAACTTTCTGACAATCTGGTTATGGTATATGATTTATTTATTCCCTGATTGTTTTCAATATTGTGTTGTGTATTGTCTTATCTGCTTGTGCTAGATTCTAGATTCTCATTGTCCTAACTGTGTGCGTTGTTGTTACATTCACCAACCTGCCTTAACGTATTGAGTTATCAGATGTGTGGTTTTGTTAAACGTGTGGAGTAAAAACCTTTATTTTGTGCTTTATGACATTATTATTGTATTAATATTGTATATATGAATGTAGTTTTGGATGAAGACAAAACTAATTTGATTGCTTATTGAAAGACCACGTTATTTATAGCAGTATTTCTCAACCATGTTCCTGTAGGACCACCAGCTTTGCACATTTTACTTGTCTCCACATTTTACTTGTCTTACTTGTAACCAAACACACCTGATTCTGATCAACAACTCATTAGCAGAAACTGAAAGACCTGTAATGGGTTTGACAGATATCTAAAACATGCAGTGTTGGTGCTCCTCCAGGAATGTGGTTGAGAAACACTGATTTACAGAATCATGGTGCAGCCCATGAATACTATTTAATTAAGT |
| 253 | lncHMBr_014 | CTTTGAAAGAGACATTTTAATGACAAATACACTGAAAACCTGATCGGAAAAAATATTCACGACACATTTATAATGCCTCATGACAATCATGTTTATGACAGATTTATGACAAGACAAACTTTCTGTCAAGACAAAGACAAAAACAGGTTGTGATGTGTTTGTTTATGCCACTTAATAACACTCAATAAAATCTCATTCACATGTGTCATGTCATGATTATAATGGATTCGCGACAGTCTTATGAACAAGTGTAACCAAAAATCATAGTTATATTGTTTGAAACAACTTGAAAGTGAGTAAATAATGTTCAATTTTGCGTGAAGTATTCTTTTAAGTCATTATTTGTTTGTTTGTGAGGAAAAGTGTGCGTCTCGATCTCTCTGTAGATGCACACTGAAGCCTTAACATATGCTTTGTTTTGCTGCAAATCAGGGGT |
| 254 | lncHMBr_015 | CGGCACTATACAGGAATTATTGAAATATTTGCATATCCTAAAATGATAGAAATTATCATTAAAACAAATGTACACGGATTTACATAGGGTGGGCTTGCTTATTAAACATCTACCAGTCCAAATAAACCTCACCAAGCAAGAACAAACACTTACCTGAAGAATTTCAAAGCTAGTGAATCTGACTTCAAGACGGTGTTCATTGCAAGCTCCAGACCTACACAAGTGCAACTGCTAGACACCAAGCAGATTATACTTTCACAGAAGGAACAGAAATATGCA |
| 255 | lncHMBr_016 | ATTTATTCGTTTTTTATATGCTCAGCCATTGTTGGTAAGCCTCATAAATAAAGACATTTGAAGGAGGAGGGATGCATTGTATCACTTTCAATGGGAAACTAAAGTAAGATTCGCTGTTTTATGGTCTTGAGTTACTGTAACAGAGCTCACATCAGTCATAAACAACACATTTCTGGATTATACATTGTTGTTGCTCTGATCATTGAGCTTTAAACATGAGAATTTTGCAAACACAAAAGTT |
| 256 | lncHMBr_017 | CTTATTAATGTAAAATAAACCACCATCGATATATGGGTCAAGTCCACACAACAAGCTTAAAAATGCTTGTAATAAGCAGACGTGGCAACACTTGGAGAAAAGTGAAGTCCTTCTAAGGACTAACAAAACGTGACACCTTCTTTCAACTGAGGTGTAGGTCAAACTGCAGACAATACAGAGTGTGCAGTGCTGCAGAGTCTGTATATGCCTGAATGTTACTATGGCTACAAAAAAAAAAAAAAAGATTGGTATGAT |
| 257 | lncHMBr_018 | GTTTGGTTTGCAGTAAATCCCTGATGATACACACCATCTTCATCCGGATCAGATGTAAAGTGTGTGTTTCTGAGGCCTTGCTAATATCACAGCTGCTTTAGTTTAGACAGACAGATATGCTGGACGAGGACAGCCAGAGCCTAAATGTTGTGCCTTGATATGGTGAAAATGCAGATAGAGACGGATGAGGCACAGAATGTGCCAGTCATTTTGTTGATATGAGTAGACCTTTAATTAGGCGGCCTTAAAATATTCTCCATGGTTGAGATTATTACAGACATACACGGCCTGGCAGACATTATTAAAGAAATTTCTCTGAACAACACATTCAGC |
| 258 | lncHMBr_020 | AAAATGTTGATTTTTGGAAACCGCTAAGCGACCCCCCCTCATTGTCCCGCGACCCGCCTAGGAGTCCCGACCCCCACTTTGAGAACCACTGGTGTTAATGACCTCTAAACCAACAGCACAAATTTCAGCACATTCAGTGCTCAGCTTACAACAAACGTTCACCCAATATTTGTTGTTGTTGTAATGGTTTTGTATAATTGCCACATCTTCCTTTGAGTTCCACTTGCTAATTGAATCCTGTTAACTGAATCCAGCTAAATTGTCTCTGAGTAGTATTTCAGAGAGAGGGTGAATGATATGGTAGTTGTAATTTGAGCATGGAAGCAGATTCAGCGAGTTGAATGTTCATGTTGGTTTAGTATTTAATCACTTGTGCTTTTTTGCACTGTAGGGACTGTTTTGAGATATTTGCTAAATGCAAGGCACACCATGTTTTGTCTCTTTACGTTCAGTTAATAATGAGAAATAATGTAAATTATAGATATATCTGAACTGTTT |
| 259 | lncHMBr_021 | AAAAAAAACTGTAAGTTTGCACCACTTTGTTTATTCACGCAAAAAAAAGGTTATTTCAGTGTGTTAGATGCTAGAAATGTGTTATTCTGGTTGATTCTGTTTTCTGTTTCATGGTTTGTTTTGTGTTGTTTCATGTACCACGTTCACACTGCAGGTAAATACGCTTTTGTCTAGCAGTTCTGTTGTTTTCGGAAGTAACAAAAGCGATTTAAAACCAAATTTACGAATGAAACGTTAGCTGTTTCTCAATATACGTTCTTCAGCGATGTAACCTCTGACATCATCATCAACTGCCAAAGCTCAGTTCCAATACTTAAGACCCCAAGAACGGAGTACGAGTGAAACTTCTCGGATTTGTTCTTAATATCGAGGACGCACCGATGCAAACTTGAGCACTGAACTCGCTCTAGAAGTCCCAGAAGTCATTGCAACCAGTGTTGCCAACTATTTTCAATGAAAAGTAGCTAAAGCCTGCCAGAAAAGCAGCTAAATGTTGTCAGATGACATCACGCACTAATTTGCATATCAGTGACATCATCACGTAGTATCTGACAAGCGTTAGCCAGTCATGTCTCTACTTTCTGAGGCGCTGTGTATTATATTGTACT |
| 260 | lncHMBr_022 | GTTTAGAGCACCTAAAGTATAAAACAATAATTTTCAACATTTTAAATAGCTGTATTAAGTTTTATTGCACATCAGTAGGTTCCCCCATGGCGATTCTATTTATAAGATATTTAAGAAAAGTCCTATATTCTGAAATAAATTTAGAGGATGCATAGCTAGCAATATGGTTTTGCTTACTATATGAGTGTTTGAGGTGAGGTGATTATCACTGTCACATGACAAAAATTAATATAATTAGTATTGTCATCATTTGAAATGCAGTAAATGGTTAAACGTTAAAGCGGCGGACATCCTACAGTTTGACAAATATTGCTGCTATTGAACATTATAATGTACTTTTTAGGTTTTTAAGTGG |
| 261 | lncHMBr_023 | GTCAAAACAGCTCGTGACAGAGCAACACATGCTGAAAGTGTCCGTGACTCTGGTCAAGCCACCAAAACATGCTAAAAACCACCTGAGTTGTCGCTTTCGTAATTATATCCCATGGTAAACTGAGGTAAAACACTGTTATCCGTATTGAACAAGTGTTGAATTATTATCCGGAGGTCTTCGTTAATTCGTCCTCGTGCGTTAAAACCGATTATAATCCCTTTCGGTAAAATCCTCTCTCTATTGTTGGTGTTTCGTCCACTGTTGACTCCTTTACTGTCCCATGTGTGACTGTGTGTGTGTGTGTGTGTGTGTGTGTGTGTG |
| 262 | lncHMBr_024 | GTAATGTTGGGTGTTATCGTACAGACACTTCAACACTCGCTGTTTCTAGAAGATCTAGCAGGGCATCTGGGCAGCTTCAGTGTACATTAGTCTGCCGCACACTGATTATATGTATCAACACTAGTTAGGGTGGATAGTGTGTGAATTGAGGCGCAGCCGGAGTGTCTGAAGTGTGCATCCAAAGTACACAAATAGCAGCAAGTGTGTCCTCACTTCTGTGTGTGTGTGTGTGTGTGTGTGTGTGTGTGTGTGTGGTGTTCTGCTGCTTCTGAAATGTGTGTGTGTGTGTGTGTGTGTGTGTGTGTGTGTGTGTGTGTGTGTGTGTGTGTGTGTGTGTGTTTGAG |
| 263 | lncHMBr_025 | CAGCAATCTATTTTGTTTACATTTGTTTTTATATCTATGTAAAATTTATTTTGATCATGCTCACACCGCAATAACAATGTGAGAGCAAGTCTTTGGCCAAGGGCAGAAAATAAGTAAAGGCAACTAACCTATATTCAAGTTAGGGCTGCACAATATATCATTTCAGCATCGATATCGCAATGTGTGCATGCCCAATAGTCACATCGCAGGATATGCAATGTCAGGATTATAGTTTAATATAGTTTAATCATAATTAAGTAAAAGTTTATCATCTGCATGCATTTTTAAAGAGATAGTTCACTCAAATATGACTTACCATTTTCATTACACCCTTCACTTGTTTCAGTTATTATGAGTTTTCTTTTTTTATTTCAACAAAGACAAAAATTATATAATTT |
| 264 | lncHMBr_026 | AGAAACTCTACTGAAACTCTCTTTAGTGCATTCGAGTTCACACTTGATGGAGCGACTGCCTCTCAGACACACATTTTATCATCTCTGCCCAGTGTGCCATGGGGGCTTTGTGTAGTTCTGACAGACTGTATGCCAAAGTGTGAGCCAAGCCAAGGCTAGCAGATGCTAACTGGATGAGCTGAACATAACTGAAATCCAGACCCCCTGTATGAGTGTCTCTCTCCTTTCTCTTGCCCTCTCTGTTTTTCTAATTATCCAAGTTCCTCTTGTTAAAAAAAAAAAAACTGTGGTCACACCATGACACAGTAATGGTGTTG |
| 265 | lncHMBr_027 | CTGTAATGTTATAAGAAACATTATAAATTGAACACATCCTTACTCAACAGGTTCGCTTATAAAAAGAAATCATATGTAATCCACACTTTTTAACAGTAGTGCACAATTTATTGGCGGTTAGTTTTTTTTGCATGAAACTGTAAATAAAGCTGCATGACCACACTTGTACACAAGTTTCTATCTATTACAGATGTGTTTAACTGGATAAAACCCAGATCACTGACTTTCAGTTACTGGTGTTAAGTGATATGATTGTTGAGTAATGTTTTCCATTCTCATTTTACAGTTCCACTGAAGTGAAGTAAGTAAAACAGCGTGAGATGCTATCAAGTCAAATAAGCCTGTGTAGGCGTACAAATTAAACCTGAAATATGTAATCAAATTTGAACACACAGAAAACTAAAAGTCAAAACTAGTCACATCAC |
| 266 | lncHMBr_028 | CTTTGAGTTGCCTTTTTGCATAAAAATTAGGACAAAATGTGCATAGAGATTGACATTTTACAGTCAATTTAACATTAAACATTATACAATGCTCAACAGATCATTAAAACAACATTCAGATGACCCGAAACATACCTTTCAACATGCATGATGTTAAACATTGCATTATTTTACAACGTTCATATATTGAAAGCATCTAATTGGTTAAAAAGTTTACTTTCGGTATCTTGGAGTTGACTTTTTGCATAACAATTAGTGCAAAATGTGCATAAAGATTTACATTTTACAGTCAATTTAACATCAAACCTTATACAACGCTCAACAGATCATTAAAACAACATTAAGATGAC |
| 267 | lncHMBr_029 | AACTACACTAACACACACTACAGTAGCGTGCACACACATGCTGTAAACTACACTAACACACACTACACTAACACACACTGTAGTAACATGCGCACACACATGCTGTGAACTACACTAACACACACTACAGTAATATATGCACACACATACTGTGAACTACACTAACACACACTACAGTTACATGCGCACACACATGCTGTAAACTACACTAACACACACTACAGTAACATGCGCACACACATACTGTGAACTACACTAACACACACTACAGTAAAATGCACACACACACATGCTGTAAACTACACAAATACACACTAGAGTAACATGCGCACACACATACTGTGAACTACACTAACACACACTACAGTAACATGCACACACACATGCTGTAAACTACACTAACACACACTACGGTAACATATGCACACACATACTGTGAACTACACTAATACACACTACATTTACATGCGCACACACTACACTAATGGACAACACCCACTGCACTAACAACACTCAATCAAACACACACACACACACTACACTAATGCACCGTTACACACTACACTGACACACACTACAGTAACATGCGCACACACCCTTAAGACTACATTTACACACACTACACTAATGTACAATACCCAATGCAGTAACAACACTCAATACAACAAA |
| 268 | lncHMBr_030 | TTCAGAGACACTGCTGATCCTCCCACAGCAAAAACAGGATGGTAAACTAAAGATTTCAGCCCTCTCTGGCATCTGGATTATATTTGAGTGTGTGGTTTCAGTGAGTCATGCGGAAACACTGCGCTTTCACCTCTCTCACACTCGCTGTATGAGACACCAACTCAACATCATCTCTACTGTTAGCTAGACGACACACAGCACAGTGACGTTACAGTGAATGTGAAGATGCTCCAGTTCCTGTCGTTAGATGTGATCAGGTCTCATTACACCACTGGAGCATTGGAGATTCTGCTCAAATTACCTGTCAAGTCATAAGTTGTGAAAAATGGA |
| 269 | lncHMBr_031 | TTTTACGCTTCAGGAACAAGACATGGCTGATTACTCCGATGTTATCGTACACTTCAAGGACAAGCCAAGGCTGATCACACCCGTGTGATTGTATGCTTTAGGGACAAGCCAAGGCTGATCACACTGGTGTGATCGTACGCTTTAGGAACAAGCCAAGGCTGATCACAACGGTGTGATCATGTGCTTCAGGGTCAAGCCAAGGCTGATTACACCGGTGTGATTGTTTGCTTTAGCGACAATACAAGGCTGATCACACTGGTGTGATCGTACGCTTTAGGAACAAGCCAAGGCTGATCACAATGGTGTGATCATGTGCTTCAGGGTCAAGCCAAGGCTGATTACACCCGTGTGATTGTATGCTTTAGGGACAAGCCATGGCTGATCACTCTTGTGTGATCGTATGCTTTAGGAACAAGCTAAGGCTGATCACAACGGTGTGATCATGTGCTTCAGGGTCAAGCCAAGGCTGATTACACTGGTGTGATTGTTTGCTTTAGCGACAAGCCAAGGCTGATTACACCAGTGTGATCATGTCATAACTGTATGCTTCAGGGATCAGCCTAGGTTGATCACACCAGTGTGATTGTGCCATGATCGTACACTTCAGGGATCAGTCTAGCCTAATCACATTGGTGTGATTAGCCTTGGCTTGTCACCAAAGCATACAATCACAGCTGTGATTGTGTCGTGATCGTACACTTCAGGGACCAGTCTAGCCTGATCACACTGGGAATTAAAGCCTACAATCACGGTACAATCACACCGGTGTGATCAGCCTTGGCTTGTTTCTGAGGTGTACGATCACACCAGTGTGATCAGCCTAGACTGGTCACTGAAGCGTACGATCACATCGGTGTGATCAGCCTTGGCTTGTCCCTAAAGC |
| 270 | lncHMBr_032 | CAAACCTGGAATCGTAAGAACTGGGTTACATCATAATTGCATTAAATAAACGTATAATCGTGGGATTCAAAATTGCAATAAATAAACAGAATTACGAGATTCGGTCAGAACTGTGTCGAATGAAGAAAGACAAATAATTACAAAAAACCTTTTGAATTTAGAGACAAAGAGTTGTAAAGAACTCAGGTTTGCTTGAAATAAACTCAGAGTTGCGCAAGATAAGCTCGCAATTGCAAAAAACTTAGGATTTTGCAAGAAAAGATCAGAATTAAAATCAACTCTAGATTACGCATGAGATGAACTTAAAATTTGTGCAAGATTAGCTCAGAATTACAATAAACTCAGGACTGTGCCAGTTCAGAATTTAGGATTGTGTAAAAGATAAATTTAGAACTGCGCATTAGATAAACTCAGCTCAGAATTACAATAAACTCAGGACTGCACAAGATATAAACTTAGAACAGCACAAGATAAGCTCAGAATTTTAGTGGACTCAGAATTGCGCAAGATAAACTCGAAATTGCACAAGCTAACCTTAGAATTACAATAAACTCAGAATTGAGCTAGATAAACTCAGAATTAGAATAAACTCAGGATTGCACAAGATAACTTCAGAATTACAATAAACTCAGGATTGCGCAAGATAAACTCAGAATTACAATAAACTCAGGATTGTGCAAGATGACCTAAGAGTTACAATAAACTCAAG |
| 271 | lncHMBr_033 | AAGAGTTCTCGTATTCAAAATAAGCCACCGCACACCAAAGCCAGGCAAAACAGCGTGGCCATGAAGGAACGTTTACCTTGCACAGTCGGTGCCTTTATCAGTATTACAACCTTATTATTAGTACCGTTTTCGCTCCTTTGATATCTGAAACTGCTTTGATCTGAAGTGTGTTACTTATTTGTTCTTTTTAGGCACCTTACTTTTTTGTGCAGTTCACAAATGTGAGTGGAGAACGTCGTTTTTAACCATCCAGTGTTTCTCTGAGCTCTTCAGAAAAAGCACACACACAAAAG |
| 272 | lncHMBr_034 | TAAATACAAAATCTTTATTGCAAACTGTTATCAAAATTTCCTTTTATTCGTAATTATTTATTCTGTGGCAGAAACAATCCTCCATTCAGCTTTTGCTTTGCATACATTTTTTTTTTATCTCTCCGCTTGCGCTCAGCATTGCTCTCAATCCCACGATCCACCACTCGGCTCCTATAGAGAATGAACTGGAGTGGGATGATCCGCAAACAGCGCAGAAGAGTTTAGTGCCGATATCAGATCACAGCAGCTTAGTTCGGTTCCTGTACACACAAACACACACAGTCTTTCCTCACTCGTGTAAGTGTGAGGGTTCTTTGAGGCATGAGCTTTATTCAGTGATCGTAAACGCCGCCCCAGATGGATTATAGGAAGCGTTTCACACCCAGAGGGGAGGTTTCTGGGCGCTCCTGAGGGATTGTGGGTAACAGAGGAACGAACTGATGAGCTTATGGCTTCCTAACACCTTTTATTCTCCTCTTTTCTCCTGATTTCCGCTCTGCACCTTTTGGTTCCCCATGGAAATTATCGGTTATCTTGGAGTAGGATCACATTTAATAGATCGGGAATAAATAATCAGAATCATTCCAAACCTGTATGATTAGTTTTCTTTCTGTTAAAAATGCTTTAAAGAACTTTTACAAATGACAAAAAC |
| 273 | lncHMBr_036 | TTTGAAACTAGCCTATAGCGCCATCTGGTGCTCAAAACTAGCCTAGGGCACCATCTGCGGATCAAAACTAGCCTAGAGCACCATCTGCTAGTTAAAACTAGTCTAGAGCGCTGTATTCTGTTCAAAACCAGCCTAGAGCTCCATCTGCTGTGTAAAACTAGCCTAGAGCACCATCTGCTGTTTAAAACCAGCCTAGAGCAACGTTTGTTTACTAGTGTAAAGCGCCATCAGCTGTTCAAAATTAGGCTAGAGTGCCATCTGGTGTTCAAAGTTAGCTTAGAGCAGCATGTGGTGTTTAAAACCAGCCTAGAGTGCCTTTTTCTGTTCAAAATTAGCCTAGAGTGCCATCTGGTGTTCAAAACTAGCCTAGCGCGCCATTGGCTGTTTAAAGTTAGCTTAAAGCGCCATCTTGTGTTTAAAACCAGCCTAGAGTGCCATCTTCCGTTTAAAATTAGCCTAGAGCACCATTTGGTGTTTAAAACTAGCCTAAAGCGCCATCGGCTGTTTTAAATTAGCAGACAGTGCCATCTGGTGTTATAACAAGCCTAGAGCAACGTTGGTTGTTCAGAACTAACCTAGAGCGCCATCTGCTGTTTAAAACAAGCCTAGAGGTCCTTCTGCTGTTTAAAACTAGTCTAGAACCCAGTCTGCTGTTAAATACTAGAGCGCCATCTCCTTATAAACATTCCCTAGAGCGCCC |
| 274 | lncHMBr_037 | CGCCTCTCAGTTAATTCAGTATCTGAAGAGACGTTCGCTCATCTGTCTAAGCTGCGGGTGCTAGACATTGGTCACGGAAACATTTCTCCCAACCGAGGCCAAGCCGGAGACAATATGGAGGAAGAAGAGGAGGAATTAACACAAGAGGAAGAGGAGACACAGCCTTGAGTCAGAAACACGTGAAAAACAGTAAATAAATAAACAAGTAATGCCCTGAATCTCCCTGCACTCTCAAAGAAATTAAAACATTTGTATTGGTAAATGCACCGTGGCTGCTGACGAGATTATTTCCATTGACTGATTACATGCTAGCAAAATGGTAAAGCTAATTATTTACGTATAAAAGAAACATGCTAGCTAGCTAAACGCTAATTCATGTTAACAACAATATGCTAAGCATGCAACCAACACAACGACAAGCTGATTCATGCTAGCATCTTAAAACATGGTAAACATGAAAGCATAGACGTGTTAGAATTGTGATAGCAATGCTAACGTTAAAATCTGCTAACAACATATTGATGTTAAATGATAGCAGTGATGTGTTTTATTATAGTAGGATTGATTAATACAGTGTTGGAACATGCTATTTCTAT |
| 275 | lncHMBr_039 | TTAGTATTTTGGAGGCCAGTGTTGAACAATTACTCCGCACATCCTTACTTTCTTCTTTCTCTCAGCATGTAATCCATTACTTTATTGCACAGCTCTTTCTGTGGATTCTTCCTTTTTCTGTGTCACATTTTTGTTCCTCACATTCTCACTCTTGCTTGCTTCTTTCTGAAGTGCTGTAGGAAAAGGTTATTTCCAGGGTTGGTTTGTTTCAGATAAGGATCTCCACAAGATCTAGATCAAGCAAGGGCATCTTCTGTCCTGCAGTCAGCTGTGCTCGACCCTCCAGGCCTTTAGATTGGCTGGATG |
| 276 | lncHMBr_040 | ACCCTTTACTGAGAGAGAAACAGTAGATGATTGTATTGAGACCCATCCTAAAACTCCTTTACACCAGTACTGACCCTGATCAGACTCAGCAGCTCCTCTGATAGTGAGAGTGTCTCTGTTTACAGTGTAATGACCATCAGACTGTAACGTTACACAGTTGCTGTCTTTATACCACTCATATGTCCAGTCACTGTGATCAGACTCAATCACACATTTCAGATTGACTGTCTCTCCAGTGAATACAGCACTGTCTGGTGTCACAGTCAATGCTGGTCTTGG |
| 277 | lncHMBr_041 | CGACTCCTGAGTCTGGGTCTGTCAATGTCCATTTATCTATTGCTGACCTAGAAGGTGAGATTGTGAAGGGTTTAAATCTCTGTTTACTGCAAAAGTTCAAAAGTCAGACCAAGCTTAACCTCTGCCGACCTTTTCCACTCTTCTCTGTAGTTATAGGCCTTGCCAATTAGTGACTTTAAAAAAATCTCTGTTCTGATCCACACAGCTGCTAGGAGAGCACATTTTTATATCTGCA |
| 278 | lncLMBr_001 | CTAAATTATAGCATGTCACCCTAAAGGCGTGATTTACCGAATCATTCAATTGGCTCATTCAAAAGGGCTGATTCATTTCAAAACCATTTTGTTTTATTTCTCAGTAAACCAGATTTTATACTAGTTATGTATAAAGACTTGTGTTTTTAAATGAATCAATCATTCTGAATGAATTTATGGAATGAATTACTCACTGAATCTCCCATTAACCCTTTATCAGGCAATGGATAATTCATGCACAGTGTTGCTCTGAGATGCCCCAATTAGAATTTTTTTATTTTATTATTAATCAAAAAATTGTCTGTGCAGATGGCCTAGTGGTTGGTGCGTCGAC |
| 279 | lncLMBr_002 | CAGGACAGAGAGTTCTTGTAGACATCTGAATAAAAGGCAGTTAGGGAAATTTACAGATTAATCGGGCTGTTGTTCAAATCAGAGCTGTTGTAGGATAAATCCGAGTGATTTTCTCTCTTGTGTGGTCTTGGATCGCTCATTCTGCGAGCGGCTCACTCGTTCTTTCACACTGGCCTGCTTCCAAAAACAATCAGCTTGGGAAATTACCTCCAAAAATCATGTCGGCTTAAACAAATAGCTCTCTGAGTGATGGCTCGTAACTGTTTGTATTCCAACCACTCCCGATGCTAATTATGTTGGTTAAAATGAAGGATGAGTTTAATTTGTAAGAGTCCTGAGGCACAAGTCCTTATGTAAGGACATTGTTGAACACATTGAGATTAGTGTTAGTAAAGCACAAGACAAGGCAAAAGGGCCCTTCGGTGTTACCTCATTTGGCTTA |
| 280 | lncLMBr_003 | CCCACGTCACGAATCTGCAGAGATGCTGAAGGAGCCGCGATCATAAAACGGACGTTTACCAAAACACGGCGCCGTTTGTGCTTGTGTCAGAGCAAAGGATAAACAAATGGATGAACGTTCTGTGTTTTTACACCATTTAAGGTCTTCGACATGATTGTAAATCGCATTTTGTTAGGGTTTTAAACCACACCCGGCGCTGTGAGGATCGGGACTGAGTGGGCACCACAGAGCTCCGATGCCAGTCCCCGGTTTCCTTCGCTGGTGTTAGGAGACAAGCTCACAGCAGAACCACAGACAGAATGAGCGGAGGATTGGAGCAGATAGACATCCTGTCCACGGGCATTCTGGTGAAGGAACGATGGAAAATA |
| 281 | lncLMBr_004 | CCACGGTTTAATAACACAACTATTAAAATCTAAACTAAGTTACGATTTTGGTTCAATAAATTAAGCTACGTCAAACAAGAAACATTAATTGGAAGCACCATTACACACACACACACACACACACACACACACACACACACACACATATTAGAAAGGAACACTAAAATGTGCTCTAGCTTGACATTCAACACATTACTCACAGTGTAAAAGCTCACGACCACAAAAACAAGTTACGGCAGTCATCTTCAAGCATATCCGTCCTCACAGTTCCTCAAGTAGCTGCGATACAAACGTGTCATCAGTGAGTACGTTTACATGGACATCAGATATCATTATTCACCTTATTCTGAATAAGGCAAGATTATTATTGAGGTTTTACATGATGTTAACACTTTGATGTTCCAGTTTTACATGTTATGGATTGATTAATGGCATATCACAGTTGGTGTTGTTAAGACGTTTAAGTTTGTCATGAATATAGCATTTAATTCTGACATGGTCTTAAACAATAAATAAAAAGTTTACATGTCGTCATACATTTTTTTTATTTTCA |
| 282 | lncLMBr_005 | CACAAACACAACACACACTTACACACACGGGTTCACTGTGGGTGGAGAGACACGCGTCCAGCTCAGACATGGACCTGACGGACTCCACTGCCGTTTAATAGCCGCTGTTTGATCGCTCGGAGTGTTTATAATCTCTGACTGACAGACAGAAGAGCAGGCGAAGTGTTTGACGAATCATGAGCGCGCGCGTCCGCTGTTTTTTAGCCTCTTCACGTGATGCTCATCTTTTAGTTTACTAATCATCGTCCCGCAAAACATTACGATGAAGGACACTGGAGAGTCAAAGGACCAGC |
| 283 | lncHLM_001 | ATTTTATTTTCAAAATCCTTTCATTAATACTGTATAAAATTCAAAGAATGGCATTTATTAGAAAGATACTGCTTTTTGTAACATTATTAATTGCTTTTAGCAATCTCATTCATCCTGGCTGAATAAAAGTATTACTTAAATAATATATTCTTTTATTACTGACCCCAGAACTTTTCAAGCACCACAAACAGCATATTGGAGTGATTTCTGAATGACCAGACTCAATAATGAC |
| 284 | lncHLBrBl_001 | ATTAAACTTCACGCACCTCTCAGCTGCTGTAGATTAGGAGTGTATGTGGATCACAGAGCAGGAACTCTGTCTTTCTACAGCGTGTCTGACACATCAATGAGCCTCATCCACACCGAACAGACCACATTCACTCAGCCGCTCTATCCTGGGTTTTTACTTGCTTTCGGATCGACTATAAAGCTGCTGTGAAAAGAAGAGTACAAAA |
| 285 | lncHLBrBl_002 | TTTAAGTTCATCATTTAAGTTGTTGTGGTGTAGCCTGTATGTTGCCTTTTGACTTTTAAAGTTAGCTTGCATATTTTAGAATACCAAAGACCTTGGTGTCAGCTAAAAACTTATCTTTTATGTTCTACTGAAGAGGTCCTGTACGTGTACAAAATTTTCATATTTGGGTGAACTATCTTTTTGAGGCAAAATGAGCTGAGAACAACAACCAGCAGAAGCTTATTTATGTTGCATCAAACCATGCAAATGCATTAGAATTTTAAGTAATACCGATTTATTGTGCCCTTTAAAATTGTGATTCTGTCCTTACCAAAGATTCAGAGGGGCACTTTTGTTGGTCAAATTAGGACATCATTTTCTATCGGATTTACATGAGTGAAAAGAGTGGAGACAGTTACAGTTGATGTTGTTATCATTGTTTGTATTTACACTTGTTGGTTTTAGATGATATATTTAAGCTGCCATTTTATTGTATAGCCATATATTACATGTCATTTTGTTTACACATTTGAGAGCTTGTTTAACCATCTAGTCATCCTTGTTTACGAATCCAAAATCCTACTATAATGATGTTCCTTGACTATATAGTAAAATCTTGATCTTTAGACAAGAGCACAGCGATCTGTCCATTTACACTATATACGCACAGAAAAACACCATTTACTTCTCTCATCAAGCATTTGGTAATGGTCACCACATTTTTGTCACAACAGTCAAAAGCTTGTCGAAAACAGGAAAAATGCACTAAAATTGTAAATAAGTGAAA |
| 286 | lncHLBrBl_003 | GCTGTGGTAGCAGGAGTTGTGGCGTTGAGTCTGTATTTAAAGCATACAATTTCCCTGAATTCTGTGATGGACATTCATTCCTAAGGATTGGTCACAAGATGGCGATTCTCAGCTGCTGCAGGAATGAGGAACGCTGGCTTTGCTGCGATGTTGGATCATAAATTGGTAATCAAGTAAGATTGAATATTGTTGAATAATGTAAAATTGTTGATTGTAATACTTTTTGCATAATGTGTTGGTTCAAATAGTAAGTTATATAGCAAGTTATATTGAATGTGAAATTATTTGCAAAGAAATGTTTG |
| 287 | lncHLBrBl_004 | CTAAGATCCTGCACGGGAGAGAGAGGGAGAACGTGAATGCGAAACTGAAAGCGGACTGCGCCTCGTGTAGCAGAGCAAACTTAAGCAATTAAGCCGGACAGGTTTGATTTAAGGATTTAATGTGTTTAGGGAGTGAGTAACAGCAAATAGGCTATTCTGTGTAATGTTTTTTGTTTTAAATGCTCCCAGCATTAGTTAAAGTGCGTGAGAAACGTGCAAAAGGGACTGGTTATCAGATCAGGAGACAGTAGATACTGTGAACGAACTTTTCATCGATGCGTATAACGACGATACAGAGGATTTCCCGCTTTTGAAGCATATATACTGTTTGGACCTGCCGAGGGGTTCGGGGGTTCCTAAATACAGAGGATTTGCTGCTGTGTACCTGCCGTGGGTGTCAGAGGCTGTCTACACACCCAATTGAGTGCCCTAGATCAGGAGAGCTCAGTGGATTAAAG |
| 288 | lncHLBrBl_005 | ACGCTATTCGAAAAAATTCCACACTAAAATCAGCGAGATGTGGTGCGAATGGTGTGTTAATAAAAAAATGAACTCAATAATAATGGAGTGTGATGAACATGAAGCCGCATAGCATGATTCTTTGGAAACAAATGTAAAACTGACCGGACCAAAATGAAACACCCACAATAAACAAACAAACAAACAAACCTTCGATTAATAGATGAAAGACTTCAACAAATTCTTGACCCAGAGCGCATAACCTGAAAAGATAACCCACTGTGTGCTTGTGGGATTGTGACACGTTTAGGGAAAGCTTCCTTCAGGCACAATACAACAACTACAAATGGTATGGCTTAGAGTAGACTGAGCTGTGAATTGGGACTAGACAGCAAAGCACAGAAGAAGAAGAAAAAAACACCACTTTAACACCAATAAAACAAATCAAACTATACGATAAAACAAGTGCAACCAGGTCATGTGCTAATACTGACAATTATGATCAGTAACTCACAATGAGGTTTCAATTCTATGCAGTTTGTGTTCCTTGTACTCTTAATACTATAACAAGCATCTTCTATATCTAAAAGATTAAATAAGATAGAGAAATTTTAAAAAATTACATCATATAATACATATATTAGTATAATAACCAGTGTTGGGTAAGTTACTTTTAAAAGTAATACAATACAATCTAAAGTCACTTAAAAAAACTAATGTGTTACTTTTTAAGGAAAGTAATGTGAACGTCTGTGCTGTGTTTTTATATTTTGCATATGTTAAACTATTGAAAAAAACAATCAAATAAAGAATAAGATAGGCTTAAAGGGATGGTTCACCCAAAAGTTTACTCACCCACCAAGTCGTTATAAAACTTTAAGACTTTCTTTTTACTGTTAAACACAAAAGAAGATATTCTGAAGAATGCTGAAAACCTG |
| 289 | lncHLBrBl_006 | TATTCCTCTGCTATCCACAGCTGCTAAATTCACTGTTTCGACTTTATTTGTCAGGTTTTTGTCCTCAAACTGCTCCTGCGTGTAGGATTAGTAAGCCTTCAGTTGTGTTTTGATCTGATTTCTCACTGTGAAATAACTCGAATCATTCAGCGTAGTGATACAGAGCTGTAATGCACTCAAAACCTGCTGGAACTATTTATCTGACCTGCACACCTTAGAATGCTCACCGGCCAATCAAAATCAAGCAGAATAAGTAAGGAGTAATCAATGGATTGGCTCTTTTTATGTCATTAAGTATGTTTAAAATGCACGACAAGCCATAACTACTGCTTATTCCATGGTCCAAATAATATAGACCTCCTTTCACATTTCTAGGTTTCTCAGCAGCGGTTGTCAATAGAGAGCATTTCAGTTTGCGCACATAAAAAAGAAAACCTCGACATTAAAAATCATGTGAGACTGAAAACTGCAGAATATTTATATATTTCTATAGTCAAATGCACTGACCTGCTCATACACTACATTAGGAATACCTCTTTCGAAGCAAATATGTAGCTCTAAAGCTGTATAAATGTACAAATGTTAGT |
| 290 | lncHLBrBl_007 | AAAATTTTTAATTAGTAGATGGGGCTCTAGGTAAGTTGTCAACAGCGGATGGCGCTGTAGGCTAGTTTTTTTTTACCAGTACTTGGTGCTCTATGCTAGTTTTTAACAGTGCTGTTTGCTGGGAAAACTAGCCTAGAGCAGCATCTGCTGTTTAAAACTAGCCTAGAGCCCTGTCTGCTGTTAAACACTACCCTAGAGCGCCCATTCGCTGTTAAACACTAGCCTAGAGTGCAGTCTGCTGTTAAAACTTGCCTAGAGCACTGGCTACTGTTACAAACTAGCCTAGAGCGCCATCTGCAGTCAAAGCTAGCCTAGAGTGCCTGCTACTGTTAAAAAATAGACTAGAGTGCCGTCTGCTGTTAAAACTTGCCTAGAGCGCCGTCTGCTGTTAAAACTAGCCTAGAGTGTCATCTGCTGTTAAAACTACCCTAGAGCACTGTCTGCTGTTGAAACTAGCTTAGAGCGCCATCTGCTGTTAAAACTAGCCTAGAGCACTGTCTGCTGTTGAAACTAGCTTAGAGCGCCATCTGCTGTTAAAACTAGCCTAGAGTGTCATCTGCTGTTAAAACTAGCCTAGAGCGCCGTCTGCTGTTAAAACTACCCTAGAGCACTGTCTGCTGTTGAAACTAGCTTAGAGCGCCATCTGCTGTTAAAACTAGCCTAGAGCACTGTCTGCTGTTGAAACTAGCTTAGAGCGCCATCTGCTGTTAAAACTAGCCTAGAGTGTCATCTGCTGTTAAAACTAGCCTAGAGCGCCGTCTGCTGTTAAAACTACCCTAGAGCACTGTCTGCTGTTGAAACTAGCTTAGAGCGCCATCTGCTGTTAAAACTAGCCTAGAGCACTGTCTGCTGTTGAAACTAGCTTAGAGCGCCATCTGCTGTTAAAACTAGCCTAGAGCACCGCCTACTGTTAAAAACTAGCCTAGAGCGCCATCTGCTGTTAAAACTAGCCTAGAGCACCGGCTACTGTTAAAAACTAGCCTAGAGCGCCATCTGCTGTTAAAACTAGCATAGAGCACCGGCTACTGTTAAAAACTAGCCTAGAGCGCCATCTGCTGTTAAAACTAGCCTAGAGCACCGCCTACTGTTAAAAACTAGCCTAGAGCGCCATCTGCTGTTAAAACTAGCCTAGAGCACCGGCTACTGTTAAAAACTAGCCTAGAGCGCCATCTGCTGTTAAAACTAGCATAGAGCACCGGCTACTGTTAAAAACTAGCCTAGAGCGCCATCTGCTGTTAAAACTAGCCTAGAGCGCCATCTGCTGTTAAATCTAGTCTAGAGCGCCG |
| 291 | lncHLBrBl_008 | TTCAAACCCCACAAAGCTGAAAGAGCTGGATCTGAGTAGGAACCAACTAGGAGACTCTGGAGTGACACAAATCTCTGCTCTGTTGCAAAATTCACAATGCGCTCTGCAGATACTCAGGTAAATAAGCAGAATTTAATATAAAGTTTATAAACAGTTCATATATTTAACGTTGTTTGTCTTACTTTAGTCTTTCAGACTGCAGTATCAGTGAAGAAGGTTATAAAGCTCTGGCTTCAGCTCTAAAATTAAACCCTTCACACCTGATAGAGCTGGATCTCAGAGGAAATGATCCTGGTGAATCCGGAGTAAAGGAGCTCTTTGACAGTCTACAAA |
| 292 | lncHLBrBl_009 | TATAAAGGCCCTGACAGTATGCTGAGAATTTATAAATCTATTAAAACTATTGGTTTTCCATAATGTTCTAGTGAAAGGAAAAAAGTCTACATCTATAAAAATCTATAAACAGCAGATTTCTATCTGTTCAAGTCACAAAATTTACCACAGTAACTGTCACCAACCTTACGGTTCTTATCAAGGTTTTATTACTGCTTATTTTACCCTGTGTGTCCATTCTAATGTTCACTCTGTGCCTTTTTTTGTCTTTGGGTGTAATTAACTGGTATACACATTAGGCATGGGCTGGCATAAGTTTCTG |
| 293 | lncHLBrBl_010 | AAGACACTTCAGTCTATTGCACTCACAAGTTAGGCCTCCAATAGGATGGACGGGAAACTCACAAACAGCACTAAAACTGAGTCTCCCGCTAAGCACTGATAGGTTGAATATTATTGATCCCCCCACAAATTTTACAGAAGCGTTTACTCGTCGTCGTAAGGCAGTTTGGTGGTCTGCAGAGAGCCCAGTTACACCTCTCTACCCCCCATCTTTAATACAGAGCAGCTAAAACCACAAGAAAG |
| 294 | lncHMBrBl_001 | CAGCAGTACACACACACACACACACACACACACACGCACACACGCACACACACACATACACACACACACACACACACACACACAAATAGAAAAATAAGAGGAAGTTTGACAATTCTCAGATTATTTAGATTATTAATATACGGCTGTCGACCAACATTATATCTGATAGTTGATGTGATTATTCAGTTTTATTATTATTTCATGTTAAAGACTTTTATGTTTATGTTCATGCACTCAACACCGTATTAAAGTCTGTTCAGAA |
| 295 | lncHMBrBl_003 | TCCGGAACACAACTGTTGCACTCTATTTCACATAAACAGAGGCATCCTATTTGAATTCATGCATCATTTTAGCAGGAACCTATAGAGCATTCAGAAAGTAAATGATCTGTATGAAGATTTGGTTTTTCACTGTCCATCCCTATGGTGGATGAGTTCATAAATCCTCTAGTCTTTTACCTCATTATAAATTAGCCTTTGGATTTTGTGAGCGCACATCATTTAGCCTGTGAACTGTACTCAGGGGATCCATAAACGTTCCCGCTGCAGACTCAGATCTTGATTTATTGTTTTGAT |
| 296 | lncHMBrBl_004 | CTTCTAAAAAGGTTACAAAAGTTTCAAATGTACTTTGTTCACACAGTCAGCATTTGCAGAAAAACAACAGGAAGTGGATCAAACTAACAACAGCAGCGGTGTGGAGCCACTTTACAGAAAATTAACCCTGTAAAGCCTGCACATATTCTACAATAAACCTGCTATGAAAAACATGATCTTAATAAAAACTTAATAAGAAACCCAGCAAGTAAAATACTTTTTAGTATCATTTGTGGGTCTTTTAACTGTGAAATAATGTGACTTTTTTATTGTTAGCAAAGTTTACTTGACTGAAGCAACTTAGTTT |
| 297 | lncHMBrBl_005 | TGACAATGAAATTATACATACTTAGAAAGGCCTAAACTGGTGATATTTATCCATCTTAATGATCCATATATAAATCAAGCCAAACTGTAAAATGTTATGTTTTTCTTGTGAACCAGACCAGCCCTGAACACATGCTGGTCTCAAAATATGAAAGAAAACATGTTTGCTTTGAATCAACCATATTTGGCTCTCTAACTAAATTCTAAGGGCCTTATTTTGATATAAAAAAGTTCAAATCATGCTATTTGTATTTATAAGACTGCTATTAACCTGAGAATACTATGTTTTTACATGTAAAAAGTTTTTTGGACTGAAATTACATAGGACACTAATATTATGATGCTAGAAGAGCATTGAAATAAAAGCGAATAACATTCTCCAAAGTGAGCCTATGAGTATCCCATGTAATATAAGTACAAGAGTGCAGCACTGACCATGTATATGATTTAAAAAGAAATGTGCATGGTATTAATGCTTACATGACCACCGTTTAAATTAAAAGTGAAACTGTGCCTGTGACAGCTATCCCCGAGATAAAAACTGAAAAAAAGAGAAGTTGCATTGGAAATCAAATTAAAATTATACAGATTTTATACAAGATCCACACAAGTAGTACATAAATACCTGATTTTTTTTCTTCCAGAAGATTTCATTTGTAAAATTGCTACTGGCCTGCAGAACGACTGAGCAGTAGCTATAAG |
| 298 | lncHMBrBl_006 | GCAAGCTGAGTGAGAGTCACGCAGTTCATAGCCTACGGCCTAGGAGAGGAGTTCTGCGAGAAGATATTTGCAATACATTAAAACATTCTCGACTGTATAAGTTGTCATCGCATTACTTTATGACTATTTCCGGGTGAGATCACGGCTAGGTTCGAGTGTCTGTGAGCTGAACTGTTCGCGAAATAGCGGATACGTGTGGAAAGGGAGGGTGTTCAAAGCAAACAGGGAAAAAACTGAGCGATCGCTTGTTTCGCTCAGCCAGCTGAAAGAAGCAGGAAGCGGATCAAATCAGTTCACCTGTCGAAGGAGAGACGGTATTGGGAAGCTTCGTTCATTTTTTTTTTTCGTCGTGTCGTGATTACATCTCAAGTCGATGTTTTTTGGAGTTTAAATCGCAGTAAACTTTTAACATTCATTCTTTCGCTAATCACACAGAACTGGTGTCGTACATTTGAGCCCAGTGGAACACTCTG |
| 299 | lncHMBrBl_007 | ACACGCTCAGGACTGGATTTTCGCAAGGATCATTCTCCACCATTACCTCTGCTATAAAGCTACTCCTACAACAAACTGTGAGAGGGATTGCTCAGTGATCTCTGGCATCTAAATGGAGTCTTTTCCTGTCTGCAACAATAATAACCAATCTGGAAATCCCCCTGCTTTGCTGCCGTCTGGTATGGTTGGTCACGTGATTTAACGCAGTCCCAGGGGCTCAGAGCCCTATTGCCCTGGTCTCTTGGATGGTCCTGTTTACTTACACCATCTTATTGCTGCTGAGAATTCACAAATGGCCTCGGGACTAAAATATGGTCATCCCCTGTTCATTACTGCCCAGGTCATCTAGTAGCACAAAATATCGAGGAAGAGTAATTGGATTAATTTAGTATATTATCATTATAGATTTGTTAAAGATGTTCTTGCTCTTCTATAACACACACATGCACAAACACACAGATATATGCGCTGTGCACTTGTATACAGTCTAGTTTATAAACTGTGTATATACACTCACCGGCCACTTTATTAGGTACACCTGTACAACTGCTCGTTAATGCAAATTTCTAATCAGCCAATCGCATGGCAGCAACTCAATGCATTTAGGCATGTACACATGATCAAGATGATCTGCTGCAGTTAAAACCGAGCATCACAATGAGAAAGAAAG |
| 300 | lncHMBrBl_008 | TTACGTTCCTGCCATACACTTCCACTGTAGAGCAGCGGAATTACTGATGGCCAGTTCCTCACAAGACTAAGCGACGGCAATACGATTCCCTCAGAGATTTAATTGACTGCCACAAGTGCGCATCATATCTTTGCAATGGAGCCAGAGGAGAAAAACCCAGAATCTAAATATGGCGAGCCAAGGAAATTTGATCCCACATTTAAAGGCCCGATTTATAACAG |
| 301 | lncHMBrBl_009 | CAAAGGCGTGTGTCAACTCTGTGTGCCAAGGGCTGCTCCGCTTTTACCAATGCGATTAATGAAGACAACGAACCAATTGAGGGAACACTAAACTAATAACCTTCCGTTTCGTTTCGTCTGTTGTTTTTAATACGGACGAGCTTCGTCTCTGCAAGGAATCAACCCTCTGCACGAGCACAAAGGAAAATTTAAAAGGCAAGCAAAGGTCGGTTGTGGAATGTCACAGCTTTCTCAAACATCCAGACCTTTATCCTTGCTCGTCTGCAGTCCTGCTGTCTGATTGGATCATCC |
| 302 | lncHMBrBl_010 | CAATAATAACATTAGCCATCGTGTTTTGTAAATTAAAGCCTCACACACACTTGCATTTATTATTGGTCTCTAAGATTGCCCTTATCATATAGCTTAATTACAGCTCTGCATTCAACCTCCCGCTATTCATCCAAAGACAGTCTCTGTTTTAACACCAAGACATTTCTGCTGCCTTAACAATAAGATTCTTTTCCTCTTGTCATTGTAATGGCAGGGATCCACTAACGGCTAGCAATTAAGCATTTAAACAGTCAAGACTGTTATTAGAGTCTTAAAAATAAGCCATCAAATGGAGGCACATTAACAAAACATCTGGCGTTTGAGAAGAGCTTCAGTCGTGCTTTGTGCATTTATTTGTGAATGTTTAAAACCACTCTTGTTTCCTAACCTAGTTTCTGTAACCTGTTAAACAGCAATCGCTGTTTCCTGTTTTTGCAAATGTTCTTGTTTCAAGAATGGCAGAAACCACAGGAACATTGTCTGAATCACTGGTCTGAAGCAGCAGAAATGAATGATTGAAAAACTGTCAGTGAA |
| 303 | lncHMBrBl_011 | CGTACGAATTAGCCACTAAACTAGCAAAGTGTAAAATACTTGCGTTTTCTCGTGAGATGAGGTATTTTCTGGTGCAGTTCACAATTTTACAGTCATGATAAGCTTCTCATGAAAAAACAAACACACGGCTCATTTCTGAATGATATGACTGTGATTTATTGAGATAACAAAAATAAAAAAAACAATAGTTTACCACACTGATCTGTGGTGCAGGCGTTTAATATCTAATGTCATTTGCATGATTAATATATTGCCGTTTTGGCTTTGTTTACTTCTGTTGCATTTACTTGTGATCACTGATAAGCTTTAAAAGATGATGTTTACGTTTGACATCATTTGCCAGACACTTTTATCCAACACAACTTTATAAATGTGACCCTGAAGAACAAAATGTAAAAAAAAATAAAACAAAAATTTAGATGTGAATGGCTA |
| 304 | lncHMBrBl_012 | CAAAAATCATAAATAAATAAATAAAAGCACAGCATTGTCCATGATCTTGATACAAAATATAGTGGAGTAAAAAAAAAATCAATAATTAATAAATCAACATTTACCAATAAGCCACAAATTGTCAGATTTTCATTGCAAATTTACACAATACCTTGTGTACCACTTTCTAAATAGGCTTCCCAAGTTTATATTAGCATCGTGGGCCTAGTGATTGCTGTATCAAGATCTTAATTACATTTTTAAGTCGGATTTAACACATTGCAGATTTTAAAATAAAATTACGTTACAACAGTGTGGCATAAAATTAGTTGGCGTTAAAAACTCAGCCGTGTATTATAGCACTATTTATACAAACAGCAGTAAAAGAATGGCAAACAACGTATTGTGCCACTTACCAAGCACTATCCATTGACGTTATGTGTTGGATCAGCCTGATCTCATGAGGATACGTAACTATTGTACATTTTGTGAGTTTAGTGAGTAATTTGTACAAATTCCTCGAGTTCAGTCATACAAAAAATGTACGATTATTAAAAGGAGGCATGGCCAAAACCAAAACCCCACCTCTTAACCTAACCGTCATTGGGGAATGAGAAAATTGTAGTAAAATTTGTGAATGAGAGCTTACGAATTAATGCGAATAGCCACTAAATAAAAAAAAAAAAAGTAAGAGTTTG |
| 305 | lncHMBrBl_013 | TTGAATTCTAATAGTTGGAAAACATTTGTAAATGGCCAATATGTAAAATACATATAGTAGCATTGACCAAGAAACTAACAAATTATTCACTAGAATTAATCCTAGCTCTCGAGCAGGCTAGCTCTGTAGTAACTTAATTTAGCCTGCTTTAATGCGACCGAGTCAAGCCAGGATAGTTTAAAAATCCCAGCTTCACATTTTGCTTTTGTGCAATACCTCTGTGGGGTTATCAAGTTTATGTCTAAAGACAAGATCAAAACAAAGCTTCACAATGTTCTATAATTGTAAAAATGTTTTTTATGTTAATATTCTTGAAAAATAAC |
| 306 | lncHMBrBl_014 | TAGGTACCATATAGGTAGATCAGCTCAGCGGCTCCATAATACCATAACAACAAAGTGGGATTGAAGTCGAGCTGGCTTCATGTCACCTAAAACCCAAGAATGGTGCAAACTAAACCAAAACTTGACTGGCTAGCCAGCTAATCCAGCTTCATTATATGGCCTCAAGGCTTACTTGCACTGAAAATATTTAGCAGTCACAATTTTTAATACTAATGTAATTCTCTTTTATGACCAAAAATCTAGTTTTGTATATCATGAAATGGCTT |
| 307 | lncHMBrBl_015 | TTTAGGGGTGAGGTTTGGAGCTATGCCTCCAAACAATCATACACTATCATACAAATGAACGCATATGAATGACCCACTAAACTGACAAAACGTAAAATAGTTTTGTTTCCTCGTGAGATCAAGCTGGTAAATCACATACTGAGCACTGTATTAACACTTTTACATAGAGTACACTTGAAAATAACAAAAAGTGCACGTTGCATTATATATTATGATTTCATTTGTGGCCAGTGACCACTAAATCGTCTTATATGATCGTGCTGTTTACTTAAATACCAGTGAACACATTTGAGTTCAATTTTATGTTTGAAAAGCAACAAATGAACCCCACCTACTACATTAAAGATGAGCTGAGCTTAGAAGACAATAACAAAGGTAGTCTTTGAATTTTGCAATAAAGCAGGACTTACTATGGAACATTGATTCCTTTAGATTAAAAAAAGAAATACGGTTTTATGTAAGAATTGATATCGGAGTAATAATACAGAGTATTTTAAACTAACATCACACACTTCTGATTTTCCTCTTCTCAGAACTGCTACTTCAATTTTTTATTAAGGCAATA |
| 308 | lncHMBrBl_016 | CACACACACACACACACACAGGAGAGAGAGAGGGTCTGATCCAGAGTCAGTGAGGACAGCAGAGTTTTATTATTCAGCACATCATTTAATAATAATGGACATGAAATAGAAACTCAGCTCAGTGTTTGCATTTCTGGCTTTAATGTTTAATACACACTGATACACGCATGAACACACAGTACACCTGCAGACATGCGCTAGATTTGACTTTCCTGATAAGTTCTCATGCAGTCATCTTATTTTAATAAAGCACACACACACACACACACACACACACACACACACACACACATTACTTAGAGAATAATGCCCTAATGCCACTCAAACCTCATTAAGTGTGTGCTCATGAATATGCAGATTAGTCCCCGCCTCCATTATGGAGCTTTACATCTGCAGAAACATTGTGAATATGAAGCGATTCTTTGAGTTATTATCAATTGTACTTTAGTAAAT |
| 309 | lncHMBrBl_017 | CTTTGATTATTAAAAAAAGCATTACACATAATTTGTCATAGCTTCAATACTGTCTGATGATCCTTTCGGTGGACATTTAAAATCATGTATGTGTGATAAAACGGCTGTAAAAGTTCAATCGAGATTATTATTGCTGCCACCTACTGGTGAAAGATGACGGCTAAATAACCACTAACTTCTGAAACGAGCGCTGAATGCAAAAGCCTGGAAGGAAGGCACACTTTTGGCTGAGGGACATGTAGTGAAATGGTGCAATTTCTGTCCTTTCTGATTAGTTTATGCAACAATTCACTTCACAACAGCATGTGAATGAGACCTGGCCCCCCGAAACACGTTCATGACGATCATAATGTTTACAGTTCTACTCGACACATTCACAGCATCAAAAAAATAAGGCAATAAACATGGGCCAAAACATGGCTTTACAGAGAAAAAAAAAAGTGCACAAATGGATGTGAATCTCTGTATTTTACTAAATATGCTACTGAAGTTATGGCCTTGCCACACAGCGCCATCTACTGCTCCGAGGTCAAAA |
| 310 | lncHMBrBl_018 | CAACAGCGTGGTAAGAGTGAGGCTAACCTACGCTTTATTTAATGCAGTACAGTATCAGGTGACTTCTCTAAACCAGAACACGGAGGACAGAAAACACACACCAGTATGAACAAGAAGAGACGCTCAGCATCCTGTCACAGGTGGGTAACAAATAATAAAAAAATCTGATTCAGTGGCTGTTGACTGAAGAATAGCACTTTATATATGTGCAAGGATGTGCTTAAAAAAAGTTTATGAACATTAAAAAAAAAAGATTGTAGTGCTACATAACATCGCTCCCTTTAAGTGCTGCTGAAAGCATTTTACAACACCTCAATCACAAATCAAAAATATATTATTGCAACTAATTAATACTGAAAATACATGATTAACATGAGCATTTCATAACCATAAAAAAACTCTGTTATCAAAAATTAAAGTAGGAATTCCTCCAACAGGAGATTAAGTTAAAAGTATA |
| 311 | lncHMBrBl_019 | GCTCAAAGTTGCATACCTTTATTTTATACTAATACTTGTGAATGTGATTTTATTCTTTATAGTTGCTTATAAAAACTAGGGTTTGGTTTGTCGTGGTTTTAGGCAAAAGGTGCTAGTAGCCAATATACCTTAAAAAAAATGTTGCGCATGAAATTGTGAACAAGTTTGCCATCTATGTCTGCAATGCAATACTGTAGCAGATCACATTTGACCTGTCCGGAGCGTTAACTATACTTTTGTGGACTCGGTTTTGTGAAAACGGTTAAGAACATGATTAGATTTCAG |
| 312 | lncHMBrBl_020 | GGGGTTAAAAGAGAGCTGAAATCCAGTCAGTAAAAGACTCTTTTTGTATTTGTATTGTGTATAGTTTGCTAACAATTGTGTATATGTGCCTGTACATATTGTGAAGGGGACGTGCTCCTTTTTTTTTTCTCCTCCAACTTGGACAGAGTTACTGTCTCCATTCAGGAGAAATTGCGAGCCAAAATGTTGACATGAGTGAAATGGATCATTTGTATCTGGAAACAAATAAAGCTATAATTTTAAC |
| 313 | lncHMBrBl_021 | GTTGATGCTTCAATGCTAGAGGTGCCTTGTTTTTATTTTAAATCCGTGCCTAAGAAACGTAGGTAAGAAGCTTGAAGCTGTTCCCCTGACACTTGAGATTTGTGGATAGTATTTTTTGGCAGCGGTCAGCGGCTCGCATTGAAGTAAACGGCTGATGCACTTTGATTTGACGCGGTGCAATGTATACAGGCACCTGCGCGCTTACACCCTACCTAAATACACACATGTACCCCGGTAAAGCTTTGTCATGTGACAAACAAGGTCATCGCTAACCACAACGCACATCTTAATCCTGACCTGTTGTATTTATAAAAGACCATTGATGGTTACAGCGCTAGAATGTGTTTGTTTTTGAACCCACAATGCATTTGTCTGTTAGCAAGCGTTCAGTATTTTGTAAAGGAAACCAAACGTCTGCTACTTGAATGACAGAAAAGCCATCTGTGTGTCTAAACCCAGTATAGTAGCTTAAATGACAGTGCCGTTCGCCAATATTGGCACCCTTGGTATAGAATAAGGATTTGAGAAATGTCTTTGTCTGAACTTTTTGCTCACAACATTAGAAAATAAAAAACTGAACTGAGAAAAAAAAATCAATTGAGCAGGAGAATTCAGTAAACGCCTGTTACTGTTTCTAACTCTGATAAACAAATAGAGTTGCTATTTATTACGACAGTTGTTGGTGTAAAAACCTTGGTGATATTTTGACTCGTTATAG |
| 314 | lncHMBrBl_022 | ACTCGTCCACATACACTCCTACTCTCTTACAGGAGTCTGCGGACACACGCGTGTCGATGTGCTTGTAACCGTACCAAAGACCGAATGTGTCGTCAGAGCAGATCAGGCACCAGGAGTTATCATTGAATCCAAACACACAGTCAGCGTCCTTTCCTTTCCTGCCTATCCCTGCGTATGTGATGGCTATGTCAGCCTGTCCAGTCCATTCAGTCTCCCAGTAGCAGCGTCCAGGCAGTGCTTCTCTAGACAGGACATGAGGACGCTTGTCAAATCTCTC |
| 315 | lncHMBrBl_023 | CCCCGCCCCTGGAGAATTGTCAGTCTACAATAATCGATTATTGGCTCCTTTACTAGAAGGCAGGACTTCCTGCACTGCAGTGGCCAAATTGACTGTTACCTTTTTCCCCATTCAAAACTATAGAAGTGACACTTCTTGCGTGTTCTATGGTCTTTATCCTTACCCAATTTGTCAAACCCAAAAACGCTAGTCTTTCCGAAGTGTTCCAGATACTGTCAATTGTTTTACACTATTAATTTTTGCCTTGGGACATCCATTTTTGTTAACAAATCACCACAACTTTAACCAGACCATGCCAACATCAGGCTGATATTGTGTAGTCTGAACCCAGCATTAGTGCTTTGCAGATGTACAAGAAAAATTCCTGTGTTAGTTCCATTTCTTGTTCCA |
| 316 | lncHMBrBl_024 | CTAGTGGCCCTAGTTATATAGTGTGTATTTATAAGTCAGTTACAAGCTTACAGCATGTATGTTTAACATGGACATTAAAAACAAAGAATGTCCAATCTGTGTGTTATCTGTATCATCCACTTAATAATGTATAAACAAAGGAGGTAATACGGCCACAAAAGCCTGTGAATGAATTACTGCATTTCTGCTGCCACCATGTGCATCTCCGATCTTTAAGCCAAGAGAAACGGTCAAAGGGAACCTGACAAGGTCCAACAGCAGCTCACTCTAAATATACAAAGTGT |
| 317 | lncHMBrBl_025 | AGCTATTTCTGGCGGGACAGCAACATTTAGGAAGTGTGGGCCACGGGCCAGCGAGCAGTTTAATGACACGCCTGCGTCACATGGTCTCTTGTCACAGGAGCTCCCGCCATTGTCAGGGTTGAGCGCGGGCACGCACACACAAGGTTATTTGTTTTTCCAATGGGGCCTTAAGAGAGCAGCTGCAGGTGAAAGGAGCCCCCTCTGCAATTCACCATAGAAAACACAGCAGTCCACGGCCTGTCCTGTGCTGCACTGGCCAGCAAACCCTAAAGTTTCAAATA |
| 318 | lncHMBrBl_026 | GCAAAATCACCTGTGTTTATTTACAAAAATAAGATTGCAAAGCAACAGCAGATACATTTTAGCCAAGAAAAACAAAGTCTTCCGTAAATTCTAAGCTTATTTGCACTACAGCAGAAATACAGGAGCTTGCCGTAGTGAGGCACTGGGAAACTGATGTAAATCGAGAAGCACAAAGAGACGTGGAAAATGAAGATAACAGAGCGAAGTGTGTGGCGTTTCACACAGATAAGGAACAGCAACTTTTGTTTTCAGAAATAATGAGGCAAATTTAAGTGAGTTTATCTTAAAAACAAGCAAAATAATCTCCCAACAGGGTAAGCAAAAATCATCTTATAGTAAAACTGATATTTTGC |
| 319 | lncHMBrBl_027 | CCTGCAATGGTTTCTTTTAACCAGCACAAACTTATTTCAACCATTGAACATATTTTACGTAGCAACATATTCAGTCAAAGGTGTTAAACTGGGTGTAATTAATATGTGATCAGCTCAAAGAGAAAATAACATTAGTAACGAATGTACATACCATCAGATATAATTGGAAAAACAGCCATAATCAAAAAAATAAGACTCTCCATGCTCAGCTGAAGTCATGATCACGTCTCTATGCTGTCTAAGGGAAAATCTGTAAATGATCTGGTCATGAAAACATTGTGTTAATTTGCTTTCATTTTCAACGAAGAGTTCATTGCTACAAATCCAGACTAACCAGTTTTGGCTAAACTGAAAAACTTTACTATTAAGGAATAAAACCACCTGCAATACGTCTGAAAGTGCATATCATCAAGGGTTCGACACCAAATGAAAACAGTTTGAAGGATTTAATGGCAAATTGTAGCTTGTTTATCCATAACAAAAGATCTAGA |
| 320 | lncHMBrBl_028 | CATAAAACCAAAGTTTTACTCATTTAAAGACTCAAACAGCAGTGTATATAGTTTGAGTGTATGATACAGTATCAGCCTTTGTAGTGGTGTAGTGTAGCATAGCTTTGTATTACACCATTATTGTATTCCTTTAAACAGCTCATGTCCGATGCCAAAAAATTAAATATTAGTCTCAATATTGGATATGAAAATAATGTCCTAAATTGCCAAACTTACTCCTATGCATGACTTTTAGATATTTAACCATGAACGATGCAAAGGTAAATGCTGTAAATAATACCTCTGATATTTGTAACGCTGTATAAATTGTACAGAACAATTGTTTGGAAGAAAATGCTTGCCAGAGATGTTTATCAAATAAAGGGCTTCCAG |
| 321 | lncHMBrBl_029 | CTTAAGCCAAAAAATAATCAGTTATCCGCTGATTGATCGGAGCATCACTTTCATAGATAAACCAAAGAACTACACTAAACTGTGTTGGTACACAAAACTGTGTGTGTTGAGTTAAAACTGTAAAGTACTATCAATGTGTTTTGTACTCTGACTACTTTATCATGTCTGAATATGTTTTCTGAAATGTTTGCAATGTCTGTGTCTTCCTGTATATATATTGCCTTGTCATGTTTGTTGTGTCTCTTTGTTGTGC |
| 322 | lncHMBrBl_030 | GAATCATGTAAACAAACCCCCAATGAGACATATACAAACTGAAAACTGCTGAACTGAAGCACTGCTGTGTTTACTGAGCACTGAGAGACACTATCTCAGTCATGACCAAACACATCATGATGGACAGACATTAGTCAGTGTCTTATTGCTGTGAACCTGATCAGCTGTTGTGTTTGAGTGTGCTGATGCTGTTACTGCAGTAATCCACTAGAGAGCAGTCTTATAAAGCTGTCCAGTGGAGGAACTCCTGTTGAAGGAGTTTTTATTGGAGCTTTGTGTGCGTG |
| 323 | lncHMBrBl_031 | TATATTTTTATGTATAGTTATAATTAAACTACAGACACCACTAACAAGCGCTGAAGAGGGCAAAATGCCATATATTTTGTGCACTATATATTTTAAAAGCGCCTTAAGGTCATATTTAGTTTAATCTTTCTGTGCAATCAATCAGAGCTTATTGAGTGCCAAATTTTGCGTCCGCTTCTGCTGTGAGATATTTTACCACTAACAGATTATGAGGACCAGTGTTTCTTTTCTGCCTTTCTCTGCTGAAAAGGTCATTTTTCTTTAGTTAGTTTGTTAGATATGCACATGTCCTGATTGTGATCCTGATGTTTTTAAATGTTTTATGTTGTTGTTGGTGATTTTCTGATTCGTCTTTGCCACTAACAGTTATCTGTATCTATATTGTTATGAATTAAACTGTATAAACATTGATTAAGGCTTTATTTATCCATTCATGAACAAGCCATTTCTCTGAGAGAAACTAATCGTGTTGCTCTTTCAATACAAATAAAGATTCG |
| 324 | lncHMBrBl_032 | TTTAAGCCTTTTATTGCTATTAAAAACCATTCTCCTGAATGAAAATCCAGGCAAAAAATAAGATTTGGCATTGTGACATGATGGACAAAAGGAAAATGAATCCTGAAACGGTGTGCTGAATGAGTGAACACTGTTTCCAGAAGGAATTAAACAGTTAGCTGTTAGTGAACACCATCACTACTTACAAACAGAAAAAATAGGGGGGGGGGGGGGGGGGGATGGGTGAAAAGACAGCAGCCAAGGTTTTTAAACAGCACATTCTCAATTCCATAAACTCTGAATGATATTGAATGATTGATATTGCCGACAGTCACACAGAGCTCCTCAACAACACACTGCTGAGTAGTGTTGGCACCAGTGTGGTTTGAGCTTGCACATCCAGGTCACTTTTACTACAACACTACATCAACATTTACTCTAATCAGATTCAAAGCAAAGCTCTGCAATTAGCACTTAGTACAGTACTTTCATTAAAGGTTAATCCGTTCATAAAATGACTGACACACCCCAGCGAACAATTTTGTGTTAAATAGACGTCTAATAAATGTAATCAGCTTGGCTAAAACAAAGCTAAACTTGGGCTGTCAGTGAAAATCTAATAGACGTCTAAGAATACGCCAAGTCATATAGAACGATTAGACAGACTTTATATGTGTTGTGTTTGTTTGATGACTAGTCTACTTTTAGCCTATCCTAAGACATCTATTAGATTTTCACTGA |
| 325 | lncHMBrBl_033 | TGCAATTGCTGATTTTTTTCCTCTTGCAATGCAAATGTTATACCTCACAATTTTGTATTTTGATTTGTGAAATATGAATTTAGCATTTTTCTCTCACGATTCTTCCATATTCAGCAGTTGTGCCCTTTTCCCCCCTTAGTTTTGCGACTACAGTGTTTTAACTGTTGTGAGTCAGACTCATACCTCCTCACTGTATGCACACAATTTTGAGAGACTTGCAGGTTTAACTCGGCACATTTAAAAAACTATTTTCATATTTTGCCATTCTGACTTTTGTTTATTGTTATTGTGCAATTCACAGAAAGACAAATATGAAAATATGTGAGAAATCAAATCTAAATTGCAAGAAAAACAAGTTTTAATTGTAAAATATGGTCGGCA |
| 326 | lncLMBrBl_001 | CCAGATTTGCATAATTTAATAATAAATCAGCATTTGATAAATTACAATCATAATAACAATCATTCTTCACAGAAAATATCTTCAAAAAGATCTGATATCAAATAAAAGAGTCTGAAGGAAGGTACCATTTGTGAACCTTTTTTTTTTTTCTTTCTTTTTTCTCTGAGGTGAATAATCCGCCTCAGGGGTTTAAGTTGAACACGATACACACAATTCTGTTCAGTGACATTCTTGAAATGGGATCAAGTCCAAGGAGGAAGGAACAAGCACTACGTAAATTCCCACAGAAACATTTTGTTCGGTCACACAGAGCGATGTGACGTCGATCACTTCTTTGTTGTTAGAATGAACAAGAAATAATTAGGAGGCATGAGTTTAGCACAGAACTATTAACACTGGATGATCAGCAACAACAACAACAACATCAACAAAAATAACTATTTACACTCCATGTGAAATTAAAAAGTGCTTTACATACACGTGATCTTAATGGTCTTGCCATAATAGCACATATTATTTATTAAACAACTTCATGG |
| 327 | lncLMBrBl_002 | CTCGATCAACAGATTTTTTAACAGTGTATATACAGTAATTCTGAGGCAAAACAAACATCATCATCCTACATTTATTTTTTTTTTTCGGCGTGCTAGTCCTCTAACCCACCTCAAACCTAACTTTATTTAGCTCTCCCCATCAATTCTGCTTAATTTTTAATGCTGGGAAGGGTAGATAGGCATTAATAATGCACTGTGGTGACCATTCATAATTGATGAACCGTTATAGAGCAAATCCAACACTGATACCCGGTTTATTTAGAAGTCATCAGAACGATCAATCTTAAATGTGAGCGCAAGACCTTGAGAAACCATCAACAAATAATAAAACGAACTCCTTTTCCCTTCGGCGTTCGGCTAAAATTAGAGAAAAAACATCACTGAAAGCATTTAAAGGTGAGTTTAATTAGCTTTCGTACATATCGTGCTAACTACTGGACTTGAATTGATATATTTGGGCTTTTATTCAATTAAACGGTCAGTCCATAATTCCTGAGACTACAAGACAAATGTGACATGAAG |
| 328 | lncLMBrBl_003 | GAAGATGATGATGATCAGCATGTGGGGTATCTACGTTGCAATCTTCATCAGTCGCATCCTAAATGAATGAATCGAGCTTTACATCTCTTGTGGACATTTAACGTTTAAACCGAGGAGATGAAATGAGACAATAACCAAAAATCATCGTCAAAACAGCTGCCACCCGTACAGCAGACAAAATGAGCAAGGACGCGCCTGCTGACCGGT |
| 329 | lncLMBrBl_004 | GTTTACTATTTTAACAGCATATTAATATTTAATGCATTAGGCTGATAATTCACAACCACAAGTGCTAAAACCTGACAGATGCTGAAATCACTCAGCATATGAATACTGAAGTAGTTTAGTTTTATTAAGGCACAGATATCGAGTCTGTAGTGACATGCTGTTAATAAACCAGTGCCAATTTTAGATCAGTGTTAATCTGATTGATATGGAAGTAGATGATTTATGAGTACGTTATCCATAATATTGTGATGCATGCCACTTTGCAAATTAG |
| 330 | lncLMBrBl_005 | TACCTTGCATCATGGTTGCAGCACTTCATTCCTTGGTTTCACGCGAACAAACACCCTGACATGAGATACAGCAGTGATCCGGGAGCCGGCCGTTGAAGGTAAGGCTCATAATGCCCAGCACCGCACGTCCTCGTTTCTTGAGTTCCCCGGTAACGTTAACCGGTTTGGTTAGGCCCCAGGCTCCGCCGCATTAGCAGCATCCACACACGCCATCACCACTGAGGGGATGCTGAGGACAGCCGTTGAACGGAGAATGGAAGTGTGGGAGGGTCGCGTAATTACTAACCACACTATGGCGAAG |
| 331 | lncLMBrBl_006 | CGTCACTGCGCGTTCGGTGAGTGTGCACGCGCGCGCGGCCCGGGTTTTTACCGCAATGCATCAGTCGGAGCGTCACCGGGAGCTCCAGAACTCCGCGACCGCCTGAACGAGCCGCGCATGATGGCGATTCACCGACACCGAGCACCGATAACCGGACACACGAGCCAGAGGAGCGCCTGAGGAGCAGCACAAGGCGAGTACAGCATCTGTGTGTGTGTGTGTGTGTGTGTGTGTGTGTGTGTGTGTGTGTGTGTGTGTGTGTGTGTGTGTGTGTGTGTGTGAGAGAGAGAGAGAGAGGAGACTGAGAGGATGCGCTGCGCGCACACACACCTATCTGCGCACACGTATATATACACACATCTATAAACACTCACCTAC |
| 332 | lncLMBrBl_007 | TCTGACTGTCCTATGGCCGCACAAACCGGTTGCGTTAACCCACCGCAAGCGATTCCTCTCGGTATTAGTCCACCTTCCCTGAGGCTCGTGTGTCACCGGAGATAATGTTTGGTTGCCCGGTATGTGACTCAAACTATCCGGTCGGGATTTCGGGTACGGTTGACAGATTGACGTGACGGCCAGCTAGCCAGCTAGCTAAAACGGTAACGGTAAGTTCCTCTCAGTCAGCCCAAATTCAGTCCCGAGTCTCTGTTTCTGTGACGGCGACGCCTTCAGAAACACGTCTTTGGAAAGACTAGGGCGGTTTGTAGAGGTGTTATGACGTTATAGCAAGTAAAAGTCCACTTATGTCGCCTCCGCTCTTCGACAGAGCTAACTTGCTACTGCTAGTGGCCAGGCTAACCAGCTAAGCTACATTCCCATCTGAGAGGAAACAAAAATCCAGTCGTGTCTTCGCTTAAAGTCCCCGGTCTGCCATGAATTTCGCTCTCGGCTCTAGTGGCTTTTTATAAGCGCGTATTCCCGTGGTTTTCCGCCAGTGGTCACATAAAGTAAGTTGGAAGCAGAACTCTCTCTCTGC |
| 333 | lncHLMBl_001 | CACGCACATCTGTTGTTGTCTTCCTCTTATGAGTCCACTCTTTTGTGACGTAATCCTATTATGACTGTTGTCTGTTTGGCTTAGGGGAATAACAAGCAGGTTACAGAAAACTCAAATGGTGACAGCCTATGGCCTTGTGGACTTAATCTACTCATGTACATGGTGCCCATTATAAGTCTACCCTAAGTGCACATGAAGTGTGCTATTAGTGACGGGGCTTTGGTGTGTCAGGGCTTCAATCCCTTTGCAGGTGTTTGAACATCAGGAATATCGCCAACAAGATCAGCTAACATGGGTACACTACTAAAATGAGACGCTACTGTATGCTTTCACCCTTAAAATAGGATTTGTAGAAATGTCAAAATACTAGCGTTGTTTCAGATTTAACAGTAGCAAGATGAATGTATTTAAGAAAGATTTGTATTACCTCATTTTTAAACTATTTTTC |
| 334 | lncHLMBl_002 | GTCTGATCCAAGCGTCTCCCCGGAGAGTCGTCAGTCTATAGCAATCGATGATTGGCTAAACAAGTGACATGTCTTGTGTGTTCTATAGTCTTTAGATCCGCTCACTTTAAAATCTCTGGTGTCAACACCTATTTGCTGACAAAATAAGCTGACTTTTATTATATAGACAAACAAAATGTCAAAATTATGGGAAGCTTTAGAAAGATGGATGAGTTTTCAGTTCTAC |
| 335 | lncHLMBl_003 | TGTGGATATGTGCTGAATGCATCTAATAGGAGGCAGTGCCATATATTTGTATTGAGAACAGAATCCTGAGGTTCTGATGTCCAAAACTAATTAGTCATCAGTTATTTTTTTTTAAATGTATTTTTGTACTGAGCCTCTAGACGAGAATATTTGAGTGTGTGTGAAATCTACATGCTTGATCAAAATGTGAAAGTGTCGACTCCTGCATTTACTTCAGTGGGACTCGCTCTGTGAATGAACTCTTTTCCCGCCATGCAGCATTTTTGATGATGTCACTATAACATGACGGCCCTCAGGTTATGTTCTGCTATAGGAATAAATGACCGTGTTATATATCACAATAAAGGATCAAGC |
| 336 | lncHLMBl_004 | GCTTACCTGTTTATTGGATGTATTTTTCGTATATCCATCTTTTGATATTGGGATACTTTAAAAGTTGTTTTAGTGACATTTAAACACTGAGGAGATCACGACCGCATCAGATTCATCCGCGGCAAACAGCGGGGCCGAACCACAGCTCATGTCATGGCGACAAAACAATGTTCCTCCGCGAGTTACTTGTAGATTTCAACCACTAGAGGGCCAAACGCAACAACCTGTAGCTATGCT |
| 337 | lncHLMBl_005 | CTTCACTTAGAACACAAATCTGCTTTGGGTCTTTATCACCCCATTCCATTGGCCAGTCAGTCAGAAGCACTTGTACCATGTTTACCACACTGTTGACCTCAGCATAATTTAACCATGATGTGGATGTGATGGTGGAACTCAGATGGCATTCTCCTCTGACATGATGAAAAATCAAGGGTTTTAGCCTCGGGTGTGGATGCACATCTCCTCTGGCCTTGATCCCATCACTCACATAGAAGTAAGTGGATACAAAATCCACAATTTCTGTGGTCGAG |
| 338 | lncHLMBl_006 | GGAGAGGGTGCTCTAGGCTAGTTTTTAACAGCAGACGTCGCTCTAGGCTAGCTTTTAACAGGAGAGGGTGCTCTAGGCTAGTTTTTAACAGCAGACGTCGCTCTTGGCTAGCTTTTTTGACAGCAGCTGGCGCTCTAGGATAGTTTTTAACAGGAGATTGCACTCTAGGCTAGTTTGTAACAGCAGATGTAGCTCTAGGCTAGTTTTCCTTACAGCAAATGGTGCACTAGGCTAGTTTTTAACAGGAGATGGAGCTCTAGGTTAGTTTTAACAGCAGACATCAATCTAGGCTAGTTTTTAACAGCAGACGTCGCTCTTGGCTAGCTTTTTTGACAGCAGCTGGCGCTCTAGGATAGTTTTTAACAGGAGATTGCACTCTAGGCTAGTTTGTAACAGCAGATGTAGCTCTAGGCTAGTTTTCCTTACAGCAAATGGTGCTCTAGGCTAGTTTCTAACAGGAGATTGTGCTCTAGGCTAGTTTTTAACAGCAGACATCAATCTAGGCTAGTTTTTAACAGCAGATGTAGCTCTAGGCTAGTTTTCCTTACAGCAAATGGTGCTCTAGGCTAGTTTTTAACAGGAGATGGTGCTCTAGGTTAGTTTTA |
| 339 | lncHLMBl_008 | GCACAATTGGCAGTCACAAAAACAAACGAAAAGACTGAGGAACACAAATGACAATACAATTGAGTGCATATACATAAACGTATGCTGAATTATTCTGTATTTAAAACACAAAAACTGCTTTATTTTCCATAGATTCTGCCAATTTTCATCTTAGATGTACAGTATTTGTGACCATGGTCTTTGTAGTGACCACAAGCGTACATTAATCATATCAGATTTATGTATCATCTAGAAGCATTTTTGGCTTGATTGATGGTTTGATAGGGTGGGATATTTGGCAGAGCTACAACAGTTTGAAAATATTTATCAAAAAATTA |
| 340 | lncHLMBl_009 | CTCTTCATATATACGCTTTTATTAGGAGACCCCCAATTAAAGGTGCAAAGGAATAAACTCTGAATCTGATATAATTAACGTGGCCTGATTAGGACACTAATATTTCGTTTCAACATTCAAAGGGCTCTTAATATTTACAGCACTTGGCCCCTGAATAGGAGTCGATTCAAATCTCTGAATATTAGACTTTAAATAACAGCGAATAATCTTATAGAAGAATAAACTTATACAAGGTGCAAATCAAGTAACAGCTTGTTACTTTCTGAATAATACATCATCTGTACATCACAGTTATGTTCGGTGACAAACAATCGGTCGAGTTATAAATACACAATCGAACAAAATGCATGGAGAAAACTTAGTTACAGCATTTTCGTTAGCCTGTGCTGGATCGACACCCCTTTAGATGTACGACAACAACAAAAAGAAAATAAAAAATGCATCTCCTTATTTACAGTTGCGATTCTCTTCACAGCTCAATTCCCAAAGGTAAAACACTGCAAGAGAGTGACACGAACAACACAACGTGACCGTCGACAGCTGGTCACTTCTGCTGCCGCTCGCTCACTCACTCGCAGTGCTTATGTTGTGTAACGTACCACATCAAGTACTTTCTTTCACTCTAAACCAA |
| 341 | lncHLMBl_010 | CCTCTCAACATCTTAAAGTCTTCCTTTGGCATCACTGTTATTCCTCAATTTTGTTTTTCGATAAATTCTTTTAAATTTCTGCAGAAGTGTTCAAACGTGCTGTAGTAGAAGGCCATTTTGTCAGACGCAGCATCTGAGATGTGAACATTTGATGTCTCAGTTTACCAGGATGTAGGGAACTTCCTCTCTTACTTAAAACTAGAATGTTTTCAACTACAGAGGGGGGATCAAATGGCAAGTACCTCACAGTCAAAAAGGTGCCTTAATGTCTGAGAAAGTAATA |
| 342 | lncHLMBl_011 | CAGCTCTGCTGTTAGCTATACATGTCACCATTGACCTGAGTGTGTGAGTGTGTGAGTGTGTGAGTGAGTGTGTGAGTGTGTGAGTGTGTGTGTGTGTGTGTGAGTGTGTGAGAGTGTGTGAATGTGTGCGTGTGTGTGTCTTACAGCAGGATGGTCTTTGCCGAGTGTTTTCTCCCTGATGGAGAGCGCATCATTCAGCAGGTGAGCCGCCTCCTTATACTTATTCTGATCCCNN |
| 343 | lncHLMBr_001 | CCAAAATTCATGAAAATATACGTATTACATCATCTATTAAAAATTGCTTGATATTTAACCACATATGATATTTTGGAGGGTTTTGAACAAACAGTGTCAGAATTTAAGCCAATTTTCCACCATTTTGCATTTCGCCCCCTAGTGGTAATCGTTTCAAGAAGCCATTACCACTTCTTTATCGAGGCTGAATTAATGATTTCAATGACAGTACTGATTGTATGATTATATGCACAAAAACAGGGATTGTCTAATATCCAGAAATTAAGGCAGCCATTAATATTAGAATTGTCATTTTACAGTTTTAAGAAACAAAACATTATTAAAAATATTGTTTTCCTGAAAGACTTGCTTTTTATTTTTCAGTGAATTTGGTACGGTACGATAAGT |
| 344 | lncHLMBr_002 | CGGAGCATTTACACTTCGCAGTGCGATTTCTGACGAGCAAGAAGAGAGAGAAGCGGAGATGTGAATCATCCGCTGTCTCTTTAAAGGCACTTAGCGAAGCTTCACGTATTCCGACAAACACGCCGTCGTCTCTGCCTTCAGCCGCAGCGCGCGCGGGCAGCGTTGAGGAATGGACGCCTGCAACTTACTCTCTTCTGGATAACGAACTCTTTTCTTGCTTGGATATGGTTTGTTTCTGGTGGATTGCGTGCTGAGGAAGGATCTTTGGGGATATATATAAGAGCGTTGACGCGGACCGCGTTGTCTCGGATGGTTTTGTGGCGTCTCTTCACGCTGAAACTTGCTGATAGAATAAGTTCCTATTTTTAAATTGCGCGACACACACGCGGAGGCTTATTTATGCTGTTTCTGACACAGTGGCTCATGGATTGGGTGGAAATTAAAGCTGGGCCGGCGACATGAGTCCAGAGCGCGGTCTGCAGTATGTACACAAGCTGATGTTATTGTATAGCGTTGCAACAAAGTTGCCGTGCG |
| 345 | lncHLMBr_003 | GCGCGCGGTGTCTGCTGGACGCGGCGGCCGCTCAGCGTTGGATCACAGAGACTCTGAGCAGCATCAGGACCGCTCGAGCAGTGCGGGCAGGAGCGGTGCTCGGACCCTGTGCCGGAGCTCACAGCATCGCCTTCATTGCCTGCAAGTGCTCGGACAGACGAGCCGCCGCCTGCGTGCTCCAGATCGACCCATCAGAATCCAGTGAATATCTACAGATGGTTCAGTCTGCAAGGAATTCGGAGGAACAAAATCTAGAGGCGTATATGAAGAACGGACATCTCTATTTTAGAGCCATA |
| 346 | lncHLMBr_004 | GCACCTTGTGTATATGCAATCTTGATATGTTTGGTCTTGGTGGCATACTGTAGATCTGCTACTGATTAAATTATTGGTGCAAAGCAAGTATTGAGACTTGCTACAGTCAGTATATTCACATTGTCAGAAAAAACAGTTTAACACTCTGCCATTGCAAACATAACATACAGTAAATATAACCGTCATTGCAGATCTAAGCATAGGTGGAGTTGGGGCTGAAGAGGGACAGAAAGTTGCAGATTGCTAGTGCAAATGACACTGAGGTACTCTAATGTTGAAGAGCAAGCTCATTT |
| 347 | lncHLMBr_005 | CATTGTCAATATAACACAAGTAAAGCAAAAATCAGTACATGTATTCTTTTCGTTTACAGTTTGAGGTGAAGTTCTACATGATCAGGTTATGTTTTGACAGATTTACTGAGACTCACTCGTCCTGAACATAACCTTCAATAAAAACAGAAGCATGGTCTGAGCTCTGGATCACAGCAGGTCTCCGTCAACACCAATGGAAATGCATGC |
| 348 | lncHLMBr_006 | GTGAGATGCTAATGGTCTAATGCGAGTTAATGATTTATGCTAAGCTAAGCTAACAGTGCCCCTGCAAGACCCGAAGATCAGCTGAAAGGATTCAGAAAGTGTAAAATGAGCCTATTTTTAAAGAAAGGTGGAGTGTTCTTTTAATGAAAGGTGATGATCCGTACATGTTGACTACCGTACATGAGTAGGAAGGATCAAGGTGTTTAAGGACTTGAATATAAAAAATAATGGACGAAGTACTAGAAACGCCTCATTTGTAGCATCTTAAACCTCCAGATGAACATGAGTGTCCATCATTATGTTGTTTATGGACATCAGTGACATGTTCTGCATGTAGTGAGACTCAGGAAAATACCGTTTATGATATGATAAAGCCAACTAGAGATAATAAATCTGAACAGTGTATATTTTGTGAAGTTCAGGTTTAATCTTTTTTGTAGCCATGACTTTGTGTCTGTACAGAATGTGATTTAATAAATCTTTTTCAAGAGTTCACACTTAGCTGATGATTGATTATAAAGCTT |
| 349 | lncHLMBr_007 | ATATATTTATTAGTTCTAGAAAAACGTCTTCATATATATTTTTATTTGTAGATATGCTGACGGTCCTGATGAAGATGTACAATCAGTCAATGAATATTCTATCGTTCTTCGTTTATTCCATGAAGTAAATGATTTGTAAATAGCAAATGATGCTCTTTATTATGCAGAAAAAAGCAATAATGTTTGTGCAGAGGCCTTGTTTATTTTTTGGTTAGATACATTTTAGTAGATTAAAATGTATTATTTTCATATATGT |
| 350 | lncHLMBr_008 | TATTTATATAGTGCTTATATCCATCCTGTCATGTTTCACGGCCTCTTAGAGCGTATCGCTCTTATAGACGGACTCATGGAAACAGTGGGAACATGGAAACGGCAGTAAATCTCTTCAGGGACTGCGGCACAATGAGTCCTGCGAGGAATTTCCTGCGATAAGCGTCTACTTAAGCCGTCACTGCGTTTATTTTGGCCCGGACCTCACTATTCTTGCGCCAATGTAAACCAGACACCTGCTGCCCGGGACAAAGCCGAGCGCCGCGTGTGGATTTCACAGCAAAACCGATCACTTTCAAGAACAACCGATGAATGTTGCACTACATAAAACTTACGCTTTGTGAAATATCAACTTGATCGGCATGAGAAGTGTTTGTCTTCTGTCAGGTTTGGAGTTTTTGTATTTTAAAGATGATTTATGTATACAGGCGAAG |
| 351 | lncHLMBr_009 | GTGAAATCCAAATATGAACTTGTATAATATATATGTAATTTGCATATATTTCCATGTATCAGATTTTTATGTGGGGTAGCTGCCTGTTAATAACTGAATTTCAAGCCTGTTTGTGAGTGAATTTATATATTCACGCAATTATACTCAATAGCGCCACTTGTGGCAAGCTTGTGAATGCAATGCAGGTAACTAAAGGGGGGAACTAAATCATTCACATACTTGCGTATGAGTACGTTCAGCCTTTCAAAAGAATTTTGCAATTGGTCTATCTCAATCCTGTCTGTTTTATACACCCCCGCCCCTAAAATACCATGATCCCTGTAATACAATCCTCTTCACACTTGTACTAACATCATAAGCGCTTTTGCCAGGACTTAAGATATAATGTTTCTACATCTTAACCCTTTCCTTTTGAACTCACAGTTGTCCCTACTTCTTTCCCTCTTGTCCCAGAGCCAACTCAGTCGATGACAGTGGTATATCTTTGGTAGGGAGAGGTTTGGGTTGGGGTAGAAGGAAGTAAGGGAATGGTTTCACCCCCCACTGTTTTTAAAATTAGCTTTATAGCATTCACTAAATCAGCGGACCTCTACTAAGGGTTTTATTTATAATTGGGCCCCAAGATGTTCTCATAAGTGTATTTTACTGCGTTTACAATTGGTGTACGCAAGTGGTATGCATTTTATAGTTCTATTTTTTCATATTTTGATTTGATTGAACTGTAATATAAATGTCCACTCAGCAAATTGTTCAAGTTTTTCAAACCCCCTAAATAAAAGTAACCTCAAACAATAAAAAATAT |
| 352 | lncHLMBr_010 | CTCGGGTACAAAATGCTAGTTTTGCGTCATTGTGTTGTTTTTAATTTCACTATAATGACTGCGATCGCTATATTCACCGTTTGATTAGATTACACGAGCTGGACTCACTGGTCAGATGAGCTAAAAACAGAGGACATCTGCTGGTTAGAACGGGTAATGCAGAAAGAAAAGCATAAAACAATTTTAAAAATTATTATTAATCAGCGTGGACACAAATATAGTTATTAGTATATTTATATAAACAATGGAGTATGTAGGATATTTGCTGGTTACAATGGGGAAATTCAAAAGAGGTATCAATTGCTAGTTTTGTGTCATTGTGTTGTTTTAATTCCACTACAATGACTGTGATTGCTATATTGACTGTTTGATTACATAGATTACTTGAGCTGGATTCACTGGTCAGATGAGCTAAAAACGGCACACACTGAGGACATCTGCTGGTTAAGAGGAGCAATGCAGAAAGAAACGAAAAAATATATCATTAAGCCGTGTGGACATAATTATGGTTATTAG |
| 353 | lncHLMBr_011 | AAAGTTATATCCACATTTATAAAGGTCATAAATACAGTGCTAATGAAGGAAATTTGACTCTCTATACTGAAGTGAAAGTAAAATCAACATTAATTATGGCACTTATTGAGGCATAATCACGTTTATTAAATAAAGCAAGCTAATAATTAAGGCTAAAACGTCATAAATACTGTGCTAATGAAGGACACTATTGTAAACTCAAAGTGATATCCACATTAGTTTTGGCACTTATTGTGGAATATTCAAGTTTATTGATTAAGAAAACCTAATAATTACGCCTTAAAGGTCATTAATACAGTGCTAATAAAAACAATTTGACCTTCTATTCTAAACTGAAAGTGATATCCATATTAATTATGGCACTTATTGGGGAATATTCTAGTTTATTAACTAAAACCAGCTAATAATTAAGCCTAAAAGGTCATTATTACAGTGCTAATGAAGGAAATTTGACCCCCTATTGTAAAGTGAAAGTGATATCCACATTAATTATGGCACTTATTGAGGGATATTCTAGTTTATTGAATAAAACAAGGTAATAATTAGGGCTGAAAGGTCATTAATACAGTGCTAATGAAGGAAATTTGCCCCCCTATTCTAAAGTGAAAATGATATCTACAATAATTATGACACTTATTGAGGAATATTCAAGTTTATTGACTAAAACAAGCTAATAATTTAGGCATAAAGGTCATTAATACTGTGTTAATAAAGTCAATTTGACCCCTATTCTAAAATGAAAGTGATATCCACATTAAATATGGCACTTATTGGGGAATATTCAAGTTTATTGATTACAACAAGCTAATAATTAAGGCTAAAAGTCATTATTACATGGTAATAAAGGCAGTTTGAGCTAAAGTGAAGGTGATATCCACATGAATTATGCCACTTATTGAGGAATATTCTAGTTTATTGACTAAAACTGTTTTTAACTAATGCTTATTTTTTAACATTAATAAAT |
| 354 | lncHLMBr_012 | AACTGACAAAATGTAAAATACTTACGTTTCCTCGTGAGATCAGGCTGGGATGTCCAAACTAGGTCCCGGAGGGCCAGTGTCCTGCATAGTTTAGCTCCAACTTCCTTCAACACTTTTCTAGTATACCTAAAAAGAGCTTGATTAGCTGGTTCAGGAGTGTTTCATTTGTATTTGAACTCACATATTCAGGACACCAGTCCTCCAAGAGTTTGGACACCCCTCTTTTATAAACTCTTTGGTTTCCTCCAAGCGTGTAAAGCTGTGTTTTGGGCTTGTTCAGTGAAGTTTCTCACACACACTACTGTATTCACCATAGGATTTCTCGCTGTACCTCATGCCTGTAATGTTTGCTGCTTCCATATTTTTGGTATATTGATGATTTTGCGTCTATTAGCGACATATCATAGTGTACATAGTAGAACTATTTATTGAGAGCGTTATACTTTTGTAAGTTATATATTTTTTATTGTTTCTGTAAGTTATTTGTACAATGGTCTTA |
| 355 | lncHLMBr_013 | TGAGGAGTCGCAGCAACTCTGGAGTTAAACTTGACAACTACGCGCGGATAGTTCATCAGACTATTCTGCGTCATCAAGATCCAGTGACTGGTCTGTTGCCAGCAAGCAAGGAGCAGCCTGATGCCTGGGTGAGAGACAATGTTTACAGCATCCTGTCGGTCTGGGCTCTTAGTCTGGCTTACAGGAAAAATGCAGATCGTG |
| 356 | lncHLMBr_014 | TATATTCTTTAGCAAATTCTCTAGTAATTCTGCAGTCTAAAGTGGATGAAAACAGCATAGTGTGTTGATTTTTAAATCTTTCTCCAAACAGCAGTTGAAGTCAAAAAGTTTGAAGAAGTATAAAACTATTCCACAGAAAGAGAAGAAGGCATTTCTGAGCTAACTCTTTGTGAAAATAAAAAGCAGAGTAAACATGAAGAGCAAATCCTCCTCCGTTTACATCAACACAGAACTGAAGGCTCTCTCGCTCCCCCTGCTGGCTCCTCAGTCCTAACACATTTGTGGTTATTGTTGAACTGAGTAATATAAATATATGTATATGAAAAAAACAAACTGTCAGCTCAGTACACTTTGGTCCTAAAGGGGACTCTCTCTCTCCAGGGGTCGTGTGTTTGCGTGTGTGTGAGTATAGTGCTCAGCATAAATGAATACACGCCTCATATATCTGTCTTATTTTAATGAATATTTTCTATAGGATGCTTTACAATAATATATTTGTGCATATACATTAGATTAATCAGCACCAAAGCTACAACTGGAACTTATCTACACTTTAAAAATGCTGAGTTCCACACAAGCTGAATTGTTTTGGGACAACATGAAGGAATTAAGTTAACTTATTAGTTTTTACAAATTTAAGTGGATTAAACATAAAATAAACAAGTTGTCCCAAATAAAACCTCAAGAATTGTGTTGTTTTGGTTCATTTTAAGTAAGTAGTTTGAACAAACAACAAACTTCCACTTATTTGAGTGTAAAAAACACAATTTCAGATCATTGTCCAAAAATAGTTCACAGAAGTTAATATATTGGGTGTTTATAATATGTATAAATGATATACTCTACCTGACAAAAGTCTTATTGACTATCTAAGTTTTAGGAACAACAAATAGTAACTTGACTTCAAGTTGATTATTTGGTATCAGAAGTGGCTTATATAAAAGGCA |
| 357 | lncHLMBr_015 | GGAAAATCGACGTTCTGTGTGATCATATTGTTAAAGTTAGTTTTAGCGTTTAGTTATTATCATAACAGAGAGATCAGAATGCACTACAACCGAAGCGATCTGTCTGCGTGCAGATCCTCTGCTGTTTCTGATTCACCAAATGCTGGTGATTTCTATCTTTGATCCAGGGCCCTGAGGACTATAGGCTACACAAGCCATTAAACTGTATGGTACTCTCTCCTCTTAAAACTTGAAAACACTTATATATCGCTCATAATCATAATGTTCAGATGATTCAATGTTTCTTTTTATTTTGAATATCTCCCTTGCTCTTTCAATACATGAAACCAAAATAGGAAAAGGTTGTTTTGCGAGGCATCTTACTTAAGGCATTTTTCGGGGGTCCCTAATGTTACACGTGATGGAAATTTGTGATTGATTTGCACTTTACACTGAAC |
| 358 | lncHLMBr_016 | CGTGCGCAAGGAAGGAGAGAAATCAAACAATTCCTGTCGTATATGCATTTTGTTCTTGTTTTTTGATTGTTTGTTTTGAGAGCACTGCTGTGGCCGTCACTCGCCGGAGTCTGTGAGTCGTGTGTCCGCCAATGCACGAGCACACAGGAATGCGGTGATGCTGAGAGGCCGGTGTGAAGAACGCGCGCGTTGTCACAGCGACAGAGCAGCTTGGATACAGAGCGTCACTGCAACGTGAACGCGCATTAGAAAGGGAGAGAGAGAGAGCGCGACGGGCAATCAAAAATCTCACAGCATCTCTCCCATCGCAAAGGCTCCGGGAATCACGGAAAAACGACAGCGGAGCCGGTGAACACCACGACCGCCTTCAGTAGCTGGAATCAGACGCCCGAGATGCCGTTAGGTTAAACTCGAGACTCGGCTGCTAAAGTCGGTGCGTTAAATTGGCATTTGTTGCCGGAAATCCGAAATTATATCACGTCAAATATGTTAAAACGGAAAGAGAGTGTTGATTAGACGCACAGAGCGGGGAAACAGGTCATAGAAGTGAATTTGTGTTTGTTGTTTCATCTTTTCACCATATGCGCTATGATGGGAGAAGCTGGCTGACGTTTCCAAACAAGCTTTTTTAACGGACACATTCAACTAACGTTAGACTTCATTTACGCAGACGTTCCGTTGGTCGAGCAACGCGCGCGCACAATTCTTCACTTTTGAGGTTTCGTCTGAATGTGTCTCGTGTCGGGGTGTTGTCGGGAGAAACCGTGAAACATTTGCTGCTGTTCTCGGACAACGGAGAATCTTGTGGTTTATTCCGAGCCCAGGCTGATGGAATTGCGCTGACCAGCGACTAGGGAAAACGAGGAAATACCCATAGATCTCCAGTAGCCTACACGTAGACCTCGGATTTCTTCCTGCGGCTCATTGCCAGGT |
| 359 | lncHLMBr_017 | GCAAGAGCCTCGGAGCAGACACTTCAAGGGCACGTTTATTTTCAAGAGAAAATATCAGATACAGCGTTTACTTGATTAACAAAACCGACTATCATAAATCAGTAATACGGATTCTACTCAATACATTGATTTGAAGGTCTAGTTGTTTGGTTTCTAATTTCTGACAATGCATGTTAATTATCGCATTAATATATACGTAAACAAGAAAACACACAGTATAAGCCGGATTCCCT |
| 360 | lncHLMBrBl_001 | CCAAGTGTCAACGTCAGAGTGAGGCTATTGTCACTGACTTCTCAGAGCAGGGTCATCTTTTTACCATCCCATTTCTGAACATGCAACAACTTCCCTCCATCTAAAGTTATAATAGACTTGACTTTTCGGTAGTCTGCAGTGGTCTCGTTGACCTACTCTCCCAGTTTGAAATTGATTTCTGTGGATTTAAAAGTGCTGGCGGTTTTAATTGTGAAAACGTCACCCTCCTTGGAGATGATGGTTGTGGGTT |
| 361 | lncHLMBrBl_002 | GCCAAATACAGCAAAATCTAACAATGTGTTTCAACTATATTATATAGGGATAACCATATTTGTGCACATTGCACACTTGCAAATAGTTTATTAATCTACAAATCAATTTGTCGGCTCTGAAACGAGAATCAGAAAGATTAGGGAGGAGAAAATGTTTGGGGAGATGCAGGGAACACTGTTCTATACCCTTAGATTGTTTTTGATCATTTACATTGTTTGATTGTCACTCGTGTGAATAAACTCAGACTTATGCGGTTTGAACTCGCCATAAGGCAATGACAGTGACCGACTGATGCAGACTCCCTAAAGCCCTGTTCAGACTAGTTATGACAGATCATGGGTCTGTTCTTTGTGCCTCGCTTAAATGATTTAAGATGATTTGGCAGATCCTGGATATTTTAATCTTGATAACTGATCTCTGGCTAATAAGGTTCTTCAAACAAGTTTGTGAATCAGATTAAAATGTCTGGATGAACTGATCTAAAATCGCTGTGTGTGTTGTGAAGGACAGATCTATCGTTTCCAAAAATCATGATCAGCAATGCAGTGATTGGCTGTTGGCACAGCAGCGTAATGACATCATTAAATTAATATTCGATTATCCATGAATTACATCGAATTCATTGGAAACGGTTTGTAAAATATGATACGCATCTTTACAACTTTGTT |
| 362 | lncHLMBrBl_003 | TGGTAATGAAAAAAAGACAAAGATACAAATAAATAAAAAAACAATTTGGTTATTAAAAATACAGTATTTAGGTCAGCAAACTGCACAGTATAGTGTGGAGACAGGCAGTAGAGAGGTCATACAAACATTAAAGTAGTGCAGGACGGATATTACAGCAGTCAACTAGGTGCTAAGTTTGCTAATTCAGGACAGGATCCTACATTCAAAGACTATTTAATTTAAACAAAAAATGGCAGTATTTAACTCCAGTTCACATACTTGTGCATTTAGTGTATAGTATATGTCACATCGCTGTGCATTTACAAAAACATGAACAGTAAAATATGCCTGAAAGTTGGATTTGGTGAAAACTCATGTCAGTCAGTGCAGAAATGTTAGTGTGCTATATTCGTTAGTGCGATGTGGGATGTTTTCCAGTTATTTACTACAGTGAATAATGCCTTCTAAATGGATACACGTCCAAAAAATTCATCTGTCAAACGTTTAAGATCAGTCAGCTTTCTTTTGATGTTTTCTTAAAGCATGTTTATTACCCAAGACTGCATTTATTTGATCATAAATACTGGAAAACACTTTAAATAGTC |
| 363 | lncHLMBrBl_004 | GAGGAGGAAGGGTGAGGCTGTGCTGCAAACCCATCTCGCAGTTCACCGACCACACACACATTTTACACTCACACCCGGACTGGGGACACGCCGAGAAGGGCGAGCTGGACTCTCTGCCCTAGTTCAGCCATTTATTACCAGCATTATCCACTATTTGACTCTTTTATGCAAGAGAATCGAGCGTTTTTGTACTTTTCAACCAGCTGTTGATTCAAAGTGGACCACGGAAACAAAGCAGACACGGCAACCGGACTGCTGGACCAAACGCGACTTTCCCTGGCAAATGTGGTTTTATTTGTGATTTTCTTGGATGAATCACGCGTGTTAAGGT |
| 364 | lncHLMBrBl_006 | TGACATTTGAAATGTCTAAGCCATTGAAATCTCTTCCCACATAAGGAGCACAAATGAGGTCGGACACCTGCGTGACTTTTCATGTGCGTCTGTAAGTGTGAAGCCAAACTAAACGTTTCATTACACTTGCCACAATCAAACGAGCTTCCCTCCGAGTGATCGAGCAGGTTATTGTTGAACCCGCGCTGTTTTCCGAGCCTCTTCCCAGACTCAGAAGACTGCAGTTTCTTTGCAGAATGCCTTTTCAGATGACACTTCAGATCTTTCCGATGTGTG |
| 365 | lncHLMBrBl_007 | CAGGTTTGTCTCGAAGAAGGTCCAGTACAAGCTGAGCATAGAGATGCCAGAGGTCTGCAACGGAAGTGACTGCTCTTCATAGGAAAAACAAGTCACGCACACAGACACACACACTCACACTTCTGTCTTTACCAGGTCACAGGACTGCTGGTCCAGGGTTACGGCTGCCTAAAAGCCAAAAATGTGATGCTGCTTTCAGTGAGTTGTTTGGTGAATCTCACTAAATCTGACCAGAACTGGATCCCAGA |
| 366 | lncHLMBrBl_008 | CACGATCTGCTCCCCAACTCCGTTCTCTATTTTTGTGCTTCCTTGTGCTGCCAAGCCCCTGACGTAATGCTCCGTGTTAATTATTAGCATGTGGGAAGCGATGCCGAGACTCTCCTCTGCTGCTTAAACAAGAGCGACTGACTGCGGGGAGGAAAACCTCGTCAAGATGGCCAAACACTCTGACCCAGAGTGAAGGGGCACCGCTGCTCCCTGTCCCGTCCCGTCCAGTCCTGTCCTACGACCATCACCAATGCAACTGCG |
| 367 | lncHLMBrBl_009 | AAAAAATTTAGACTGAAAATTCTAATGAAAGTCTATGAGAAGGTGCATTTCTTAGCACAACACTGGCAAACTCGCAAACTGGCGACCAGGTGGGCATGTTGAGCGATGCAACAAAGTTGAGAATCCTTGAACTTTATGCAAATGAAGAGTGACTTTCTTGAGCGACAGCCAATAGGAGCACAAGCAGAGCTCCCGTAATCTTCTCTCAGATCCTGCAGAGGCCAGTTGTAGTCTCTGTGCTGTATACCTACACAGACCTGCGTTTGCAGCAAATCACCACCCAGAGCGACAGGCAGCTACAAAGCTGCGGCTAGTGTGAATGAGGCATGCACTATAAGTCAAATACTAGTGTCTTGTAAAATAGTATGTACTTCATCTCAAGGACAAAATAAATTAGTTATTAAAAATAAA |
| 368 | lncHLMBrBl_010 | CGCAAACCGCTTCTTCAGCGTCCGACGAAGGGATTTAGAGTTATACTGCGGTCCATTCGACAAGTCAAACTGTTCAATTTGAGGAGTTTTCCACACAAAATGTTTATTGTCCGACGGCGTCTCCTCTGCGCGTGCACGGCAGTGTCATTGATTTCATCTGGACAAATCGGTCTAATGGATACTGTATGATCTGAAAGAAAGGTGCTTGTTTTATAATGTGTCCTGGAGTCTATTGAATAACTGGCTGACGTGGGGATCACGCAGGGGGGCTGGCCGAAAAAGGAGAGCCACGGACGGGCAGAGGGGCTGCGCCGCCGTGCGTCTGGGATTACAGTAAACACAGCTGGTTTCTTCAATAACCTGATAGGCTCTGATGAGAAAATGCAATCACTAGCTTACAAAACGCGAAGGAAACTATGTAGCAAATATTCA |
| 369 | lncHLMBrBl_011 | CGAAAAGCAGGAAGTGCTGAATCTTCATTGACAGCAGTGCAGATCTCTGTTTACCTCTGTTTTTATGACTCTCGTATGCAAAAACTTAATAACAGGCAGCTTATTCTTCCTCACGGAACCGTTATCTGCACCGTAACCCGCCCCCTGGGGAACATATGATTCAGGTTTGAGAAGCTGCGGAGAAGATGAGTCGAAGCAGGAACCCTCCGCAGAAGGGCCAAGGGGCCGCCAGGGCCAAACAGGT |
| 370 | lncHLMBrBl_012 | TTTTGATTGGATATTTTTGTTAACTAGACTGTTTTCATATTGTATTTTTCTAACATTACTTCCTGTTCATGTTAGTAGTACAAATTTAGAAAGTGGTGCAATGCACTGAAACTATTATAAATATATGAATATAAATCTCTATAGAGTGCAGAATGTGCTATATTCTTGGACCAGCCATTGCAGCTCCTGTTAGTAGAAACAGCAGATATTGTTTTGTTCCATGTTCCTAAAGATGTTTAATAAGATGTTTCTGAGCAAATC |
| 371 | lncHLMBrBl_013 | CTAATAATTCTGACATCGACTGTATTTAAATGAGCAGAGAACATAATGCTAAATAAAACACTGGCAAACAAAAATTGCCTACTCGATATTGCAGATTCCCTCTTCACAATCTAAACAGCTTGAACAGAGACAGAGGCACCAAGAGCTTCATCACAGTCTGATCCGTGCATGTTTTACGCTTTTGGTTTTCACCTTTGAGAACTCACTGAATATATTAAGTCATAATTGAGATGATTCTTTAGGACATTTTTAGAAGTTATCTCTTATAAAAAAGGAAAAATACACCGAAGGGTGCGTACTCTTCTACCTTTCTGGTAGCTCTGTTTAGGTAATATCAGTTATGGCACACGGTGCCTAGACACTAAACGCACGGTACTGCAAATCACGCAGAACCATAAATCATAACACTTCAGACATGTGTAGAAATTAGAGAACTTTTCATGCTATTATTAAATATGATCTTTT |
| 372 | lncHLMBrBl_014 | ATTTTCAAAGACTTTTTAATGTTTGGATGAATCAGTACACAGACCCAAGATTGTCGTTTGCTTGTGTGCTATTAAACCCATTGCTTTGGTTTAAATCCCTTCACTGAAGACTGATGCTGAATTCCACATAAAAACACAAACAGAAGCAGAAATGTTTTTTAAAAAAGTTGAGATATTCCTCTTGGATTTGCTTAAATCAAATAAAAATAAAGTGCCTTATATAAGCTATTCATGAGCTCAGCGCTCAGTCTTCAGCACTGGGTTTAGTGTAAGTCTGCATTATGAAATCATCCTCTATTTTTTTATGCATAACAGTGGTTGAGATCCATTAGGCACCGCTGAATTAAAGTCCCTTTTCATTAGATATGTACAGTTTGCATCTTACGGAATTGACTACATGGCTCTTGGCTAAAGTTAAAGATTAGATAAAGTGCAGTCGGCATTTGAGAAAAACTAATACAAATAGAACGAGATAGGAGCCGATTGCATGATGAACTGCACTGACCCAAACTGGAATGTAAATTTTCTAGGTTTTATTCGTCTACTGTTGCTCCATTACTGGAATAAAGTATAAAGTGTCTCAATAAAAGATAAAAATGAAAGCTTTTATTACAC |
| 373 | lncHLMBrBl_015 | CATACAATTGACGTTGGAGTAAAGATTACAGGTGTCTTAAAACATTTTCATATTTACAATCTACATCACCGACTGACTAGTTTCAACACCTGTTGTAACTATTTCTCCTCATCTCTATGGCCCACACATAATATATAAAACATACAGAACAGCTTTTTCATGACACAGATTGACTGAGGAGATTGTCAGAATCTGACATTAGATATTAAAAATAACAAATATGTCCCATCTTGGAGCAGTGCAGTAGGAAGTGATCATATGATTACCAACATCACATTTCCTCATAAAAAAGACTGACAATATAAAAATATTCACATAAGACTCATTCGATCTGTTTCACTGAGAATGCACTGGGTTTCAAAACTGTTTACACCACATACAGCAGTTTCATATAAAACAATCAATGTTGTGGTTAT |
| 374 | lncHLMBrBl_016 | CTCACCTCCAGTGCTGACCTGCATCCATCCTGAGGGCTCTACGTCAGTCAAAACTCATCAATCTGACTTGCATCCTCATCTTGAGCCCCATCTCTTCAGGCTTTTATTTGGAGTGGACGCCGCTTTAGTCAGTTCCCCGGTTATTCTGTGTGGTTTACCTGATGGACGTATTTGCTGCTTTCCTTTGCTTGTCCCGACTCTGACTGGTCCAAGAGGAGAGCAGAGATCTCCAATCAGAGTGCTTCAGAGCTTAGAGCAGCCAATCGTATTCATCGGAACTTGCATCTCTGGAGAACATGGACCTCAGAGTCTCATTGTAGTTGGTCAGAGAGG |
| 375 | lncHLMBrBl_017 | AAATGACAGGTTTAAATACTCAGCAAGACTAAAGCTCTAAATATTAAAGGCATTTTCTGCATTGTAATCTTGATACACAGGTTCAATGTTGCTGGGCAATGTTGCTTGGTTATTTTTCTATTAAGATTGGGCAACAGTATTGTATCTAATGTAGCCCTAATTAGTTGCTCTGTGTCTCACCCGGTTGCCCTTTTATGGCATCATTGTTCAAAACAACATTGCTGAGTAATATCTAATTTTTACATGTTAAAGAGACTGTTTACCCAAAAATATACAAGCTTAAATATTACATCAGTATGAACTAATGATGAGTTAAGTAATGATGACTGACTAATTAAGAACTGAACCTAAGGGGATAGTTCACCCCAAAACAAACACATTTAAACTCTCCTTTCATAATTCTAAATCGGTTCAATTTATTTCTTCTGTTGGACACTAAAAAAGATTTTTTGAAGAATGTTCGAAACCGG |
| 376 | lncHLMBrBl_018 | TTTCTTCAAAATATTTTCTTTTGGTATTTAAAGAAAGAAACTCAGAAATGTTTGGAACCACTTAAAGGTGATTACATTTTAATTTGAACCCTTTAGGAGTTACTTGCAAATAAAACTGATGTCCAGATTGTAAGTTGAATCATTAATGATTGAGTAAATCTGATAAAACATTGTTTTTCTGGTACCTGGGGATCTGAATCGTGTGCTGATCCACTATGAACATAATATTGCTACTTTAGGTTTTGTAATCATTCAGAATGACCTTTACTACCAATTATAGTTTAATTACGGCTTGTAAAAGGCATAAATATCATATAACACCTTTTTCTGAATTGTAACAGTACATTGTCAATAAAAT |
| 377 | lncHLMBrBl_019 | CAAGAATGTTGAAAAACTACAAACATGGCCGATTACGAAGACTGATGGTGACGTAAAGAGTCTTTTGTTAGGATGTTTGAATGATTCTTAATTATTAATATCTAATCCTCTAAAATATATTTATTTTAACATTTATATTTCATCGCCAACAACTTCTGAACTTCTGTTGGACGCGAATTCGATTAGAACTACAAATACAGGTTAAACTGTGTTAAAAAACTACAAACATGGCCGATTACACAAGATCGATGCTTAAGTAAAGAGTCTTTTGTTAGGATGTTGGAATGATTCTTACTTATTAATATCTAATCTTCTGAAATATATTTATTTTAACATTTATATTTAATCTCCAACAATGTCTGAACTTTTGATGAACGTGATTTCGATTAGAACTACAAACACAGGATAAACAGTGTTGAGAAACTACAAACAAGGCCGATCACACAAGACGGATGGTTAAGTGGAGAGAGTCTTTGGTATAGATGTTTGAATGGATCTTAATTAATATCTGACCTATGTATTAATATAATATAATATGAATATATATATTTATTTTAATCTCCAACAACCTCTGAATGCATAATTATGCAAATTTGAGATTGTTCTGCATCTCCAATATGATACAGTAGGACACTAAA |
| 378 | lncHLMBrBl_020 | TATTCATGAAGGACAAGTTTAATCATACCTGCCAACATTTGTCTTGAGGGATGGGCGGGGCTGGTGGTTGTGTGTTTGTCTCAATAACATAGTTGAGAACCGGGTTGGTAACTACTGTAGTGTTAGTGAACGGGTTTCGGGCGGCTGCAGCGATCGGATACTATACCGAAGTTGGCAACCCGGAGGTGTTACAGACTACCAAAAGGCTGAGAACTAAATTACGGGAGTTTTCCGGTAGAAATAACAAAATGGGAGGGTGGTGGGAGATGGGTCTGAAATATTGGAGACTCCTGGGAAAAATGGGAATGTTGGCAAGAATGCGTTCAATTGTTG |
| 379 | lncHLMBrBl_021 | CAAAATGGCTGCGCCCTTGCGCAAGTGTTAAAAAAACACTTATCTCGCCTGAAAGTCAAATTAACATCTTAGGTTTCACTGTGTTGATCATCATCGAGGACGACAGACAGTGCACACAAATAAATCAGTGGATTAAGGTATTTCGTGCCTGCCCTGATTCAAGCAGATTGCATTTTAAGGGACATGACGATGGGCCGCTTCGCCTAACCCACATCAAAACGCAGACCAGCGCAGATACCCCTGTCACCCTGCGCCTGCAGGCCCTGTACAGACTGATTTGTGTAATCACCCGCAGAGGCAACGGATCTACAGCTCGAGCTTTGGAGGACTGTTTATTTTGGAGGTAGCGTTACTCGTACAATTTACAAAACCCCTCAGCATTTCGACATTGTGCAAAATACCAAAACTTGTTCTTTGACACTGTGGTCTCGGTCCCCTAAATGTAAGTTATAGTGTGTGTGTGTGTGTGTGTGTGTGTTTGTGTGTGTGTGTGTGTGTGTGTGTGTGTGTGTGTGTGTGTGTGTGTGTGTGTGTGTGTGTTGTAGTTGTAACGTCAGTTTCTGTGTACGTATAGTATAGTATAGTATTGTCTAGTCTAGTCTATGTCTTTCTATCTATTACGTGTGTGTGTCACAGGTGCATACCATTACTTTAGGTCACAATTTACTGTAAAAGCAAACCATTAACCAACATTACTAGCTTGATAAACTACTAATTAGCTGTTTGTTAATAGTTAAGGTAGAAGTTAGGTTTAGGTTTTGTGTAGGATTAGGA |
| 380 | lncHLMBrBl_022 | CTTTATTCTTGTATGAAATACCCCGCATATGGCACATATACTTACTGTATGCATACTGTATATGCACTTCATTCTGCCATATCATAATTCATTTAGGTCAGTAGGCGGGCTTCTGCAATAACTTAGAACAAAAGGTTTATTTGAATTTTGGAGACTAGGTCAAGTCTTTTTGGGTGCTACAAGTTTCTTCGTTATTAGACTTGCAGTTTGCACACAGCACGCAATTGAGTGATAGAGTCACATTACATTTTTAGTACTTATTATAACTATAGCTATCTTAAATTTTCACATTGTGGAAATGCTTTCATATTGAACCTCATACCTGCTTGATCAGTTCCTATGCAGTCAGCCTATTAGTGCTTAAATTGAAATTTTTAAATGGTAATTTCTTAGGAGAGATGAATTGTAACTTTGTTGTAAAAATTGTC |
| 381 | lncHLMBrBl_023 | GTAGATGTCGCTGTGCTCTACCCCGCGTGAACTTAAGCACGTTGCTCCTCCAGATTCCCACAATGCCTCGTGTTTACCTTCTCTTTGACAGCGCCGATGCTCCTTACTGGCTTGTCTAGTTACCGACTGGAACTCAGCGTGATTCCAGCAGTTCTACATCCTCTGGACCATCACCAAACAGCAGGATAAATCCAACACACTCCTCCAGGCGAGCACCGGAGACTGTCGCCGTCCTGGACTGTGTTTATGTCCGCGGCGGTGGAGCTCGACACTTCCTCGG |
| 382 | lncHLMBrBl_024 | TTCAAAGTACATCCATGTATGCATTTTTGCTGCAAGTATTTCCTAAGCCAGTTAGCAAAGCAGCTAAACTCTCAAACTACCACAAATCACCGTTATTTACTTAAATGAACAGATATTTTGTGCTCTGAGTGTTTGCTTGGATCGACAACATGTCCACAAAACAATTGTGTTACACTGCAACGGCTTTTTTAGTATAATACTCTCAATTATTTCATTTTAATTAGTAATTTTGTCATCAATTAAACTACAGGGTGGGCCATTTATATGGATACACCTTAATAAAATGGGAATGGTTGG |
| 383 | lncHLMBrBl_026 | CACAATTGCACACATTTAACCAACTTGACAACCTTAACTCAGTTATTTCGAGTTAGTATTAAAACAAGCATTTTTACACATATATTTGCTCGGAAATTAATTAAGTGTAAATTTGAACTCAAACAAGTTACAAACTCCAGCTTTAAGCAGTAATATGCACTATATCGCACACATTTAAAATGTAACGCTCTTCACTATCAACAAAGTTCATACTAGTTTGAACCTGCTCTTCTGTATCAGGACATAACAATAACAAATACTATTTTGCTCTTGGCAGGCCTTAGAACGTCACAACGTCACATGACCTCTCACACCGTGTGATTATTTATTGCACAAAAATAAAGGTGAGATAAAAAAGCCATCTATGATTCAGATCCTGTACATGTACTTTCGATTACATTAGCTTTAGTTATACAGCAGATGCTTAAAACTGACGAGGCATTCAGTGATTCAACACGAAGAGGCAATACACACAAAAAGTGCAAGATATGAAGAATAACTGTGCTCAGAGAATTGGGTGCTAGAGTAAAGGGAGTTTTTTAAAGAGAGACAGAGAAGACTGTTTCTACAAGTAGTCTCTCAAGCCCACTCACTTCATAAATGCAGTGTTTTTATATGTGTGTGTGAGTGTCTGTTTATTGTAAAATATACGTGTATTCCTACAATCAACACACACACACACACACACACACACACACACACACACACACACACACAAAACCCC |
| 384 | lncHLMBrBl_027 | GAGAGAGAGTTTGGAGCTGCGGTTAGAAAAACAAAGAGAAACAGAAGTTCTCAAAGCAGCATCAATTATCCAAGCTCATATACTGGGCTACAGAGCACGGAGGCAGTACAGACGCCTGTTGCTGTGCATTGTTGTCATTCAGAAAAATTACCGTGCTCTATATTGGCGCCGACGCTTTCAGACAATGCGATGGGCCACCGTCACCTTACAGAAGAGGCTCAGAGGTCAAAGGGCACGAAGGCTCTATGTTCACCTTTTAGAGGAGAAG |
| 385 | lncHLMBrBl_028 | GGTTGAAAATGTGTACTGTTGGTGTGCCTTTGGGAACAGGGTTGGGAAACCCTTCTATAAAGGACACGTTATAAAGCTATTTATTAGCATAGGACTGTCTCTGTTTTTGTTGTGATCAGTTTCGCTGAATGTACCTTGCAAAAAAAGTAAGAAGCAGCAGGGATCAATGACATATCTCCAAGGAAGAAATTGTAAATCTTGATATGCAAACTCTACATAAACATTGCACAGAATCTCATGTATGATGGCGGGTGAGGGTTTAAAACAAGCACAACTTGTTTACTGAGACGTCAGGGCAGATTTCTCAGTCCAGACTTGTTTGTATTGAGCACTTCTGGTGTTGCACCCATGTGTCCTTTACCCTACCAACAGCACTGAGTACCACTTTAAAAAAAAACAAGAACGATGCATTTACTCCCGAACAAGTCAGTCTATATGCATGTACAAATGATGAAAATCAATTAAACTTTTTATCTG |
| 386 | lncHLMBrBl_029 | ACCGTCGCCTTCATCGCTGTGTCTCCGGCGGGACATTTCTGCAGTTCAGCGAACACTGAGGAGGGAAACACTGAGGGAAATGCGCTGATATATATAAAGCAAAGTCGCCTTCCGTGGAGCTCGGAGCTCTGAATGGAGCAACCGTTAAACACACGGTACAAATAAAGCGGTTTGATTTAAGGAGAACACATCCGGCGTCGACGGAAACACGTGTGATTCGCGGGC |
| 387 | lncHLMBrBl_030 | GTGATCATTCTGAAGTTTGTGAGTTTTGCTATTGCTATATGATAGTTTGTAAAACGGCTTGACTGTGTTCATAGAAAAATGTTAGTTGTGCTCAGCAAGTCCTCCGGAAAAAGCGAATGACGTTGACTGTGAATGTGATTGTTTATGTGAAAGTCACATATTTTTCCATAGTGTAATGTCGATCTGATGTTTTGGTGAGAGTGTTCATGCAACACAATGAATGAGTCAAAGTGAAAGGTAACGTGGTGTAGCAGGGTGAAGAAAACAGAGTAAAAGAAAGTGCTGTAATAGCAAGGCAATTGGACTGTATTTTTTATTGACAATGAAAAGTTGTTTGATC |
| 388 | lncHLMBrBl_031 | ACAACATTCTCGCTTTTATTCGGATAAGCAACTCCTCCACCTTTCACGGGCATCCTGATTCCTTTGTGTTTAAGGGAAGAAAAAAACTACTTCGGCGCTGGAATAATAGCGATTATTTGTGATCTGTCGCTGTCGTCACCCTCTAGGATTCAGCATGGCTTTGAGCAAGAGATCCTTGTGAAATCGTCCCTGGTGGAGCGATCAGCGTCTGAGCACGAGAAGGCAGACCCTGGGAAACATTTTTGCTCCGAGCGACTTTCAACCCAAGCC |
| 389 | lncHLMBrBl_032 | CTGCTGTCCTGCTGCTCAGATGTGCAGAGTTTCCTTTTGTTTTTGCTCTCGGTTTTCTTCATTCTGATGGATGGCGGCTGAGCGCTACTCTAATGTTAATATTTTGTTGAATCCAGTGGTCAGTGCATTCAGATACTGAAACAAATCTCGCCCTGGTACATGGTGAGCAAACCTCTCCTCAACTTTGGTCCACAAATACAGAATAAATGATGAACGCAGGTTTGCTTTTGTGTTGCACTCTCGACCTTAAGTTACAACCCCATCTGCCATGACAGCCAGTCTGTTTTTCTTCTCTTTTTCCGTCAAATACTGCTTTGTTTTCAGTTTTATGTAAACTATGCGGTTTTAGCGCCGAAAATCACCCGGCGGAACACGCTAGCTCGCTAGCAGTTTGTTGATAAAGTTTGACAACATCCGCAAAATAAACGCCTTTTGAGTTTACCAAAATTGAGAAAGTGATCGTTTAAAAGCAATGTGTAAATGAACTCGCCGGGCTCGAGTCTGTCACCGTGAGAGTGTAACGACAGGTTATCATTATGCTGAATAATGCCGCGTTTCCAACGTGTTTGCTGCCGTTTGAAGTCTTCCAGCGGATTGTCTCGTCTTGGTGAACCAATACTTTAGCATTCCAGTTATAACAAAACAAAGAGCTAACGTATAAAAACAGAAAATTTCCTCATCCACTAAACTGCCAGCAGACTGGAGATACACTTCGCCGCAGAGCCACAACACAGCGTTTAACTGTCCATCATGTCTCGTGAGTCTAGAGAGCGAGCTCTGTTTTGACGCGGATCATTATATCTAGCGGTCGCGAAGCTCCGCGTTTCTCCTTCAGTCTCGCTTTTCTTCAGACGTCAGCCCGGCCGGGTGCGGGGGAAACGGCTCGGTGCCGAAAACAATCGCTAATCAGACCCGGTGTTCT |
| 390 | lncHLMBrBl_033 | TAAGCATTAAAACATATAGTTTTGACATGTAAGCAACAGCAATGTTTGCACATTTCATTGGTTAACATCCAAGTGTGTTGCGTTTAATCCATCCCATTTTTACCAATCAGCATGCAGGATCTGTTTATTTATGACACTGGTTACATACCATTATTTGAAGCAGAGATATGCTGCTAAATGTGCTTGTTTTATCATTATTTAAACATTTTGGTCACACTTTATCTTAACGTACAATTCAACAAACCATAAACTAAGACTTTTATCTCAATTAATTACTAATTAGTGGCTTATTAATTTTCAGTAAGGCAGTAGTTGTGATTAGGATAGGGTAGGATTAGGGAAGTAAAATAAGTGAGAGAGTGCTAATAAACAGTTAATGTTTTAGTATTCGGCAGGTAATAAGCCAAAGCAAATGGTGTGAATTATTACCCCAACTAAAGTGTTACCAAAATCTCGTATTTTTAAAGATGTATTTCACAGAGCACAATCTGACATGAAGTAATTCAAAGCCATCTACAAACGAAATGATTCCACCAAATAAATATGAGGATCGAAATCAGTGTAGGAGGAGCACTTCACCTACACACTGACTATGCATAGTAAAACATATCATCCACATGGAAAATAAACAACATATTCTGATTATATGTTCAAATTAAATGCAAGGAAATAATGCAAAATACCCTTTCATTATGCCATCAGTTTAAATAGCGCAGAAAAATCATAATTTGGATTAAATGAAACTATTACTGGTGCCAATAGACTCCAGC |
| 391 | lncHLMBrBl_034 | GCACTTTGTACAAGAACGCCATGTTTCATCAATATTAGTCCCAAAGCTGTAGACCAATCAGATGAGCTCAGGGGCAGGGTAAGCACTGCAAACTTGAGTTTAGAGCAGAGCCATAGAGAGAAAGAGTTGCAACAAGTATACTTACTCCAACGGAATGTTGTTTCAACAGCAATAGACTGATTTCACGCGGACACCATTTTTTTAAAAAGCGAAATCGAGGCTGCGGCGGGAAGAAACTTGAAAGTGTCGTTGGAAGTTACATAGGAACGTTGTGTACCTGGCTGTATATCTTATCAGTGAAGAGAAAGTGACACAAATTTATCATTTCACTGCCATCCGAGTGACTTGAAGGTCCGTTCTGGATGAATGGTGAAAATAAAAAGGGATATCAGAGCTTATTTTCAGCTAAGTTAAGGGAAATGGCACTAGTAAGCTAACGTTTTCTTTCCCAAACATGTTTTAGATGCCATTTATCAAACTCA |
| 392 | lncHLMBrBl_035 | ATTGTGTTCTAGGACTGCACAATTTATCATTTCAGCATTAACATCGCAATGTGCGCATCTGCATAAGTCACATAGCAAGAAATGCAATGTTGAGTCTGAATTATAGCTGATCAGGAGCCACAGAACACATGATTTGTAGAGTCACTGCTGTTTAACCATAACAGAGTGAAAGTTTATCATTGGCGTGTGTTTTTAAGTCCTGTGACTGATTACTTCAAGAGAGTTTAAAACATTTGGGCATCAAAAAAATTATGTTTGATTTTTTTGTCTTGTTTCTAATCCAA |
| 393 | lncHLMBrBl_036 | TTATAGTGTGTAGTTATTATAAAGTGTTACCAAGATATCTTCACTAAGTGAACCTTTGATCATCACATCAACAGAAAACCTGATGATGGCACCAATGTTGTAGGTTTGCTGTGTTTTCTTGAAAGATTTTCCTTATTCTGCAGGCCTGCTTAAGTTTTGAGACACTGTCCGACCACACCTACACATTTTCTTGTGTATCGTTTGATTACCATGCAGTGCTCATTGTTCATACGGATGGTGTTTTTAGCATGTCTGGTAAGTCCAGTTTTCTTTTCTACTCGTATACTATTTTGATTTACACAGAAAGTCTAGCTTAGGAAAACGTTTTCTAGTAAAATGTTTCTCAACCCACTTAGAAGACATTTTGTATCATCATTAGATCAAGTGTGGAGATCCTGCTCGTTTTTTCCATGATATTTCCTTTTGTGAAATAATTGATTTTTTTGAAAGGGAACTTTCTAACCAAAGCGGTGTAATCAAAA |
| 394 | lncHLMBrBl_037 | ACACACATATTTGCAAGCAAATCTATCAATATTTCACTGTTGTTTTTAGCAGCACACCACCGTCCAGCAGCTTCTGTCCTCACAGCAGGATCTACAGAGTTCAGCAGGTTTAGTCAGAATTATAAAGCGCTCCGAGAGGTCAGAAGTCAGAGGTGGAGGTCAAACCGAGCCGTGACCTCCATCACTGACCGTCACGTGTGCTTGTGTCTG |
| 395 | lncHLMBrBl_039 | CAAAAGTGTTTTCATATTCATAGAGGAAAATATTAAGTGCAAGTGAATTGTTCACTTTCATGTTTCAAATATCATTTGTAAAAATAAAATATCAAAATAATTTCACTGTGTATGTCTGTTGTCCTTCACTGACTCAGTAGGTGCAGAGAATAGTGTCAAACAGTCGTGTGTGTATAGATTATCCTGTCACAAAATGCGTAGAAAATCCTTCACTACAGTAATAGTTTGATTGCGGTGTTTATATGTCTGTACTGCACTTAATAATGCAACTAAAATCAGGAAATTCTTAATGTCTTAATCCGATTTCTGTTTAGTTCGATTATGACCCTAATCAGATTAAATTGTTCAAAAATCACTGTTTACATGGTAGACTCTTAATCAGAGTATTGTCTTAATCATATTAAAATCTTATTATTGGTGTCCATGTGAACGTACTCAATGTTGCATTGCCATTCAGCACAACATATATTTAAGCAAAAATCAAATCAAATACTATGACTAATTATACAAATAAGTGATCATATTCAGTTTTATTAAATTTTAATTGATTAAAAGTTTCTTGCTGACAAATGTATTAAAAAGCTGTTTGAAGGAAAAAGTTTATTTAAATATGATTTAAACAAGTATTGTGGGGCTG |
| 396 | lncHLMBrBl_040 | GTGCCCGCTGCGACTCCTGTCTTTGGCTGATTGTGTCTGTAATTTAATCGAGCGTTCTGTGTTCGTGTCTGCTTTGTTGTGATTTTTCCGTCAGGCGGGGCTGTCCGCGTCTGCAGGGTGGCAGCTGAAGGACCCCGAGCGTCTGTCTGGCTGAGTTGTTTCTGTCACCGCCGTCTATTCGGTAAATCTCCGTCTGGAATCTCCGGTTCGACGGCTTTTCCCTCTCAGTCGGACATCAATCGCGTTTATTCGCCATCATCGCGCGGTAAACACCGTAAACCGGCGCAGCGTGAGTCCGAATCGGATCCGGATCGAGTATGAGTGCGCTGGAGACTCTGGACCGGCTGGAGCTGTGCGAAAGTCTGCTGACCTGGATCCAGACGTTCAGTGTGGAAGCGCCA |
| 397 | lncHLMBrBl_041 | ATAAATACTAGTAGCATAAAAGTAGATACCTGTGCATTTTGTAGATGAAAATGGTTTGCCAGAGTATCAGTAAAGAGATTAAGAACTCCTAGTTTGTCATTGTGAGCTTTTGTTTATTATTCACCTTGTTTTGCTTCACATATTCATGTCGTCAGCAAGGAGCTCTCAGTGATGGATTTCCCAGTAATTGAAATGAACACTTTACTCGGTAATGACTTCATTCTCCGTGTCACTTGAGAGTCTGCGTTTGGTTTACAGCATCAACACTAAGAGCATCACCATTATCTGACAATCAACCGAACACTAGCGCTGCTCATTCTGAAGGACCGTCTCGTCTCGTTTTACTCTCTATTTACGTCTGCCGTCATAGTTTTGCTGCTACTTAGACTGTAGGGTCTGAGTGAAATGCTGACAAGGGCTCGAATTAGTATTGCAATTTTACTAGGTGATCAACAGCTACTTGTTTTTTGTTTGTTTATTTTTCTTACATAGTGAGTGTGTGCAAATAAATAGATTTTCTAACAAGCTGTTACATTTATTTCGAAAAGTGCTGGCGTAACGGTTCACTGTGGAGCCAGATCAGTCCCAAAAATAATACGTTCACAAAATAAAGTTCACACTTGCAGAGCACAATTCACATTTATACAATCCAATTCGTAAATTTAAACACAATTCATAAATACACAAAACACAATTTGTAAAATTCAC |
| 398 | lncHLMBrBl_042 | ATTTGTTATATTAAAAAAAATCCCATTTAATTTGAGTGAAATATTGAAGTAAGCCCCGTACATTCACTAATTGTGAATTGTTAAAATTCTACACATTTTGACTATATATCCATACTGTAGGGCCGGGCAATTATTCAAAGACATTATTTTCATGCGCATTTTATCAGTAAAGCTGGTCCAGATGGAGCTGAGTTTACTACACAGAGTCGTAGTTCACTGACGAGCTACGCACAAACATGTTTAAAGTCAGTTTAATCTGTTGATAATGAATTCAAGATGATCTTTCTGATGATAACAGCTGTTCATGTCACTTCCCAGTCAATTACGGCTCTGTGTAGTAAATGCTAAAAACCACATATAATTACGGTTTTACTGGCAACTCGCTTCATAACTTGGCTGATGGGTTTTAGAAGGAGCAAAACAATGGTTCGGAGGTTTTACAACACTGTTGATCCACTGGTTTGTCCAACCTGATCTCACGAGGAAATGTAACTATTTTGCGTTTTGTCAGTTTACTGTCTAGTTTGTATGAATTCGTACTAGTTTAGTCGTACGAAAATGTACAATGTTTAAAAGGAGGCATGGCACTAAACCCCACCCCTAAACCCACCCGTCATTTGGAGATGAGCAAAACGTACTGAATTGCATAAATGATCTAGCCACTAAATCTAAAATTGTCGTGAGATATCATTGGTTTGTCCAATAATGTCGTCCCATTGTGACTATTTTTACCTCACATGATC |
| 399 | lncHLMBrBl_043 | TCCGTTTGCACAATCCCTGAGAGCAGGCACAGAAAACATGAGCGGAGGCAAAATTACCTCTGACTGGCCATCTGAGCCCCCTGACTGAAGAGGAAATCACCAATTACTACAATGTCTGAAATATAAAGGCTTAAAGATGGTCTTGTTCCAGAGCCCACAGTTGTTGATGAATCCTTCCACAGTTACTTGGAGGTAAAGGCTAGGAAGAGAAATCTGCAATCATTCGGTCACAATGGCTGCGTTTCTGGGCCTTGTGTTCGGTGATCTGGTGGCTTACCTGTACCTGTGATGGAGAATGAATTTTTCTATTTGGAAAAGTTCAGGCAGAATCTGTCTCTAGGTGGAAGCTTAATGAATATTAATCACTCAATGAGTGCTTGACAATGTAGAATAAGT |
| 400 | lncHLMBrBl_045 | GTGTGAACTGCTCCTTTAAGTGTGTTTGTGTGCTCAGGTGAGTGAAGCGGGCGTTCACAGGCCGCATGTTGGAGGAATTCATGGACGGAGCAATGATGGCTCGTACTCACTGGTGCTCGCTGGGGGATTCGAGGATGAAGTTGTGAGTGTTTCTTTATTAACGATTATGTATTGTGTTGTGTGTGTGTGTGTGTGTGTGTGTGTGTGTGTGTGTGTGTGTGTGTGT |
| 401 | lncHLMBrBl_046 | GCTGGCTTTAGTTACCCATTCTAGCCTTCAATATGGCACCAATGTTTATGGCATTGAAGGCAGGCAATCAAACATAATCGCTAAAGGTTGTGCCTCTTGTTGCCGAAGAGGTTTTCCATGAACCGCTCTTACTAGCTGGACTGTTACACCTAGATGAGCCAGTTAAGGCACTGACGAATGAAGAGCAGACCTGTAGTTATACTGTAAAGGTGCTCGCTGAGTAATAGAAAGGAGTCAGGGTATTGACATTACAGAGTGCTATAAAGTATCCAGCTGCGGCAGTGACATTGTAAATCTCTCTCTAGAGGAGACAGTCTTTGCTTTGGGTCAGCCGGTGGGGTTTCGGTCTGGGTAGTTTGTCACAGTCAGACATTGAATAACAAAGTGGGTCCTAAGGGCTGTGACACATTAGAAAAGCAGACAGACATCAGAAACATCATTGGATAATGATATGACAAGAATTCCACAATCAAACTATGGCAGACAAATCTAATTTAACTTGGAAATAATCAAATAAAGCTTTTTTTAAGAAGCAAAGGAAAGACTCTGCACTGTTTCTTGGTCATTAATTAATTTAAGCTTAGTTAAATTGAAT |
| 402 | lncHLMBrBl_047 | ATTCTTTGAGCTCATTTATTCATCTTAAAACATTATATTACATCATACAGTCGGAAAATACATATGAAGGAGTTGGACAGTACCTTCAAACGAAGAGCAATGAGAGTATATTTAATGATAAAGCCAAATCCAGCAATATCTGCACTATAACTTTGCCAGAGGGAAAACCTTATTAGATTAGCTGGGCCACTTCACTTAAATCACATTTAACAGCTATGTATTCATGTCTATGCGGACCCCCTAAATCCCCCCAAACCCCCTATATTTAT |
| 403 | lncHLMBrBl_048 | TACATAATTTACAATTGTTTTTGCTTGTACTACAGGGCTGCTGTATTCTTTATTCTGATTAGCTGATGAATATTCTAGGTGTGCAATTATTTTGAAATAGATGCACAGCTAAAGTAGTTCTAAGCAGATTGTGACCGCTTTACAGTTCCATATCACTACGCCGAATGATTTCAGTTATTTTATATCCTACAACTGTTAAAAATTAATCAGAACATAAGGATTTGCAGGAGATAATGACCAAGATGGCTTGTGTGATGATTTGGGTAATTTCCGATGCAGAAACTATTCAGCTAGTTATATTGGTGCCATAAACATAGATGAATCAAACCCGCCAGGATTTTTAGAGGGGGTTTAAATCAGAGCACATTTATTGCTGACATGAAAAATACAATCTAGATATTGTTTAATAAACAAAC |
| 404 | lncHLMBrBl_049 | CCTGGATCGTGTCAAATCGTCAATGTCCAAATCCAGCTAACTGAGTAATCCACGTACGAAGAACGGACCCCAGGGCACTAATTTCCAAATAGTGAAGGCTTGATCTGGTGAGCAATAACCTTCCATATTCTCTTGATTATAAAGGAATGCTTGAAAACCATTTCATTAGATTTATACAGCGTTTTACACAACCAGGTCCCAGTGCTCAGTGTAAAAGTAGTGCTGTCACCCAGTGAACTCAATACACCTGTGAAACTTACCTGAGCTCTCTACCAGCACCTGCTCTCAGTAAACACACACTGGACTTAGTTCAGAGATTCATAAAGTTACAGATAATAGTAATATAAACATTTCAACACATTTTTAATGCATCACTACTCTCTTGAATAATTAGTTTTGAACAGCAGATTTTAACAGGCTATGCTAGATTTTAACAGCAGACAGCGCTCTAGGCTAGTTTTTAAACGGCAGATGTCGCTTTAGGCTAGTTTTGAACAGCAAACTGCTCTAGGCTAGTTAGGAACAGCAGATGACGCTCTAGGCTAGTTTTTAAACTGCAGATGGCGCTCTAGGCTAGTATTGAACAGCAGACTGTGCTCTAGGCTAGTTAGGAACAGCAGATGGCGCTCTAGGCTAGTTTTAACAGCAGACAACGCTTTAGGACAGTTTTAACAGCAGATGGTGCTCTAGGGCTAGCTCTAACAGCAGATGGCGCTCTGGGCTAGTTTTCGACAGCAGATGGCACTCTAGGCTAGTTTTAACAGCAGACGGCGCTCCAGTCTAGTTTTAACTTTTAACATCAGACTGTGCTCTAGGCTAGCTCTTACAGCAGATGGCACTCTAGGATAGTTTTAACAGCAGATGGTGCTCTAGGCTAGTTTTTAACAGCAGATGGCACTCTAGGCTAGTTTTAACAGCAGACTACAATCTAGGCTAGTTTTAACAGCAGATGACATATGCTAGGCTAGTTTTTTAC |
| 405 | lncHLMBrBl_050 | CCTGGATCGTGTCAAATCGTCAATGTCCAAATCCAGCTAACTGAGTAATCCACGTACGAAGAACGGACCCCAGGGCACTAATTTCCAAATAGTGAAGGCTTGATCTGGTGAGCAATAACCTTCCATATTCTCTTGATTATAAAGGAATGCTTGAAAACCATTTCATTAGATTTATACAGCGTTTTACACAACCAGGTCCCAGTGCTCAGTGTAAAAGTAGTGCTGTCACCCAGTGAACTCAATACACCTGTGAAACTTACCTGAGCTCTCTACCAGCACCTGCTCTCAGTAAACACACACTGGACTTAGTTCAGAGATTCATAAAGTTACAGATAATAGTAATATAAACATTTCAACACATTTTTAATGCATCACTACTCTCTTGAATAATTAGTTTTGAACAGCAGATTTTAACAGGCTATGCTAGATTTTAACAGCAGACAGCGCTCTAGGCTAGTTTTTAAACGGCAGATGTCGCTTTAGGCTAGTTTTGAACAGCAAACTGCTCTAGGCTAGTTAGGAACAGCAGATGACGCTCTAGGCTAGTTTTTAAACTGCAGATGGCGCTCTAGGCTAGTATTGAACAGCAGACTGTGCTCTAGGCTAGTTAGGAACAGCAGATGGCGCTCTAGGCTAGTTTTAACAGCAGACAACGCTTTAGGACAGTTTTAACAGCAGATGGTGCTCTAGGGCTAGCTCTAACAGCAGATGGCGCTCTGGGCTAGTTTTCGACAGCAGATGGCACTCTAGGCTAGTTTTAACAGCAGACGGCGCTCCAGTCTAGTTTTAACTTTTAACATCAGACTGTGCTCTAGGCTAGCTCTTACAGCAGATGGCACTCTAGGATAGTTTTAACAGCAGATGGTGCTCTAGGCTAGTTTTTAACAGCAGATGGCACTCTAGGCTAGTTTTAACAGCAGACTACAATCTAGGCTAGTTTTAACAGCAGATGACATATGCTAGGCTAGTTTTTTAC |
| 406 | lncHLMBrBl_051 | CTCAAACTGGTTTCGGATGATGACTGAACTTTCATTTTTGACCGAACTGTCCCTTTACGATCTGCCTAATATGTGATTGTGGTGTCCTTGGTCACATGACTCAGATGGGAGGAAGCAAACAGGAGACAGTCATGGTGAGTCACAGAAGCACAAACTCCTCCGTGCTGCTCCTCTTCTGATTTCGCCTCAGTTTGTCTTTGGTTTTTGTGTGTGTGATGTCCATGTCTGACGTAGAGCCGCTTTGTCCCTCGCTGTGACCCTCAACGAAGGTGAAGATGGGGTATTTCTGCTCGGATGATGACAGAGCCTG |
| 407 | lncHLMBrBl_052 | GTTCGAGTCCCTCAATCATCCTCTGAGTGAAAATATGAATCTCACAATCATGCAGAAGATGTTGGAAAGGGTTTAGATCTGCAAAAGATGCTGGAAAACTGAAGAATCTGCAGGAGATTGAGGATTTTTCTGTAGAACAGCAGTCAGTTTAACTGTTCAGAACAAACAAGAGACTCATGAAGAACCATCTCAAGGCAGAAAAACACTCAGAGATCATCAGTATACCACTAACGGTACTAAAAACCAGG |
| 408 | lncHLMBrBl_053 | AGGTTCTGTAAATCAGAGAGCTCAGAGTCTGTAGGTTCTGCTGGTGCTCATCCAGCAGTGGAGGCTCTTCGGCAGCAATCGGCTCGCTCTGGTTCTACTCTGGCCAGACGCTCTGCTTCTGTCACGGACCCCTCTCCCAGTCAAAAACCCAACCAGACACACACAGAGTCCCGCTCACAGAAACACAATAGGATGCTGAAGGAGGTTGTTGCTAAAACGCTCAATAAACACGGGATCGGCAGCGAG |
| 409 | lncHLMBrBl_054 | ATTTATGTATATATGTATTTCTTTTATATAATTCTGTCTGCATAGGTTCTGTAAAGACATCTCTTTGATGTGTACTTTCCTGAGGCCTCTTCGAGGGAAAGTGACTATATCCTGACTGACAAGAAGAAATAGTTGTTTTTAATTGTTACATTAGGTGTTTGTTTTGCATTTTTTTAAACTATTACTGCCCTTTGAAGAAAATATTTCTGATTAACAGACACTGACTTTCATTAATTTAAATTCAATTTCATAACACAGGCAAGAAGGCAAAAGAATGACATATTTGATTTTATAAATAAGTCTTGATATCTTCTGAAATTTTGCCAATGAGAAATAAATAAATATTCAGAGTTTATTTAACTGGGAAAAATATGCTCTGCAGCTTCAAATTAATTTAAGATCTAAATATCTAAGGGCATTTAAGAATGTTCAGACATAAGCAAGTTAAAAATACACTTTGGTATAACTACTGGACAAACCTCAGCTGTTTAAGGCACAGGAAGTAAATAGGACCATGGCTCAGCTATAATTGGGTAACCAGGATCAAAATCTGTTATTTAAAAAAAAAAAAAGATGCAGA |
| 410 | lncHLMBrBl_055 | TGTGATTAGATGGATATAACTTATCTGTAAAATCACCTGTAAACAACCTGAGCTTCTGTTACAACGCGCAAAACACCACTTGTGTACTTTTACAGCGATCAAAAACCATATTAACTTTATAAAACGACCATAAATGATGTTTAAGTATTAAACGCAGAGATTATTTCAGATTTCTCCGGCGCACGAGAGGATGCTGTTAGCTTGTTAGCATCCGTCTATGTGTGTGCGCGGAGTTTTAACGGCTCTGTCAGCGCTCACGGTCTCTATCGGGTAACGAGCTCCCGGAGTAAGTGTCGAATACAGCGGGTCCCGGTGAACGGAAAGTCCCGGTAGAGAAGCCGGAGGATGATGAGGGTGCGATGAATTTCGGCCCAGCTACTCTCCGCAGGTTACCGGAAACATCAGAGCGCA |
| 411 | lncHLMBrBl_056 | AAAAACTCTCAGTAAAAACAGCATTTTTTGCAGTTTAGTCTCAGTTTCTTCCAGCCAGAATTGGAGTTATGAGTTCTCCTAGTGCTGGCTGGGCACAACTCTTTTAATCCTACATACGGTGGCTCAGAGAGTTCAAATGTGTCTCAAGTTAAAGGGTTAGTTCACACATAATTGAAAATATACTCATTATTTACCGTTCCTCATGCTGAGTTTCTTTCTTCTGTTGAATATTTGAAGAATGTTGGAAACCTGTAACCATTGACTTGCATAATATGACAAACAAATACTATTGAAGTCAATGGTTACAGGTTTCCAACATTCTTCACAATATCTTCTTTTGTGTTCAACAAAAAAAGAGAAACTCAATAGTGAGTAAATGTTGACTTAATTAAATTGTTTTAGTTTTCACCCCTGAAAAATGTAGAAAAAATTTTCATCAGATTGTCACCACGCACATATATTAATTGATACAGATATTAAATAAATGTAAATAATAATTAATACAGAAAAACATTGAAGGACACAGTGGAAATGTGTCAGGGAGCCCAAAAAGTGACAGACCAGATGCTTTCTGTTTAGAGTAAGCAGTTCATTTAATATTAATTGTGCAATTGGTCAGAATATTATGGATAGAAAATATACAGAATAATAAACAAAAACTTGCATACTGCTCTCAAAAGACAGATTTTTTGCTTATTATTCTGTTTATTTTGATCGAGCACCTGCTTAAAGTGTCTTTAGGATGTGCGAACTCTGAGCTTTGATCTATTGGCTAAAAATATACAAAGAAGAAACTGTAAGCTGTAGACATTAAAGGGATAGTTCACCCAAAAATGAATGTTCAATCATTATTATTTCCTCTTCCTC |
| 412 | lncHLMBrBl_057 | TTCATTTTCATCCCTAACATAACAAAATGTAGTCCATTTGAACAGTGCACAAGGGTTAAAACCACTACATTTTAATGTTTCCATTGTGTTTTGGGACACATTCTGTTACAATTTGGTGGTTATGAACATGCCACCAGTGAAGGCAATGAATTACCAGAAAAATCCACAGGGACTCTTAACAGTTTCCATGTAAAACCACTACAACTTTCGTTATAACCATTATTCAATAAGTGTTTCTACTGTTTTTCAGCAGGATACTGGCAAATAGATCCTGCTTTGTACTTCTAGAAAATGTGATCAATACGTGAAGAGTTACTCTTTGACCTCTTCTTTATATTGACTGTTAATCTGCCCTAACTCTTCTGTCCAAAGGTCAGGTCATGGACTACGATGGCTGCTTCACACTGTGCAAGAATTCACGTAAAGCTATCATCCATGGCACAGCCTTAAATACAGTTACAAGCTGAAGGACGCTTCTTTTGCATTTGTCTTCTTGTTAGAAGTTTGGCTTCAGGTTTCAGATCAGTATTACGCAG |
| 413 | lncHLMBrBl_058 | TCTAAAAATATGTTTACCTGTTTGCTTTAATATCATTATATAAATAAATAATTTAAAACAGAGCTAAGGTAAATGGTATATTGTCAGAAATATCATTTCCCAGTACTCAACAACAATATCATAAGTTTTTCTAGGATCGTGCAGCTCTAAATATCACTTTTTGGATTGTGCATTTGCTCTTAATTGGAGAATGACCCACCAAAACCGAAGAATGTGTCTAGTGCGTATGTGTACTACTCATTTATGAAGGGTTTGAACCCTGTGAATGGCAGCTGATTAAGTGCACTAGATTGGTTGATTTAGATCAGCTGATCTCTCCTTCTATAAAGGTTGTGCATTCTATTAACATTTTATGAGGGCAATAAGAAATGTAAGTGCTGGTATTATAATTATAGTGCTACCGCTTGCAGAAA |
| 414 | lncHLMBrBl_059 | ACTCACTTCATGCTGCCAGGAGTGTTTTCAGATTTTTGGCTGTCCACACATTCAGTTCATTCATAAAGCTTATTAATCTGCTAATACACATCTTCCATCCTTCAATGACATTCTGCCACCTTGACTATTAATCGTTAGGACTCCAGTACAAAAGCAGGTTATAAAGTACAATGTGTTATTTTTTTAGATGTTGATAATTATAGACAATTATACACAGTAACGTTTGAAACAGAAGGGGAAAGAGTAATTATAATTCCATTTATCAATCTGCTAATACACATCTTCCAGGACCTTCAATGACATTCTAG |
| 415 | lncHLMBrBl_060 | AGATAATCTGGCGATAGTCAGATGTAAGTATTAGCTTGTAGAGTATTGCCGTTAGGTACTATAGAGCAGGAAAGAGCACACCTGCATGTTTTATTATCTAACGATTCCTAAAACGCAACAGACAAAAAAAGCCAAGGATCCAATATATTTACGCTGCATTACGTCTGCATAAATACTACGCTATGAACAAAATAAAAGTCCTAGGAGTTTTGTTTATCGGAGGGATTTTTAGCATCACACTGCAAC |
| 416 | lncHLMBrBl_061 | GCGCAGTGGCGTGCGCCTGTAATCCAAGCTACTGGGAGGCTGAGGCTGGCGGACCGCTTGAGCTCAGGGGTTCTGGGCTGCAGTGGACTATGCCGATCGGGTGTCCGCACTAAGTTCGGTATCGATATGGTGCTCCTGGGGGAGCCTTGGACTACCAGGTCGTCTAAGGAGGGGTGAACCGGCCCAGGTCGGAGACGGAGCAGGTCAAAGCCCCCGTGCCGATCAGTAGTGGGATCGCGCCTGTGAATAGACACTGCAGTGCAGCCTGAGTAATACAGCGGGACTCAG |
| 417 | lncHLMBrBl_062 | AAGAAAGCAAAGGTTTTAATTGGGACGGAGTGAAGGGAAGTAAAGAAAGTCAAAGGCAACAACAAACCTCATTAGAGAGATTCACTATAGCCCAGCAGAATCGAGCAACTGAGATCACATGACATTTCATCCCTAAAATGATACCCGATGAAAGCCGTCGTCTAAAAAGTGTCGCATAGTGACACCATGGCAGTGGATTCAACAGTAGATTCCTCCTGACAGCTGAAAGAGCTACATCTGTATAAATAATGGGTATAAAGCAGTAAAGCAGAACAGTGATAGGGTCAAAGCAGATGAATTACATACTTAGAAAAATTAAAGAGAAAGAGCAGAATCTTTTTACATTGTTTTACAAGCTTTCTTTAAAAAAT |
| 418 | lncHLMBrBl_063 | AAAAAGACAGAAATCTCTCAGATTTCATTAAAAATATCTTAACTTGAGTTCCAAATATGAACATTTCCTTTGAGTTTGGATTGACAAAAGGCTGAAGTAAATGACAGTTTTCATTTAGGGGTAAACAAACTTAGTTAAGGCCAATTTACACTGCACTGACAGACAGAAACCAACACAGACAATACCCCAAATTACACTGCATCACTTCTCAAACATTCCACGATCCAACAAGTGCCAACTAACACCTTCAAAAGCAACACGCTCCCCTTTTCCACCAAAGCAGTTTAAGTGCTTCTTTAGAGCCAGAGCCTAGTTAAAGTTAGTTTTTGTTTCTTTACATTCAAAGCACCAGTTCTAAACCAGAAATAATGGTTAATTAGCAGCATCAAAACATTACAGAGCTAATAGCAATAACCGTTTACATCAGGAACAGAAGCCGAAGTTACCCTACCAGTGTTTACCAACCACACAAACACAATCTTTAGAA |
| 419 | lncHLMBrBl_065 | CCTGTGTGTAGGAGTTTTTAGGAAAACTATCCTTTAAAGAGTCACACAAATCTCACAATACCAAAGAAATCTTGAAGTTTGGTGACATACCTTCAAATGTTCTTTTTGGGGTGAAATATCACTTGGAAATGTACTCTTTTAGCTAGAGCTTGAGGTCTTTTCATTACTTTTCATTGATATTTTACATCACTAAACAAACAGTTCAAATGTTTAAATGCCCAATTCAAGGTGGTTAACGTTGTATTTCACAAAATATTGAACACTTGAACACAAGCTCAGATTTAAAGGGTTAAACTCAGTTCCTGGAAGGCCACAGCTGTGCACAATTAAGTTCCAACCATAAATAAACACACCTGATCAAACTAATTGAGTCCTTCAGGCTTCAAGCAGGGTTGGAACTAAACTGTGCAGGGCTATGGTCCTCCAGGATCTGAGTTTGACACCGCTG |
