# Supplementary material for: Dynamic Expression of Long Non-Coding RNAs (lncRNAs) in Adult Zebrafish
Source: PLoS One. 2013 Dec 31;8(12):e83616. doi: 10.1371/journal.pone.0083616 (PMC3877055; doi:10.1371/journal.pone.0083616)
Supplement: Table S2 — A dataset of 77 putative lncRNAs that are predicted to have predominant expression restricted to particular tissue type investigated. (DOCX) [file pone.0083616.s003.docx]

**Table S2:** A dataset of 77 putative lncRNAs that are predicted to have predominant expression restricted to particular tissue type investigated.

| **S. No.** | **LncRNA ID** | **Sequence Description** |
| --- | --- | --- |
| 1 | lncH_001 | ATGGAGTTGAGTAGTCTCATGCGAGACTCCAACAAACAAGGCTTTTAACATCTAACGCTGCTACCTGAACCTGCTGTTTCTTAGCTTTTGTCTAAAGTGATCAACACCCTGCCATGTCCTTCCCTGATCCCTCTGTTTCTCTTGCACTTGCTTACATGTTGGCTTAGACTACAGACACAAGCCGTCCAATCCTCCCAAGCTTCTGTCCCCACCTTCTTCTTACATGTCTACCCACACTCCCTCCACACCCTAGCATCCACTGCTTCTGCCCTTTACCCCTTCCTCTGTTTCCCTACTTTACCCATTTCCTTTTTGGTACCCCAACCCCCAACAACCGTTCA |
| 2 | lncH_002 | ACACGCTCAAGCAATACACACATGGTTTGTGTGTGTTTGTGTGTGTGTGTGTGTGTGTGTGTGTGAAAACTGCTGGAGAGAACAATGCTTGCAGAAGAAGCGGCGCTTTACAAAGCCTGTAGATCAGAGTGAGGCAGTGATGGCTGTCAGCAGGCCTGTAGACGGAGATAAGCTCCACTGTCACCGCATTCACTCTCTATTAGATTAGCCAGACAAACCAACTACCACAGCAAAACGTACCCGCTAATCGAAGAATTCAGGCCTTCCCTTGTCCTTAACCCTCGTGTACTTAATATAGAGGATGAGTCAGCTCAAACG |
| 3 | lncH_003 | CTGATTCAATATCTGCGAGAGATTATGGCTGATTATTTAGCTAAATGGACCACACACGGAAAGACGAGTCATCTCACACGCGCTAATCAATTCAAGATACGCTAGCTATTAGCACCCAGTCGAAAACCCGATCGCACTCGCTCGTATATTCAAGCTTACTGTACAGTCCAGTCCACAACAGAAGAACAGACACATCTGCTTTCTTCACTCGTCTTTCTCCTCCCCGCAGCTCAAGCTTTCACTCGCTTTCTCTCTCTGTAGTGGTTTGAGAAAGTTCCAGACATTCCTGAAAGGATTCCGCTCGGCAGCAGCCAGTC |
| 4 | lncH_005 | GTAAAACCTCAAGGGAACCAGGGTTGTATTTGCAAATAATATTTATCTTGTGCTCCTCTTATTTGTCAGATTACCGTGTGTGAATCAAAAATGATGTGCAAGGTCACCCAAAACACAAGTATTTAGGCAGTATTAATAGTCAGTTTAATATTAGGTTAGGTTAGTTTATCCCACCTTAAGCAAGTTTGATTTGCACAGGTGACAGTTTTGTTGACAGTTACACATACAGTAGAAGAACTGAA |
| 5 | lncH_006 | AGATGTTCCCCTGTAATTTACTCACTGAACTTCAATGAAGAGGTTTTTACTAGTTTAAAATGTCAGTTTCTTTTGGAGAACTGGGCTGTTGCTGTGAGACTGTAAAATCAGTTGGGACCAGACTGGAGATGGGACCTTGACTATGAGAGAATGACTATTTGAATAGTTTTAATGTCTAACTTGATTGGGAATGGGACCTATTCTTTGTAAAATAGTTAAAAATACTCAATCTTGAAGAAAAA |
| 6 | lncH_007 | TTAAAATGTAAGTTTAGTCAATGCGGCGTAATTGTAAAGCGCCATCTGTAGGTCAACACTTAAACTGCATCTAACTCAACATTCAACAGGCTGCATTGCAGCATGTTTTTAATCAATTGTTTAATCTTTTAATGAACCACTCCAGCTTCTAAATTCACCCAATATGCTTTTATTAAAAAGAAAAATTATCTATTTTCTGTTTGTAAACCACAAATAATGGCAAACGGTTAATAAATTAAGCATGCTCTCACCCGAGGGTACACAATAGCCTTATTGGTTTCAATCAAGGCTGAATGTGAAGCAAATATTTAGACTGTTGATTTGATATGATGTGAATATGATGTTGTTTTATTAACTAATCTCTCTCCACGGACAGCGACGCGCACACATTTTAATCACAGCAAATCACAATTCAATATCGCGTGCGAGCCACAGCTGCCTGACAAAATAAATGAATAATTTAGCATAAAATAACGTTCAGGGGTGATTAAAATTCAAAACAACAGTAAGAGTTTGGTACCAAGATGCCCT |
| 7 | lncH_008 | AGACACTGGGTTTCATCCACTAATAATTGCATATGTTTTTCATATTTATATGCACATTTGAACCTCACAAAAACTGTCCGCCATCTTCACAACACTTCTTCTGTTAGTGAATTTGATTAGTTCTGAGGGGGTGTCCTCGTGCAGTAATTTGCATAAGACACGCCCACAGCAGCTCCATAGACAGTGTTCAGCATATATAAGTACACGCCTCACAAATCTCTCTTTAAATTAATATTTT |
| 8 | lncH_009 | GCAAGATATGTATTAAATGATATAAAGCACAGAAGATTTTTGTGACTTTGTGAATTGTGGATTTTTCAGTTCCATGAAAATATGTGAATATTTATGCATGCTGTTTTTAGTTATGTGGAGGCGAAATCTGGCCTAGTTACAGCTTGTGTCACTTAAAGCAATATTTTACCATACAAATGAGAGTTCTGTCATCATGTACTTGCTGTTCTAGACCTAATGCTCAGCATATATAAGTACACCCCATGCAAATCCATCTTTTAAATTCATGTTTTAATCAGAAGCTATACAATATTTAATTTGTACATATACGTTATATTTGTCAGAACTGAAGCCAAATCTGGAGCTTATCTAACAAAATAACTCACGATTAACGGTCAAAAAAAACTAGTACAGTGCACCC |
| 9 | lncH_010 | AGTACGAAAGGTTTACATTTAAATTTGCTCTTACACAAAAATGACAAAATGAGGAGAAAGGTAACACAAATGCCTATTGCAGTAATGCCACCTACTGGACATAGTGGATGTTGACACAAAAGCCTGGGTTGTACTGAAATAATTCCTCAAAATGACCTGACCTAAAAAGGAAAGACAAATAAATGTTAGTAAAAACAATGTCAATCACATAAAAACTAAATAAGCAATTCTGTAAATG |
| 10 | lncH_011 | CAAAAATCAGAGGATAACTGAGTATCTGCTGAAGTGTTTCCAGAAACGCTGCAGACATCTGAGGCTGAGAATAAATAACATTCAACTTTCGAATTCTAGTAGTGAGGAAATGTCACAATTATTTCTAATTTCACTAGATTTCCAGCATTCACTGAGCTCCTGAGAGCTTCCCATTCTTTCACAAATGTTTGAAGATTTTCT |
| 11 | lncH_012 | AAGAAAACAGCCCAGTCAATTGCAATTCTCATTTTGATTTGCTTTTTAGTCAACCATGAGTTTGGGATGCTGTTTTTGCTTTGCACTTCTTGTTTCTGCACATTTGACAAGGGAGTAATGAGTACAAAGGAGTAAATGAAACTTGGCCGTTTACAGCAGGCCCCTTGACTCCGCCCCCTTGTCAAATCAGGGCCACAAAGTGAAACGCGCAGAAACGTGAAGTGCAAAGTGAAAACAGCATCACAAACTCAAGATTGAATAAATGCAAATATTTTAGTTTATTTTTGCTCTGC |
| 12 | lncH_013 | AAACAAAATCTACTGATTGAAAGGAACCTGTTTTTCTAGGGAGGTTTAAAGATGAGCAGTAAAGACCAGGAAGATGGCGCCACCCAGTGTCTGAAAGCTCAGAGGTCTAGGAGGAGAAACGGGATCACATGAATCATCATCACATCATTTTTGTGACACTGTACAATGATTAACATTTTTACATTAGTGTGAGCGTTGGGGTAAGCA |
| 13 | lncL_001 | GCTAGATACATTGTTCTCCATGAAATAAAAAGCTGACATTATTTTCTGTCCCCTTATGCCTTTACCAAAGGCAAGTCTAAAATAAAACAAATAACTAGCTTAGGTTACCTCATGTACTTCATTCCAAAACCTCTAAAGCCACAGGATAACTTTCTGTGAGAAACAGTCCACATTCAATCATTATTCACTGATAATGAAGATAAGCTGCAGCACAGTTTAGGATTAGCAGTGCACTA |
| 14 | lncL_002 | CTGTGTTGTGCGTTAATATAGTGTGGGACATCAGCGGGATTAGGAGTATTTGGTCTTAAATCGGGGTAGTGTGAACGGTCCCTCCCCAGCAATGGGTTAAAGCGGCATTACAGCTTGTAAGATGGGTGTCCCGCAGATCTCCCGGGAGACCAGCTCCACTTGGGTGTTCCAGCACTGAGCACAGACCATTTAGATGACACGGAGGTGTTCTGATGCCATCTTC |
| 15 | lncM_001 | AACTGACCTACAATTTTATCCCAAAATAAACAAGAAAATTGAGAATACATCAAACAAAGACAATCATCACATTGTCCTTTAATCTAAAATATTTAATTTTCACTTTTATTTACAACCTATTAAATCTTTAACCCACTTTCCACTAATTTAGAAAAACAAGATCAAACTATACAACACAAATGTTAATTAACACGCCGCTGCTTTACTAATCCTTTAAATATGAACTTGGGCAAGAAAAATC |
| 16 | lncM_002 | GCAAACACTGACAATTACTTTCCTGAATGTAGTTTACAGACTTAACTTACATTTAATACTTTTACACAGACTTTTATGACTTGTGTAAGTATTTTATTTTATCAGTCAGTTCATTCAGTGGTGGAGTCTGTCTGATTTAATTGTGTAGCACTTTTGATATTTTTTCCTCCCCAAGCGTGCAATATTATTATTTGAGAACAAATCTCTTTGGAATTTTATATCTATGTATACAGTCAAGCCTGCAATTATACATACCCTGTA |
| 17 | lncM_003 | CCCTCCTCAACCGCCCTGCTCCCCTGTCCGCTGTTTACTTTCACTTTCTTCCGCGACTCCCTAACGCTTCACTTTTTGTCCGTGACACCCCTCCGCCATCCGCCGCACCAGTCCGCGCGGCACCTCTTCATCCTCGGGTAACATGCCGGTGCCGTAATCCCGATCTGTTCCGGGACCTCGCAATTCACAAATTAAGCACTGCGCACACCCTAATGTGACATGGAAGTCAAGGGGGATCAAAGTAGT |
| 18 | lncM_004 | GAGCTCTTGAGCATTTCCCCATTTTGCTGCAGGACAATCCCATCATCCTCTAATCAAGCCTGTGGTTTAATCTCAATCCCGTCTCCATCCTCGTGACGCAGACTCTGGACAATTGAAGGATGTTCAAACACTTCGGCATCCACTTGAAAGTCCCGACCGCTCCTGCCAGATCTAAGTGCCGTCTTAAGACTGTAAAGACATTGATGGAATAGTTT |
| 19 | lncBr_001 | GTAGCAGCGCACTGTGGCTGTACTGTGTATTGGCACTGGTGGGAGGCATGCCTTAGTGTTTTAGTGGCAGGCGTGTCTTAGAATTTTAGCCAATGAAACTGTGCAGTGGACGGAGCTACACAACATTGTATATAGATGTTTAATATTCAACCGTGTGGAATTTTATCCAATGAGATAGCACAGTAGGTGGAGCTACACAACATTGTATATAGATGTTTAATATTCAACCGTGTGGAATTTTATCCAATGAGATAGCACAGTAGGTGGAGCTACACAACATTGTATATAGATGTTTAATATTCAACCGTGTGGAATTTTATCCAATGAGATAGCACAGTAGGTGGAGCTACACAACATTGTATATAGATGTTTAATATTCAACCGTGTGGAATTTTATCCAATGAGATAGCACAGTAGGTGGAGCTACACAGCATTGTATATAGATGTTTAATATTCAACCGTGTGGAATTTTATCCAATGAGATAGCACAGTAGGTGGAGCTACACAACATTGTATATAGATGTTTAATATTTAACCATGTGGACTTTTAGCCAATGACACTGCATAGTGGGCGGAGCTACACAGCACTGGATATAGATGTTTAATATTCAACCATGTGAAATTTTAGACCAATTGGAGTTCATAGTGGGCGGAGCTACATAGCAAGATATAATTTTGGAGCAATGAGATTGCACAGTGGGTGGAGCTACACAGCAATTTATGTATAGGTGTTTAATATTCAAC |
| 20 | lncBr_002 | TCCCAAGTCATTTTTTCCCCTCATATTTTATTAACAGAAGTTATTGATTCCAGCTGCACATATATGCACATATAACCAGTCAATCTGCTTGTTAAATATATAAAAATCTATCCCAACAATGGTGAGTTTTCTTCTGTTGTTTCTGTTTCCATCCAGGAGATCGATCAAAATCGGATTAAATCATTTGTGTTTACGCTTCAGCCATTGATGTAAACATGTAAATTCATTACCAATCCTTTATATTTGCCAACAACAGGATTAAACTGCAATAGGCAAAGGCAGCTAGATTAGATCCCCAATCAAAATCTCTAGCACAAAAAAAGGAGGGACTAATTTTGCCCGGAGAAAAGGTCTGTTTTGATGAATGGTCCCAATCAGCAGAAGCAGAATCTGGAAGTTGTGGAGGGATTATTGAATTTTAAGCCTTTGTAGAATTTTAATTAACTTTCTCTTTGGCCAGAAAACGGTTTTCCCTCAGTCGGTGCGGTTCAATAATCCAGCCACCCATGTAATAAGATGTTCATTACGAAATTGTACTTTTCACGCTTCGCTTTTATTCAGAGCAACTTCTGTCTGTTAGAGTGAGACTTGAAGCTAAAGAGAATATCAGATATACCTGGGCAAAGGTACAATAATTGGTCATTTTTTATTTTTACTGGTCAAAATAATTGGCCTCCTGCAAGACAGTTGGATTCTTGCATAGTTCTACATGGAATGACTATTGGGTCATTTAATGGGATCCTTTGAGATTTGGGATAAAACTAAATAAAATAAATACTGCTATTAGGTATATATTATCATACGAAATTTTGAAGTGATGGCATAATTAAAATGTACAAAGATTGAATCAAATTTTACATTTTAAAAGTGCATGTGTGGTGGG |
| 21 | lncBr_003 | CACACAGGGCTGAAATTCAGTTGTAGTAATGTGCATTTCTGTCTAAAATCACAACAGACTGGGGCATCTTACCATTGAAAGCAGACCAGACCAATCAGAGCAGAATAGAACCGTCTGACCAATCAGAAGAAAGCAAACCAATCAGACCAAAGAACAACTGTCTGACCAATCAGAACAAAGTAAACCAATCAGAGCACATTTGAACCTTCTGACCAATCAGATTAGAGCAGAGCCACCTGACCAATCAGTACAGAGTAAACCAATTAAAGCAGAGTAGAACCTTCGACCAATCAGAGCAGAGTAGAACCGTCTGACCAGTCAAAACAGAGTAGTCATCTGACCAATCAGAATAAGCCAATAAGAGCAAAGTATAACTGTCTGACCAATCAGAACAAAGTAAACGAATCAGAGCAGAGTAGAACCGTCTGACCAATCAGAACAGAGTAAACGAATCAGAGCAGAGTAGAACCATCTGACCAGTCAGTACAAAATAAACCTATGAGAGTAGAGTAGAGCCTCTGGCCAATCAGAGCAGAGTAGAACCGTCTGACCAATCAGAACAGAGTAAACCATTCAGAGCAGAGTAGAACCGTCCGACCAGTCAGAACAGAACAAGGTAGAGCTTTCTGACCAATCAGAGCAGAGTAGAATCATCTGACCAATCAGAGCAGAGAATAACTGTCTGACCAATCAGAACAACGATCAGAGAAGAGTAGAGTTAACCAATCAGAGCAGAGCCATCTGACCATTCAGTACAGAGTAAACCAATGAGAGCAGAGTATAACCGTCTGAC |
| 22 | lncBr_004 | GGATTACTTTTAAAAGTAACTTTCCCCAACACTAAAGGTACATATATAACATAATATATATATATATAAAGGTTTTGTGTGTGTGTGTGTGTGTGTGTGTGTTTCTCAGCTCACCCTTTCTTTAAAAGTGTGTAAAACAGGCTATTCCTCATTAGAAACAGCCTTATCAAAGCTCCTGCCTGCTCTGATACTGCTCAATATTCATCAGTCCAGAACTTCGCTTTCCTTTTAAAAATCACACAGCTCTCTCTCTCTCTCGTAGCATTGAATATGCAGGCTTCCTGATGAGGTTGCGTAATGATTATTTAATGCTGGCATAATTGCTTAAGGGCCTGCTTGTGCTCCTGTACTTACTGGCATGATACATCAGCATCATTACCAGAGCGATTGGCCCCTGATCACTTGCCTCTGATCATTGCCATCCATAGGCTTCAACAGGAAGCCACGACACACAGTGAGCTTCGTGTGTTAAACCGGACTGACGGCTGCGAGAAGATGAATCATGTATATGCACGCGAGATATGCACTTTCAAGTTCATCCGCTGGGTTTCTGGAGAGCGTGTGGTGCTAGAGAGTTATCG |
| 23 | lncBr_005 | TTTTGATTGTTGATATAAATCACTTATTGAGTCTGTCCTTACTTTTAATATTGTAGCCTGGTTTAATTCCCTCTCTGTTAAAAGCACAAATTAACTTTTAAGTTTGGTAAACTTAACAAGTAAAATAATTGGTGAGAGACAAACACCTCTCATTGATTTATTTATAGCAGCGATAGAAAGAAAAACCTCCACTATTGTGGAAGACTCTTTACATGGTTCCTTTAAGTTACTTCCGTCTGGTAGACGATATAAAGTCCCTTTAGCAAAAAAGGCTAACTACAAGAAGACATTTATTCCCATTGCCATTGTTTTTTTAAATAGGATTTTTAAAATAATTGTGTAAGTGTATTTATGTTTTGTTGTTGTGTGTGAGCCCAAAGACAATTTTTTAACCTCTGTGGGATAGACAATGACGCTTATTTGAATTGAAAAGTTGTTGGAGAAGAGATCAATGCCCCATAATGCAATACAAGAGCAAAAATAAAAGGAATAAAACCCATGAATCACAAAATACGGAAAACTGACAAAGTATGGATATTTTTCACAGTGTAAAGTTTATTTCAGATAATTGTGACCTGTGACCTTCAGTTCTTCGTTTCCTTATTTTGGGATTCACAAGTGTCATTCGTTATTTACTGTTATAAATTGC |
| 24 | lncBr_006 | ACCGGTTTTCTTAAGCAACTATTACTCAAAAGTTATGTTTTCAAAAGAAGACAACAATTTTGAGGAGAAACGTGTCGCATTTGCACATTTAACAGCTTAATTCAACCCAAAATCTGTTAGTCACACCGCATTCAAAAAACAAATGTGGAGCTACAGATACTTCATCTACAAATCCACAAAACAAGCAAGTCACATCAAAATAACCATAACTATAAATAGATAAATTAACAGGATAGATTTTGTGCAAAATCTGTTTCAGTAGCACAAATTTATGTTTTTATTGTATGTTTTTATACAGAATGGTTATGTGCGTATGATATGTAATGTTTTTGGTTAAGATGGTGTGTGTAAATGGTACAGACGTATTTAAGCATACAAAAGGTAATATCTGTGTGTAAGAACACACCAAATACAAAC |
| 25 | lncBr_007 | TGAATATTACCATCCCTAAATGACATTTGTTTACAACAAATTAGTTTTACTGTGTCCGTTAACTTAAATGAGCCTGTTTACACACACCAAATATTCAGAGGCCTTGTGAAATGTTGTATGGACTGATTTGCAGATGAAGACTGGCAGTCTGTAAGGCTGTGTGTGAAATCAAAGTCAGCTGCATCACTGCACACACACTCTCCGTGACTGAACACACACAGCACTTGAACACAACAGACAGACAAGCCTGAAATCTACTAGTTCAGATGATGCAACAAAGAGATACAAATATGTACTACAGTGCTTAGACACAGTAGGAATAGAGCAACGAGTTGGAGGAAAATAGTTTCTGCTACTTTCTTTTATAAGAACACTCCTTTTTTTGGAAATAGGCTAATTTTACAAGTAATATCATTGTAGCCATCATACGGCAGCAAAGTTCCTTGATTATTACGGCAATATTAGTGTATAGTTCCTAGCAATATCGACCTGGACAATAAGAACTTTTCATTTTCCG |
| 26 | lncBr_008 | GCACAACAATCTGCCTAGGTCAGTGTTCTCAGCAGTAAATACATGGAGAACTTTTAAACAAATGTTACTTTAAGGAAAATATTTAAACCATTATGGTAGATAGAGTTAAAAATCACTTGTTTAAATAAAAGTTTAAAGTTTTATCGAGATGGATTGTTGGTGATTGTATTGTATTTTGGCCAGATTGGGAAGCAATGAACAATGGAAAATTAAGTCCTGTTAAAAAAAGATATAAGACCTACAACACAATATTTCAGTAAATTTAAGACTTTTTAAGGCCTAACATTTTGATCTTGGAATTTAAGACATTTTAAGGCTTTTTAAGATCCTGCGGAAACCCTGATTTAACCTTTCTTAAGATAATTTGCAATCAATTACCTGTGCTAAATATGTATTTTTCTCTTGTCAATTTAAGGAAATATAATTTCCAGTAAAAATATATTTATAATAATTTTAAATACTTTTCCAGACTCTTACCAAGGTTGTCTTTACAAATATATTTACAGTATTAAAAAATATTACTTAAACTAAAAG |
| 27 | lncBr_009 | AACATTCTAGGCTACTTTTTAACAGCAGATGGTGCTCCAGGCTAGTTTTTAACAGCAAACAACACTGTAGGCTAGTTTTTTAACAGCAAATGGCGCTTTAGGCGAGTTTTTAGCAGCAGTCAGTGCACTTTTAGGCTAGTTTCTAACAGCAGATGGCGCTCTAGGCTAGTTTTCAACAGCAAACAACACTATAGGAAAGTTTTTAATAGCAGATGGTACTCTAGGCTTGATTTTAGCAGCAGACAGTGCGCTTTAGGCTAGTTTTTAACAGCAGATAATACTCTAGGCTAGTTTTTAGCAGCAGACAGTGCGCTTTAGGCTATTTTGCAGCAGATGCTGCTCTAGCCTAGTTTTAAGATGCAGACGGTACTTTAGCCTAGCTTTTAACAGCAGATGACACTCTAGGCTAGTTTTTAACAGCAGATGGTGCTATAGGCTAGTTTTTATCAGTAGAAGATACTGTAGATGCTTTACTTGACTGTGATTCAAAACTGTGCTAAAGATTGTCATGCTTAGGACTTAACCAGGTTTTT |
| 28 | lncBr_010 | GTTTCTGTCTCCTGTTCCAATTTAGTATATAATGCTCTTAAAACCAATACAAGGCCAATCCTGCTTGTGGAGGGCCATTGTCCTGGATACTTTAGCTCCTACCCTTACTAAACACACCTGAACCAGCCAACCAGCTGAATCAGCTAATCAAGGTTTTACTTGGCTTACTAGAAACTTCCAGACAAATGTATTGAAGTGAGCTAAACTCTGCAGGACAGTGAAACTCCAGGATTAGGTTTCGACATCCCTGGTTTAAACAATTAACTTTTTAATGTTCAGGTATATTACATGTTATCTCTACTTTAAAGTAGAGCTAACATAGGTAGGCTCTGTCAATCCAGCAATTGAAAGAACTTTGGATGCTGGCAATAAATGAACATATCACTAAAAGTTCAAGAAGAGTTAAGATGGAATGTATTGCTTTAAAATAGTTCCAAATAAAAGTATAAATATTCTGTGCTCAACTTAGGAAATATATTATCAAAAACTAATTGAGAATAACACCTAATATGATCAAAGATTATTTGTTTGAGTTTTTAAGAGAGAAATCAGAACACGTTTCTCATAATTTATAGTAGTATG |
| 29 | lncBr_011 | GTTGGTTTTATAAAGACTTGCATGATGGTTCCAAACACAATCAATAAACAGTGTGAAGTATAACAGTAATGTACAGTAAAATGATCACACAATAAGTTATTTTAACAGTTTTCCAGTGCTTTCATGCAATGCTAAAGAAACAACAGCAAAAGATCACAGTGATATCTATTTATACATTCATCAAACAAACGCTCAGTTTCTGCTTTGCAAGTATACGGTAGGAATCCCGTTAGCAACTTTGTTTCAAATGAACGCCAGGCAGCAGCACAAACTCACAGATAAACATCTGAGCACTGTAAACACTTCATAAATTGATAAATTCAGTGTTTTTATCTGAATATCTGCACATATTCACATATTTTTTTGTCACATATGCCATTTTGTTGTTGTTTTTGGACAAAATCTCAAAAATATGGGTTCTAAATAT |
| 30 | lncBr_012 | CAGATTTCATCTGACATATCCCATCCTCTTTTTGATTGATTTCAACCACTTCCCTCAGGATCTTGCGACTTGCTCTTTTTGGCAAGGACCAACAGACTTAAATTCTCATTTGTTCCTTGTGCTACTGATTTGCTGAATTTAATGGGAGGAGTCAGTGTTGAGTCTGAGTCTATTAATGTTGCTTAGTGTTTGTCTGAAGTGTGTGGTGTGGTGTGTATATATATGTTGTTAATCTAATGCAATGCAAAACAAATCGCCCCAAAAGGGTGCAATAAAGTTTACATTAACCGTAACAAAGCAGCGACACAGGTGAGGCTTAAGATGCTGTTTACCCCAAGCAGAACATGAGGATAAATATATCAGAAGTCAGAATTATTCGCCCCCTGTTAATTTTTCCCCCCAATTTCTGTTTAACGGAGATCAGATTTCTTTA |
| 31 | lncBr_013 | TAGAAAGGTAAGTAGACGTTTTTTAGAAGTGTGTAGTGAAAGTGCTTTTGGTCAGGAGGTTTTTGCGGTTTGTGTTGCTTTGGGTTTGAGTGTGCGATGTTGAGAGGGCAATGAGGGATGTTACTAGAAGGACAAAAGTTGGGGTTCAATCTTAAAGGGATAGGTCTTTTAAATATGTACTGACCCTCAGGCCATTGAAGATGACTTTTTTCTTTAGTAGAACACAATTAGTTTGCATAATGGTAGTCGGTCATCAATTGTTTTAGTAATTAAAAATAAAAACATCCAAATAAGTCTGAATCAATCCTCGTGACTCTTGATGATACACTGAGGTCTAATTGGTTAAAAAGCGAAATGGTTGCTCTGTGTAAGAAATTAAATGCTATTTACAATGGTAAAATCTAGCCCTTGGTTGCTTGATGACTAGGGTTGCGTATTAAAAACCGG |
| 32 | lncBr_014 | CTAGTGGTTTTAAAGCAACAGGTTTACTCATTTATTTTAAGTCAACTAGTGATTTTAAAGCAACAGGTTTACTCATTTATTTTAAGTCAACTAGTGGTTTTAAAGCAACAGGTTTACTCATTTATTTTAAGTCAACTAGTGATTTTAAAGCAACAGGTTTACTCATTTATTTTAAACAAAGTCCAGTTCGCAAACACCACATGTTTACAGACGGCTCTCCTGTGTCCGTCGTGCATATTAAATCACACTGAAGAAGTAGTTTAACAGCTCGCCTTTAAATATTTGTTTGATTATCGAAATCAATGCGCATAATGTTATGGTTCTTTTAGGAAGAAGTCATATAATTCAACAGTAAAACTCCTAACATCATTCATATCTACAGATTTAAAAGTGGAATTATTATGGATGAGACCAAATTGTGATTAATTATCATTTGTTGCAATGTAAGTTAAGAGTAAATGTGAAAGTTGAGGGCCACTGGATGGCAGCAGCAAATCATTCTGCATGATGCTTTTATTTTATGTACATTCATTTCTGTTTAATTTAGTGAGTACTTCTTTAAATTTAATGCACTTAAATCAAATCGTAGTCTTTA |
| 33 | lncBr_015 | GCAGTAAAAAGTTTTTTTTTTAAGTCAACCAAAACGCTTTAAAATAAACGGGTTTACTCACTTTTTATAAAATTAAATTAAATGAATAACTTTTTACAATGCAGTGCCTTTGTGATCAGCTTTGGGTTTAAAGGAATAGATGACCCAGAAATTAAAATTTGCTGTTAATTTATTGACCCTCGGGTCAGTCAAGATGTAGGTGACTTTTTCTTCAGTCAGATTTTTTGCTGAAACTGTGATTCATAACATGAATGATAAAACAAAAGTAATATCCACTTATTTGGATGTGCATTAAAGGTGATTTAAATAATCATTTGATTATTGTCAAAATCCACAGCTACTCATTTAGTTTTACCTGTACACTGTAAAACCCAAC |
| 34 | lncBr_016 | GCACTGCAGTACATTTCGTTACAACTATTGATAAAAATAAACACAACATATTCTATCAACGTTCACCATTTATAAACTCACCTATCATAGCTTTATTCTGTGACGCATAACTATGAGCACCAAATCTAAAAACTTGCACAGTCACTTGCTCACCAAACGAAAATATCGTAAACTATTAGCCTAAATCTTGATAATGCTACCATTAACCTGCTTTCTGGAAAACAAGCTTTTGTTCACTAAGGATTTAAAAAAGATAATACGGTAACACTAAACAATACGGTAAACACTTTACAATAAGGTTCATTAGTTAATGCATTTACTAACATGAACTAATCATGAACAACACATGTACAGAATTTATTAATCATAATTGAACATTTACTAGTACATTATTAACATCCAAGTCCATGCTTGTTAAC |
| 35 | lncBr_017 | TGTAAAAGTAATTATTATTATCAGTCAATATAAGTGATAATCGGTATTAGCCAATAAAAGCAATTATCGGTATCAGCTAATAAAAAGCAATTATCGGTATCAGCTAATGAAAGCAATTATCAGTATCAGCCAATAAAAGCAATTATCGGTATCAGCCAATAAAAGCAATTATCGGTATCTGCTAATGAAAGCAATTATCAGTATCAGCCAATAAAAGCAATTATCGGTATAAGCTAATGAAAAAGCAATTATCAGCTACAGCCAATAAATGTAATAATTGGTATCGGCCAATAAAAGCAAAAATCGGTTACAGCAAATAAAAGTAATTATCAGTACCGGATAATAAAAGCAATTATCAGTATTGACCTGTAA |
| 36 | lncBr_018 | GCGAGTATTCAAAGGGTATTTAAAAGAACTAGGGGAAATATCCTTGAACAGTCTTAAACTAAACTAAATGTACATGAAAATTCTGAATAAATTTTAGATTTGAAGAGAGATTTTGAGATTTTTTTTTTTTGTTGTTGTTGTTTGTTTGTTTTTAGAATATGCAAATTGGCATTATTTGATGCAGAAATACAGCATGTGATTGAGCTATTGTATCTCCTAATGGATTTAGCAGTGTCCACTACTTTGGGTGCAGATGTCCCATTTTAAAGACTTTTTTTCTGTGTTGTTTGGTAGGGTAGAGTGAGTTTGGACTTGCTCAGCTGTAGTGTGGACATGCAATAATGTTTTTTTTTTCAATCTATTTAAAGTGTAA |
| 37 | lncBr_019 | GCTTTTAATTCATGAAACAATCATCATTAATTAGTCACTAATTAATATTGGTCATGTTTATGACAGATTTATGACCAGTTCTGTAATTTCGGTAATGTCAAGTTGTTGTTACAAAGACAATATGTGATGTATTTGATTAAGTCAAGTTGTCATAACAAAGACATCTCAAGCAATGTCCCCTTTTGATTAAAAATCACATTACTGAGTGAATGACATACATTAGATTTCCTTCATATTCATGACATTTGAACTTATGGACACACCCTTCAACTAAAGTTTTACTTTTACCATTATGTCTTATCAAATGTATTATATATCTGTCTG |
| 38 | lncBr_020 | CAGAACACGCTGGATGAACGCAGCGGTGCCGCAATCAGCGAAATCCCGCATTCTCCATTCACATCCACACCGCGTCTGCTGCTGCCTCACATAAACAGAAGGCTATAGCATTTGTCTTAGACAGCAACTGACAGCATACAGCTGCAAGAACACAGCTGCAAGAACCGCATCGCTTGGACCTCACAGAGGTTTGATCGCATTTTAAGCTCCAATTCAATCTGGAGATCGTTTGGTTTTAAACCGAGCGCACGCACGATGGTTCATCTGCATCCCCGCCGAAGAAAACTGGTTTGGCTGTTGCCCGGTCTGCTTGGAGTCTGG |
| 39 | lncBr_021 | GTTAGCAGCGTCGCCATGGAAACGACTCCCTCACCGAGATCACGCCTCATCAAGGGCTGATCAGGGGCAGCTGCTGCTCGTTGGGAGGAGAGTATTTAACAGACGCCGTCCTCACAGACGACGAGCACGATGACAGCAGGCTACACGCTGAGTTCTCTCCTTCCACTGGGCACCCTGATCAGCACTTCACCCTGCACATTCCTTGGACACTGAATTCACCGCCCTGCACATCCTAGCACACTTATCCTTTGCTCCACTCCCTCAATAAAACACCCTACAGGGATTTATATCGCCTTTCAGTGTCC |
| 40 | lncBr_022 | GTTTGATCAATTTGTTTTGAAGACCTCTCTCGAAAGTGGAAAACACTTATTTTCTCTCTTTACCTTTGCAGTGGATTTTCTTTCAGGCGGCATGTGGTGTTTAAGTCAGAGGTCAGATCTCTCCTGCTGCCCGTCCAGTGAGGTCACAGCATCAGACTGCTACGCCTGACTGTCTTCTGGAGCCACCATCCATAATCTAAAGAACCCAGGGAAGAGGCCAAAAACCTGTCAGTACGACACCGGCTTCAAACTGCGAAGCTGAGGATGCCATAGGGAACAAACTAAGGCAACCGAGCAAATATGATATTTATACAAATTTTCAGATTAAAAGCAAG |
| 41 | lncBr_023 | CTGGTCATCTTTATTCTAATGCTTTAAAATAAGTCCCTTCTGCTCACTTCAAGACTTTATTTATTTGATCTTTAACATTATCAACATCTTGACAGCACCGCAGAACACGTCAGCAACACCCTAACAACCCTCTACAACACCCTAGCAACCATCCAAAACAGGAACAACCATGTGGAACAGGCTATGGACGCCCTAGCAGCATGAGCACACGCCAGCAGTGCATCTACAGGGGAAATACACGCGATGTTTAAACTAAACAAGCGTCATTAGAGTGAATGAAGCTGTCAGACTGAGGTGTTCATCTGCTTACAGTGGAGAGACAGCTCTACTGCTGATACACGGCAGACAGAACACACTACTGCCCCAGAGTGTGTGTGAACGGGAGAGAGAGTGAGTGAGTGAGTGTGTGTGTGTGTGTGTGTGTGTGTGTGTGTGTGTGTGTGTGTGTGTATGTCTGTGAGATTGTGAGTATGTGTGTGTGTGTATATGAAAGTGGATGCAAGTGTGTGTGTGTGTGTG |
| 42 | lncBr_024 | GGACAGTGCAGCGTGGTGTATCCCTGGCTCAGTACCATGTCTTAAACAGTGATGATATTTTCATCTATATGGGCAGTGTGTGGACGACCACTCCCTGTCACATGACATACTGATCCAGTGCAATCAATTTTCCCCAACAAGTAATTCAATCCACCTTTTGTACAATTTTAGTCAGAAATTCACAAAGAAAACATGAAGCACTGTAGACTTTCAGTTTTCGTAAACCTTTGATCAAACTCGAAAACAACTCGCACTCGTAGCAGTGCAGTGTGGCTGTATATTGGCACTGGTGGGAGGCGTGAGTTGACTCGAGGCTGCAGGCC |
| 43 | lncBr_025 | AGAAAAACGGGCGACTAGAGAGGAAGGCAAAATAAAGTGGAGAGAGCGAAGAATTAATGAAAGAGGCAAAGTGAGGGGGGATTTTAGGAGGTCTATGGATTCTGCTCTGGACTTTGGATTTTAAGCCGGATCGAACCTTGTGCTGAGCCTCATTGGAGGGCGTCCTGCGGATGCACTAGTGCGCGTGCGAGGGGTCTCTCTCTGCCCGCTGGGGAGGCGTGCCGCACAGACGGACCGCCGGCGTGCGCGTGTGTGTGTACGTGTCTCTGCGTGCCGTTTCCCCTTATGTCCTTCTTTTGGTGGCTGTGTGTGCACCAGGGCCC |
| 44 | lncBr_026 | CGTGCCTAGACGATTCAGAGTGTCATCTTCAACTTCTCCGCACGTTCACACATACTGTACATGTGTGTGTGGACAGCGACGGTAAATGAATGCTGCCCGGAGCGCGCACGAACGCGGTATGACGCCAAAAAAAAAGCTGTCTAGGTAGGCAGCTCACTAGATTGTCTGACGTCTTTTCCAGCCACGTTGACAGTCCCGTGGTAATAGAAACAAACAATAAGGATCCCGGCGCTGTTTCCTACATTGGCAGAAAGCTGGTTCGTGTTGTAGTTGTCGCGGCGCAGTCGGTTGTACGCAGCCGCTGCGTCCTCCCTCTCATGCCGATTGAGGATGTGAGCGCAGCCGCCGAGATGCTGGATCGCGGTGAGACCTGAAGCAGCCTAGAG |
| 45 | lncBr_027 | GCGCTGTGTAAGTGTGCGCGCGCGTTCCAGGGATGACGATGACGGTGTTTGGGGAGCTGTGGGTGTCTTTAAGAGCTTCTTTCTGCTCACTGCTGTCAGGATAGAGAGGCACGCGCCACCGAAGTACTCACGAGAGCAGAGAGACGCGAGAGTCCTGTTGGAAATACGTTTATCTTCATACACATCTAGGATATACTGTCTTCGGGAAACGTGGAGAACATGCAGTTGCCAAAGTCACGCGCTTTCGCTGTCAGTCTGACGCTTCTATCCGTCCTCAGCTCGGCAATGTGCGGCAGTCAG |
| 46 | lncBr_028 | GTTCAATCAATCAATCAATCAATCAATCAATATACACTACATCACTATTGAGCTAGATTAGATCCTGCTCTGTAGTATGAGCTAATTTAGTTCCCCAAATGGCGTTCCTAGAACTAAATTGTTCCCACTTCCAATACACACCAAAGAGCGGGTATTTCTGCCTATAGTTTGTAAATAGGTTCCTTGGTTGGGAAAGTCCTATAAAACTAAAATTATTTTCTTAACTGCTGAAGCACAAACACATCTGACAGTAGTTATAGGAACTATGAAAATATTCCTTCATTAGGTTTAATAGAA |
| 47 | lncBr_029 | CAAGAGGGCATTTAAGAAGAATCTTTCTTGGAAAGATGAGGCCGTTTCGGTGATACACAGCTATAACTCTCCTCCGTTCTGAAATCAGTGTTGGGTTTCTCGATGATGGAGCACCAATCACAAAGCCATTGAAGTCCTATTTCTGCTGGAGTCAAGCTTTAAGTTGAACAGCTACTAGGAAATACTGCAAACTTCAACACTGACAGGTGAAAATAAAAATATGTTCTGGCGCACTCTTGAAAATCCCATTGGAAATCAGTTAGGAATGATCCCGTAATCCAACAGCTTCCTTAATTTCCACGTGTGTCCACCCAAAATCCAAACGTGCACTGTGGAATCCTCAAAATTGATGATGTTAATCAAAAGTGGAAGCCATCATTATCCCAATCTCTCCCGCCTCGTTGTGCCTTCTTGACCTGCACGGCTCAGATCATGCGAGTTCATGTTGACCTCAGT |
| 48 | lncBr_030 | CTCAGATGAGCAGCTCTGGATATACACAAGGATTGTGTGTCTTGCCGATCAGGGGACGCCTGTGGATCATGTTTCCGTGAATACTGACAGAAATAAAGCGTATTTCACGTATCTGCTCTCTCACCGTGTCAGTCTGTATGCATTTGCGCGCGCACTGGGAGTTTCCGTCAATCTACGGATGAGACACCGCTGGAAGTCAGAGCTTCTGTGGCACAACTTTTCTGATTTATTCGTCTCAGAGGATCGGCAATTTTGTAAAACG |
| 49 | lncBr_031 | GTGTACTTATATATGCTGAGCACTCTATATATAAAATCAAATTAGTGATCATTTATTACATATATTATACTGTCAAAATAAGTGCACCCGTGTTCTTGTTCTTTGTCTTAATCTTTGTATTTATCATAATGTTTTTTGCTGTGTTAAACATTAAGTCACTGCAGTCTTCCTTGAGAGCATTTATTCTTATTTATATGTATGCCCAAAATTAGCTTGCCAAGTAGTACCTGACAAGTCTTAAATGCATGTCCATTTTGGCAGGTAACGTTATTTGGAATAGCAGAACAATTTATGTTTTTATTTAAAC |
| 50 | lncBr_032 | CTTTTCTTCTTTTTGCAATAAATGGTTTTATAAAGCTACACCACATAAAACAGAAGTCAAGATCCATTACGTTTGTAATATTTTTGTTATGCAACAATGTTGATACACATTTCCATGTACTGTATGTAAAGTTACGGCAACAGTGGAATTACTCAGTGATTCACAGACAAGTGTATGGTTTCCTGATCACCCTGTTTATTAGACTTGATTATTCTTTCATGGCTTGATTGATTTCGTTGTTGAGTTTGTTTGTCATATAATGAGTGAATCACTAAGTGCTTTTCTTTTCCTTATATATGCCATTTTTTTGTTAGATTTGTCC |
| 51 | lncBr_033 | CCAAGCTTTTTTTTAAAGTAACTGATTTCAGTCATAAACAGAAGATGCGCTGCTTAAATTAAACAGATTTTTAACTAATCAATTTGTATTCATTCATGTTTTCAATTGACAAAATACTTTTTTTAGACACATTTTAACTAATCAAGAACAAATATATTGTGGTAAATGTAAAATTTTTGCAGTGCAGACATTGTAGACAGCATTCATTGTTAGACTTTATGTTAAGGTGTCTTTGTTAAATTGTAACAATAGATGTCTCGTCATCTTTGCCACCCAAATGTACACAAGGATTTATTTCTTTTGAAACTTGCTGCATTTTTCCACCATGTTTAGATTGACAGTATATTATCGGCCTTGATAATCATCTGTTTTTAAACCGATTATTGGTCAATATATATCAGTGAATGCTTCATTAAAAGTGCTTTAATACAGTTGCTGTGTTCATTATTGTACACAGTCATTGATTATAGTGTTACCTGCAAGTGAGGCGTAAAAAATTTCTCATGTCAGTTTCTATTGTCATAATTATTATGTGATAAATAACAAGTCATATTTGGTGCTTCTTTTCCTTTAACAAGTGCTTTCTTCAAATAATAATGATAATAAAATATTC |
| 52 | lncBr_035 | CTTTTTCTTGATCCATCTGCTAGAGCCACAAAACCTCAAAAAAGTGCCAAAGAAACCTATTGAGGTGGATATGCAAGCCCCCTGGGCCATTTCCAGGTGTCGAAACGTTGAAATTCCACTGCCCACGAACAGCCTATTTCATACATTGGGATAGATTGTCTGTGGGATGTCCATATCTCAGCTTCTGGACACAATAGAGAGCCCAAACCTGCGTTTCTGTGCTAGTCTGGACACGCTCTGTCTAGGG |
| 53 | lncBr_036 | AAAAAATAACCTGTCCATTGATGTATGGAAGATTTGTGAGGTAAAACTATTTGGGTTTTAAAAAAAAAATCATGGACTATTGCTTAAACATAACTGGATAACGTGTTCTGGATCACATTTGTCACAATAATAAAAACTTGAATATTCACGTGAAGCTGAGGTTAAGATTTGAGAATAATTTTAGACTATATACTGTAAATCTGACCTTTCTTACCCTTGTGAAAAAGAAATGTACTTATAGTATATGTACTGGATGTACTTTAGTGTACTTCAAAAATGAGAGTATACTTTCAATTAACCCAATTAGCCCGCCTTTGAATTTTTTTTCTTTTTATAATGATTCTCAAATTATGTTTTAACAGAGCAAGG |
| 54 | lncBr_037 | GCTGAACAAGATGGCTGTGCGTCGAGTAACGGGCGGCTAAAAAAACGGCGCTAATAATCGCACAAAACCCGAATAACAGCGCATTATATTGCAAGAGGGGGATTGAACCATCCCCGGGTTGAATTAGCTGGAAGATTCGGAATAGAAGCTGTAGTATTTTTTTGTATTTTTGGGGTTGTAGCTTTGGTAACTGGAAAAGGGGAGGTTTCTCAACGCATAAAATCCACTAAAAACGTTTTAACAGCAGTTTATTTTTGTAAAATAACAGAAAAGCGCGCGGAAAATATAAATAAAGAGATGTCACTGCTCTGTGTTGGA |
| 55 | lncBr_038 | CTTTGCCCTAACTGGACTTGTGGATGAGCAGTGCTCTAGTCCCGCTCCTCAACTCAACTAATAAATGACTGGAAATAGTTATAAGGCGCTGTCGCTCGCGTTATGACAGACAAAACTACTCTGCCATGTCTCTGTCTGTGCGGAATACATAAAGGCTCTGCCTTGAATTAATCACTCGCTCTTTAATGGATCTTTGAGGATCGCGCTTGACGCAGTGAGGAGCTGCAGCTCTTTGTATTCCGAGGCCAAGAGGAACCTTGTGCTTTTTTTGCCCCCGTCCGGTATCTACTGCCTCTTAGAAAATGTCAAGGGGGTACATGGTGACATAGGGACCCTGGTTTGGTTACGGAGAGACTCTTGCTTCGCTCTGCGAAAGAGTTCAAGACTACAGCGGCACAGATGCGGTGCGTTTCTTTTCAACCAAGCGTCAACAAAGATCAGTTCAAGGGGAGATTCCGAGCATCATTAGTTCGGT |
| 56 | lncBr_039 | TGAATATTTTGTCAATTCAAATGACTAGCGGCTTCGACCTGGAAACAGTATTTCATACGTCACGGCTTAACAAGCGGATGGGTTTACTCAGCAATGTTTACTAATCACTTTTCTGGATTTTGACTGTGGTTTGCATATCTTAGAATGCTCAGCACTAAACATTAAAGCTATTTAGATGAGCCTGGTCACTCGCCAGATGTTGCTTTTACAGCTGGTTAGAACAAAAAACTCAATGTAATCATGCGTCACATCACGTCTGGGTAGGACACCGTGTGTTTTATGCAGTTGTTTTTGTATCTCGGTTGCATGTGTGTTGCATTTCTG |
| 57 | lncBr_040 | ATGGCAGAGTTTTCACTTTGGCTGAACTATCCTTTTAATTCTCAATACTTGCATTTAATACTTGTACTTATAAATGTTTAGGGATTCTTTAAATGCCTTATAGTTTCACTGAAATCCAGTTCACACATGTGAAAGCAAGGGTAACACTTTACAAAAAGGTGAACATTCGTTAACGTAATGATAATAAATCATGTTCCCGAAGCCATTATTAGTTCCAGTGCACTCGCCAGTGACGTCTTCATATCATTTAATAAAC |
| 58 | lncBr_041 | CTTGTGTTTGTGTGTGTGTGTGTGTGTGTGTGTGTGTGTGTGTGTGTGTGTGTGTGTGTGTGTTTGCTTTCAGACAATGAAATTGGAGGAGGTGTAGTGACCTCTCATCAGTCGTCTAACATTGAGGACCACGGGGAAAACAGAGGTCTGAACCAAAGACTAATTCAACCACCAGTTACATCAGATGGAGGTGGATGTAGCAGATGAGAAGCGCCATTGCACTCGCTCGAAAGTTCCCCTGGAGCCGGCCATTCAAGAGCTGTT |
| 59 | lncBr_042 | ATATTTTAAGAGCCGAGAGTGAGCCGAATACTACAGGGATTATATCAAAAGCAAGACTTCGACATCTGGGGGCGACGCGTCAAACTCACGCGGTGCTGAAAGCGGCGGCGTTCGCTACAATATGAGCTGTTTTGAGCCTCTGATCTGATGAACGTGCGTGAGGGGACTGTTAATGTGCCTTTAAAAATACGGAACCATTAGGCGATGTGTGTGAACCAGAGGAAAGAGCCTCAGGTGAATAGAGGATTTGTCAGAGAGGCGTGTGAGCGTGTCTGCTTAAAGCAGTCCTCAG |
| 60 | lncBr_043 | CACACACACACACGCACGCACGCACGCACGCACGCACACACACACACACACACACACACACACACACTCTTCCTCTGGTATTAATGCTTCCTAAACCAACAGTACTGTACGCTTTCAGTTCTTCAATGATGAGTTCAGAGTTTGTTTTAAAGCAGCGGTTGTAGATTTGTAAACGTGTGTGTTCTGTGTTTAGTCTGATTTGAAGAGCTCGTAGTCGTCTCTCTGGACCAGCTGAATGTGTG |
| 61 | lncBr_044 | TATTTATTCATTTCCCTTCAGCTTAGTCCCTTATTTTTCCAGGGGTTGCCACAGCGGAATGAACTGCCAACTATTCCGGTGTTGTGACATTTTATCCTACCCATCAGATTATGTTGAGTATACCAACACTGTATTTGAATAGTTCTGAGGTTGGGGTTAGGGATTAGGTAGGTTAGGGCAAGATTACAGCTTCATATCACTCTACTCCACATTCAAATTTACGTTGGTAGCAAAATTTGATGGGTAACAAATTGAGTAATCAAAACGTGCTACCTACTTTTAGAACAGGAGGTAGGACAATTTGACAAGC |
| 62 | lncBr_045 | CTTCAAAGCCAACTCATAAGGCAAACCCCGGCCGGGGCGAGAGAGGAAAAGAGTCGATCGGTCCGTGCCCACCACAGCGATGCGGGAACCACTGACCTCCGGCTGCTGATGGTCAGAGCACAGTCAACAGGTGAGTAAGTAAGTGGCGGCACTCAGAACATGCAACGATGAATACAGGAGGGATGATCGACAACCAGATCGAGACTAGACGAGACAAATGAGGATGAGATCTGATGTAGAGGAAAGACTAATACACGGATTGCTAACGCACAGAGTAACAATAATCTGACAACAGGAGGAGAAATAAGGGAGGTAGATATAGGAGTGTCA |
| 63 | lncBr_046 | CAAAATTTCTGGAGGTTAAAATTTCGCCAAGAACTTTCTAGAGATTTTTAATAAGGTTTACATCAGGACTCTGGGTCTGCCATTTCATTATTCAGCTTAAGGAGCTGCTTTACATGTTTTGCTGTGTGACAGTGCTTTCATTGTCCTGCATGAAAATGCTGTCTGACTGTGTGATGAACACAGGGAAGAAAACACACTACCCAAG |
| 64 | lncBr_047 | GTTTTGAAGAATGCATCCGTGTCCGAATCATTGGGTCCGTCCTCCGGCGCTTCAGCAGAATTCATGTTTTCCTCTTGTATCTCACAGCTCAAAATAACAAGAAATATATCCAAATTCAGCGCTCCATCATCTCAGTGTGTCTGTTTTTGACGGCACAGCCCTCAGTCAGCGCGACAAAGCACAGCTCTGCATTGCCTTCTATTGTCTCCAG |
| 65 | lncBr_048 | TGTGTGTGAAAACTGCCAGTTTTTATAAACAATAATGATAATTTTTGTAATTAGTTAACTTTTTTTAAGAAAATGTTTATACTTGGCTGTGATGGCTATGATCTCAACACCTTAGAACATAGATATTAACATTTGATGTTGCCTATGCTGTTGTTGTCTATTGGCGTAGATATATACACTAGATATCGCATTTGGATCCTGATCATGC |
| 66 | lncBL_001 | GTTTAACGTGGGCTTTCTTGTTTAAAGAGTCACTGATACTTTGTTATATACAACATTATGTCAGTCCCTAATAGGGCAGAGTGGTGACAGTTGCAACACTTTTTTTTGCATTTGCTTCAGTTTCTTAGAGACAGTTATTGTGGAAACCCAAACTGTTAATACTGTTAAGATAACACATCTTTTGGACACACATTGCTATAATATGTGTACATATCATTAGTGTAGTCCTTCATATACACACGTATACTCAGTATGCTTACCTTTTCCACAAGCCGTTTGGGTATTACCACTTCTGAGGATGAATAATTGAGTGTACCCCTTGGATATTAGTCAAACATTTG |
| 67 | lncBL_002 | GTTTCACTCCAAGAAGGCCAAACAACACATTCAAAAACCCCATAGAAAGTGTCGCACCGAATGAACAACACAGGAATGAACCGAGCGCATAGACTGACAATTGACAGTCAAATTCGTTGGGCAGAAATTTAAATGCGAAAGCTCCTATTGTGACACCAGGAGCCGCGTAACATGCGAAAAGCACATCTGACCACGTCCTGATGCTGATAATCGACCCAAAAACCAGAGCTGAAGTCAAGAGTAATGCGAA |
| 68 | lncBL_003 | AGTGTACATTCATAGCAATGCAATGTATAACGGTGTAAAAATACTGATCAGTTTCATATCAATGCAGAGTGGCACGTAATAGAAACAGACCATCTAAAATCTGTTATTCCCTGAAGGTTTAGTCTAGAAATAGCTTTTTTTAGTTGTTCCCTGCAGTGGTCTGGAGAAGGTCAGTCTGCTTCACTTTCTTTCTGCCTCCATTCGTTCATCCAGATTTTTGACGTTCTTCAGGAACGTCATGACGTCGGTTGCCTCGTATTCCTCACATGTTCTGCATTCGCACTTTCGGTTCGGGCAAGGCTT |
| 69 | lncBL_004 | CGCGTGTCCTCAAATCTGCCTCTGAAACACAATGGGATCACAATCACTGCCTCCTTTGGGACAGCAGAGAACAAACTACACAGACGACAACAACAGAATTTAGGTCATTACAGAGGCATATCGACTCCTGCTGAATCAGCTGCAGCTTAAAAGAACAAATAGATGTAGACAGAAGAGTAACGGGCCCTGAGGTTTGACTGCATTACGTTGTTGTGTGTAATGGGAGAGTGTGTGTTGGACCGCTATGAAGAAGATGGGTTTCAGAGTTGTGAAGGGTTTGACAACTGGGCCTCATTTAGC |
| 70 | lncBL_005 | GATTGTCCTCAGGTGCGATGTGTTGAGGTTTGCACTGGACAGCAGAGAGGAGAGCTCAGTCTACAGCAGGGAGATCTGATCAATGTCATACAGAAAACTACTGATGGGTTCCTGGAGGGCCGTAGAGTACAGGATGGACAGCGGGGCTGGTTCTCGGCCTCATGTGTAGTAGAGATCACTAATGAACATGTACAGAGACGTCACCTCCGTCAGAGGTATCATGTCCTACAGACGGCCACACGCCTGCTGAAACAGCGCAACAGAGGCCTTGAGCAAACAACCACAAAATGCCTAAAATGAGAGACC |
| 71 | lncBL_006 | TCTGCTCCATCACTGCATTTTTCTTCAAGAACAGGAATATCTTGCGTAATCTTGCAGTAGTACCATCCACTGTCATTGGTCTTTGTAGAATTAATCTTGAGTATTTGCCAAGTGTACACTTTTTCTTTTATCCTGCTAGGAAGAGAGCCACAGTCTAATGTTGGTGTCTTGCTGTAATGCCAGCTCACTTTGTGGTTTTTGCTTGGCTCAAAAGTGCAGTTGATTTTGACTGATGCTCCTTCACAAACATTAATTTGTT |
| 72 | lncBL_007 | CCCCCTTTAGAAATCTTTAGTGTGCATTTACTGAGTGATTAAGAGTTAAGAGCGTTTGAATCCACAGATACCGAGGCAGGTAAACACCAAGTCAATGACGTACAAACCCAAGTTAACATAAGTCATGAACGTGACCACAAACTGGATGCTCCATACGCAGCTGTTCCCAGGACAGTCTTTAGGCCGCGGGTTTCCTCTGAAGCTGTAGATCGGCCACAGGATTGCTGCCGAA |
| 73 | lncBL_008 | TGATATTAAAGTGGTGTTTACACCATTTCTGCATTTTGAAATCTCGGCAACTGGAGCTGGAGCTGGAGGTTTGTAGTACTCAGCATGTTGTTGCAGTGTTTACAGTGCTGATCCTATACTTCTGGGTTTCTTCCCACCCAGCCTTGCTTTCGTGTTTTAAAATGGCGGGCTCATGAAATAAGCGGCGGATGTATGATAGGGCCCAGAGTATGTGTGAAACAGGCGCA |
| 74 | lncBL_009 | TCAATATGTGAAAAAAGATGGAGAGTGCTGTGGGTCGTGTGTACAAGTGGCCTGTATTTATGATGCTCCAGACAAAACCACACATGTCCTCCAGGAAGGACTGAGGTACAATTTCACATGCTTGAATGTCACGTGTCTGAAAAGGAATGACGTGTTCACGATCAAGGAGAGTTATAAAAAATGCCCTTCCTTTAATCAAGACAACTGTGTGGAAGTAAG |
| 75 | lncBL_010 | TTTTAATTCAGCTGTACATGTTTGCCCTATAGGAAGAGCATATGAAAGGCTGAAGAAGCCTGAAAGTGTCCTCTATTTGAGTGAAGCTCGGGTGTTGATGAAGTTCAGTTGATCAAGGATAATGTTGTGCTGGAACTGCCGATATCCGGTGACATTGAAGACGGGTTGTTCTCCAGGAATTTATATCCATCTTTAAACAGAACCTTGAGGATGTCCAGAACGACCAA |
| 76 | lncBL_011 | GGGCAGAAGAAAAAAAATACAAAAGCATGAGACAGAATAAATACGTACAGAACTTAAAATGATATCGACATGCAGCTGAAATCAAGAAAGCAAAGAAACATGGGATTTAGAGTGCATACTGTATGTAGTGTTCAACATGGGCATTACAGCCAAGGGTTGTGACAGGACGTCTGTAGATGTCTGACAGAAATAAAGTCTAACAAGTGAAACTGCTGATAGTGTTTGTTGAGTTATTTAAAGATCCTCTGTTGAGATTTTG |
| 77 | lncBL_012 | TCTGGGTTTGTTTTACTTGAGCTTTTGACTTCACAGCATTAGTTTTCCTCTTGTTGTATGTTGTGATTGTATGTTATGGCGTGTGTAACCACTAGATGGCACCAGAGGTTTGGAAAGGGTTATGTGAGATGAGAGAGTGCTAGAATAAGACAGTATCAGGCAGACTGAACTTGTAAATATAGAAGACACTCGTCCAACTCGTCGTCATTATTATAAGACGCAAACATGAGATGGTACCAGAGCATAGCAAGACAAATGCC |
