# Supplementary material for: Dynamic Expression of Long Non-Coding RNAs (lncRNAs) in Adult Zebrafish
Source: PLoS One. 2013 Dec 31;8(12):e83616. doi: 10.1371/journal.pone.0083616 (PMC3877055; doi:10.1371/journal.pone.0083616)
Supplement: Table S3 — FPKM values of 2,266 lncRNA transcripts across the five tissues of adult zebrafish (Transcript ID with prefix “U” indicates data from Ulitsky et al. (2011) and Transcript ID with prefix “P” indicates data from Pauli et al. (2012)). (DOCX) [file pone.0083616.s004.docx]

Table S3: FPKM values of 2,266 lncRNA transcripts across the five tissues of adult zebrafish (Transcript ID with prefix “U” indicates data from Ulitsky et al. (2011) and Transcript ID with prefix “P” indicates data from Pauli et al. (2012)).

| **S.No.** | **Transcript IDs** | **Heart** | **Liver** | **Muscle** | **Brain** | **Blood** |
| --- | --- | --- | --- | --- | --- | --- |
| 1 | P281 | 114.288 | 127.798 | 201.232 | 102.772 | 48.4675 |
| 2 | lnc_HLMBrBl_061 | 30.7856 | 309.45 | 28.0136 | 5.06401 | 80.2689 |
| 3 | U411 | 0.320684 | 235.801 | 23.1232 | 0.693413 | 0.866324 |
| 4 | U450 | 4.64307 | 195.61 | 43.5601 | 1.60094 | 22.0433 |
| 5 | lnc_HBl_010 | 1.29718 | 0 | 0 | 0 | 64.726 |
| 6 | P382 | 2.21379 | 0 | 0.489857 | 0.0297001 | 146.461 |
| 7 | U204 | 0.150512 | 49.8869 | 0.595802 | 0.0511734 | 3.45157 |
| 8 | U497 | 21.9371 | 54.6096 | 12.5052 | 8.40289 | 35.3766 |
| 9 | U515 | 10.8501 | 34.1821 | 42.6038 | 23.2836 | 7.43881 |
| 10 | U531 | 32.6931 | 2.11051 | 39.4986 | 3.97443 | 14.9528 |
| 11 | U330 | 41.9909 | 5.95211 | 47.5677 | 1.23807 | 6.7733 |
| 12 | lnc_LMBrBl_006 | 0 | 0.740387 | 2.67203 | 47.5405 | 0.15307 |
| 13 | U170 | 0.652265 | 0.545986 | 0.10035 | 0 | 29.3633 |
| 14 | U460 | 3.41142 | 3.46645 | 3.99253 | 2.73699 | 21.7334 |
| 15 | lnc_HMBl_008 | 0.732178 | 0 | 1.15212 | 0 | 18.3973 |
| 16 | lnc_HBl_017 | 0.448944 | 0 | 0 | 0 | 15.8672 |
| 17 | lnc_HMBl_018 | 1.82348 | 0 | 1.17943 | 0 | 16.6953 |
| 18 | lnc_HM_001 | 25.1505 | 0 | 0.740528 | 0 | 0 |
| 19 | lnc_HLMBr_008 | 27.494 | 2.44361 | 5.5268 | 0.782801 | 0 |
| 20 | lnc_HLMBrBl_051 | 2.82214 | 10.033 | 1.25649 | 0.179519 | 4.0116 |
| 21 | P63 | 3.81841 | 8.68518 | 2.92828 | 2.34649 | 2.49564 |
| 22 | lnc_L_001 | 0.176305 | 15.5315 | 0.419746 | 0.0330654 | 0 |
| 23 | lnc_LBl_001 | 0 | 12.6818 | 0 | 0 | 2.94454 |
| 24 | lnc_HLMBrBl_041 | 0.545238 | 12.3845 | 0.861144 | 1.06694 | 0.854511 |
| 25 | lnc_LBl_003 | 0 | 11.7278 | 0 | 0 | 0.430704 |
| 26 | P717 | 0 | 13.1327 | 0.116698 | 0.00743662 | 0.0183563 |
| 27 | lnc_HLMBrBl_031 | 6.56875 | 21.559 | 3.86528 | 6.89739 | 10.7966 |
| 28 | lnc_HLMBl_002 | 0.51887 | 24.9917 | 2.54552 | 0 | 1.62628 |
| 29 | P246 | 2.91562 | 16.7764 | 2.79966 | 1.39111 | 2.89146 |
| 30 | lnc_LBr_003 | 0 | 18.5185 | 0 | 0.107384 | 0 |
| 31 | lnc_LMBr_004 | 0 | 20.7822 | 0.678822 | 0.0677636 | 0 |
| 32 | lnc_Br_048 | 0 | 0 | 0 | 24.3698 | 0 |
| 33 | P612 | 2.68842 | 0.137793 | 3.1615 | 25.7492 | 0.0312063 |
| 34 | U619 | 0.117667 | 0.0717992 | 1.39265 | 12.7062 | 0.0326743 |
| 35 | lnc_HLMBr_005 | 0.304305 | 2.15426 | 1.09782 | 13.8703 | 0 |
| 36 | lnc_MBr_032 | 0 | 0 | 3.39623 | 13.8334 | 0 |
| 37 | lnc_LMBrBl_005 | 0 | 1.05278 | 4.42902 | 13.5744 | 0.418345 |
| 38 | lnc_MBr_013 | 0 | 0 | 4.82652 | 16.9928 | 0 |
| 39 | lnc_MBr_002 | 0 | 0 | 0.662891 | 15.6108 | 0 |
| 40 | P610 | 0 | 0.122543 | 1.53809 | 16.5218 | 0 |
| 41 | U306 | 0.0978345 | 0 | 1.78013 | 17.1325 | 0 |
| 42 | lnc_HLMBrBl_045 | 0.778306 | 16.0661 | 5.09104 | 19.5252 | 1.30103 |
| 43 | lnc_HLMBl_011 | 2.9094 | 4.94546 | 11.4738 | 15.3223 | 9.75879 |
| 44 | lnc_HLMBrBl_047 | 3.30355 | 3.82981 | 7.79256 | 13.6757 | 2.2208 |
| 45 | P25 | 4.20071 | 5.60966 | 3.583 | 8.06734 | 3.91801 |
| 46 | lnc_HLMBrBl_029 | 2.09175 | 9.00819 | 2.5767 | 12.6561 | 3.27678 |
| 47 | P109.P110 | 1.58391 | 9.84513 | 4.77501 | 9.53804 | 4.57454 |
| 48 | P994 | 1.55421 | 0.192938 | 22.1118 | 7.657 | 1.08812 |
| 49 | U628 | 1.96883 | 1.54332 | 19.1321 | 4.60232 | 3.95863 |
| 50 | U354 | 3.19751 | 2.68257 | 21.5834 | 3.42518 | 0.705825 |
| 51 | lnc_HLMBrBl_004 | 11.4135 | 12.6581 | 11.5224 | 3.29982 | 3.29465 |
| 52 | lnc_HLMBrBl_062 | 6.94152 | 4.56548 | 10.1002 | 4.44398 | 3.7655 |
| 53 | P1035 | 4.8553 | 6.92786 | 9.87319 | 9.28585 | 5.3905 |
| 54 | U616 | 12.5198 | 4.1245 | 6.51468 | 3.37485 | 4.53382 |
| 55 | P686.P688 | 7.46815 | 5.3614 | 6.04321 | 1.1814 | 0.693507 |
| 56 | U29 | 7.45715 | 3.93908 | 6.12991 | 1.31681 | 3.71848 |
| 57 | U131 | 10.2989 | 1.57832 | 1.6529 | 1.12389 | 0.790179 |
| 58 | lnc_HMBr_016 | 9.29213 | 0 | 4.30575 | 1.68583 | 0 |
| 59 | lnc_HMBl_009 | 7.63464 | 0 | 4.1388 | 0 | 0.36577 |
| 60 | U303 | 7.94506 | 1.84382 | 4.22347 | 0.688664 | 1.43735 |
| 61 | lnc_M_004 | 0 | 0 | 12.7134 | 0 | 0 |
| 62 | lnc_M_003 | 0 | 0 | 10.9187 | 0 | 0 |
| 63 | lnc_HLMBr_007 | 0.42058 | 1.39727 | 10.5635 | 0.283208 | 0 |
| 64 | P528 | 1.60107 | 0 | 17.849 | 0.103519 | 0 |
| 65 | U587 | 2.23873 | 2.65849 | 16.7338 | 0.337989 | 0.995184 |
| 66 | U289 | 3.40117 | 2.20712 | 8.55783 | 1.18698 | 4.9513 |
| 67 | U553 | 1.38704 | 3.47493 | 11.1876 | 2.92198 | 1.61618 |
| 68 | U600 | 0.358551 | 4.67876 | 6.05398 | 2.18564 | 0.0196944 |
| 69 | lnc_HMBr_010 | 0.904097 | 0 | 8.28493 | 3.4762 | 0 |
| 70 | lnc_HLMBr_004 | 2.62191 | 0.0718911 | 5.46546 | 2.88472 | 0.0082151 |
| 71 | lnc_HMBrBl_031 | 3.41708 | 0 | 6.68668 | 2.61085 | 1.08213 |
| 72 | U390 | 0.358187 | 0 | 4.93923 | 0.406634 | 0.668741 |
| 73 | lnc_HMBrBl_025 | 1.0896 | 0 | 5.58511 | 0.223467 | 0.693539 |
| 74 | U466 | 1.58366 | 0.740481 | 5.62868 | 0.974538 | 1.41658 |
| 75 | lnc_HMBrBl_029 | 1.50047 | 0 | 6.3852 | 2.04811 | 0.54222 |
| 76 | lnc_HLM_001 | 0.495722 | 1.69175 | 8.70027 | 0 | 0 |
| 77 | lnc_HMBl_019 | 1.40113 | 0 | 7.43325 | 0 | 0.163919 |
| 78 | U338 | 1.38272 | 0 | 7.24121 | 0.145298 | 0.59498 |
| 79 | P975 | 0.0036434 | 0.00814052 | 6.81342 | 0.0114696 | 0.00677766 |
| 80 | lnc_M_002 | 0 | 0 | 7.13721 | 0 | 0 |
| 81 | lnc_M_001 | 0 | 0 | 7.17625 | 0 | 0 |
| 82 | U75 | 4.28293 | 0.773937 | 2.96191 | 5.3312 | 12.1238 |
| 83 | U659 | 8.38468 | 2.41457 | 4.57167 | 3.77277 | 9.0682 |
| 84 | lnc_MBl_001 | 0 | 0 | 0.764917 | 0 | 9.45382 |
| 85 | lnc_MBl_004 | 0 | 0 | 2.41326 | 0 | 9.17236 |
| 86 | lnc_BrBl_001 | 0 | 0 | 0 | 1.5764 | 9.79455 |
| 87 | U516 | 0.377213 | 0.138165 | 0.254 | 0.0981895 | 11.4337 |
| 88 | U679 | 5.86212 | 0.847641 | 1.42312 | 0.9041 | 12.5264 |
| 89 | lnc_HLMBl_004 | 3.82354 | 1.62081 | 1.49697 | 0 | 8.72291 |
| 90 | U164 | 2.54803 | 1.00162 | 1.43625 | 2.75346 | 10.6564 |
| 91 | lnc_HBr_007 | 0.309924 | 0 | 0 | 7.8189 | 0 |
| 92 | U645 | 0.0026082 | 0.0753648 | 0.745784 | 8.37665 | 0.0309927 |
| 93 | P735 | 0.230406 | 0.465759 | 1.25556 | 8.18657 | 0.0998519 |
| 94 | lnc_MBr_012 | 0 | 0 | 1.73739 | 9.48173 | 0 |
| 95 | lnc_HMBr_009 | 0.275873 | 0 | 0.356407 | 9.45049 | 0 |
| 96 | lnc_MBr_018 | 0.145223 | 0 | 0.673873 | 9.28003 | 0 |
| 97 | lnc_MBr_015 | 0 | 0 | 4.63415 | 5.90216 | 0 |
| 98 | U309 | 1.81981 | 0.518662 | 3.85221 | 5.2685 | 2.23871 |
| 99 | lnc_HLMBrBl_035 | 2.50145 | 2.32182 | 1.49049 | 5.66666 | 0.910352 |
| 100 | lnc_HLMBrBl_055 | 1.59343 | 1.97056 | 2.60132 | 3.08906 | 1.64746 |
| 101 | lnc_HLMBrBl_012 | 0.4077 | 2.69652 | 2.37907 | 3.76375 | 2.06688 |
| 102 | lnc_HMBrBl_020 | 0.22754 | 0 | 1.39216 | 4.87222 | 1.43522 |
| 103 | P1127 | 0.547813 | 0.644183 | 0.997435 | 5.50272 | 1.1812 |
| 104 | P51 | 0.455736 | 0.334925 | 1.09304 | 5.28358 | 0.0592623 |
| 105 | lnc_MBr_010 | 0 | 0 | 1.59316 | 4.70774 | 0 |
| 106 | lnc_MBrBl_001 | 0 | 0 | 1.76088 | 5.2238 | 0.332631 |
| 107 | lnc_HBrBl_017 | 0.76638 | 0 | 0 | 5.64314 | 0.320518 |
| 108 | lnc_HBr_013 | 0.26993 | 0 | 0 | 5.3948 | 0 |
| 109 | lnc_HLBrBl_002 | 0.497911 | 0.296714 | 0 | 4.99696 | 0.0650909 |
| 110 | lnc_MBr_016 | 0 | 0 | 0.830235 | 6.68983 | 0 |
| 111 | lnc_MBr_027 | 0 | 0 | 0.479653 | 5.8613 | 0 |
| 112 | lnc_HBr_003 | 0.582174 | 0 | 0 | 6.3056 | 0 |
| 113 | lnc_Br_046 | 0 | 0 | 0 | 6.01454 | 0 |
| 114 | lnc_Br_047 | 0 | 0 | 0 | 6.42348 | 0 |
| 115 | lnc_HBrBl_015 | 2.66638 | 0 | 0 | 3.80234 | 1.03955 |
| 116 | lnc_HMBrBl_026 | 1.17333 | 0 | 0.332482 | 3.19647 | 1.34137 |
| 117 | U559 | 1.76546 | 0 | 0.467001 | 3.1003 | 1.16316 |
| 118 | U487 | 1.59268 | 0.163064 | 1.83431 | 3.64684 | 0.086885 |
| 119 | lnc_HMBrBl_022 | 0.742613 | 0 | 2.61524 | 3.7004 | 0.708445 |
| 120 | U441 | 0.614552 | 0 | 1.01505 | 4.22382 | 0.704602 |
| 121 | lnc_HMBrBl_019 | 0.888603 | 0 | 0.986593 | 4.10721 | 0.679246 |
| 122 | lnc_MBr_004 | 0 | 0 | 1.14993 | 4.29676 | 0 |
| 123 | lnc_MBr_026 | 0 | 0 | 0.599907 | 4.34553 | 0 |
| 124 | lnc_MBr_019 | 0 | 0 | 0.718397 | 4.38703 | 0 |
| 125 | U646 | 0.0531853 | 0 | 0.564569 | 4.67487 | 0 |
| 126 | U508 | 0.0702031 | 0.0905251 | 0.50936 | 4.8524 | 0.0205705 |
| 127 | U575 | 0.0118754 | 0.0344141 | 0.299384 | 5.09767 | 0.0391611 |
| 128 | U250 | 0.0450696 | 0 | 0.501232 | 5.06637 | 0.0589753 |
| 129 | lnc_HBr_008 | 0.435474 | 0 | 0 | 3.86298 | 0 |
| 130 | lnc_Br_043 | 0 | 0 | 0 | 3.66242 | 0 |
| 131 | lnc_BrBl_004 | 0 | 0 | 0 | 3.90504 | 0.244016 |
| 132 | P694 | 0.0182261 | 0.0352152 | 0.56171 | 3.83482 | 0.0480818 |
| 133 | lnc_HMBrBl_032 | 0.0534722 | 0 | 0.48182 | 4.12838 | 0.0698508 |
| 134 | lnc_HMBr_028 | 0.131921 | 0 | 0.67467 | 4.09485 | 0 |
| 135 | lnc_Br_044 | 0 | 0 | 0 | 4.30845 | 0 |
| 136 | P1036 | 0.0686207 | 0 | 0.379009 | 4.49328 | 0.0180742 |
| 137 | lnc_HLMBr_016 | 0.119505 | 0.235876 | 0.351652 | 4.3252 | 0 |
| 138 | U15 | 6.82202 | 0.390353 | 1.09912 | 4.37468 | 0.058428 |
| 139 | P364.U214 | 8.25635 | 1.54626 | 1.58058 | 3.26699 | 0.518078 |
| 140 | lnc_HLMBl_008 | 8.19608 | 0.967919 | 0.803685 | 0 | 2.13807 |
| 141 | lnc_HBl_020 | 6.80582 | 0 | 0 | 0 | 1.26942 |
| 142 | lnc_HMBr_017 | 6.77208 | 0 | 0.626992 | 0.286267 | 0 |
| 143 | P813.P814.P815.U495 | 6.99324 | 0 | 0.655531 | 0.622547 | 0 |
| 144 | lnc_HBrBl_005 | 4.37934 | 0 | 0 | 1.84147 | 0.776582 |
| 145 | lnc_HBr_021 | 5.14862 | 0 | 0 | 1.9607 | 0 |
| 146 | lnc_HBl_019 | 4.74752 | 0 | 0 | 0 | 1.39441 |
| 147 | lnc_HMBl_004 | 4.69809 | 0 | 0.959307 | 0 | 1.10922 |
| 148 | lnc_H_011 | 4.82683 | 0 | 0 | 0 | 0 |
| 149 | U61 | 4.86632 | 0.18863 | 0.0698419 | 0.189964 | 0.16802 |
| 150 | U39 | 5.83824 | 0.227227 | 0.169174 | 0.593975 | 0.0503287 |
| 151 | lnc_HBl_012 | 5.77154 | 0 | 0 | 0 | 0.359801 |
| 152 | lnc_H_013 | 5.78179 | 0 | 0 | 0 | 0 |
| 153 | lnc_H_012 | 5.7949 | 0 | 0 | 0 | 0 |
| 154 | lnc_HLMBrBl_007 | 3.32228 | 1.48351 | 1.33855 | 2.16766 | 4.75553 |
| 155 | U87 | 3.47294 | 2.12002 | 0.824074 | 1.16331 | 2.59098 |
| 156 | lnc_HLBrBl_001 | 4.33894 | 2.20089 | 0 | 1.20291 | 3.84908 |
| 157 | P1103 | 6.62682 | 1.7985 | 1.27359 | 0.783325 | 5.42372 |
| 158 | lnc_HMBl_006 | 4.09573 | 0 | 1.39216 | 0 | 4.30565 |
| 159 | lnc_HMBrBl_004 | 4.45294 | 0 | 0.853116 | 0.916237 | 5.89757 |
| 160 | lnc_HLMBrBl_059 | 5.38142 | 2.02778 | 5.08741 | 0.545962 | 1.417 |
| 161 | P111 | 3.81843 | 2.48216 | 5.06941 | 1.28368 | 3.27701 |
| 162 | lnc_HMBr_002 | 4.06793 | 0 | 2.06755 | 1.60446 | 0 |
| 163 | lnc_HM_002 | 4.57641 | 0 | 3.45035 | 0 | 0 |
| 164 | U607.U608 | 1.69104 | 0.378473 | 3.14823 | 2.07349 | 1.27164 |
| 165 | lnc_HMBr_004 | 3.01568 | 0 | 3.24418 | 1.13348 | 0 |
| 166 | P1107 | 2.37254 | 0 | 3.35176 | 0.79613 | 0.895447 |
| 167 | lnc_HLMBl_009 | 2.9327 | 0.374875 | 3.84735 | 1.63776 | 1.70861 |
| 168 | lnc_HLMBrBl_009 | 3.93045 | 0.656852 | 4.16211 | 2.23691 | 2.05933 |
| 169 | P351 | 0 | 3.65379 | 1.91573 | 4.48452 | 6.78848 |
| 170 | U47 | 2.81188 | 0.580158 | 0.0870354 | 0.258424 | 6.32487 |
| 171 | P751 | 0.585749 | 0.39169 | 0 | 0 | 7.54877 |
| 172 | lnc_HBl_008 | 0.379782 | 0 | 0 | 0 | 6.27473 |
| 173 | U422 | 1.22034 | 0.154466 | 0.140798 | 0 | 6.55145 |
| 174 | lnc_HBrBl_018 | 0.37339 | 1.21987 | 0 | 1.63319 | 4.74849 |
| 175 | lnc_HLMBrBl_015 | 1.04559 | 1.29162 | 1.27671 | 0.313251 | 4.19048 |
| 176 | lnc_HBl_013 | 0.263535 | 0 | 0 | 0 | 5.28218 |
| 177 | lnc_BrBl_008 | 0 | 0 | 0 | 0.29919 | 5.76442 |
| 178 | lnc_HMBl_011 | 0.87459 | 0 | 0.482993 | 0 | 4.90532 |
| 179 | P14 | 1.08126 | 0.690605 | 0.472711 | 0.157556 | 5.59711 |
| 180 | lnc_HBl_015 | 2.07666 | 0 | 0 | 0 | 4.73398 |
| 181 | lnc_HMBrBl_030 | 1.78675 | 0 | 0.496829 | 0.217949 | 3.41382 |
| 182 | lnc_HLMBrBl_033 | 1.28686 | 0.294892 | 0.775776 | 0.827339 | 3.94716 |
| 183 | P119 | 0.255296 | 0 | 0.0551667 | 0.341616 | 3.28694 |
| 184 | lnc_HBl_007 | 0.53033 | 0 | 0 | 0 | 3.60408 |
| 185 | U468 | 0.801094 | 0.355242 | 0.145188 | 0.226074 | 4.01111 |
| 186 | lnc_HBrBl_013 | 0.527069 | 0 | 0 | 0.428497 | 3.96164 |
| 187 | lnc_HBrBl_004 | 0.977563 | 0 | 0 | 0.379636 | 3.74727 |
| 188 | lnc_MBl_003 | 0 | 0 | 0.903103 | 0 | 3.60744 |
| 189 | lnc_BL_008 | 0 | 0 | 0 | 0 | 3.87444 |
| 190 | P711 | 0.15955 | 0.233224 | 0.170992 | 0.0824478 | 3.90851 |
| 191 | lnc_HBl_009 | 0.413045 | 0 | 0 | 0 | 4.40661 |
| 192 | U257 | 0.0446147 | 0 | 0.0384143 | 0.0020959 | 4.40637 |
| 193 | lnc_HBl_014 | 0.11768 | 0 | 0.02592 | 0 | 4.54104 |
| 194 | U452 | 1.18993 | 6.56308 | 0.591766 | 0.284742 | 0.179606 |
| 195 | P38 | 0.624694 | 7.1806 | 1.40717 | 0.370447 | 0.307735 |
| 196 | lnc_LMBrBl_002 | 0 | 6.18992 | 0.550927 | 0.883332 | 0.203873 |
| 197 | P385 | 0.234429 | 6.73137 | 0.454535 | 0.429481 | 0.123248 |
| 198 | lnc_LBrBl_001 | 0 | 6.39364 | 0 | 0.20639 | 0.133941 |
| 199 | lnc_LMBr_002 | 0 | 6.53295 | 0.233026 | 0.189394 | 0 |
| 200 | lnc_HLMBrBl_052 | 3.10079 | 4.45052 | 4.01565 | 0.928999 | 0.559474 |
| 201 | P495 | 3.15121 | 5.13798 | 0.970178 | 2.01354 | 0.747347 |
| 202 | U624 | 3.8851 | 4.59848 | 2.12393 | 2.92717 | 1.84998 |
| 203 | P752 | 1.295 | 3.71776 | 2.52069 | 0.221851 | 2.52177 |
| 204 | lnc_HLMBrBl_060 | 2.46919 | 3.01349 | 2.72967 | 0.951188 | 2.55009 |
| 205 | U267 | 1.71964 | 2.8464 | 1.87586 | 1.14545 | 1.61357 |
| 206 | U489 | 1.94139 | 2.04473 | 2.58014 | 1.29355 | 2.01618 |
| 207 | lnc_HLBrBl_008 | 1.69746 | 2.68716 | 0 | 3.57167 | 0.544571 |
| 208 | lnc_LBr_005 | 0 | 1.48351 | 0 | 3.09666 | 0 |
| 209 | lnc_LBr_006 | 0 | 1.38718 | 0 | 3.92298 | 0 |
| 210 | lnc_HLMBr_014 | 0.156714 | 3.71024 | 0.431896 | 1.65199 | 0 |
| 211 | lnc_LMBrBl_004 | 0 | 3.77959 | 1.09565 | 2.44065 | 0.244016 |
| 212 | lnc_HLMBrBl_006 | 0.746779 | 3.65962 | 0.527021 | 2.79975 | 1.18712 |
| 213 | lnc_HLMBrBl_028 | 0.0877393 | 3.21551 | 0.625491 | 2.52454 | 2.61783 |
| 214 | lnc_HLMBl_005 | 2.06523 | 3.683 | 0.531054 | 0 | 1.671 |
| 215 | lnc_HLBrBl_009 | 2.45593 | 4.21111 | 0 | 0.573568 | 1.46421 |
| 216 | P828 | 0.930602 | 3.66002 | 0.508972 | 0.329297 | 0.1502 |
| 217 | P310 | 0.219753 | 3.98876 | 0 | 0.0679377 | 0.571823 |
| 218 | P527 | 0.0430092 | 4.52105 | 0.0530931 | 0.00935536 | 1.06331 |
| 219 | U676 | 0.267631 | 5.14389 | 0.764001 | 0.0946068 | 0.65923 |
| 220 | U345 | 0 | 5.10401 | 0.103713 | 0.00360473 | 0.0237347 |
| 221 | P932 | 0.100347 | 5.15064 | 0.2167 | 0.0209616 | 0.164808 |
| 222 | U2 | 0 | 4.50867 | 0.326652 | 0.375316 | 0 |
| 223 | lnc_HLMBrBl_057 | 0.0759257 | 4.60181 | 0.531225 | 0.21255 | 0.49365 |
| 224 | lnc_HLMBrBl_016 | 1.55601 | 1.79144 | 0.367748 | 0.31058 | 0.544571 |
| 225 | U136 | 1.35092 | 2.73551 | 0.687383 | 0.00981656 | 0.644313 |
| 226 | lnc_HLMBr_002 | 0.228821 | 1.84958 | 0.355969 | 1.21094 | 0 |
| 227 | lnc_HLMBr_001 | 0.690263 | 2.14671 | 0.57175 | 1.17915 | 0 |
| 228 | P291 | 0.580745 | 2.34243 | 0.928293 | 0.685374 | 0.381865 |
| 229 | P296 | 0.427622 | 2.4943 | 1.10117 | 0.97706 | 0.401776 |
| 230 | U242 | 0.0357558 | 2.73645 | 0.092038 | 0.0329568 | 0.043565 |
| 231 | P869 | 0 | 3.05265 | 0.0352055 | 0.00448937 | 0.169562 |
| 232 | P719 | 0.415533 | 2.62594 | 0.716497 | 0.150863 | 0.533551 |
| 233 | U302 | 0.0517667 | 2.73479 | 0.559676 | 0.259985 | 0.00168545 |
| 234 | P1059.P1061 | 0.19921 | 3.01273 | 0.440663 | 0.432111 | 0.29115 |
| 235 | P580 | 0.0680765 | 2.24231 | 0.337552 | 0.014582 | 0.167551 |
| 236 | P953 | 0.0038561 | 2.02807 | 0.038778 | 0.00447745 | 0.0172178 |
| 237 | U179 | 0.0356346 | 1.97409 | 0.119367 | 0.0260215 | 0.232968 |
| 238 | U367 | 0.106771 | 2.07536 | 0.10561 | 0.191985 | 0.182249 |
| 239 | U191 | 0.245098 | 2.27361 | 0.230167 | 0.866135 | 0.459114 |
| 240 | lnc_LMBrBl_007 | 0 | 2.08152 | 0.318367 | 0.697162 | 0.269542 |
| 241 | U80 | 0.031322 | 2.02911 | 0.27304 | 0.344653 | 0.411014 |
| 242 | P125 | 0.184768 | 2.21894 | 0.140473 | 0.445141 | 0.401606 |
| 243 | lnc_LBl_002 | 0 | 1.16797 | 0 | 0 | 2.28773 |
| 244 | U563.U564 | 0.145137 | 1.49782 | 0.408246 | 0.0432616 | 2.95028 |
| 245 | lnc_HLBl_003 | 0.562591 | 1.96393 | 0 | 0 | 4.2144 |
| 246 | lnc_HLBrBl_004 | 0.923675 | 2.82957 | 0 | 0.0895661 | 3.35188 |
| 247 | P446 | 0.21737 | 0.180476 | 0.393894 | 2.53504 | 2.56456 |
| 248 | lnc_HLBl_002 | 1.20872 | 0.963067 | 0 | 0 | 2.32217 |
| 249 | lnc_HBrBl_012 | 1.6187 | 0 | 0 | 0.130736 | 2.24463 |
| 250 | lnc_HBl_006 | 1.71655 | 0 | 0 | 0 | 2.59569 |
| 251 | lnc_HMBrBl_003 | 0.16957 | 0 | 0.463624 | 0.805481 | 2.81297 |
| 252 | lnc_HBrBl_016 | 0.445184 | 0 | 0 | 0.332012 | 2.6623 |
| 253 | P1098 | 0.143655 | 0.43418 | 0.166446 | 0.132805 | 2.61741 |
| 254 | lnc_BL_007 | 0 | 0 | 0 | 0 | 3.11441 |
| 255 | lnc_BL_012 | 0.0396172 | 0 | 0.0141107 | 0.00153836 | 2.71892 |
| 256 | lnc_BL_006 | 0 | 0 | 0 | 0 | 2.87578 |
| 257 | U473 | 0.160477 | 0 | 0.452445 | 0.132791 | 1.98489 |
| 258 | lnc_HMBrBl_023 | 0.455451 | 0 | 0.847146 | 0.116359 | 1.76314 |
| 259 | U472 | 0.734935 | 0 | 0.26412 | 0.155172 | 2.25271 |
| 260 | lnc_HMBrBl_010 | 0.762738 | 0 | 0.533953 | 0.498622 | 2.28104 |
| 261 | lnc_BrBl_007 | 0 | 0 | 0 | 0.804787 | 1.86137 |
| 262 | U291 | 0.048787 | 0 | 0 | 0.011151 | 2.00517 |
| 263 | lnc_BL_001 | 0 | 0 | 0 | 0 | 1.75717 |
| 264 | P874 | 0.0251564 | 0 | 0.217374 | 0 | 1.85139 |
| 265 | U280 | 0.0464407 | 0 | 0.296712 | 0 | 2.46592 |
| 266 | lnc_HBl_011 | 0.361229 | 0 | 0 | 0 | 2.2997 |
| 267 | lnc_HBrBl_001 | 0.294166 | 0 | 0 | 0.163949 | 2.26239 |
| 268 | P269.U149.U150.U152 | 0.12041 | 0.0296252 | 0.0463396 | 0.028607 | 2.24601 |
| 269 | lnc_BL_002 | 0 | 0 | 0 | 0 | 2.20977 |
| 270 | lnc_BL_003 | 0 | 0 | 0 | 0 | 2.2792 |
| 271 | lnc_BL_004 | 0 | 0 | 0 | 0 | 2.3119 |
| 272 | lnc_HLMBrBl_014 | 1.92985 | 0.773913 | 1.47341 | 0.935567 | 2.59924 |
| 273 | lnc_HLMBrBl_048 | 2.40486 | 1.29162 | 1.02137 | 0.313251 | 2.97389 |
| 274 | U484 | 1.41391 | 1.15493 | 1.03141 | 0.550623 | 1.427 |
| 275 | U542 | 1.69875 | 1.24091 | 0.576468 | 0.723422 | 1.93582 |
| 276 | U185 | 1.15674 | 1.31143 | 0.838267 | 0.468344 | 2.44607 |
| 277 | U142 | 0.887025 | 0.805113 | 0.895876 | 0.174278 | 2.1029 |
| 278 | U184 | 1.39208 | 0.867139 | 0.994106 | 0.209436 | 1.86664 |
| 279 | lnc_HMBrBl_027 | 0.0846152 | 0 | 2.2008 | 0.0805626 | 2.96556 |
| 280 | P571 | 1.50211 | 0 | 2.17797 | 0.765689 | 3.40995 |
| 281 | lnc_HLMBrBl_013 | 0.54364 | 1.10896 | 3.24455 | 0.262476 | 1.99699 |
| 282 | lnc_HMBrBl_028 | 0.970696 | 0 | 1.5236 | 0.126364 | 2.03239 |
| 283 | lnc_HLMBrBl_030 | 0.961571 | 0.867411 | 1.77292 | 0.745811 | 2.11707 |
| 284 | P998 | 0.943315 | 0.486075 | 2.62952 | 0.673195 | 1.98736 |
| 285 | lnc_HLMBrBl_020 | 1.41455 | 0.895719 | 2.20649 | 0.15529 | 2.54133 |
| 286 | lnc_HLMBrBl_024 | 1.83718 | 1.07637 | 2.27255 | 0.590632 | 1.91909 |
| 287 | U470 | 2.21188 | 0.86418 | 2.11709 | 0.451471 | 2.21388 |
| 288 | lnc_HBrBl_011 | 2.76832 | 0 | 0 | 1.33179 | 3.19926 |
| 289 | lnc_HMBl_007 | 3.74733 | 0 | 0.440091 | 0 | 2.29 |
| 290 | P911 | 3.78575 | 0.499917 | 0.073137 | 0.0844952 | 3.04343 |
| 291 | lnc_HBr_001 | 3.48007 | 0 | 0 | 0.209332 | 0 |
| 292 | lnc_HLBr_002 | 3.53581 | 0.244815 | 0 | 0.398657 | 0 |
| 293 | lnc_H_010 | 3.7963 | 0 | 0 | 0 | 0 |
| 294 | lnc_HBr_019 | 3.81602 | 0 | 0 | 0.317063 | 0 |
| 295 | lnc_HBrBl_019 | 3.09924 | 0 | 0 | 0.601454 | 1.15473 |
| 296 | lnc_HBl_002 | 2.3907 | 0 | 0 | 0 | 0.997894 |
| 297 | U101 | 2.53252 | 0 | 0.541378 | 0.402304 | 1.3091 |
| 298 | lnc_HMBr_003 | 3.11085 | 0 | 0.885803 | 0.191189 | 0 |
| 299 | lnc_HMBr_041 | 3.15185 | 0 | 0.764917 | 0.364817 | 0 |
| 300 | lnc_HMBl_002 | 2.93035 | 0 | 0.557947 | 0 | 0.727346 |
| 301 | lnc_HMBrBl_014 | 2.76908 | 0 | 1.14067 | 0.255855 | 0.75295 |
| 302 | P390.P391 | 2.31553 | 0.150842 | 0.218596 | 0.99559 | 0.188726 |
| 303 | lnc_HBr_005 | 2.91087 | 0 | 0 | 1.05093 | 0 |
| 304 | lnc_HBrBl_006 | 2.96278 | 0 | 0 | 0.858801 | 0.535612 |
| 305 | U274 | 2.94569 | 0.94426 | 0.0573918 | 0.264661 | 0.071184 |
| 306 | lnc_HBr_004 | 2.99343 | 0 | 0 | 0.457012 | 0 |
| 307 | lnc_H_008 | 2.84722 | 0 | 0 | 0 | 0 |
| 308 | lnc_H_009 | 3.08266 | 0 | 0 | 0 | 0 |
| 309 | lnc_HBl_018 | 2.53763 | 0 | 0 | 0 | 0.290841 |
| 310 | lnc_HBl_005 | 2.43634 | 0 | 0 | 0 | 0.559474 |
| 311 | lnc_H_007 | 2.61127 | 0 | 0 | 0 | 0 |
| 312 | lnc_H_005 | 2.53763 | 0 | 0 | 0 | 0 |
| 313 | lnc_H_006 | 2.53763 | 0 | 0 | 0 | 0 |
| 314 | lnc_HMBl_012 | 3.53769 | 0 | 1.82952 | 0 | 1.02104 |
| 315 | lnc_HMBl_001 | 3.8482 | 0 | 1.44898 | 0 | 2.22969 |
| 316 | U62 | 2.55882 | 0.980618 | 1.68846 | 1.65933 | 1.16875 |
| 317 | lnc_HLMBrBl_003 | 3.14777 | 0.825454 | 1.73508 | 1.63219 | 1.78237 |
| 318 | lnc_HLMBrBl_023 | 2.92155 | 1.18966 | 2.5574 | 0.901477 | 1.16201 |
| 319 | P806 | 3.56241 | 1.3253 | 2.27509 | 0.689554 | 1.8614 |
| 320 | lnc_HLMBr_017 | 2.46031 | 1.67709 | 2.34621 | 0.375027 | 0 |
| 321 | U6 | 1.23963 | 0.662601 | 1.90492 | 0.226717 | 0.649828 |
| 322 | P701 | 1.73712 | 0.387311 | 2.3451 | 0.388435 | 0.671832 |
| 323 | U30 | 1.27506 | 0.78198 | 2.39374 | 0.69292 | 0.836801 |
| 324 | lnc_HLMBrBl_008 | 2.8539 | 1.34826 | 1.18954 | 0.806518 | 0.775082 |
| 325 | lnc_HLMBrBl_036 | 2.85772 | 0.528547 | 0.821633 | 0.579835 | 0.67418 |
| 326 | U155 | 2.59543 | 0.297684 | 0.949333 | 0.64598 | 1.09467 |
| 327 | lnc_HMBl_017 | 2.57329 | 0 | 2.17531 | 0 | 0.294741 |
| 328 | lnc_HMBr_001 | 1.93075 | 0 | 1.46142 | 0.533972 | 0 |
| 329 | lnc_HMBr_023 | 2.53401 | 0 | 1.57096 | 0.334108 | 0 |
| 330 | lnc_HMBl_003 | 2.11011 | 0 | 0.972759 | 0 | 0.672318 |
| 331 | lnc_HMBl_014 | 1.91757 | 0 | 0.858396 | 0 | 1.22586 |
| 332 | P661.U387 | 2.11931 | 0 | 1.696 | 0.257585 | 0.675591 |
| 333 | U70 | 1.95984 | 0.380806 | 1.71871 | 0.537594 | 0.87357 |
| 334 | lnc_MBr_007 | 0 | 0 | 4.0143 | 1.66662 | 0 |
| 335 | U657 | 0.307192 | 0.357818 | 2.88199 | 0.652507 | 0.0962742 |
| 336 | P718 | 0.634401 | 0.64823 | 2.74998 | 0.764478 | 0.615778 |
| 337 | lnc_HLMBrBl_058 | 0.844435 | 0.652391 | 3.09833 | 0.63401 | 0.272872 |
| 338 | U622 | 0.0486467 | 0 | 3.04102 | 0.0694474 | 0 |
| 339 | lnc_HMBl_005 | 0.12689 | 0 | 3.21685 | 0 | 0.16331 |
| 340 | lnc_HMBr_037 | 0.801378 | 0 | 3.83855 | 0.122075 | 0 |
| 341 | lnc_HMBrBl_012 | 1.0914 | 0 | 3.64513 | 0.205687 | 0.37488 |
| 342 | lnc_HLMBl_003 | 0.519446 | 1.63169 | 1.32358 | 0 | 1.83738 |
| 343 | U262 | 0.689298 | 2.0107 | 1.64495 | 0.202087 | 1.25291 |
| 344 | lnc_HLMBl_001 | 0.095005 | 1.74927 | 2.74104 | 0 | 0.615313 |
| 345 | P529 | 0.415833 | 2.32625 | 2.18288 | 0.812224 | 0.68404 |
| 346 | lnc_HM_003 | 0.525051 | 0 | 2.28659 | 0 | 0 |
| 347 | lnc_HMBl_016 | 0.703529 | 0 | 1.90329 | 0 | 0.833692 |
| 348 | lnc_HLMBrBl_054 | 0.206992 | 0.416304 | 1.75102 | 0.507027 | 0.718778 |
| 349 | P643 | 0.551747 | 0.871345 | 2.13844 | 0.600031 | 0.354267 |
| 350 | U615 | 0.793231 | 0.0322282 | 1.41719 | 0.442041 | 0.00184061 |
| 351 | U485 | 0.833153 | 0 | 1.44259 | 0.702211 | 0.180804 |
| 352 | P747 | 0.0445864 | 0 | 1.5856 | 0.0388129 | 0 |
| 353 | U640 | 0.0889419 | 0.523249 | 1.41793 | 0.125035 | 0.233503 |
| 354 | lnc_HMBrBl_015 | 0.142425 | 0 | 1.3189 | 0.657419 | 0.185381 |
| 355 | lnc_MBrBl_004 | 0 | 0 | 1.61784 | 0.644599 | 0.364608 |
| 356 | lnc_HMBrBl_033 | 1.40977 | 0 | 1.75876 | 1.45387 | 0.757435 |
| 357 | P105.P99 | 0.44329 | 0.185349 | 1.5061 | 1.13881 | 0.68631 |
| 358 | U57 | 0.661244 | 0.17109 | 1.43874 | 1.65149 | 0.327088 |
| 359 | U141 | 0.928913 | 0.294547 | 1.68074 | 1.00695 | 0.0989579 |
| 360 | P447 | 0.995515 | 0.580856 | 1.84039 | 0.749387 | 0.305733 |
| 361 | U682 | 1.15874 | 0.567614 | 1.37676 | 1.27313 | 0.249272 |
| 362 | U379 | 1.28467 | 0.522414 | 1.30334 | 0.721345 | 0.39336 |
| 363 | lnc_HLMBrBl_056 | 1.3841 | 0.256675 | 1.34323 | 1.0888 | 0.509575 |
| 364 | P531 | 2.84689 | 1.50505 | 1.38579 | 2.33859 | 0.37064 |
| 365 | U176 | 1.79001 | 1.29373 | 0.948649 | 2.89446 | 1.56071 |
| 366 | P489 | 2.25828 | 1.90881 | 1.57442 | 2.6771 | 1.24243 |
| 367 | P646 | 1.19882 | 1.19007 | 2.02548 | 1.74589 | 1.99149 |
| 368 | lnc_HLMBrBl_022 | 1.20911 | 1.86231 | 1.71174 | 1.7945 | 0.912552 |
| 369 | U97 | 1.59204 | 1.36778 | 2.26092 | 1.31417 | 0.954424 |
| 370 | P1014 | 1.75444 | 1.05795 | 2.47365 | 1.97981 | 0.815285 |
| 371 | lnc_MBr_029 | 0 | 0 | 1.65687 | 2.41849 | 0 |
| 372 | lnc_HMBr_022 | 0.129357 | 0 | 2.30527 | 2.47557 | 0 |
| 373 | lnc_HMBr_008 | 1.10809 | 0 | 1.43299 | 2.26295 | 0 |
| 374 | lnc_HMBr_015 | 1.28523 | 0 | 1.03056 | 2.50035 | 0 |
| 375 | lnc_HLMBrBl_018 | 0.25573 | 0.802217 | 1.29879 | 1.89588 | 0.493598 |
| 376 | P248 | 0.625521 | 0.179234 | 1.69158 | 2.28636 | 0.2953 |
| 377 | lnc_HMBrBl_001 | 0.202609 | 0 | 1.76915 | 1.86297 | 0.513701 |
| 378 | U446 | 0.110202 | 0.0802329 | 1.69739 | 1.90093 | 0.108848 |
| 379 | P13 | 0.441007 | 0.0657598 | 1.55602 | 1.97537 | 0.193693 |
| 380 | U49 | 0.82581 | 0 | 0.793858 | 1.5994 | 0.389976 |
| 381 | P153 | 0.486671 | 0 | 0.80067 | 2.10363 | 0.401083 |
| 382 | lnc_HMBrBl_018 | 0.370498 | 0 | 1.10921 | 1.70754 | 0.120037 |
| 383 | lnc_HMBrBl_013 | 0.147736 | 0 | 1.16502 | 1.64971 | 0.37869 |
| 384 | lnc_HLMBrBl_050 | 0.411327 | 0.352196 | 0.934091 | 1.58267 | 0.0766769 |
| 385 | lnc_HMBr_014 | 0.098376 | 0 | 0.951291 | 1.54842 | 0 |
| 386 | lnc_HMBr_032 | 0.272173 | 0 | 0.859618 | 1.50066 | 0 |
| 387 | lnc_HBr_012 | 0.664455 | 0 | 0 | 3.09666 | 0 |
| 388 | lnc_HMBr_040 | 0.734418 | 0 | 0.515279 | 2.50035 | 0 |
| 389 | U275 | 0.875717 | 0.233619 | 0.397405 | 2.52732 | 0.213011 |
| 390 | lnc_HBr_018 | 0.186695 | 0 | 0 | 2.56644 | 0 |
| 391 | lnc_Br_035 | 0 | 0 | 0 | 2.50657 | 0 |
| 392 | lnc_Br_033 | 0 | 0 | 0 | 2.52551 | 0 |
| 393 | lnc_MBr_037 | 0 | 0 | 0.204198 | 2.79881 | 0 |
| 394 | lnc_Br_036 | 0 | 0 | 0 | 2.69223 | 0 |
| 395 | lnc_Br_037 | 0 | 0 | 0 | 2.72446 | 0 |
| 396 | lnc_BrBl_006 | 0 | 0 | 0 | 2.44876 | 0.277968 |
| 397 | lnc_HBrBl_003 | 0.327039 | 0 | 0 | 2.42144 | 0.281616 |
| 398 | lnc_Br_032 | 0 | 0 | 0 | 2.32408 | 0 |
| 399 | lnc_Br_030 | 0 | 0 | 0 | 2.12911 | 0 |
| 400 | lnc_Br_029 | 0 | 0 | 0 | 2.16424 | 0 |
| 401 | lnc_Br_031 | 0 | 0 | 0 | 2.19897 | 0 |
| 402 | lnc_HMBr_027 | 0.101683 | 0 | 0.494298 | 2.11752 | 0 |
| 403 | U230 | 0.104748 | 0.13687 | 0.537559 | 2.31337 | 0.178378 |
| 404 | lnc_HL_001 | 0.0401821 | 0.0593738 | 0.320462 | 2.58722 | 0.00493607 |
| 405 | U11 | 0.105695 | 0 | 0.364594 | 2.45689 | 0.0696053 |
| 406 | U7 | 0.122798 | 0 | 0.324455 | 2.56367 | 0.0809181 |
| 407 | U26 | 0 | 0 | 0.372289 | 2.35864 | 0 |
| 408 | P721 | 0 | 0 | 0.432243 | 2.33343 | 0 |
| 409 | lnc_MBr_040 | 0 | 0 | 0.264096 | 2.27353 | 0 |
| 410 | lnc_MBr_023 | 0 | 0 | 0.248924 | 2.33701 | 0 |
| 411 | U174 | 0 | 0 | 0.253571 | 2.41314 | 0 |
| 412 | lnc_MBr_006 | 0 | 0 | 0.340649 | 2.42633 | 0 |
| 413 | lnc_LMBr_001 | 0 | 0.891562 | 1.09742 | 3.39645 | 0 |
| 414 | lnc_HLMBr_006 | 0.156127 | 0.947599 | 0.913373 | 2.70853 | 0 |
| 415 | lnc_LMBr_005 | 0 | 1.10104 | 0.933488 | 3.04371 | 0 |
| 416 | lnc_HMBr_006 | 0.289055 | 0 | 0.755528 | 2.87972 | 0 |
| 417 | lnc_MBrBl_002 | 0 | 0 | 0.891197 | 2.71344 | 0.0734716 |
| 418 | U326 | 0.0999705 | 0 | 0.917389 | 2.77688 | 0.19703 |
| 419 | P6.P7 | 0.160852 | 0.386847 | 1.04398 | 2.67652 | 0.0872653 |
| 420 | lnc_MBr_021 | 0 | 0 | 1.05237 | 2.47991 | 0 |
| 421 | lnc_MBr_038 | 0 | 0 | 1.15301 | 2.61748 | 0 |
| 422 | P68 | 0.394788 | 0.383086 | 0.391057 | 3.21962 | 0.103996 |
| 423 | U182 | 0 | 0.375051 | 0.277689 | 3.18265 | 0 |
| 424 | lnc_LMBrBl_001 | 0 | 0.460181 | 0.177075 | 3.1174 | 0.19746 |
| 425 | lnc_Br_040 | 0 | 0 | 0 | 3.11529 | 0 |
| 426 | lnc_MBr_001 | 0 | 0 | 0.544244 | 2.90682 | 0 |
| 427 | P730.P731 | 0.0085736 | 0.0661489 | 0.322351 | 2.98825 | 0.0131665 |
| 428 | lnc_MBr_035 | 0 | 0 | 0.413832 | 3.18809 | 0 |
| 429 | U218 | 0.0397959 | 0.0657736 | 0.350803 | 3.17502 | 0.0187597 |
| 430 | P1037 | 0.250631 | 0 | 0.686545 | 3.25908 | 0.509164 |
| 431 | U186 | 0.154212 | 0.257964 | 0.473634 | 3.54875 | 0.231709 |
| 432 | P396 | 0.128413 | 0.0532697 | 0.348459 | 3.52493 | 0.0241819 |
| 433 | P931 | 0.214002 | 0 | 0.512508 | 3.56662 | 0 |
| 434 | U249 | 0 | 0 | 0.424115 | 3.38291 | 0 |
| 435 | lnc_HMBr_034 | 0.0600334 | 0 | 0.546389 | 3.40516 | 0 |
| 436 | lnc_MBr_005 | 0 | 0 | 0.564764 | 3.49078 | 0 |
| 437 | lnc_MBr_039 | 0 | 0 | 0.565685 | 3.54769 | 0 |
| 438 | P656 | 1.88494 | 0.661674 | 0.931465 | 1.37324 | 1.31568 |
| 439 | P169 | 1.51036 | 0 | 0.78721 | 0.952924 | 1.0096 |
| 440 | lnc_HMBrBl_024 | 1.96542 | 0 | 0.993658 | 0.871794 | 1.13794 |
| 441 | lnc_HBrBl_021 | 1.91404 | 0 | 0 | 1.04666 | 0.887374 |
| 442 | lnc_HBrBl_002 | 1.96542 | 0 | 0 | 0.653846 | 1.36553 |
| 443 | P541 | 1.31095 | 0 | 0 | 1.39432 | 1.25786 |
| 444 | U331 | 1.3474 | 0.186491 | 0.384515 | 1.33641 | 1.02871 |
| 445 | lnc_HMBrBl_021 | 1.66299 | 0 | 0.24175 | 0.809601 | 0.280294 |
| 446 | lnc_HMBr_018 | 1.69746 | 0 | 0.735495 | 0.93174 | 0 |
| 447 | P532 | 1.52613 | 0 | 0.927249 | 0.402741 | 0.216382 |
| 448 | lnc_HMBr_033 | 1.70438 | 0 | 0.933488 | 0.608742 | 0 |
| 449 | U298 | 1.35191 | 0.333524 | 0.76669 | 0.386671 | 0.517717 |
| 450 | U443 | 1.61243 | 0.469743 | 0.552055 | 0.210875 | 0.149283 |
| 451 | lnc_HLMBrBl_010 | 1.79196 | 0.61285 | 0.723395 | 0.29464 | 0.257671 |
| 452 | lnc_HBl_016 | 1.91404 | 0 | 0 | 0 | 0.221844 |
| 453 | lnc_HBrBl_007 | 2.0576 | 0 | 0 | 0.363975 | 0.404856 |
| 454 | lnc_HMBr_039 | 1.91757 | 0 | 0.429198 | 0.184525 | 0 |
| 455 | lnc_H_002 | 1.5109 | 0 | 0 | 0 | 0 |
| 456 | lnc_H_003 | 1.82135 | 0 | 0 | 0 | 0 |
| 457 | lnc_HBr_014 | 1.74461 | 0 | 0 | 0.204002 | 0 |
| 458 | lnc_HBl_004 | 1.20705 | 0 | 0 | 0 | 0.425148 |
| 459 | lnc_HBrBl_008 | 1.37937 | 0 | 0 | 0.300016 | 0.531401 |
| 460 | lnc_HMBrBl_017 | 1.29369 | 0 | 0.177529 | 0.426242 | 0.791619 |
| 461 | P754, lnc_HMBrBl_034 | 1.23052 | 0.062269 | 0.56755 | 0.233928 | 0.0686552 |
| 462 | lnc_HMBrBl_016 | 1.21768 | 0 | 0.224717 | 0.364435 | 0.242727 |
| 463 | U90 | 0.919065 | 0.131273 | 0.192881 | 0.31667 | 0.0884062 |
| 464 | P435 | 0.979719 | 0.215014 | 0.218438 | 0.486289 | 0.207992 |
| 465 | U146 | 0.92643 | 0 | 0.775636 | 0.860077 | 1.62666 |
| 466 | U341 | 1.04462 | 0.604787 | 0.665507 | 0.756486 | 1.27367 |
| 467 | U283 | 0.875082 | 0.445385 | 0.533496 | 1.08359 | 1.51827 |
| 468 | lnc_HLBl_004 | 1.23815 | 0.540442 | 0 | 0 | 1.60594 |
| 469 | U45 | 1.28041 | 0.409576 | 0.397704 | 0.24458 | 1.50936 |
| 470 | lnc_HLMBrBl_002 | 0.757206 | 0.348997 | 0.660846 | 0.104469 | 1.1402 |
| 471 | U644 | 0.827027 | 0.118848 | 0.174325 | 0.0168146 | 1.01482 |
| 472 | U346 | 0.922559 | 0.211026 | 0.305164 | 0.141523 | 0.963196 |
| 473 | P1132 | 0.201519 | 0 | 0.978135 | 0.199388 | 0.912552 |
| 474 | lnc_HLMBrBl_027 | 0.63445 | 0 | 1.28674 | 0.268095 | 1.14317 |
| 475 | U620 | 0.218112 | 0.142858 | 0.153093 | 0.719841 | 0.416809 |
| 476 | P907 | 0.266119 | 0.312516 | 0.230385 | 0.64687 | 0.734042 |
| 477 | U276 | 0.357321 | 0.172401 | 0.303127 | 1.11105 | 0.477108 |
| 478 | lnc_HLMBrBl_017 | 0.268168 | 0.546592 | 0.212981 | 0.946752 | 0.695523 |
| 479 | P127 | 0.303353 | 0.276837 | 0.551406 | 0.462827 | 1.05731 |
| 480 | lnc_HLMBrBl_026 | 0.371915 | 0.317296 | 0.239247 | 0.753828 | 1.11055 |
| 481 | U444 | 0.697309 | 0.638991 | 0.307979 | 0.754684 | 0.832951 |
| 482 | P1063 | 0.669929 | 0.194234 | 0.422688 | 0.652609 | 0.565469 |
| 483 | P328.P329 | 0.810061 | 0.233732 | 0.556264 | 0.866522 | 0.837298 |
| 484 | lnc_HMBl_010 | 0.441842 | 0 | 0.272397 | 0 | 1.42636 |
| 485 | U27 | 0.211032 | 0.448482 | 0.410761 | 0.031675 | 1.43852 |
| 486 | U558 | 0.16388 | 0 | 0 | 0.0308078 | 1.22881 |
| 487 | U48 | 0.133609 | 0.163139 | 0.0739822 | 0.0226604 | 1.14066 |
| 488 | U198 | 0.031218 | 0.241923 | 0.154008 | 0.0253192 | 1.57707 |
| 489 | lnc_BL_005 | 0 | 0 | 0 | 0 | 1.37289 |
| 490 | P1088 | 0 | 0 | 0 | 0 | 1.57852 |
| 491 | U228 | 0.0187644 | 0 | 0.0196327 | 0.135005 | 1.51072 |
| 492 | U539 | 0.0371266 | 0.0390614 | 0.120108 | 0.040492 | 0.748351 |
| 493 | lnc_MBrBl_010 | 0 | 0 | 0.143676 | 0.113923 | 0.819695 |
| 494 | P395 | 0.0905457 | 0.211526 | 0 | 0.014917 | 0.857794 |
| 495 | U33.U34 | 0.205308 | 0 | 0.0403659 | 0.0073152 | 0.933078 |
| 496 | P360 | 0.0248596 | 0 | 0.0669227 | 0.00852419 | 0.970171 |
| 497 | U241 | 0.0726029 | 0 | 0.0703633 | 0.0314043 | 1.00933 |
| 498 | U586 | 0.236596 | 0.237132 | 0.305283 | 0.02918 | 0.786445 |
| 499 | U86 | 0.294559 | 0.0977073 | 0.124203 | 0.176766 | 0.865458 |
| 500 | U637 | 0.430498 | 0.17304 | 0.221101 | 0.0364426 | 0.899093 |
| 501 | U300 | 0.241593 | 0 | 0.132153 | 0.0795965 | 0.69594 |
| 502 | P951 | 0.23201 | 0 | 0.188962 | 0.0244292 | 0.723408 |
| 503 | lnc_HLMBl_010 | 0.263883 | 0.169547 | 0.230973 | 0.00899958 | 0.586528 |
| 504 | U570 | 0.222067 | 0.13003 | 0.09551 | 0.0368914 | 0.70067 |
| 505 | U572 | 0.303873 | 0.205362 | 0.138149 | 0.0528344 | 0.668246 |
| 506 | U585 | 1.23544 | 0.129437 | 0.400458 | 1.97074 | 0.674623 |
| 507 | U454 | 1.16994 | 0.745672 | 0.759973 | 2.29527 | 1.08575 |
| 508 | U536 | 1.00735 | 1.20703 | 0.394264 | 2.24715 | 0.451416 |
| 509 | U206 | 0.800048 | 0.833632 | 0.885568 | 2.22462 | 0.644997 |
| 510 | P619 | 0.873559 | 1.24921 | 0.9524 | 1.81992 | 0.563008 |
| 511 | lnc_HLMBrBl_065 | 0.95005 | 0.583089 | 0.913678 | 1.57599 | 0.246125 |
| 512 | lnc_HLMBrBl_039 | 1.29577 | 0.741074 | 0.422275 | 1.72856 | 0.402342 |
| 513 | U334 | 0.642211 | 0.400368 | 0.493802 | 1.05647 | 0.300451 |
| 514 | U336 | 0.695281 | 0.27257 | 0.636358 | 1.14143 | 0.324144 |
| 515 | U307 | 0.768092 | 0.34757 | 0.642127 | 1.32037 | 0.349018 |
| 516 | U684 | 0.625559 | 0.561338 | 0.205126 | 1.24994 | 0.042228 |
| 517 | lnc_HLMBr_012 | 0.663424 | 0.504931 | 0.586673 | 1.25834 | 0 |
| 518 | U436 | 0.873882 | 0.556455 | 0.485958 | 0.946826 | 0 |
| 519 | P66 | 0.847353 | 0.490039 | 0.460553 | 1.1229 | 0.108657 |
| 520 | U490 | 0.989193 | 0.86283 | 0.396454 | 1.14481 | 0.733387 |
| 521 | U681 | 0.643202 | 0.994056 | 0.555399 | 0.890061 | 0.6359 |
| 522 | U555 | 0.64036 | 1.3875 | 0.440087 | 0.914151 | 0.615743 |
| 523 | U382 | 0.522934 | 1.19594 | 0.31551 | 1.46846 | 0.305121 |
| 524 | lnc_HLMBrBl_021 | 0.344414 | 1.46546 | 0.550605 | 1.34103 | 0.385971 |
| 525 | lnc_HMBrBl_005 | 0.426606 | 0.816973 | 0.54163 | 0.735898 | 0.353375 |
| 526 | U100 | 0.536695 | 0.926042 | 0.564127 | 0.760821 | 0.160114 |
| 527 | P469.P470.P472 | 0.381741 | 1.2541 | 0.548358 | 0.962014 | 0.125875 |
| 528 | lnc_HLMBrBl_063 | 0.598394 | 1.04275 | 0.809638 | 1.14179 | 0.110945 |
| 529 | lnc_HBr_022 | 0.892574 | 0 | 0 | 1.58714 | 0 |
| 530 | lnc_HBr_011 | 0.643535 | 0 | 0 | 2.26426 | 0 |
| 531 | lnc_HBr_020 | 0.470303 | 0 | 0 | 1.8439 | 0 |
| 532 | lnc_HBr_006 | 0.418237 | 0 | 0 | 1.98392 | 0 |
| 533 | lnc_HLMBr_015 | 0.098086 | 0.603229 | 0.711029 | 1.54277 | 0 |
| 534 | P392 | 0 | 0.817602 | 0.631847 | 1.58422 | 0 |
| 535 | lnc_HLMBrBl_042 | 0.206264 | 0.615283 | 0.463302 | 1.50384 | 0.539095 |
| 536 | lnc_HLMBrBl_034 | 0.692779 | 0.528547 | 0.410816 | 1.57384 | 0.33709 |
| 537 | P1054.P1055 | 0.0038834 | 0.0255189 | 0.187881 | 2.13313 | 0.00472684 |
| 538 | U133 | 0.0912974 | 0.0352799 | 0.281373 | 2.07513 | 0.0160566 |
| 539 | U8 | 0 | 0 | 0.300987 | 1.84304 | 0.0364627 |
| 540 | lnc_MBr_034 | 0 | 0 | 0.352781 | 1.92824 | 0 |
| 541 | P1083 | 0.0303714 | 0.0704176 | 0.216984 | 1.93415 | 0.0160243 |
| 542 | P1091 | 0 | 0 | 0.14173 | 1.94797 | 0 |
| 543 | P18 | 0.007926 | 0 | 0.224167 | 2.01821 | 0.00522957 |
| 544 | P445 | 0.0614754 | 0.369113 | 0 | 1.9434 | 0 |
| 545 | P832 | 0.132741 | 0 | 0 | 1.75771 | 0 |
| 546 | lnc_Br_018 | 0 | 0 | 0 | 1.63493 | 0 |
| 547 | lnc_Br_019 | 0 | 0 | 0 | 1.63949 | 0 |
| 548 | lnc_Br_022 | 0 | 0 | 0 | 1.68837 | 0 |
| 549 | lnc_Br_020 | 0 | 0 | 0 | 1.67054 | 0 |
| 550 | lnc_Br_021 | 0 | 0 | 0 | 1.67238 | 0 |
| 551 | lnc_Br_028 | 0 | 0 | 0 | 1.96877 | 0 |
| 552 | lnc_Br_026 | 0 | 0 | 0 | 1.8951 | 0 |
| 553 | lnc_Br_027 | 0 | 0 | 0 | 1.9258 | 0 |
| 554 | lnc_Br_024 | 0 | 0 | 0 | 1.81468 | 0 |
| 555 | lnc_Br_025 | 0 | 0 | 0 | 1.81468 | 0 |
| 556 | U583 | 0 | 0 | 0.109197 | 1.79798 | 0 |
| 557 | lnc_MBr_030 | 0 | 0 | 0.133185 | 1.84278 | 0 |
| 558 | lnc_LBr_004 | 0 | 0.639364 | 0 | 1.34153 | 0 |
| 559 | U127 | 0.045008 | 0.115429 | 0.135878 | 1.5669 | 0.118309 |
| 560 | lnc_Br_016 | 0 | 0 | 0 | 1.44473 | 0 |
| 561 | lnc_Br_017 | 0 | 0 | 0 | 1.51637 | 0 |
| 562 | P174 | 0 | 0 | 0.0742259 | 1.4912 | 0.01539 |
| 563 | U324 | 0.035562 | 0 | 0.131295 | 1.49527 | 0 |
| 564 | lnc_BrBl_005 | 0 | 0 | 0 | 1.29456 | 0.318338 |
| 565 | lnc_BrBl_009 | 0 | 0 | 0 | 1.41708 | 0.26386 |
| 566 | U551 | 0.0079725 | 0.184605 | 0.10857 | 1.33109 | 0.0526025 |
| 567 | lnc_Br_015 | 0 | 0 | 0 | 1.36395 | 0 |
| 568 | U12 | 0 | 0 | 0.136546 | 1.32724 | 0 |
| 569 | lnc_Br_013 | 0 | 0 | 0 | 1.20941 | 0 |
| 570 | lnc_Br_014 | 0 | 0 | 0 | 1.28475 | 0 |
| 571 | P355 | 0.0022856 | 0 | 0.133758 | 1.18569 | 0.00301717 |
| 572 | lnc_MBr_017 | 0 | 0 | 0.137957 | 1.20138 | 0 |
| 573 | P154 | 0 | 0.330392 | 0.49917 | 1.32893 | 0.0721446 |
| 574 | U377 | 0.100697 | 0.19796 | 0.372197 | 1.30601 | 0.256798 |
| 575 | lnc_MBr_041 | 0 | 0 | 0.586253 | 1.33271 | 0 |
| 576 | U14 | 0 | 0.0966262 | 0.368459 | 1.12268 | 0.0109737 |
| 577 | lnc_MBrBl_007 | 0 | 0 | 0.450897 | 1.18851 | 0.121699 |
| 578 | lnc_MBrBl_009 | 0 | 0 | 0.416994 | 1.17812 | 0.227637 |
| 579 | lnc_MBrBl_003 | 0 | 0 | 0.496067 | 1.72075 | 0.13193 |
| 580 | lnc_MBr_011 | 0 | 0 | 0.318728 | 1.72514 | 0 |
| 581 | lnc_HMBr_024 | 0.135136 | 0 | 0.347477 | 1.75047 | 0 |
| 582 | U641 | 0 | 0 | 0.283131 | 1.4028 | 0 |
| 583 | U285 | 0.0968271 | 0.00924725 | 0.197088 | 1.42553 | 0.0482159 |
| 584 | lnc_MBr_025 | 0 | 0 | 0.361989 | 1.52603 | 0 |
| 585 | lnc_MBr_022 | 0 | 0 | 0.413832 | 1.59404 | 0 |
| 586 | lnc_HMBr_029 | 0.0775408 | 0 | 0.279391 | 1.62229 | 0 |
| 587 | U22 | 0 | 0 | 0.198994 | 1.64868 | 0 |
| 588 | lnc_MBr_014 | 0 | 0 | 0.250724 | 1.63833 | 0 |
| 589 | U666 | 0.671262 | 1.86242 | 0.873751 | 0.690178 | 0.115311 |
| 590 | U635 | 1.05479 | 1.34884 | 0.691472 | 0.992636 | 0.264104 |
| 591 | P484 | 1.22973 | 1.6289 | 1.00412 | 0.64976 | 0.564852 |
| 592 | P152 | 0.503851 | 1.12727 | 0.954383 | 0.255227 | 1.24346 |
| 593 | P658 | 0.805128 | 1.37293 | 0.726639 | 0.392055 | 0.84878 |
| 594 | U54 | 0.997219 | 0.994958 | 0.885448 | 0.26406 | 0.422053 |
| 595 | P905 | 0.865484 | 0.793733 | 1.29392 | 0.904369 | 0.821848 |
| 596 | P141 | 0.0528743 | 1.18397 | 0.0339979 | 0.0321834 | 0.0267369 |
| 597 | lnc_HBl_003 | 0.0236666 | 1.28357 | 0.0742675 | 0.00945659 | 0.511508 |
| 598 | U99 | 0.0057598 | 1.60222 | 0.133075 | 0.00462851 | 0.106371 |
| 599 | U483 | 0.0263374 | 1.70085 | 0.113962 | 0.0825731 | 0.0345959 |
| 600 | P793 | 0.0469651 | 1.18676 | 0.767021 | 0.0256631 | 0.123654 |
| 601 | U479 | 0.264123 | 1.1084 | 0.449824 | 0.332925 | 0.443506 |
| 602 | U247 | 0.43898 | 1.0855 | 0.614756 | 0.489246 | 0.301109 |
| 603 | lnc_HLMBrBl_043 | 0.506594 | 1.86363 | 0.401482 | 0.0928554 | 0.246843 |
| 604 | U567 | 0.295035 | 1.73015 | 0.689719 | 0.307833 | 0.323072 |
| 605 | U425.U427 | 0.333278 | 1.92202 | 0.754462 | 0.107049 | 0.276565 |
| 606 | lnc_HLMBr_011 | 0.115228 | 1.36336 | 0.422936 | 0.560976 | 0 |
| 607 | U421 | 0.091755 | 1.63624 | 0.306502 | 0.560668 | 0.0600226 |
| 608 | U89 | 0.554967 | 1.45581 | 0.245423 | 0.173719 | 0.131 |
| 609 | P437 | 0.257313 | 1.54783 | 0.294682 | 0.292365 | 0.335386 |
| 610 | U254 | 0.471826 | 1.70082 | 0.451124 | 0.475558 | 0.47722 |
| 611 | lnc_HMBr_007 | 0.507174 | 0 | 0.672239 | 0.67076 | 0 |
| 612 | P1005 | 0.648708 | 0.026159 | 0.503946 | 0.436392 | 0.189221 |
| 613 | P441.P442 | 0.610426 | 0.148096 | 0.62059 | 0.379003 | 0.100439 |
| 614 | lnc_HLMBrBl_066 | 0.685554 | 0.175372 | 0.453692 | 0.704191 | 0.0391251 |
| 615 | lnc_HLMBrBl_046 | 0.669158 | 0.403199 | 0.307766 | 0.856503 | 0.0871735 |
| 616 | lnc_HBr_010 | 0.680916 | 0.0150026 | 0.360438 | 0.955629 | 0.0382224 |
| 617 | P404 | 0.691133 | 0.0685844 | 0.248213 | 0.948684 | 0.213675 |
| 618 | lnc_HMBrBl_006 | 0.532048 | 0 | 0.422072 | 0.937598 | 0.345031 |
| 619 | lnc_HMBrBl_011 | 0.497767 | 0 | 0.482263 | 0.982135 | 0.128836 |
| 620 | U256 | 0.654015 | 0 | 0.526613 | 0.969978 | 0.179956 |
| 621 | lnc_HLMBr_010 | 0.159114 | 0.483335 | 0.933042 | 1.19778 | 0 |
| 622 | P438.P440 | 0.210387 | 0.414039 | 1.16626 | 1.00971 | 0.0676509 |
| 623 | lnc_HMBr_020 | 0.0831352 | 0 | 0.980554 | 0.946566 | 0 |
| 624 | U687 | 0.295724 | 0.0951936 | 0.770343 | 0.831267 | 0.0072244 |
| 625 | P699 | 0.340938 | 0 | 0.94275 | 0.782257 | 0.0079427 |
| 626 | P106 | 0.329933 | 0 | 0.492983 | 0.993751 | 0.239166 |
| 627 | lnc_HMBr_025 | 0.443314 | 0 | 0.546943 | 1.12404 | 0 |
| 628 | U194 | 0.32409 | 0.271258 | 0.797602 | 1.13668 | 0.212995 |
| 629 | U297 | 0.44309 | 0.139342 | 0.83741 | 1.22764 | 0.220875 |
| 630 | U81 | 0 | 0.506551 | 0.68296 | 0.432894 | 0 |
| 631 | lnc_HLMBrBl_019 | 0.149761 | 0.67704 | 1.0552 | 0.351049 | 0.0576225 |
| 632 | P370 | 0.470471 | 0.309439 | 0.885374 | 0.752652 | 0.232528 |
| 633 | U626 | 0.222516 | 0.496591 | 0.70514 | 0.579449 | 0.0881044 |
| 634 | U24 | 0.398367 | 0.587224 | 0.725466 | 0.592396 | 0.174181 |
| 635 | P494 | 0 | 0.538458 | 0.584619 | 0.608134 | 0.564727 |
| 636 | U415 | 0.611392 | 0.748362 | 0.83007 | 0.304489 | 0.648024 |
| 637 | U111 | 0.363154 | 0.642775 | 0.571484 | 0.582823 | 0.825142 |
| 638 | U633 | 0.483578 | 0.597339 | 0.494925 | 0.425644 | 0.501572 |
| 639 | lnc_HLMBrBl_067 | 0.340674 | 0.722418 | 0.528278 | 0.414545 | 0.576216 |
| 640 | P481.P482 | 0.0368488 | 0.211876 | 0.754297 | 0 | 0.238664 |
| 641 | P336 | 0.0487343 | 0.121582 | 0.482617 | 0.00560244 | 0.0369317 |
| 642 | U323 | 0.0376763 | 0.164133 | 0.596594 | 0.0228727 | 0 |
| 643 | U147 | 0.144737 | 0 | 0.676261 | 0 | 0 |
| 644 | U568 | 0.0551304 | 0.0133605 | 0.654149 | 0.0649867 | 0.0702585 |
| 645 | P937 | 0.226395 | 0 | 0.602816 | 0.278639 | 0.155329 |
| 646 | P143.P145 | 0.323667 | 0 | 0.486521 | 0.143262 | 0.0150747 |
| 647 | U71 | 0.186995 | 0 | 0.520119 | 0.0308434 | 0.17221 |
| 648 | U498 | 0.306822 | 0 | 0.6287 | 0.08504 | 0.150237 |
| 649 | P615 | 0.217578 | 0 | 0.312825 | 0.0604864 | 0.0317699 |
| 650 | P802 | 0.185682 | 0.037921 | 0.256534 | 0.158747 | 0.0539523 |
| 651 | U578 | 0.200558 | 0 | 0.292411 | 0.173566 | 0.16512 |
| 652 | U691 | 0.146835 | 0.0457011 | 0.411468 | 0.203827 | 0.0339103 |
| 653 | U543 | 0.085183 | 0.204259 | 0.361163 | 0.152594 | 0 |
| 654 | U74 | 0.0272571 | 0.158742 | 0.405266 | 0.244751 | 0.053846 |
| 655 | U677 | 0.0520402 | 0.0431287 | 0.359991 | 0.0959033 | 0.00980337 |
| 656 | U590 | 0.0049935 | 0.0144406 | 0.360204 | 0.00996776 | 0.0065916 |
| 657 | P1122 | 0.0304003 | 0 | 0.385496 | 0.00821418 | 0 |
| 658 | U541 | 0.10429 | 0 | 0.409579 | 0.00560029 | 0 |
| 659 | P980 | 0.111797 | 0.0446289 | 0.314932 | 0.0185173 | 0.035611 |
| 660 | P727 | 0.153951 | 0.0457012 | 0.330792 | 0.0284469 | 0.0156273 |
| 661 | lnc_MBr_003 | 0 | 0 | 0.651411 | 0.468943 | 0 |
| 662 | P983 | 0 | 0 | 0.757641 | 0.541833 | 0.343918 |
| 663 | U106 | 0.231096 | 0.260839 | 0.595368 | 0.542631 | 0.375706 |
| 664 | P71.P72 | 0.322728 | 0.0163749 | 0.633591 | 0.72151 | 0.325085 |
| 665 | P1095.P1096 | 0.302874 | 0.0704889 | 0.847337 | 0.69016 | 0.287284 |
| 666 | P34.P35 | 0.0078928 | 0 | 1.04267 | 0.0797265 | 0.0155665 |
| 667 | P224 | 0.165109 | 0.0409208 | 1.21779 | 0.203335 | 0.00311359 |
| 668 | U178 | 0.277617 | 0.13456 | 0.979885 | 0.0376139 | 0.0762027 |
| 669 | P556.P558 | 0.5126 | 0.0162238 | 1.05774 | 0.00195414 | 0.00478844 |
| 670 | U196 | 0.554967 | 0 | 0.987552 | 0.14783 | 0.545804 |
| 671 | U36 | 0.271911 | 0.068773 | 0.951746 | 0.355775 | 0.404963 |
| 672 | U636 | 0.281411 | 0.0865974 | 1.02092 | 0.0263187 | 0.367475 |
| 673 | U118 | 0.231729 | 0.0263119 | 1.14324 | 0.171196 | 0.233813 |
| 674 | lnc_HLBrBl_005 | 0.162453 | 1.20301 | 0 | 0.771219 | 0.0531923 |
| 675 | lnc_LBr_001 | 0 | 0.966671 | 0 | 1.12292 | 0 |
| 676 | lnc_HLBr_001 | 0.0961027 | 1.18051 | 0 | 1.03426 | 0 |
| 677 | lnc_HLBr_003 | 0.199943 | 0.401513 | 0 | 0.730764 | 0 |
| 678 | P381 | 0.254432 | 0.207412 | 0.230302 | 0.869322 | 0.118342 |
| 679 | U85 | 0.0675466 | 0.295642 | 0.144145 | 0.90191 | 0.0888987 |
| 680 | lnc_HLMBr_009 | 0.0944913 | 0.281085 | 0.316292 | 0.868638 | 0 |
| 681 | lnc_HLMBrBl_011 | 0.105587 | 0.315213 | 0.237607 | 0.514588 | 0.137947 |
| 682 | P282 | 0.135152 | 0.209931 | 0.299227 | 0.558343 | 0.19706 |
| 683 | P798 | 0.223056 | 0.479513 | 0.261201 | 0.667171 | 0.163381 |
| 684 | P29 | 0.354891 | 0.324234 | 0.307725 | 0.708804 | 0.218782 |
| 685 | lnc_HBrBl_014 | 0.305095 | 0 | 0 | 0.85478 | 0.198352 |
| 686 | lnc_HBrBl_009 | 0.438928 | 0 | 0 | 1.10989 | 0.141716 |
| 687 | U9 | 0.190628 | 0 | 0.0605293 | 1.1206 | 0 |
| 688 | lnc_HMBr_011 | 0.358521 | 0 | 0.213637 | 1.12254 | 0 |
| 689 | P1094 | 0.189593 | 0.0261401 | 0.283936 | 1.14708 | 0.0952991 |
| 690 | lnc_Br_011 | 0 | 0 | 0 | 1.10079 | 0 |
| 691 | lnc_HMBrBl_002 | 0 | 0 | 0 | 0.945709 | 0 |
| 692 | lnc_Br_010 | 0 | 0 | 0 | 1.00922 | 0 |
| 693 | lnc_MBr_009 | 0 | 0 | 0.241132 | 0.982135 | 0 |
| 694 | lnc_MBr_033 | 0 | 0 | 0.214298 | 1.03959 | 0 |
| 695 | P759 | 0.0635621 | 0.0120039 | 0.187684 | 1.10754 | 0.102034 |
| 696 | lnc_HMBr_013 | 0.0567562 | 0 | 0.128494 | 1.06535 | 0 |
| 697 | P121 | 0.14654 | 0 | 0.153635 | 1.07937 | 0.0060422 |
| 698 | P483 | 0.0052274 | 0.0604686 | 0.180354 | 0.772301 | 0.148349 |
| 699 | lnc_HBr_017 | 0.176173 | 0.017573 | 0.158881 | 0.864285 | 0.108244 |
| 700 | P86 | 0.0132303 | 0.0198878 | 0.163832 | 0.796518 | 0.0113411 |
| 701 | U556 | 0.025493 | 0 | 0.304599 | 0.796432 | 0 |
| 702 | lnc_HMBr_036 | 0.110496 | 0 | 0.249585 | 0.836735 | 0 |
| 703 | lnc_Br_005 | 0 | 0 | 0 | 0.706987 | 0 |
| 704 | lnc_Br_008 | 0 | 0 | 0 | 0.783548 | 0 |
| 705 | P69 | 0 | 0 | 0.0646209 | 0.680633 | 0 |
| 706 | U1 | 0.0082941 | 0.0479249 | 0.0519141 | 0.676833 | 0.00136895 |
| 707 | P663 | 0.0039724 | 0.0689867 | 0.0665795 | 0.731613 | 0.0157262 |
| 708 | P1089 | 0.0096578 | 0.0279124 | 0.0873725 | 0.765984 | 0.00255411 |
| 709 | U552 | 0 | 0.0325539 | 0.117971 | 0.74918 | 0 |
| 710 | lnc_HBr_009 | 0.0877393 | 0 | 0 | 0.841514 | 0 |
| 711 | P916.U560 | 0.0023484 | 0 | 0.0415253 | 0.890323 | 0.000674182 |
| 712 | U92 | 0 | 0 | 0.0654868 | 0.914964 | 0 |
| 713 | P432 | 0 | 0.0112952 | 0.0612216 | 0.930255 | 0.00257911 |
| 714 | P785 | 0.0104163 | 0 | 0.0990203 | 0.835008 | 0 |
| 715 | P742 | 0.0036064 | 0 | 0.113009 | 0.887218 | 0 |
| 716 | P4 | 0.0377482 | 0.0364721 | 0.0925683 | 0.885668 | 0 |
| 717 | lnc_HLBr_004 | 1.00172 | 0.402354 | 0 | 0.366225 | 0 |
| 718 | lnc_HLMBl_006 | 1.04954 | 0.789821 | 0.150541 | 0 | 0.170955 |
| 719 | U665 | 0.583303 | 0.0874025 | 0.0350834 | 0.00133892 | 0.104247 |
| 720 | P338 | 0.392869 | 0.187365 | 0.174459 | 0.0852334 | 0.0243935 |
| 721 | U119 | 0.831191 | 0.0139957 | 0.147374 | 0.101112 | 0.14618 |
| 722 | P358 | 0.822153 | 0 | 0.312483 | 0.048117 | 0.0668346 |
| 723 | U288 | 0.523117 | 0.543479 | 0.335031 | 0.286176 | 0.201918 |
| 724 | U451 | 0.457645 | 0.544072 | 0.376727 | 0.559629 | 0.301948 |
| 725 | U240 | 0.617802 | 0.766563 | 0.141973 | 0.413865 | 0.0853152 |
| 726 | U442 | 0.511449 | 0.843937 | 0.264835 | 0.497755 | 0.2458 |
| 727 | P210 | 0.165128 | 0.704078 | 0.279541 | 0.795588 | 0.188577 |
| 728 | P405 | 0.0317403 | 0.560934 | 0.138428 | 0.457119 | 0.0832935 |
| 729 | P286 | 0.127298 | 0.659207 | 0.180391 | 0.50855 | 0.0186258 |
| 730 | lnc_HLMBrBl_032 | 0.161467 | 0.717315 | 0.267471 | 0.487543 | 0.264361 |
| 731 | P450 | 0.23812 | 0.558282 | 0.102653 | 0.41673 | 0.250358 |
| 732 | U310 | 0.25767 | 0.720682 | 0.0604573 | 0.448538 | 0.113211 |
| 733 | lnc_HLBrBl_003 | 0.145475 | 0.568609 | 0.0522948 | 0.0404477 | 0 |
| 734 | U370 | 0.217256 | 0.565818 | 0.0227464 | 0.0436647 | 0.210256 |
| 735 | P870 | 0 | 0.839775 | 0 | 0 | 0.0690855 |
| 736 | P782 | 0 | 0.745514 | 0.109809 | 0.042496 | 0 |
| 737 | U407 | 0.0267475 | 0.650875 | 0.0449174 | 0 | 0.0423435 |
| 738 | lnc_L_002 | 0.003735 | 0.701765 | 0.0039009 | 0.0104245 | 0.00986174 |
| 739 | U674 | 0 | 0.731453 | 0 | 0 | 0 |
| 740 | P126 | 0.192272 | 0.72031 | 0.378385 | 0.175786 | 0.100119 |
| 741 | U505 | 0.343644 | 0.769548 | 0.336684 | 0.247945 | 0.174082 |
| 742 | U238 | 0.0661963 | 0.780579 | 0.144639 | 0.0843579 | 0.34733 |
| 743 | U96 | 0.264874 | 0.836603 | 0.260497 | 0.184726 | 0.423112 |
| 744 | P122 | 0.156022 | 0.918794 | 0.203976 | 0.132029 | 0.245689 |
| 745 | P492 | 0.266477 | 0.901663 | 0.238771 | 0.146138 | 0.173553 |
| 746 | U365 | 0.0696155 | 0.57308 | 0.312905 | 0.152449 | 0.193653 |
| 747 | U102 | 0.251293 | 0.532917 | 0.28336 | 0.0856521 | 0.129351 |
| 748 | P64 | 0.249011 | 0.450802 | 0.391941 | 0.104101 | 0.116349 |
| 749 | lnc_HLMBrBl_040 | 0.187178 | 0.485496 | 0.330948 | 0.180627 | 0.144816 |
| 750 | U393 | 0.259384 | 0.443147 | 0.561459 | 0.0238477 | 0.269323 |
| 751 | P896.P897 | 0.18929 | 0.399214 | 0.439754 | 0.236735 | 0.252357 |
| 752 | U188 | 0.156316 | 0.258496 | 0.342962 | 0.0625539 | 0.245578 |
| 753 | U408 | 0.186702 | 0.366466 | 0.338963 | 0.105308 | 0.163336 |
| 754 | lnc_HLBrBl_007 | 0.224004 | 0.329112 | 0 | 0.447209 | 0.147071 |
| 755 | U523 | 0.112988 | 0.376609 | 0.103225 | 0.317831 | 0.0424934 |
| 756 | U566 | 0.0908912 | 0.470664 | 0.110848 | 0.281069 | 0.0935138 |
| 757 | P241.P243 | 0.305171 | 0.489051 | 0.180429 | 0.209772 | 0.218586 |
| 758 | P1105 | 0.303162 | 0.542595 | 0.222267 | 0.237013 | 0.215069 |
| 759 | P308 | 0.294947 | 0.430889 | 0.130891 | 0.238097 | 0.377496 |
| 760 | U612 | 0.33735 | 0.482039 | 0.213762 | 0.233862 | 0.336482 |
| 761 | U386 | 0.175005 | 0.371153 | 0.237323 | 0.20875 | 0.1466 |
| 762 | P613 | 0.205359 | 0.340885 | 0.227226 | 0.245559 | 0.193335 |
| 763 | U329 | 0.30431 | 0.348985 | 0.191996 | 0.27655 | 0.235468 |
| 764 | U50 | 0.193359 | 0.354842 | 0.172076 | 0.338253 | 0.187772 |
| 765 | P94 | 0.204381 | 0.354765 | 0.245106 | 0.404948 | 0.207462 |
| 766 | U522 | 0.0510927 | 0.409509 | 0 | 0.0188811 | 0.264505 |
| 767 | P1076 | 0.296431 | 0.35716 | 0.0929581 | 0.120305 | 0.224994 |
| 768 | P878.P879 | 0.200253 | 0.291454 | 0.0352072 | 0.118582 | 0.101543 |
| 769 | P882 | 0.172706 | 0.219137 | 0.0642575 | 0.0625347 | 0.140392 |
| 770 | U573.U574 | 0.152532 | 0.244118 | 0.0556558 | 0.0861959 | 0.219279 |
| 771 | U554 | 0.165487 | 0.274036 | 0.161411 | 0.175779 | 0.343028 |
| 772 | U342 | 0.156829 | 0.37674 | 0.151311 | 0.140604 | 0.242053 |
| 773 | P52 | 0.198474 | 0.348687 | 0.170677 | 0.153123 | 0.252576 |
| 774 | P211 | 0.0827512 | 0.363059 | 0.177563 | 0.0171307 | 0.0272062 |
| 775 | P459 | 0.0236521 | 0.465737 | 0.0203696 | 0.0265549 | 0.0896839 |
| 776 | U432 | 0.102432 | 0.4448 | 0.044727 | 0.0649723 | 0.0506857 |
| 777 | U290 | 0.0075514 | 0.349253 | 0 | 0.0348483 | 0.034892 |
| 778 | P386 | 0 | 0.333107 | 0.0614789 | 0 | 0.0372037 |
| 779 | P801 | 0 | 0.341622 | 0.0406132 | 0.0154193 | 0.131266 |
| 780 | U158 | 0.0458625 | 0.34527 | 0.02885 | 0.077215 | 0.133129 |
| 781 | U526 | 0.16134 | 0.280907 | 0.0849958 | 0.28654 | 0.117 |
| 782 | U63 | 0.0813849 | 0.237991 | 0.0872724 | 0.21887 | 0.240829 |
| 783 | lnc_HLBrBl_006 | 0.0590969 | 0.284726 | 0.0411537 | 0.262388 | 0.223648 |
| 784 | P448 | 0.0660648 | 0.194749 | 0.0721689 | 0.112238 | 0.0866613 |
| 785 | P945 | 0.0300131 | 0.260369 | 0.0732062 | 0.149771 | 0.0528232 |
| 786 | U352 | 0.120239 | 0.234362 | 0.12888 | 0.165723 | 0.131788 |
| 787 | U388 | 0.107636 | 0.254519 | 0.118221 | 0.201547 | 0.130966 |
| 788 | U527 | 0.688897 | 0.211759 | 0.116269 | 0.343478 | 0.453216 |
| 789 | P31 | 0.8338 | 0.120892 | 0.116948 | 0.151112 | 0.311557 |
| 790 | U550 | 0.693982 | 0.298434 | 0.271005 | 0.12462 | 0.327563 |
| 791 | U21 | 0.639303 | 0.339847 | 0.254066 | 0.365863 | 0.43247 |
| 792 | U604 | 0.570056 | 0.457065 | 0.352264 | 0.365339 | 0.503065 |
| 793 | P624.P625 | 0.702857 | 0.205891 | 0.463138 | 0.451869 | 0.346416 |
| 794 | U580 | 0.775059 | 0.442391 | 0.419373 | 0.434186 | 0.3253 |
| 795 | U103 | 0.470007 | 0.492576 | 0.178282 | 0.285557 | 0.748533 |
| 796 | lnc_HLMBrBl_049 | 0.600928 | 0.496322 | 0.273087 | 0.169616 | 0.561504 |
| 797 | U72 | 0.384704 | 0.378555 | 0.201457 | 0.163227 | 0.614281 |
| 798 | U463 | 0.51983 | 0.264839 | 0.296304 | 0.219863 | 0.598144 |
| 799 | U603 | 0.5956 | 0.0733689 | 0.177057 | 0.0913717 | 0.652144 |
| 800 | lnc_HMBl_015 | 0.49188 | 0 | 0.237823 | 0 | 0.382027 |
| 801 | U295 | 0.459996 | 0.0887528 | 0.441614 | 0.055236 | 0.576679 |
| 802 | P67 | 0.367595 | 0.0314779 | 0.260817 | 0.394412 | 0.764105 |
| 803 | U327 | 0.531079 | 0.0102109 | 0.324413 | 0.263035 | 0.414222 |
| 804 | U627 | 0.505535 | 0.115899 | 0.381647 | 0.421188 | 0.502714 |
| 805 | lnc_H_001 | 0.381246 | 0 | 0.060026 | 0.461511 | 0.0557829 |
| 806 | P209 | 0.309541 | 0.0408021 | 0.281252 | 0.589423 | 0.23194 |
| 807 | P666 | 0.451307 | 0.158392 | 0.219846 | 0.46413 | 0.29473 |
| 808 | P864.P865.P867 | 0.37709 | 0.150429 | 0.135604 | 0.693716 | 0.099318 |
| 809 | P311 | 0.410028 | 0.270626 | 0.157376 | 0.588244 | 0.159687 |
| 810 | P971 | 0.167193 | 0.141914 | 0.361516 | 0.437657 | 0.016773 |
| 811 | lnc_HMBr_031 | 0.254231 | 0 | 0.375463 | 0.513949 | 0 |
| 812 | P120 | 0.310865 | 0 | 0.428161 | 0.534652 | 0.0509119 |
| 813 | lnc_HMBrBl_007 | 0.362745 | 0 | 0.274974 | 0.194618 | 0.11223 |
| 814 | U667 | 0.459865 | 0 | 0.282132 | 0.24621 | 0.0159428 |
| 815 | lnc_LBr_002 | 0.33687 | 0.12501 | 0.180838 | 0.284342 | 0.10184 |
| 816 | lnc_HMBr_012 | 0.374514 | 0.0722276 | 0.313472 | 0.321843 | 0.115336 |
| 817 | U529 | 0.179943 | 0.0401372 | 0.101921 | 0.395776 | 0.255564 |
| 818 | U669 | 0.0915457 | 0.0441571 | 0.0479373 | 0.369464 | 0.0855724 |
| 819 | U459 | 0.0838958 | 0.0179775 | 0.110532 | 0.424745 | 0.139437 |
| 820 | P32 | 0.180532 | 0.0869895 | 0.130241 | 0.363537 | 0.107812 |
| 821 | P928.P929 | 0.112367 | 0.0650644 | 0.113888 | 0.353066 | 0.0889734 |
| 822 | U130 | 0.0897639 | 0.104839 | 0.153481 | 0.325237 | 0.0708679 |
| 823 | U642 | 0.220544 | 0.155011 | 0.21083 | 0.382001 | 0.0440709 |
| 824 | P1041.P1042 | 0.309159 | 0.175006 | 0.132534 | 0.440558 | 0.119318 |
| 825 | U59 | 0.243797 | 0.159105 | 0.117338 | 0.272734 | 0.213462 |
| 826 | U528 | 0.224457 | 0.190324 | 0.218383 | 0.228632 | 0.209439 |
| 827 | U134 | 0.239733 | 0.269201 | 0.194795 | 0.283431 | 0.22666 |
| 828 | P930 | 0 | 0 | 0.11859 | 0.335171 | 0 |
| 829 | U189 | 0.007701 | 0 | 0.113536 | 0.366526 | 0.0101545 |
| 830 | P254 | 0 | 0.0263516 | 0.0572484 | 0.331969 | 0.084059 |
| 831 | U52 | 0.0051556 | 0.0298666 | 0.108193 | 0.372419 | 0.108827 |
| 832 | lnc_Br_002 | 0 | 0 | 0 | 0.440528 | 0 |
| 833 | P581 | 0 | 0.025803 | 0.0186841 | 0.460737 | 0.00587968 |
| 834 | P880 | 0 | 0 | 0.0311839 | 0.400053 | 0 |
| 835 | lnc_Br_001 | 0 | 0 | 0 | 0.409433 | 0 |
| 836 | P237 | 0 | 0 | 0.00907496 | 0.392847 | 0.0171386 |
| 837 | U211 | 0.0102103 | 0.0256559 | 0.0609011 | 0.369587 | 0.0019258 |
| 838 | P412 | 0.0471083 | 0 | 0.054928 | 0.390766 | 0.0483375 |
| 839 | U255 | 0.0600472 | 0 | 0.0858137 | 0.446891 | 0 |
| 840 | lnc_HBrBl_010 | 0.0588616 | 0.0176652 | 0.0669605 | 0.420924 | 0.0262449 |
| 841 | U17 | 0.0417812 | 0.0439701 | 0.063645 | 0.440862 | 0.00501242 |
| 842 | P763 | 0.0681672 | 0 | 0.195602 | 0.283409 | 0.0597387 |
| 843 | P835 | 0.0616645 | 0.119457 | 0.152077 | 0.224999 | 0.0677228 |
| 844 | U569 | 0 | 0.0905891 | 0 | 0.233031 | 0 |
| 845 | U500 | 0.029458 | 0.114101 | 0.0622303 | 0.252776 | 0.0117728 |
| 846 | U544 | 0.0076472 | 0.0885278 | 0.0800894 | 0.327511 | 0.00504581 |
| 847 | P319 | 0 | 0.0449975 | 0.0653367 | 0.269026 | 0 |
| 848 | P181 | 0.0254218 | 0.0441089 | 0.0637827 | 0.265919 | 0.010067 |
| 849 | P335 | 0 | 0 | 0.0662377 | 0.205699 | 0.0399431 |
| 850 | U557 | 0 | 0 | 0.0694236 | 0.247869 | 0.0218095 |
| 851 | lnc_HMBr_026 | 0.0029897 | 0.00107911 | 0.027257 | 0.248168 | 0.000987068 |
| 852 | P91 | 0 | 0 | 0.0521194 | 0.25114 | 0 |
| 853 | P199 | 0 | 0 | 0 | 0.254795 | 0 |
| 854 | U273 | 0.0090477 | 0 | 0.0190922 | 0.270773 | 0 |
| 855 | lnc_HBr_015 | 0.0118297 | 0 | 0.0165219 | 0.290478 | 0.00520356 |
| 856 | U35 | 0.0525433 | 0 | 0 | 0.325736 | 0 |
| 857 | lnc_HMBr_021 | 0.047556 | 0.00560767 | 0.0425189 | 0.28508 | 0.00128151 |
| 858 | U93 | 0 | 0 | 0.0439963 | 0.305581 | 0 |
| 859 | U16 | 0 | 0 | 0.0647093 | 0.339206 | 0 |
| 860 | P383 | 0.0078095 | 0 | 0 | 0.333961 | 0.0102973 |
| 861 | P305 | 0.0060522 | 0 | 0.0381509 | 0.340637 | 0.0319322 |
| 862 | P863 | 0 | 0 | 0.288216 | 0.555645 | 0 |
| 863 | U259.U260 | 0.0758958 | 0.0661627 | 0.262341 | 0.397465 | 0.116187 |
| 864 | lnc_MBrBl_006 | 0 | 0 | 0.235673 | 0.410867 | 0.0357216 |
| 865 | U672 | 0.0320912 | 0 | 0.203633 | 0.460085 | 0.0845785 |
| 866 | lnc_Br_003 | 0 | 0 | 0 | 0.587364 | 0 |
| 867 | P984 | 0 | 0 | 0.0372262 | 0.623769 | 0 |
| 868 | lnc_Br_004 | 0 | 0 | 0 | 0.632268 | 0 |
| 869 | P938 | 0 | 0 | 0 | 0.642795 | 0 |
| 870 | P1116 | 0 | 0 | 0.0686613 | 0.462668 | 0 |
| 871 | lnc_Br_039 | 0.0121353 | 0.00737922 | 0.0639277 | 0.492344 | 0.00337364 |
| 872 | U193 | 0 | 0 | 0.0812375 | 0.532415 | 0 |
| 873 | U474 | 0 | 0 | 0.0461955 | 0.517239 | 0 |
| 874 | P1090 | 0 | 0 | 0.0477785 | 0.540949 | 0 |
| 875 | P277.P278.P279 | 0.122111 | 0 | 0.0411981 | 0.708819 | 0.0918246 |
| 876 | P1034 | 0.084368 | 0.0552071 | 0.0997143 | 0.602638 | 0.134505 |
| 877 | U571 | 0.159168 | 0 | 0 | 0.493456 | 0.0261691 |
| 878 | P1031 | 0.263213 | 0 | 0.0311039 | 0.478426 | 0 |
| 879 | U517 | 0.176436 | 0 | 0.186357 | 0.500238 | 0.129193 |
| 880 | P952 lnc_HLMBrBl_005 | 0.164089 | 0.073579 | 0.100492 | 0.535009 | 0.0458086 |
| 881 | P197 | 0.101537 | 0 | 0.0758997 | 0.538024 | 0.00423653 |
| 882 | U116 | 0.107194 | 0 | 0.0880834 | 0.536517 | 0 |
| 883 | lnc_Br_009 | 0.0977231 | 0.065647 | 0.105854 | 0.449673 | 0.0540173 |
| 884 | U475 | 0.141304 | 0.0227209 | 0.106895 | 0.433697 | 0.072515 |
| 885 | U673 | 0.135747 | 0.0218252 | 0.0789761 | 0.491969 | 0.104498 |
| 886 | U207 | 0.164994 | 0.038762 | 0.0839971 | 0.474452 | 0.0850124 |
| 887 | P823 | 0.162217 | 0 | 0.381431 | 0.229788 | 0.316076 |
| 888 | U653 | 0.355909 | 0.054401 | 0.454779 | 0.136449 | 0.271607 |
| 889 | U236 | 0.18322 | 0.228575 | 0.305535 | 0.192085 | 0.310275 |
| 890 | P530 | 0.152162 | 0.111147 | 0.284999 | 0.0156964 | 0.30026 |
| 891 | P324 | 0.119787 | 0.199759 | 0.292239 | 0.0562701 | 0.382854 |
| 892 | U95 | 0.470723 | 0.0172356 | 0.0373976 | 0.0309526 | 0.401089 |
| 893 | P1049 | 0.285184 | 0 | 0 | 0.0356242 | 0.41915 |
| 894 | U397 | 0.314437 | 0.0357384 | 0.0388702 | 0.0198328 | 0.528598 |
| 895 | U361 | 0.220513 | 0.0313849 | 0.060412 | 0.00360077 | 0.224231 |
| 896 | U355 | 0.252901 | 0.0212131 | 0.0307019 | 0.0234652 | 0.295054 |
| 897 | U371 | 0.229797 | 0.1358 | 0.0688464 | 0.0864945 | 0.272259 |
| 898 | P23 | 0.239139 | 0.138502 | 0.0601898 | 0.00383594 | 0.309206 |
| 899 | P784 | 0.214618 | 0.125609 | 0.0922065 | 0.0355996 | 0.310416 |
| 900 | P976 | 0.2157 | 0.0649634 | 0.122092 | 0.286893 | 0.403382 |
| 901 | P732 | 0.306178 | 0.111163 | 0.242464 | 0.32538 | 0.353123 |
| 902 | P375 | 0.312947 | 0.133257 | 0.144755 | 0.110693 | 0.151806 |
| 903 | P213.U121 | 0.367842 | 0.0390204 | 0.115046 | 0.214364 | 0.190163 |
| 904 | U576 | 0.335261 | 0.0888406 | 0.227104 | 0.137282 | 0.381199 |
| 905 | U175 | 0.409818 | 0.104044 | 0.190375 | 0.0733431 | 0.281354 |
| 906 | U428 | 0.362662 | 0.123597 | 0.302264 | 0.119899 | 0.260326 |
| 907 | lnc_HLBl_001 | 0.200345 | 0.402354 | 0 | 0 | 0.522005 |
| 908 | U537 | 0.248161 | 0.105878 | 0.10914 | 0.0854441 | 0.423817 |
| 909 | U213 | 0.209783 | 0.203002 | 0.165933 | 0.113052 | 0.437887 |
| 910 | U181 | 0.225936 | 0.089245 | 0.0615373 | 0.0969407 | 0.569 |
| 911 | U351 | 0.191028 | 0.151374 | 0.0964937 | 0.155918 | 0.57967 |
| 912 | P124 | 0 | 0 | 0.214962 | 0.0869169 | 0.467381 |
| 913 | lnc_BL_011 | 0.102383 | 0.024698 | 0.191355 | 0.0533904 | 0.302641 |
| 914 | U162 | 0.109691 | 0.0374096 | 0.217051 | 0.11942 | 0.289351 |
| 915 | P722 | 0.117231 | 0 | 0.0636818 | 0 | 0.424696 |
| 916 | U618 | 0.158085 | 0.0285707 | 0.0206558 | 0.00591619 | 0.453214 |
| 917 | P11 | 0.101403 | 0 | 0 | 0.0346874 | 0.463883 |
| 918 | U461 | 0.0860135 | 0 | 0.0201818 | 0 | 0.415703 |
| 919 | P41 | 0.0714885 | 0 | 0.0200403 | 0.0238583 | 0.441579 |
| 920 | P513.P514 | 0.0192197 | 0 | 0.0134075 | 0.0230503 | 0.448141 |
| 921 | P164 | 0.0128848 | 0 | 0.00244434 | 0.00559678 | 0.49646 |
| 922 | P1087 | 0.0135896 | 0 | 0 | 0 | 0.57272 |
| 923 | U64 | 0.0108123 | 0 | 0.0282646 | 0.00215907 | 0.574435 |
| 924 | U292 | 0.0456318 | 0 | 0 | 0 | 0.569842 |
| 925 | U488 | 0.0411468 | 0.00383385 | 0.0179909 | 0.0306226 | 0.570512 |
| 926 | P970 | 0.0539684 | 0.0222866 | 0.0241586 | 0.0199835 | 0.562342 |
| 927 | U138 | 0.0123488 | 0 | 0.0261812 | 0.18098 | 0.292814 |
| 928 | lnc_Br_012 | 0.104113 | 0.139725 | 0.104852 | 0.189764 | 0.242987 |
| 929 | U577 | 0.143104 | 0 | 0.0667493 | 0.0893596 | 0.174804 |
| 930 | U233 | 0.208488 | 0.0341146 | 0.0369812 | 0.101969 | 0.215515 |
| 931 | lnc_HLMBrBl_053 | 0.159238 | 0.0772643 | 0.0563421 | 0.0108244 | 0.157297 |
| 932 | U662 | 0.101457 | 0 | 0.0412631 | 0.00230137 | 0.137635 |
| 933 | P607.P608 | 0.110627 | 0 | 0.0337547 | 0.0129257 | 0.18715 |
| 934 | P233 | 0.124465 | 0.107739 | 0.0310039 | 0.0770825 | 0.316288 |
| 935 | P74 | 0.0419793 | 0.0811561 | 0.0736051 | 0.0169083 | 0.286035 |
| 936 | U389 | 0.0276742 | 0.145504 | 0.00525996 | 0.00401756 | 0.312166 |
| 937 | U668 | 0.0414171 | 0.0871721 | 0.0157719 | 0.0271236 | 0.367689 |
| 938 | P860 | 0.0853435 | 0 | 0.0817093 | 0.0438156 | 0.245525 |
| 939 | P903 | 0 | 0.0472322 | 0 | 0 | 0.236056 |
| 940 | U429 | 0.0054026 | 0.0626042 | 0.022683 | 0 | 0.19956 |
| 941 | U129 | 0.0180394 | 0 | 0.056733 | 0 | 0.255874 |
| 942 | lnc_BL_010 | 0.0101987 | 0.00453238 | 0.0065449 | 0.00187282 | 0.217546 |
| 943 | P377 | 0 | 0 | 0.0256959 | 0.0197336 | 0.207633 |
| 944 | P452 | 0 | 0 | 0 | 0 | 0.272256 |
| 945 | U412 | 0.0226143 | 0 | 0 | 0 | 0.327007 |
| 946 | P47 | 0 | 0 | 0 | 0.0120392 | 0.297324 |
| 947 | U217 | 0.007385 | 0 | 0.00773285 | 0.00886535 | 0.311865 |
| 948 | U77 | 0.0110484 | 0 | 0.0750987 | 0.00220647 | 0.331766 |
| 949 | U420 | 0.0792189 | 0.0143173 | 0.0258776 | 0.0158119 | 0.323513 |
| 950 | lnc_MBl_002 | 0.0489885 | 0.0125811 | 0.0249862 | 0.0086679 | 0.277422 |
| 951 | P845 | 0.0576425 | 0 | 0.00670179 | 0.0230436 | 0.295857 |
| 952 | lnc_HMBl_013 | 0.0691485 | 0.00499588 | 0.0108053 | 0.00137323 | 0.30474 |
| 953 | P811.P812 | 0.231177 | 0 | 0.0302361 | 0.17256 | 0 |
| 954 | U187 | 0.155005 | 0 | 0 | 0.157335 | 0 |
| 955 | P1128 | 0.158632 | 0 | 0 | 0.140591 | 0.0690821 |
| 956 | P430.U266 | 0.200406 | 0.13496 | 0.117234 | 0.239221 | 0.0245982 |
| 957 | lnc_HMBr_030 | 0.284316 | 0 | 0.111757 | 0.259566 | 0 |
| 958 | P114.P115.P117 | 0.281226 | 0.0572271 | 0.127028 | 0.219644 | 0.0392141 |
| 959 | U165 | 0.087423 | 0.0374697 | 0.108424 | 0.0828475 | 0.119655 |
| 960 | P960 | 0.0890456 | 0.0404352 | 0.103254 | 0.143237 | 0.072667 |
| 961 | U190 | 0.146193 | 0.0150993 | 0.0545881 | 0.11675 | 0.0999318 |
| 962 | P1114 | 0.163459 | 0.0602529 | 0.0508615 | 0.14437 | 0.0687978 |
| 963 | P62 | 0.165938 | 0.0657235 | 0.0285054 | 0.143316 | 0.135031 |
| 964 | U381 | 0.128312 | 0.112008 | 0.0556702 | 0.125625 | 0.124635 |
| 965 | P518 | 0.179375 | 0.0182075 | 0.118535 | 0.181138 | 0.0623012 |
| 966 | U409 | 0.128143 | 0 | 0.0997537 | 0.233398 | 0.0629329 |
| 967 | P760 | 0.132295 | 0 | 0.152993 | 0.239309 | 0.0820472 |
| 968 | P410 | 0.0947058 | 0 | 0.102049 | 0.216984 | 0.155587 |
| 969 | U245 | 0.197331 | 0 | 0.0967881 | 0.236343 | 0.166204 |
| 970 | U683 | 0.180823 | 0.0530565 | 0.133331 | 0.254703 | 0.130635 |
| 971 | U173 | 0.13039 | 0 | 0.146485 | 0.0000144 | 0.0675849 |
| 972 | P958 | 0.152329 | 0.0375062 | 0.10853 | 0.0518304 | 0.0855509 |
| 973 | P250 | 0.163861 | 0 | 0.143145 | 0 | 0 |
| 974 | P899 | 0.210986 | 0 | 0.101008 | 0.0195219 | 0 |
| 975 | P75.P76 | 0.104988 | 0.00819276 | 0.103572 | 0.0530835 | 0.00187156 |
| 976 | P726 | 0.11157 | 0 | 0.079559 | 0 | 0 |
| 977 | P155 | 0.13056 | 0.00421468 | 0.0549257 | 0.021843 | 0.00193264 |
| 978 | U588 | 0.242041 | 0 | 0.0757166 | 0.0589344 | 0 |
| 979 | U125 | 0.241039 | 0 | 0.0753872 | 0.0880091 | 0.0451564 |
| 980 | U322 | 0.171916 | 0 | 0.0461647 | 0 | 0.0565118 |
| 981 | P297 | 0.20157 | 0 | 0.0580116 | 0 | 0.0724661 |
| 982 | U308 | 0.147519 | 0.0334255 | 0.0301715 | 0.0307002 | 0.0432859 |
| 983 | P1039 | 0.1821 | 0.0351227 | 0.0685878 | 0.0434046 | 0.0150452 |
| 984 | U561 | 0.217683 | 0 | 0 | 0 | 0 |
| 985 | P788.P789 | 0.178141 | 0 | 0 | 0 | 0.0263858 |
| 986 | P550 | 0.182609 | 0 | 0.0193255 | 0.00737955 | 0.00915395 |
| 987 | U524 | 0.209423 | 0 | 0 | 0.0595316 | 0.0457679 |
| 988 | U135 | 0.207196 | 0.0166471 | 0.0120393 | 0.0321912 | 0.083563 |
| 989 | P758 | 0.283072 | 0 | 0.210541 | 0 | 0.0617086 |
| 990 | U123 | 0.319296 | 0.00940951 | 0.100762 | 0.0202987 | 0.0114726 |
| 991 | P547 | 0.338957 | 0.0345069 | 0.169083 | 0.0299077 | 0.0333161 |
| 992 | P1026 | 0.321767 | 0 | 0 | 0 | 0 |
| 993 | U360 | 0.309519 | 0.0620434 | 0 | 0.0519657 | 0 |
| 994 | U128 | 0.307297 | 0 | 0.101857 | 0.0995691 | 0.149355 |
| 995 | U235 | 0.313518 | 0.0769185 | 0.0896029 | 0.02985 | 0.164644 |
| 996 | U335 | 0.301192 | 0 | 0.0222574 | 0.01701 | 0.163672 |
| 997 | P1081 | 0.243638 | 0.00527219 | 0.0418769 | 0.0123471 | 0.106031 |
| 998 | P810 | 0.279718 | 0 | 0.0302224 | 0.0426357 | 0.0769312 |
| 999 | U91 | 0.310573 | 0.00542127 | 0.0665501 | 0.0358493 | 0.0792922 |
| 1000 | lnc_HLMBr_003 | 0.170485 | 0.249424 | 0.0915368 | 0.0176689 | 0 |
| 1001 | P373 | 0.143942 | 0.131035 | 0.0737203 | 0.0671883 | 0 |
| 1002 | lnc_HLMBrBl_037 | 0.17903 | 0.16498 | 0.0618936 | 0.0591322 | 0.0743274 |
| 1003 | P918 | 0.208633 | 0.141213 | 0.108034 | 0.0725288 | 0.0942339 |
| 1004 | U358 | 0.143893 | 0.130314 | 0.242776 | 0.0986327 | 0.119853 |
| 1005 | U197 | 0.147043 | 0.144809 | 0.0916539 | 0.0700292 | 0.123876 |
| 1006 | U60 | 0.0989526 | 0.143748 | 0.10976 | 0.0617509 | 0.164833 |
| 1007 | P616 | 0.127233 | 0.102154 | 0.103339 | 0.0859423 | 0.16564 |
| 1008 | U651.U652 | 0.150657 | 0.120469 | 0.130912 | 0.113258 | 0.180692 |
| 1009 | P792 | 0.0170722 | 0.143281 | 0.141214 | 0 | 0 |
| 1010 | P315 | 0.0503282 | 0.166602 | 0.119698 | 0.0816892 | 0.0282067 |
| 1011 | P818 | 0.0700305 | 0.216974 | 0.151641 | 0.0579114 | 0.0132058 |
| 1012 | P827 | 0.0698802 | 0.101017 | 0.124705 | 0.0324358 | 0.0651213 |
| 1013 | U395 | 0.0941142 | 0.110673 | 0.114654 | 0.0722466 | 0.0296938 |
| 1014 | U313 | 0.0739598 | 0.0862094 | 0.0944134 | 0.0363091 | 0 |
| 1015 | P851 | 0.0637602 | 0.0929678 | 0.101909 | 0.0261454 | 0.0209829 |
| 1016 | U547.U548.U549 | 0.0933118 | 0.201629 | 0.0371552 | 0.028297 | 0.116537 |
| 1017 | P868 | 0.0852493 | 0.140826 | 0.0508891 | 0.00161911 | 0.0589497 |
| 1018 | P397 | 0.0824493 | 0.189346 | 0.0342019 | 0.0261864 | 0.0642619 |
| 1019 | U638 | 0.0980068 | 0.189933 | 0.0691187 | 0.0176811 | 0.0573984 |
| 1020 | U44 | 0.0076879 | 0.177788 | 0.00803002 | 0.0168611 | 0.119255 |
| 1021 | P352 | 0 | 0.116311 | 0.0211486 | 0 | 0.065954 |
| 1022 | P942.P943 | 0 | 0.154872 | 0 | 0 | 0.0693023 |
| 1023 | U232 | 0.0447629 | 0.0946307 | 0.0637853 | 0.0147781 | 0.0922605 |
| 1024 | P90 | 0.0594834 | 0.110156 | 0.0511484 | 0.0379391 | 0.0647861 |
| 1025 | U205 | 0.0208978 | 0.155441 | 0.0499668 | 0.0477183 | 0.0945686 |
| 1026 | U166 | 0.0197022 | 0.136859 | 0.0660403 | 0.088343 | 0.0831979 |
| 1027 | P831 | 0 | 0.258401 | 0.0966134 | 0 | 0 |
| 1028 | P275 | 0 | 0.246488 | 0 | 0 | 0 |
| 1029 | P944 | 0.0196121 | 0.265328 | 0.0137459 | 0.00526142 | 0.0258666 |
| 1030 | U481 | 0 | 0.290415 | 0.0116522 | 0.00978232 | 0.0191706 |
| 1031 | P166 | 0 | 0.294283 | 0 | 0 | 0 |
| 1032 | U270 | 0.011799 | 0.295967 | 0 | 0.00314909 | 0 |
| 1033 | P302 | 3.66E-06 | 0.18067 | 0.00000368 | 0.121 | 0.00000371 |
| 1034 | P465 | 0.0064329 | 0.179098 | 0.0133885 | 0.136239 | 0.0170268 |
| 1035 | U413 | 0.0697143 | 0.134512 | 0.008113 | 0.0775176 | 0.0408876 |
| 1036 | U51 | 0.071835 | 0.149978 | 0.0375195 | 0.119371 | 0.0474162 |
| 1037 | U317 | 0.018119 | 0.105821 | 0 | 0.0447762 | 0.0238405 |
| 1038 | P993 | 0.0459913 | 0.0983737 | 0 | 0.0663341 | 0.0226068 |
| 1039 | P1051 | 0.0331497 | 0.0821185 | 0.0428479 | 0.0578718 | 0.0291774 |
| 1040 | P697 | 0.0367473 | 0.0868976 | 0.0453448 | 0.0426069 | 0.039694 |
| 1041 | U648 | 0.0034667 | 0.0701392 | 0.00361995 | 0.00552744 | 0.0251721 |
| 1042 | P824 | 0 | 0.0931661 | 0.016842 | 0.0107209 | 0.0248042 |
| 1043 | U403 | 0 | 0.101838 | 0 | 0.0143501 | 0 |
| 1044 | U296 | 0.0071054 | 0.0957646 | 0.0148129 | 0.00941772 | 0.00562985 |
| 1045 | U513 | 0 | 0.0648492 | 0 | 0.00905817 | 0 |
| 1046 | P950 | 0 | 0.0695196 | 0 | 0.00972164 | 0 |
| 1047 | P638 | 0 | 0.0750313 | 0 | 0 | 0 |
| 1048 | P326 | 0 | 0.0855187 | 0 | 0 | 0 |
| 1049 | U631 | 0.0624909 | 0.121864 | 0 | 0.0172537 | 0 |
| 1050 | U137 | 0.0399105 | 0.137812 | 0.0166352 | 0.0127281 | 0 |
| 1051 | U325 | 0 | 0.139747 | 0 | 0.00977195 | 0.0158223 |
| 1052 | U311 | 0 | 0.119962 | 0 | 0 | 0 |
| 1053 | U534 | 0 | 0.116984 | 0 | 0 | 0.0132665 |
| 1054 | P545 | 0 | 0.113573 | 0.0387881 | 0 | 0 |
| 1055 | U339 | 0 | 0.12465 | 0.0680332 | 0 | 0.0282563 |
| 1056 | P327 | 0 | 0.177971 | 0 | 0 | 0 |
| 1057 | P57 | 0.016243 | 0.200913 | 0.0324838 | 0.00996104 | 0.00851775 |
| 1058 | U457 | 0.0260519 | 0.151253 | 0.0183153 | 0.0631704 | 0.0114483 |
| 1059 | P548 | 0.0565947 | 0.164475 | 0.0291004 | 0.0654393 | 0.0184162 |
| 1060 | U337 | 0.0289513 | 0.16761 | 0.0260007 | 0.00662576 | 0.0545757 |
| 1061 | U439 | 0.0435624 | 0.169229 | 0.0308822 | 0.0237493 | 0.0191202 |
| 1062 | P182.P183.P184 | 0.026925 | 0.133479 | 0.0676654 | 0.0443557 | 0.0425756 |
| 1063 | U343 | 0.0129906 | 0.147697 | 0.0540251 | 0.0206751 | 0 |
| 1064 | U293 | 0 | 0.159986 | 0.0387609 | 0.0520047 | 0.0242086 |
| 1065 | P1010 | 0.0322041 | 0.0939222 | 0.205938 | 0.0660493 | 0.0211955 |
| 1066 | P414 | 0.0016535 | 0.0286733 | 0.245148 | 0.00790794 | 0.00218298 |
| 1067 | U332 | 0.0359136 | 0.0445522 | 0.241216 | 0.038855 | 0.00338676 |
| 1068 | lnc_HLMBr_013 | 0.0200652 | 0.0582968 | 0.106002 | 0.0162616 | 0.0132226 |
| 1069 | U76 | 0 | 0 | 0.12492 | 0 | 0.0193305 |
| 1070 | U347 | 0.0046697 | 0 | 0.13665 | 0.00186362 | 0.00924642 |
| 1071 | U418 | 0 | 0 | 0.0967482 | 0 | 0 |
| 1072 | U678 | 0.0086143 | 0 | 0.108999 | 0.0208849 | 0 |
| 1073 | P1109 | 0 | 0 | 0.114585 | 0.0146404 | 0.0119301 |
| 1074 | P797 | 0.0203795 | 0 | 0.153849 | 0.0212934 | 0.0269247 |
| 1075 | P87 | 0.0275848 | 0 | 0.175815 | 0.022525 | 0.0363277 |
| 1076 | P96 | 0.0389491 | 0 | 0.171689 | 0 | 0 |
| 1077 | U315 | 0 | 0 | 0.183614 | 0 | 0 |
| 1078 | P629 | 0.0077917 | 0.0207698 | 0.173639 | 0.0073855 | 0.00197879 |
| 1079 | P510 | 0 | 0 | 0.0669135 | 0.0768969 | 0 |
| 1080 | P1070 | 0 | 0 | 0.0677091 | 0.10366 | 0 |
| 1081 | P1110.P1111 | 0.005831 | 0.00996153 | 0.0943085 | 0.0629887 | 0.00723098 |
| 1082 | P321.P322 | 0 | 0 | 0.115652 | 0.0925451 | 0 |
| 1083 | U183 | 0 | 0.0379115 | 0.0824919 | 0.0631496 | 0.0517434 |
| 1084 | U540 | 0.0294546 | 0.0284343 | 0.0411937 | 0.0708883 | 0 |
| 1085 | P113 | 0.0208065 | 0.0501151 | 0.0470741 | 0.0511448 | 0.0137345 |
| 1086 | P200 | 0.0366883 | 0.052847 | 0.0415151 | 0.077046 | 0.02132 |
| 1087 | P325 | 0 | 0 | 0 | 0.110126 | 0.0795937 |
| 1088 | P1 | 0.016526 | 0 | 0 | 0.108554 | 0.0652547 |
| 1089 | U277 | 0.039532 | 0 | 0 | 0.0816835 | 0 |
| 1090 | lnc_HLBrBl_010 | 0.0412814 | 0.00492569 | 0.0163517 | 0.0652131 | 0.0173466 |
| 1091 | lnc_HLMBrBl_001 | 0.0280658 | 0.0187061 | 0.0168794 | 0.0497962 | 0.00997822 |
| 1092 | U67 | 0.0228252 | 0.0188673 | 0.0136491 | 0.0521474 | 0.0172141 |
| 1093 | P201 | 0.031757 | 0.0193232 | 0.010471 | 0.0932637 | 0.0264799 |
| 1094 | lnc_MBr_020 | 0.0144316 | 0.0024206 | 0.0265751 | 0.0796541 | 0.0257815 |
| 1095 | P912 | 0.0190472 | 0.0084683 | 0.0367051 | 0.0933967 | 0.0270823 |
| 1096 | U79 | 0.0304782 | 0 | 0.0324432 | 0.112321 | 0.020063 |
| 1097 | P53 | 0.0346723 | 0 | 0.0613515 | 0.10651 | 0.0105614 |
| 1098 | U686 | 0.0220809 | 0 | 0 | 0.124602 | 0 |
| 1099 | U509 | 0 | 0 | 0 | 0.115087 | 0 |
| 1100 | U372 | 0.0032273 | 0.00466126 | 0.00841391 | 0.104653 | 0 |
| 1101 | U353 | 0.0025554 | 0 | 0.0213332 | 0.11907 | 0.00168698 |
| 1102 | P188 | 0.0077058 | 0.00317944 | 0.0172139 | 0.129585 | 0.000726842 |
| 1103 | lnc_Br_023 | 0.0074811 | 0.00864385 | 0.0171623 | 0.127385 | 0.000987861 |
| 1104 | U156 | 0 | 0 | 0 | 0.0818345 | 0 |
| 1105 | U19 | 0.0097129 | 0 | 0 | 0.0956242 | 0 |
| 1106 | U349 | 0.012786 | 0 | 0.0101165 | 0.0773421 | 0.00562381 |
| 1107 | P734 | 0 | 0 | 0.0107506 | 0.0759026 | 0.00170023 |
| 1108 | P644 | 0.0034613 | 0.00151335 | 0.0199687 | 0.0744463 | 0.00457028 |
| 1109 | U139 | 0 | 0 | 0.0210714 | 0.0808108 | 0 |
| 1110 | U625 | 0 | 0 | 0.0163173 | 0.085169 | 0.00343354 |
| 1111 | P477 | 0.0039159 | 0.0453493 | 0.0164111 | 0.0815448 | 0 |
| 1112 | lnc_MBr_024 | 0 | 0 | 0.0221771 | 0.0935839 | 0 |
| 1113 | P107 | 0.0107255 | 0.0142998 | 0.0206501 | 0.102425 | 0.0065366 |
| 1114 | lnc_LMBr_003 | 0 | 0.0262652 | 0.0285299 | 0.0981711 | 0.00598459 |
| 1115 | P257.P259 | 0 | 0.0222484 | 0.0333827 | 0.112672 | 0.0030126 |
| 1116 | U503 | 0.0836359 | 0.0597285 | 0.0869009 | 0.195523 | 0 |
| 1117 | P578 | 0.0801369 | 0 | 0.0348415 | 0.22475 | 0.028888 |
| 1118 | P136 | 0.0864945 | 0.0173462 | 0.0348475 | 0.210356 | 0.0495179 |
| 1119 | P537 | 0.0302952 | 0 | 0 | 0.153527 | 0 |
| 1120 | lnc_Br_041 | 0 | 0 | 0 | 0.173014 | 0 |
| 1121 | P533 | 0 | 0 | 0 | 0.141152 | 0 |
| 1122 | lnc_Br_042 | 0 | 0 | 0 | 0.151914 | 0 |
| 1123 | P387 | 0 | 0 | 0.00664653 | 0.154894 | 0.00419185 |
| 1124 | P1040 | 0.0049004 | 0.00707567 | 0.0287259 | 0.16119 | 0.0052581 |
| 1125 | U66 | 0 | 0 | 0.0594523 | 0.176412 | 0.00931497 |
| 1126 | lnc_Br_045 | 0.0041916 | 0.0103751 | 0.0243364 | 0.203742 | 0.00514 |
| 1127 | P433.U3 | 0.0136428 | 0 | 0 | 0.197756 | 0 |
| 1128 | U656 | 0 | 0 | 0 | 0.19258 | 0 |
| 1129 | P690 | 0 | 0 | 0 | 0.206073 | 0 |
| 1130 | U592 | 0.0203074 | 0.0839119 | 0.078893 | 0.141405 | 0.00434219 |
| 1131 | U368 | 0.0885143 | 0.0454888 | 0.045202 | 0.144334 | 0.0103869 |
| 1132 | U621 | 0.0384866 | 0.0123631 | 0.0446792 | 0.151829 | 0.0282257 |
| 1133 | P522 | 0.0526763 | 0.0306932 | 0.0425375 | 0.174575 | 0.0379171 |
| 1134 | U658 | 0.0356128 | 0.0686497 | 0 | 0.159193 | 0.0532784 |
| 1135 | P1092 | 0.028871 | 0.0557385 | 0.0302789 | 0.162097 | 0.0698349 |
| 1136 | P1011.P1012 | 0.0109763 | 0.0706944 | 0.0286393 | 0.118038 | 0.019815 |
| 1137 | P1124 | 0.016927 | 0.0729733 | 0 | 0.140333 | 0.0200801 |
| 1138 | P611 | 0.0285137 | 0.0281084 | 0.0291098 | 0.125425 | 0.00290471 |
| 1139 | P620.P621 | 0.0179031 | 0.0352603 | 0.0210334 | 0.167392 | 0 |
| 1140 | P641.P642 | 0.0260976 | 0.0368625 | 0.0136291 | 0.160402 | 0.00842056 |
| 1141 | U399 | 0 | 0 | 0.0802554 | 0.00138125 | 0.0901118 |
| 1142 | U680 | 0 | 0 | 0.075142 | 0.029239 | 0.135053 |
| 1143 | P853 | 0.0588435 | 0 | 0 | 0 | 0.154802 |
| 1144 | U374 | 0.0521488 | 0 | 0.0049466 | 0.00377542 | 0.118932 |
| 1145 | U464 | 0.0730901 | 0 | 0 | 0 | 0.0950976 |
| 1146 | P1029 | 0.0683818 | 0 | 0 | 0 | 0.105116 |
| 1147 | P108 | 0.0213256 | 0 | 0 | 0.00865244 | 0.127502 |
| 1148 | P247 | 0 | 0 | 0 | 0.0252563 | 0.145364 |
| 1149 | lnc_BL_009 | 0.0107558 | 0 | 0.0042055 | 0 | 0.171325 |
| 1150 | U237 | 0.0065892 | 0 | 0 | 0 | 0.156427 |
| 1151 | P292 | 0.0017228 | 0 | 0.00359797 | 0.00274692 | 0.163765 |
| 1152 | U32 | 0.062326 | 0.0524921 | 0.0260345 | 0.00144787 | 0.169267 |
| 1153 | P745 | 0.0464525 | 0.0845725 | 0.0316816 | 0.0115786 | 0.145125 |
| 1154 | P685 | 0.0299352 | 0.0579801 | 0.0210842 | 0.0727743 | 0.144664 |
| 1155 | U65 | 0.0503314 | 0.026497 | 0 | 0.0660286 | 0.10867 |
| 1156 | U294 | 0.0869786 | 0.0193578 | 0.00700241 | 0.0668864 | 0.136868 |
| 1157 | U601 | 0.0528826 | 0.0179977 | 0.0585829 | 0.0248669 | 0.12317 |
| 1158 | P609 | 0.0871627 | 0 | 0.0571848 | 0.0568743 | 0.143732 |
| 1159 | P478 | 0.0388258 | 0.0519481 | 0.0870512 | 0.0424415 | 0.153948 |
| 1160 | P554 | 0.0132421 | 0.00342051 | 0.0367297 | 0.0448374 | 0.169203 |
| 1161 | U269 | 0.0277314 | 0 | 0.0567554 | 0.0385099 | 0.189079 |
| 1162 | U37 | 0.0728211 | 0.0574597 | 0.0554257 | 0.0158823 | 0.0218428 |
| 1163 | P185 | 0.0875105 | 0.0660686 | 0.00796935 | 0.00609137 | 0 |
| 1164 | P821.P822 | 0.0701422 | 0.05687 | 0.00685693 | 0.023578 | 0.0270451 |
| 1165 | U38 | 0.0917996 | 0.053314 | 0 | 0.0668444 | 0 |
| 1166 | U113 | 0.0803333 | 0.0583499 | 0.0212198 | 0.0813831 | 0.052938 |
| 1167 | P761 | 0.0721414 | 0.0850706 | 0.0433988 | 0.0760927 | 0.0277693 |
| 1168 | U132 | 0.143067 | 0 | 0 | 0.0305572 | 0 |
| 1169 | U634 | 0.123937 | 0 | 0 | 0.0787344 | 0.0350108 |
| 1170 | P807.U492 | 0.124183 | 0 | 0 | 0.074779 | 0.0545137 |
| 1171 | P288 | 0.0844278 | 0 | 0 | 0 | 0.0739067 |
| 1172 | U423 | 0.103227 | 0 | 0 | 0 | 0.0339025 |
| 1173 | P290 | 0.119705 | 0 | 0.0210778 | 0.00808356 | 0.0657369 |
| 1174 | U449 | 0.0961493 | 0.0301941 | 0.0457907 | 0.0299541 | 0.0772773 |
| 1175 | U253 | 0.107809 | 0 | 0.0616806 | 0.0275185 | 0.0387924 |
| 1176 | lnc_HBr_023 | 0.0736656 | 0.0212715 | 0.0477458 | 0.0290992 | 0.0462132 |
| 1177 | U688 | 0.0933889 | 0.0257524 | 0.0556999 | 0.0355018 | 0.0293258 |
| 1178 | U521 | 0 | 0.0593412 | 0 | 0 | 0.0942021 |
| 1179 | U431 | 0.0311739 | 0.0722855 | 0.0262081 | 0.0150446 | 0.0904597 |
| 1180 | U405 | 0.0556997 | 0.0429046 | 0.0201349 | 0.0183185 | 0.0637438 |
| 1181 | U239 | 0.0645199 | 0.0679879 | 0.0123213 | 0.0377149 | 0.0773706 |
| 1182 | P1082 | 0.0098148 | 0.0709255 | 0.0462075 | 0.0117367 | 0.0485629 |
| 1183 | U328 | 0.0337458 | 0.0433536 | 0.00783221 | 0.0179403 | 0.0371253 |
| 1184 | lnc_MBrBl_008 | 0.0365748 | 0.0363064 | 0.042283 | 0.0304386 | 0.0369804 |
| 1185 | P58 | 0 | 0 | 0.0122932 | 0.00234781 | 0.0620495 |
| 1186 | U532 | 0.0046207 | 0 | 0.00968946 | 0.00370476 | 0.079257 |
| 1187 | P253 | 0.0158689 | 0 | 0 | 0 | 0.0418815 |
| 1188 | P551 | 0.0230871 | 0 | 0 | 0 | 0.0507375 |
| 1189 | U201.U202 | 0.016757 | 0 | 0 | 0 | 0.0663463 |
| 1190 | P553 | 0.0252247 | 0 | 0 | 0 | 0.0661826 |
| 1191 | U192 | 0.0090922 | 0 | 0 | 0.0147097 | 0.107869 |
| 1192 | U350 | 0.0236765 | 0 | 0 | 0.0197258 | 0.0933522 |
| 1193 | P757 | 0 | 0 | 0 | 0 | 0.0961424 |
| 1194 | lnc_BrBl_003 | 0 | 0 | 0 | 0 | 0.0943287 |
| 1195 | P515 | 6.93E-06 | 0 | 0.00000828 | 0.00000618 | 0.094451 |
| 1196 | U282 | 0.0422102 | 0 | 0.0482605 | 0 | 0.0470035 |
| 1197 | U126 | 0.0398094 | 0 | 0.0563421 | 0.0324731 | 0.0699099 |
| 1198 | P552 | 0 | 0 | 0.0462263 | 0 | 0.0565846 |
| 1199 | U414 | 0.0056779 | 0 | 0.0475053 | 0.011341 | 0.0749428 |
| 1200 | U437 | 0 | 0 | 0.0314313 | 0.0240741 | 0.0492145 |
| 1201 | U216 | 0.0105279 | 0.0176223 | 0.0219797 | 0.0203741 | 0.0595703 |
| 1202 | P162 | 0.0232796 | 0.00122222 | 0.037488 | 0.0117751 | 0.0525437 |
| 1203 | P190 | 0.0259904 | 0.00715936 | 0.0414021 | 0.0256847 | 0.0457699 |
| 1204 | U447 | 0.0269624 | 0 | 0.0346685 | 0.00330916 | 0.0109519 |
| 1205 | P225 | 0.0348123 | 0 | 0.0229621 | 0.00438295 | 0.00241888 |
| 1206 | U279 | 0.0392768 | 0 | 0.0277871 | 0.0106757 | 0 |
| 1207 | U200 | 0.0375275 | 0 | 0.0594207 | 0.00826663 | 0 |
| 1208 | P497 | 0.0530158 | 0 | 0.0562735 | 0.0108111 | 0 |
| 1209 | U478 | 0.0728767 | 0 | 0.0576738 | 0.0589535 | 0 |
| 1210 | P630.P631 | 0.0613175 | 0 | 0.0491281 | 0.0262014 | 0.0123821 |
| 1211 | P692 | 0.063426 | 0 | 0.0337876 | 0.0390058 | 0.0208733 |
| 1212 | U650 | 0.0776145 | 0 | 0 | 0.0578899 | 0.00731338 |
| 1213 | U597 | 0.0806957 | 0 | 0.00939893 | 0.0171445 | 0.0236611 |
| 1214 | U493.U494 | 0.0827737 | 0.00579859 | 0.0088679 | 0.0407529 | 0.0223818 |
| 1215 | P80 | 0.065432 | 0 | 0 | 0.0292988 | 0.0479366 |
| 1216 | U416 | 0.0685227 | 0 | 0 | 0.0464065 | 0.0451423 |
| 1217 | P682 | 0.0380748 | 0 | 0.00723708 | 0.00553057 | 0 |
| 1218 | P840 | 0.0521316 | 0 | 0 | 0 | 0 |
| 1219 | P794 | 0.0543983 | 0 | 0 | 0.0125395 | 0.0102469 |
| 1220 | P229 | 0.0604864 | 0.0204045 | 0.00736371 | 0.0126675 | 0.0212465 |
| 1221 | P887 | 0.0623898 | 0.0152681 | 0.0203857 | 0.00924665 | 0.0307227 |
| 1222 | P847 | 0.0719831 | 0 | 0 | 0 | 0 |
| 1223 | P736 | 0.087736 | 0 | 0 | 0 | 0 |
| 1224 | U458 | 0.0660711 | 0 | 0.0352302 | 0.0135623 | 0 |
| 1225 | U263 | 0.0826087 | 0 | 0.0250008 | 0 | 0 |
| 1226 | U448 | 0.0844244 | 0 | 0.0396919 | 0.00168388 | 0.011145 |
| 1227 | U456 | 0 | 0 | 0.0727012 | 0 | 0.0220131 |
| 1228 | P914 | 0.0031288 | 0.00924146 | 0.0594786 | 0 | 0.0327299 |
| 1229 | U364 | 0 | 0 | 0.0360549 | 0 | 0.0222359 |
| 1230 | P876 | 0.0084279 | 0 | 0.0441571 | 0.0202555 | 0.0166814 |
| 1231 | U357 | 0.0121592 | 0 | 0.0515447 | 0.00989634 | 0 |
| 1232 | U630 | 0 | 0 | 0.0397056 | 0 | 0 |
| 1233 | P1073 | 0 | 0 | 0.0460115 | 0 | 0 |
| 1234 | P1084 | 0 | 0 | 0.049188 | 0 | 0 |
| 1235 | U392 | 0.0088352 | 0 | 0.0559202 | 0.0357197 | 0.034945 |
| 1236 | P781 | 0 | 0 | 0.0706967 | 0.0274763 | 0.0424926 |
| 1237 | P524 | 0 | 0 | 0.0665952 | 0.00851585 | 0 |
| 1238 | U286 | 0.0165503 | 0 | 0.0705732 | 0 | 0 |
| 1239 | P836 | 0 | 0 | 0.0762758 | 0.0293862 | 0 |
| 1240 | P12 | 0 | 0 | 0.0878649 | 0.0252788 | 0 |
| 1241 | P451 | 0 | 0 | 0 | 0.0556565 | 0.0466658 |
| 1242 | P800 | 0 | 0 | 0 | 0.0789046 | 0.0181789 |
| 1243 | U433 | 0 | 0 | 0.0104832 | 0.0786992 | 0.0375566 |
| 1244 | U533 | 0 | 0 | 0 | 0.0451081 | 0.0184616 |
| 1245 | lnc_BrBl_002 | 0 | 0 | 0 | 0.0441784 | 0.0241555 |
| 1246 | P9 | 0.0082806 | 0 | 0.0138332 | 0.0264019 | 0.0327968 |
| 1247 | P1033 | 0 | 0 | 0 | 0.0208953 | 0.0328629 |
| 1248 | P173 | 0.0095683 | 0 | 0 | 0.0306964 | 0.0252486 |
| 1249 | P941 | 0 | 0.0309599 | 0 | 0.0297148 | 0.0355493 |
| 1250 | U167 | 0.0160837 | 0.023277 | 0.0168493 | 0.0386408 | 0.0265299 |
| 1251 | P284 | 0.0142922 | 0.0196418 | 0.00744893 | 0.0552325 | 0.0367538 |
| 1252 | U675 | 0 | 0 | 0.0138038 | 0.0491538 | 0.00194227 |
| 1253 | lnc_Br_007 | 0.0011944 | 0 | 0.0066392 | 0.0455856 | 0.00893787 |
| 1254 | P683 | 0.0061661 | 0 | 0.00716488 | 0.0524925 | 0.00406917 |
| 1255 | P207 | 0 | 0 | 0.00386959 | 0.0558234 | 0 |
| 1256 | P521 | 0 | 0 | 0 | 0.0526015 | 0 |
| 1257 | P348 | 0 | 0 | 0 | 0.0486759 | 0 |
| 1258 | P409 | 0 | 0 | 0 | 0.0494071 | 0 |
| 1259 | U375 | 0 | 0 | 0.0167973 | 0.0643459 | 0 |
| 1260 | P534 | 0.0029652 | 0.00856916 | 0.0123809 | 0.0673392 | 0.00195746 |
| 1261 | U383 | 0 | 0 | 0 | 0.0655351 | 0 |
| 1262 | P649.P650 | 0.0059044 | 0.0113789 | 0.00261081 | 0.0565213 | 0.00165172 |
| 1263 | U208 | 0 | 0.00761897 | 0.00550332 | 0.0619595 | 0 |
| 1264 | lnc_HBr_002 | 0.0033778 | 0.00750315 | 0.00812276 | 0.0607248 | 0.000343137 |
| 1265 | P55 | 0.018487 | 0.0176865 | 0.00647862 | 0.0266247 | 0.0102185 |
| 1266 | P135 | 0.0191988 | 0.0206061 | 0.00617358 | 0.0382742 | 0.00431759 |
| 1267 | P151 | 0.0056817 | 0.0164335 | 0 | 0.036316 | 0.0037497 |
| 1268 | P289 | 0.0034881 | 0.0201856 | 0 | 0.0390657 | 0.00460334 |
| 1269 | U525 | 0.00406 | 0.0117369 | 0.00848256 | 0.0372441 | 0.0107194 |
| 1270 | P588 | 0.0035094 | 0.0203094 | 0.0146951 | 0.0364987 | 0.00463149 |
| 1271 | P934 | 0 | 0 | 0 | 0.0319497 | 0 |
| 1272 | P741 | 0 | 0 | 0 | 0.0239641 | 0 |
| 1273 | P632 | 0 | 0 | 0 | 0.0250668 | 0 |
| 1274 | U321 | 0 | 0 | 0 | 0.0263269 | 0 |
| 1275 | P1085.P1086 | 0 | 0 | 0 | 0.0267813 | 0 |
| 1276 | U582 | 0 | 0 | 0 | 0.0352071 | 0.00579668 |
| 1277 | P407 | 0 | 0 | 0 | 0.03237 | 0.0105749 |
| 1278 | P816 | 0 | 0 | 0.0049497 | 0.0340225 | 0.00312577 |
| 1279 | lnc_MBrBl_005 | 0 | 0 | 0.00650127 | 0.0347712 | 0.00820139 |
| 1280 | U660 | 0.0050361 | 0 | 0.00630414 | 0.0328756 | 0 |
| 1281 | P712 | 0.0051834 | 0.00390403 | 0.00563299 | 0.0354859 | 0.00178541 |
| 1282 | P585.P586 | 0.0047812 | 0 | 0.00386943 | 0.0431177 | 0 |
| 1283 | P95 | 0.0008231 | 0.00475564 | 0.00686751 | 0.0412688 | 0 |
| 1284 | P215 | 0 | 0 | 0 | 0.0425626 | 0 |
| 1285 | P293.P294 | 0 | 0 | 0 | 0.0398352 | 0 |
| 1286 | P1115 | 0 | 0 | 0 | 0.0385862 | 0 |
| 1287 | U482 | 4.85E-07 | 0 | 0 | 0.0379 | 0.000000488 |
| 1288 | P1056 | 0 | 0 | 0 | 0.0381979 | 0 |
| 1289 | P593.P594.P595 | 0.0019709 | 0.0656699 | 0.029867 | 0.0293974 | 0 |
| 1290 | P678 | 0 | 0.0545555 | 0.0198296 | 0 | 0 |
| 1291 | P596 | 0.0092281 | 0.0535964 | 0.0389568 | 0 | 0 |
| 1292 | P501 | 0.0161727 | 0.0326875 | 0.0116427 | 0 | 0 |
| 1293 | U163 | 0.0166511 | 0.0321615 | 0.0349628 | 0 | 0 |
| 1294 | P577 | 0.0220719 | 0.0189738 | 0.0273756 | 0.0210996 | 0.00465156 |
| 1295 | P900 | 0.0311291 | 0.0290026 | 0.0238782 | 0.0243013 | 0.015448 |
| 1296 | P768 | 0.0078423 | 0.0302391 | 0.0273306 | 0.0146134 | 0.0276042 |
| 1297 | P349 | 0.0131791 | 0.030491 | 0.0330706 | 0.0273661 | 0.0139168 |
| 1298 | U359 | 0.0261064 | 0.0505239 | 0 | 0 | 0 |
| 1299 | U518 | 0.0324944 | 0.0627576 | 0 | 0.0150769 | 0.014289 |
| 1300 | U611 | 0.0128461 | 0.0469421 | 0.0191658 | 0 | 0.0363413 |
| 1301 | P252 lnc_HLMBrBl_038 | 0.0070399 | 0.0271143 | 0.011646 | 0.00571284 | 0.0247894 |
| 1302 | U462 | 0.0044074 | 0.0254855 | 0.00921074 | 0.00175849 | 0.0232728 |
| 1303 | P617 | 0.0050096 | 0.0290184 | 0.0105108 | 0.00401968 | 0.019828 |
| 1304 | P599 | 0 | 0.0308546 | 0 | 0.00400033 | 0.0133858 |
| 1305 | U154 | 0.0034716 | 0.0267483 | 0 | 0.00276454 | 0.00152794 |
| 1306 | U477 | 0 | 0.028336 | 0 | 0 | 0.00645298 |
| 1307 | P709 | 0 | 0.0403734 | 0 | 0 | 0 |
| 1308 | P84 | 0 | 0.0396649 | 0 | 0.0055081 | 0 |
| 1309 | U510 | 0 | 0.0513888 | 0 | 0.00715573 | 0 |
| 1310 | P318 | 0.0008014 | 0.0509315 | 0.00167154 | 0.00446428 | 0.00105826 |
| 1311 | P570 | 0.0093001 | 0.0539741 | 0.00487296 | 0.021809 | 0.00913455 |
| 1312 | P401 | 0.0125685 | 0.0364306 | 0.0132089 | 0.0202206 | 0 |
| 1313 | P214 | 0.002549 | 0.0294617 | 0.01064 | 0.0121819 | 0.00168278 |
| 1314 | P65 | 0.0064354 | 0.0363835 | 0.0136167 | 0.0103117 | 0 |
| 1315 | U224.U227 | 0.0041363 | 0.0438138 | 0.014578 | 0.0202239 | 0.0100145 |
| 1316 | lnc_LMBrBl_003 | 0 | 0.045372 | 0.0164201 | 0.0125515 | 0.00517171 |
| 1317 | U320 | 0 | 0.0427637 | 0.0155184 | 0.0170418 | 0 |
| 1318 | P1108 | 0 | 0.0470692 | 0.0167909 | 0.0190242 | 0 |
| 1319 | U159 | 0.0182866 | 0.0176326 | 0 | 0.0121802 | 0.020113 |
| 1320 | U168 | 0.0244118 | 0.0235539 | 0 | 0.0195514 | 0.00536888 |
| 1321 | U356 | 0.0253805 | 0 | 0 | 0.00168743 | 0.0195446 |
| 1322 | U435 | 0.0236789 | 0 | 0 | 0.00951698 | 0.0234257 |
| 1323 | U507 | 0.0179603 | 0 | 0.0150049 | 0.00143202 | 0.00711332 |
| 1324 | P202 | 0.0255306 | 0 | 0.0162133 | 0.00530316 | 0.00146566 |
| 1325 | P995 | 0.024402 | 0 | 0 | 0.00259331 | 0.0107388 |
| 1326 | P1097 | 0.0259254 | 0 | 0 | 0 | 0 |
| 1327 | P848 | 0.0212273 | 0 | 0 | 0 | 0 |
| 1328 | U434 | 0.0212244 | 0 | 0 | 0.00283014 | 0 |
| 1329 | U419 | 0.0126548 | 0 | 0 | 0.0151757 | 0.0125265 |
| 1330 | P203 | 0.0114326 | 0 | 0.0120055 | 0.00918624 | 0.00754055 |
| 1331 | P506 | 0.0090714 | 0 | 0.00947969 | 0.0126693 | 0.00898119 |
| 1332 | P881 | 0.0125728 | 0 | 0 | 0.0102414 | 0 |
| 1333 | U376 | 0.0136007 | 0 | 0 | 0 | 0 |
| 1334 | P526 | 0.0107316 | 0 | 0 | 0 | 0 |
| 1335 | P1038 | 0.0110341 | 0 | 0 | 0 | 0 |
| 1336 | P626 | 0.0139354 | 0 | 0.00969327 | 0.00462385 | 0 |
| 1337 | P146.P147 | 0.0091913 | 0 | 0.00641048 | 0.00939011 | 0 |
| 1338 | P576 | 0.0094416 | 0.00237106 | 0.00342288 | 0.00391701 | 0.00216841 |
| 1339 | P1064 | 0.0104259 | 0 | 0.00651208 | 0.00413511 | 0.00068802 |
| 1340 | P981 | 0.0115263 | 0 | 0.00450703 | 0.00343904 | 0.0028538 |
| 1341 | P674 | 0.0375593 | 0 | 0.0198239 | 0.0380007 | 0 |
| 1342 | P1030 | 0.0400797 | 0 | 0 | 0.0331445 | 0 |
| 1343 | P922 | 0.0428684 | 0 | 0 | 0.0177759 | 0 |
| 1344 | lnc_HBr_016 | 0.0489957 | 0 | 0 | 0.0238137 | 0 |
| 1345 | U108 | 0.019482 | 0 | 0.0136539 | 0.0365824 | 0.0171301 |
| 1346 | U598 | 0.0198382 | 0 | 0 | 0.0319372 | 0.0174429 |
| 1347 | P1133 | 0.0297705 | 0 | 0 | 0.0300038 | 0.0196286 |
| 1348 | U169 | 0.0215732 | 0 | 0 | 0.0178962 | 0.0283653 |
| 1349 | P969 | 0.0296875 | 0 | 0 | 0.0248658 | 0.0387791 |
| 1350 | lnc_HMBrBl_008 | 0.0281872 | 0 | 0.013593 | 0.0138405 | 0.020036 |
| 1351 | P769 | 0.0355515 | 0 | 0.0190252 | 0.021137 | 0.0240242 |
| 1352 | U589 | 0 | 0 | 0.00543228 | 0 | 0.0342925 |
| 1353 | P837 | 0 | 0 | 0 | 0 | 0.0319785 |
| 1354 | P123 | 0 | 0 | 0 | 0 | 0.0372352 |
| 1355 | P212 | 0.0092876 | 0 | 0.0196052 | 0.00751585 | 0.0367277 |
| 1356 | U145 | 0.0100386 | 0 | 0.0212133 | 0.0000343 | 0.026461 |
| 1357 | U480 | 0 | 0 | 0.0176655 | 0.00224923 | 0.0223946 |
| 1358 | P178 | 0 | 0 | 0.0243851 | 0 | 0.0303442 |
| 1359 | P664 | 0 | 0 | 0 | 0.00470296 | 0.0158072 |
| 1360 | P139 | 0.0013133 | 0.00284408 | 0.00547327 | 0.00978524 | 0.0140917 |
| 1361 | P684 | 0.0018007 | 0.0103467 | 0.00180135 | 0.00139836 | 0.0155162 |
| 1362 | P419 | 0.00091 | 0.00788333 | 0.0028447 | 0.00397838 | 0.0195289 |
| 1363 | U417 | 0 | 0 | 0 | 0.014767 | 0.0235973 |
| 1364 | U73 | 0 | 0 | 0 | 0.0127204 | 0.0204364 |
| 1365 | P5 | 0 | 0 | 0 | 0.0105366 | 0.0184735 |
| 1366 | P634 | 0 | 0 | 0 | 0.0113933 | 0.0183687 |
| 1367 | P1065 | 0 | 0 | 0.0076078 | 0 | 0.0190302 |
| 1368 | U82 | 0 | 0 | 0 | 0 | 0.0228369 |
| 1369 | P657 | 0 | 0 | 0 | 0 | 0.0255602 |
| 1370 | P844 | 0.0074386 | 0 | 0 | 0 | 0.0294497 |
| 1371 | P841 | 0.006988 | 0 | 0.0054661 | 0.00486641 | 0.0242216 |
| 1372 | P861 | 0.0033457 | 0.00480469 | 0.014563 | 0.00317393 | 0.00905635 |
| 1373 | P990 | 0.0108133 | 0.00891978 | 0.0218458 | 0.00833805 | 0.00615419 |
| 1374 | P691 | 0.0073327 | 0 | 0.0308714 | 0 | 0.00966961 |
| 1375 | U398 | 0 | 0 | 0.0240885 | 0 | 0 |
| 1376 | P218 | 0 | 0 | 0.0259363 | 0 | 0 |
| 1377 | U98 | 0 | 0 | 0.0294394 | 0 | 0 |
| 1378 | U617 | 0 | 0 | 0.0317159 | 0 | 0 |
| 1379 | P20 | 0 | 0 | 0.0322022 | 0 | 0 |
| 1380 | U632 | 0 | 0 | 0.0322428 | 0 | 0 |
| 1381 | P157.P159.P160 | 0.0081051 | 0.00230231 | 0.0228003 | 0.031446 | 0.0089941 |
| 1382 | U252 | 0 | 0 | 0.0137777 | 0.0245502 | 0.00580206 |
| 1383 | P26 | 0.0012743 | 0 | 0.0132799 | 0.0311523 | 0.00126212 |
| 1384 | P713.P714 | 0.0044911 | 0 | 0.0116123 | 0.0183549 | 0.00118603 |
| 1385 | P260 | 0.0067291 | 0 | 0.0141529 | 0.0162534 | 0 |
| 1386 | P665 | 0 | 0 | 0.0179849 | 0.0177058 | 0 |
| 1387 | U144 | 0 | 0 | 0.019179 | 0.021881 | 0 |
| 1388 | U177 | 0 | 0 | 0.0147367 | 0.0112843 | 0.00923664 |
| 1389 | lnc_HMBrBl_009 | 0.0029934 | 0.00576536 | 0.0187352 | 0.0158865 | 0.00658756 |
| 1390 | P511 | 0 | 0 | 0.0223125 | 0.00915271 | 0.00693369 |
| 1391 | U41 | 0.0057085 | 0 | 0.0239778 | 0.0137602 | 0.00753021 |
| 1392 | P19 | 0.0035404 | 0.0204891 | 0 | 0.0141628 | 0 |
| 1393 | P933 | 0.0032707 | 0.0189249 | 0.00684542 | 0.0261537 | 0 |
| 1394 | P883 | 0.0004025 | 0.00699226 | 0.00503431 | 0.0224286 | 0.000532997 |
| 1395 | P231 | 0.000776 | 0.00896597 | 0.00485515 | 0.0240811 | 0 |
| 1396 | P773.P776 | 0.0095569 | 0.0059401 | 0.00435522 | 0.0229399 | 0.00638178 |
| 1397 | U579 | 0.0073086 | 0 | 0 | 0.0235644 | 0 |
| 1398 | lnc_MBr_028 | 0.008798 | 0 | 0.00306119 | 0.021031 | 0 |
| 1399 | P915 | 0 | 0 | 0.0058237 | 0.0183301 | 0.000921908 |
| 1400 | P1019 | 0 | 0 | 0 | 0.0183943 | 0 |
| 1401 | P251 | 0 | 0 | 0 | 0.0217829 | 0 |
| 1402 | P817 | 0 | 0 | 0 | 0.0202709 | 0 |
| 1403 | lnc_Br_038 | 0 | 0 | 0 | 0.0205921 | 0 |
| 1404 | U584 | 0 | 0 | 0 | 0.0209439 | 0 |
| 1405 | P536 | 0 | 0 | 0 | 0.0210115 | 0 |
| 1406 | P540 | 0.0033703 | 0.00353917 | 0.00574736 | 0.0143713 | 0.00202311 |
| 1407 | P276 | 0.0012489 | 0.00721315 | 0.00390456 | 0.0171273 | 0.000412326 |
| 1408 | P261 | 0.005142 | 0 | 0 | 0.0164906 | 0 |
| 1409 | P98 | 0.0013281 | 0 | 0.00287521 | 0.01321 | 0 |
| 1410 | P660 | 0 | 0 | 0 | 0.014255 | 0.00214556 |
| 1411 | P978 | 0 | 0 | 0 | 0.016095 | 0 |
| 1412 | U251 | 0 | 0 | 0 | 0.0144602 | 0 |
| 1413 | P256 | 0 | 0 | 0 | 0.0151478 | 0 |
| 1414 | P480 | 0.0144839 | 0.0119866 | 0.00865024 | 0.00826398 | 0 |
| 1415 | U424 | 0.0130891 | 0.0189343 | 0.0068488 | 0.00523332 | 0.00431875 |
| 1416 | P705 | 0.009087 | 0.0238633 | 0.0103381 | 0.00328703 | 0.00218163 |
| 1417 | U258 | 0 | 0.0195807 | 0 | 0.00270641 | 0 |
| 1418 | P985 | 0.002003 | 0.0214469 | 0.00813696 | 0.00156242 | 0 |
| 1419 | U231 | 0.0062528 | 0.0180622 | 0.00163014 | 0.00746344 | 0.00412835 |
| 1420 | P862 | 0.0065613 | 0.0200277 | 0.00351547 | 0.002756 | 0.00779872 |
| 1421 | P89 | 0 | 0.00763709 | 0.00272548 | 0.00928863 | 0.00176794 |
| 1422 | P255 | 0.0010771 | 0.00829449 | 0.00224504 | 0.011989 | 0.00047412 |
| 1423 | P1021.P1022 | 0 | 0.0110995 | 0 | 0.00884359 | 0.00486104 |
| 1424 | P700 | 0.0014414 | 0.0104063 | 0.00375551 | 0.0117466 | 0.00523453 |
| 1425 | P503 | 0 | 0.00797047 | 0.00287875 | 0 | 0 |
| 1426 | P974 | 0 | 0.0122054 | 0 | 0.00084087 | 0 |
| 1427 | P1112 | 0 | 0.0107115 | 0.00774037 | 0.00295496 | 0 |
| 1428 | U445 | 0 | 0.0131014 | 0.00473102 | 0.00451347 | 0.00299363 |
| 1429 | P408 | 0.0027658 | 0.00799235 | 0.00577332 | 0.00550861 | 0.0036517 |
| 1430 | P403 | 0.0040375 | 0.0116597 | 0.00631165 | 0.00401252 | 0.00355462 |
| 1431 | P361 | 0.0078684 | 0.00649306 | 0.00351544 | 0.00261914 | 0.00074214 |
| 1432 | P474 | 0.0048906 | 0.0075323 | 0.00475693 | 0.00233288 | 0.00129169 |
| 1433 | P189 | 0.006259 | 0.00730779 | 0.00652149 | 0.00449235 | 0.00165482 |
| 1434 | P746 | 0.0033408 | 0.00771961 | 0.0111464 | 0.00372088 | 0.000882293 |
| 1435 | P485.P486 | 0.0037746 | 0.0062297 | 0.00786992 | 0.00128659 | 0 |
| 1436 | U268 | 0.0014999 | 0.00433245 | 0.00625603 | 0.00119342 | 0 |
| 1437 | P940 | 0.0022901 | 0.00661426 | 0.00477481 | 0.00136615 | 0.000756019 |
| 1438 | P948 | 0 | 0 | 0.0120456 | 0.00460736 | 0 |
| 1439 | P767 | 0 | 0 | 0.0115359 | 0.00882585 | 0 |
| 1440 | P992 | 0 | 0 | 0.0161289 | 0.0123549 | 0 |
| 1441 | U486 | 0.0011468 | 0 | 0.0167518 | 0.00913236 | 0.0030285 |
| 1442 | P549 | 0 | 0 | 0.0171803 | 0.006582 | 0 |
| 1443 | U120 | 0 | 0 | 0.0173611 | 0.00665156 | 0 |
| 1444 | P118 | 0 | 0 | 0.014408 | 0 | 0.00454149 |
| 1445 | P195 | 0 | 0 | 0.0154024 | 0.00196066 | 0 |
| 1446 | P1052 | 0 | 0 | 0.0121316 | 0 | 0 |
| 1447 | P973 | 0 | 0 | 0.0120143 | 0.00151088 | 0.0024944 |
| 1448 | P79 | 0.0043455 | 0 | 0.00910881 | 0 | 0 |
| 1449 | P695 | 0.0037883 | 0 | 0.0105417 | 0.00201154 | 0 |
| 1450 | U535 | 0.0011618 | 0 | 0.00727326 | 0.00092519 | 0 |
| 1451 | P961 | 0.0021606 | 0 | 0.00720625 | 0.00137444 | 0 |
| 1452 | P356 | 0 | 0 | 0.00828365 | 0 | 0 |
| 1453 | P508 | 0 | 0 | 0.00907304 | 0 | 0 |
| 1454 | U83 | 0 | 0 | 0.0087911 | 0 | 0 |
| 1455 | P400 | 0 | 0 | 0.00891401 | 0 | 0 |
| 1456 | U314 | 0.0055219 | 0 | 0 | 0.00882221 | 0.0109328 |
| 1457 | P357 | 0 | 0 | 0 | 0.0102376 | 0.00839166 |
| 1458 | P42 | 0 | 0 | 0 | 0.0127349 | 0.00697552 |
| 1459 | P715 | 0 | 0 | 0 | 0.00971436 | 0 |
| 1460 | P1101 | 0 | 0 | 0 | 0.00848449 | 0 |
| 1461 | P130 | 0 | 0 | 0 | 0.00877014 | 0 |
| 1462 | P388 | 0 | 0 | 0 | 0.0107197 | 0 |
| 1463 | P509 | 0 | 0 | 0 | 0.0110208 | 0 |
| 1464 | P628 | 0 | 0 | 0 | 0.0116754 | 0 |
| 1465 | P838 | 0 | 0 | 0 | 0.0123232 | 0 |
| 1466 | U562 | 0 | 0 | 0 | 0.0124274 | 0 |
| 1467 | P517 | 0 | 0 | 0.00703082 | 0.0081335 | 0.0044329 |
| 1468 | P196 | 0.0005291 | 0.00152786 | 0.00275673 | 0.0063089 | 0.000349363 |
| 1469 | P221 | 0.0006614 | 0.00254658 | 0.00275679 | 0.00630887 | 0.000873488 |
| 1470 | P150 | 0.0009464 | 0.00182208 | 0.00394527 | 0.0100324 | 0.000833245 |
| 1471 | P270 | 0.0009547 | 0.00180473 | 0.00398075 | 0.0100233 | 0.00165062 |
| 1472 | P834 | 0.0016408 | 0 | 0.00342634 | 0.00784729 | 0.0021663 |
| 1473 | U581 | 0.0015768 | 0 | 0.00329223 | 0.00879654 | 0 |
| 1474 | P519 | 0.0018664 | 0 | 0.00190804 | 0.00800527 | 0.000604328 |
| 1475 | P273 | 0.0010845 | 0 | 0.00225172 | 0.00875344 | 0.00172559 |
| 1476 | P175 | 0.011286 | 0.00181065 | 0.00261367 | 0.00249235 | 0.00828016 |
| 1477 | P852 | 0.006427 | 0.00543165 | 0.00293986 | 0.0041113 | 0.010351 |
| 1478 | P858 | 0.0063654 | 0.00144154 | 0.00286076 | 0.00138865 | 0.0084064 |
| 1479 | P919 | 0.0046477 | 0.00188346 | 0.00424731 | 0.00291575 | 0.00484582 |
| 1480 | P267 | 0.0057429 | 0 | 0.00599032 | 0.00228572 | 0.0050554 |
| 1481 | P676 | 0.0026122 | 0.00503073 | 0.00544877 | 0.00277198 | 0.00574878 |
| 1482 | U88 | 0.0030822 | 0.00593611 | 0 | 0.00081854 | 0.00406944 |
| 1483 | U203 | 0.0008837 | 0.00765662 | 0.00184224 | 0.00210823 | 0.00700206 |
| 1484 | P946 | 0 | 0 | 0 | 0.00383232 | 0.0110846 |
| 1485 | P369 | 0 | 0 | 0 | 0 | 0.0084104 |
| 1486 | P604 | 0 | 0 | 0 | 0 | 0.00947666 |
| 1487 | U124 | 0 | 0 | 0 | 0 | 0.00339114 |
| 1488 | U404 | 0 | 0 | 0 | 0 | 0.00347179 |
| 1489 | P997 | 0 | 0 | 0.00310772 | 0 | 0.00589601 |
| 1490 | U671 | 0.0023383 | 0 | 0 | 0.00186638 | 0.00617337 |
| 1491 | P605.P606 | 0.0040085 | 0 | 0.00185697 | 0.00460436 | 0.00473225 |
| 1492 | P568 | 0.0021883 | 0 | 0.00273712 | 0.00452464 | 0.00346789 |
| 1493 | P347 | 0.0010994 | 0 | 0.00458806 | 0.00437701 | 0.00290333 |
| 1494 | P193.P194.U115 | 0.0056326 | 0 | 0.00470144 | 0.00279874 | 0.00148733 |
| 1495 | P525 | 0.0063909 | 0 | 0.00490767 | 0.00454623 | 0.000888403 |
| 1496 | P587 | 0.0006596 | 0 | 0.00481174 | 0.00104879 | 0.00130657 |
| 1497 | P313 | 0 | 0 | 0.00439846 | 0 | 0 |
| 1498 | P304 | 0 | 0 | 0.0055613 | 0 | 0 |
| 1499 | P921 | 0.0023041 | 0 | 0.00435343 | 0 | 0 |
| 1500 | P575 | 0.0033587 | 0 | 0.00350695 | 0.00133868 | 0 |
| 1501 | P298.P299.P301 | 0.0034978 | 0 | 0.00486603 | 0.00092847 | 0 |
| 1502 | U305 | 0.0024826 | 0 | 0.00519025 | 0.00396424 | 0 |
| 1503 | P2 | 0 | 0 | 0.00398454 | 0.00304236 | 0 |
| 1504 | P16 | 0 | 0 | 0.00397517 | 0.0045528 | 0 |
| 1505 | P138 | 0 | 0 | 0.00648211 | 0.00467035 | 0.00045632 |
| 1506 | P1123 | 0 | 0 | 0.00710084 | 0.00271058 | 0 |
| 1507 | P88 | 0 | 0 | 0.00717641 | 0.00274207 | 0 |
| 1508 | P935 | 0.0042338 | 0 | 0 | 0.00339195 | 0 |
| 1509 | U271 | 0.002427 | 0 | 0 | 0 | 0 |
| 1510 | P544 | 0.0016165 | 0 | 0.00067387 | 0.00051407 | 0 |
| 1511 | P584 | 0.003401 | 0 | 0.00166759 | 0.00095403 | 0.000264158 |
| 1512 | P161 | 0.0066831 | 0 | 0 | 0 | 0 |
| 1513 | P786 | 0.0045209 | 0 | 0 | 0 | 0 |
| 1514 | P982 | 0.0046681 | 0 | 0 | 0 | 0 |
| 1515 | P499 | 0.004719 | 0 | 0 | 0 | 0 |
| 1516 | P27 | 0.0011324 | 0 | 0.00188785 | 0.00396031 | 0.000598161 |
| 1517 | P314 | 0.0005572 | 0 | 0.00232342 | 0.00221588 | 0.00147155 |
| 1518 | P45 | 0.0005438 | 0 | 0.0022625 | 0.00205667 | 0 |
| 1519 | P677 | 0 | 0 | 0.0023077 | 0.00264186 | 0 |
| 1520 | U42 | 0 | 0 | 0 | 4.48E-07 | 0.000410238 |
| 1521 | U68 | 0 | 0 | 0 | 0 | 0 |
| 1522 | U670 | 0 | 0 | 0 | 0 | 0 |
| 1523 | U647 | 0 | 0 | 0 | 0 | 0 |
| 1524 | U639 | 0 | 0 | 0 | 0 | 0 |
| 1525 | U593 | 0 | 0 | 0 | 0 | 0 |
| 1526 | U53 | 0 | 0 | 0 | 0 | 0 |
| 1527 | U520 | 0 | 0 | 0 | 0 | 0 |
| 1528 | U511 | 0 | 0 | 0 | 0 | 0 |
| 1529 | U440 | 0 | 0 | 0 | 0 | 0 |
| 1530 | U430 | 0 | 0 | 0 | 0 | 0 |
| 1531 | U43 | 0 | 0 | 0 | 0 | 0 |
| 1532 | U316 | 0 | 0 | 0 | 0 | 0 |
| 1533 | U312 | 0 | 0 | 0 | 0 | 0 |
| 1534 | U304 | 0 | 0 | 0 | 0 | 0 |
| 1535 | U299 | 0 | 0 | 0 | 0 | 0 |
| 1536 | U281 | 0 | 0 | 0 | 0 | 0 |
| 1537 | U195 | 0 | 0 | 0 | 0 | 0 |
| 1538 | U172 | 0 | 0 | 0 | 0 | 0 |
| 1539 | U161 | 0 | 0 | 0 | 0 | 0 |
| 1540 | U153 | 0 | 0 | 0 | 0 | 0 |
| 1541 | U13 | 0 | 0 | 0 | 0 | 0 |
| 1542 | P972 | 0 | 0 | 0 | 0 | 0 |
| 1543 | P959 | 0 | 0 | 0 | 0 | 0 |
| 1544 | P93 | 0 | 0 | 0 | 0 | 0 |
| 1545 | P924 | 0 | 0 | 0 | 0 | 0 |
| 1546 | P920 | 0 | 0 | 0 | 0 | 0 |
| 1547 | P901 | 0 | 0 | 0 | 0 | 0 |
| 1548 | P873 | 0 | 0 | 0 | 0 | 0 |
| 1549 | P856.P857 | 0 | 0 | 0 | 0 | 0 |
| 1550 | P855 | 0 | 0 | 0 | 0 | 0 |
| 1551 | P854 | 0 | 0 | 0 | 0 | 0 |
| 1552 | P850 | 0 | 0 | 0 | 0 | 0 |
| 1553 | P85 | 0 | 0 | 0 | 0 | 0 |
| 1554 | P846 | 0 | 0 | 0 | 0 | 0 |
| 1555 | P839 | 0 | 0 | 0 | 0 | 0 |
| 1556 | P829 | 0 | 0 | 0 | 0 | 0 |
| 1557 | P819 | 0 | 0 | 0 | 0 | 0 |
| 1558 | P81 | 0 | 0 | 0 | 0 | 0 |
| 1559 | P795 | 0 | 0 | 0 | 0 | 0 |
| 1560 | P780 | 0 | 0 | 0 | 0 | 0 |
| 1561 | P771 | 0 | 0 | 0 | 0 | 0 |
| 1562 | P723.P725 | 0 | 0 | 0 | 0 | 0 |
| 1563 | P710 | 0 | 0 | 0 | 0 | 0 |
| 1564 | P70 | 0 | 0 | 0 | 0 | 0 |
| 1565 | P675 | 0 | 0 | 0 | 0 | 0 |
| 1566 | P652 | 0 | 0 | 0 | 0 | 0 |
| 1567 | P627 | 0 | 0 | 0 | 0 | 0 |
| 1568 | P618 | 0 | 0 | 0 | 0 | 0 |
| 1569 | P582 | 0 | 0 | 0 | 0 | 0 |
| 1570 | P543 | 0 | 0 | 0 | 0 | 0 |
| 1571 | P523 | 0 | 0 | 0 | 0 | 0 |
| 1572 | P507 | 0 | 0 | 0 | 0 | 0 |
| 1573 | P505 | 0 | 0 | 0 | 0 | 0 |
| 1574 | P500 | 0 | 0 | 0 | 0 | 0 |
| 1575 | P496 | 0 | 0 | 0 | 0 | 0 |
| 1576 | P488 | 0 | 0 | 0 | 0 | 0 |
| 1577 | P467 | 0 | 0 | 0 | 0 | 0 |
| 1578 | P463 | 0 | 0 | 0 | 0 | 0 |
| 1579 | P462 | 0 | 0 | 0 | 0 | 0 |
| 1580 | P443 | 0 | 0 | 0 | 0 | 0 |
| 1581 | P413 | 0 | 0 | 0 | 0 | 0 |
| 1582 | P406 | 0 | 0 | 0 | 0 | 0 |
| 1583 | P389 | 0 | 0 | 0 | 0 | 0 |
| 1584 | P384 | 0 | 0 | 0 | 0 | 0 |
| 1585 | P378 | 0 | 0 | 0 | 0 | 0 |
| 1586 | P363 | 0 | 0 | 0 | 0 | 0 |
| 1587 | P36 | 0 | 0 | 0 | 0 | 0 |
| 1588 | P359 | 0 | 0 | 0 | 0 | 0 |
| 1589 | P341 | 0 | 0 | 0 | 0 | 0 |
| 1590 | P334 | 0 | 0 | 0 | 0 | 0 |
| 1591 | P330 | 0 | 0 | 0 | 0 | 0 |
| 1592 | P323 | 0 | 0 | 0 | 0 | 0 |
| 1593 | P295 | 0 | 0 | 0 | 0 | 0 |
| 1594 | P287 | 0 | 0 | 0 | 0 | 0 |
| 1595 | P285 | 0 | 0 | 0 | 0 | 0 |
| 1596 | P271 | 0 | 0 | 0 | 0 | 0 |
| 1597 | P264 | 0 | 0 | 0 | 0 | 0 |
| 1598 | P235 | 0 | 0 | 0 | 0 | 0 |
| 1599 | P198 | 0 | 0 | 0 | 0 | 0 |
| 1600 | P177 | 0 | 0 | 0 | 0 | 0 |
| 1601 | P17 | 0 | 0 | 0 | 0 | 0 |
| 1602 | P15 | 0 | 0 | 0 | 0 | 0 |
| 1603 | P149 | 0 | 0 | 0 | 0 | 0 |
| 1604 | P133 | 0 | 0 | 0 | 0 | 0 |
| 1605 | P1131 | 0 | 0 | 0 | 0 | 0 |
| 1606 | P1130 | 0 | 0 | 0 | 0 | 0 |
| 1607 | P1129 | 0 | 0 | 0 | 0 | 0 |
| 1608 | P1126 | 0 | 0 | 0 | 0 | 0 |
| 1609 | P1080 | 0 | 0 | 0 | 0 | 0 |
| 1610 | P1079 | 0 | 0 | 0 | 0 | 0 |
| 1611 | P1043 | 0 | 0 | 0 | 0 | 0 |
| 1612 | P1027 | 0 | 0 | 0 | 0 | 0 |
| 1613 | P1025 | 0 | 0 | 0 | 0 | 0 |
| 1614 | P1015 | 0 | 0 | 0 | 0 | 0 |
| 1615 | P1013 | 0 | 0 | 0 | 0 | 0 |
| 1616 | P10 | 0 | 0 | 0 | 0 | 0 |
| 1617 | P1003 | 0 | 0 | 0 | 0 | 0 |
| 1618 | U287 | 1.23E-06 | 0.00000121 | 0.00000117 | 0.00000121 | 0 |
| 1619 | U272 | 0.0001115 | 0 | 0.00010887 | 0.0001099 | 0.000112685 |
| 1620 | U40 | 0.0005394 | 0 | 0 | 0.00053234 | 0 |
| 1621 | P179 | 0 | 0 | 0 | 0.0009989 | 0.00165891 |
| 1622 | U55 | 0.0004574 | 0 | 0 | 0.00109126 | 0.0012081 |
| 1623 | P1017 | 0.0004416 | 0 | 0.00092059 | 0.00175585 | 0 |
| 1624 | P954 | 0 | 0 | 0 | 0.00165811 | 0 |
| 1625 | U109 | 0.0005247 | 0 | 0 | 0.00125203 | 0 |
| 1626 | P589 | 0 | 0 | 0 | 0.00105904 | 0 |
| 1627 | P859 | 0 | 0 | 0 | 0.0013153 | 0 |
| 1628 | P1067 | 0.0006306 | 0 | 0.00131438 | 0.0010027 | 0.00124919 |
| 1629 | P1119 | 0.0008177 | 0 | 0.00085223 | 0.00097526 | 0 |
| 1630 | P272 | 0.0006939 | 0 | 0.00144696 | 0.00055204 | 0 |
| 1631 | P909 | 0.0014876 | 0.00122728 | 0.00177144 | 0.00101348 | 0.000841933 |
| 1632 | P131.P132 | 0.0016844 | 0 | 0.00233796 | 0.0008919 | 0.00171522 |
| 1633 | P559 | 0 | 0 | 0.00310688 | 0 | 0 |
| 1634 | P1020 | 0 | 0 | 0.0027548 | 0 | 0 |
| 1635 | P908 | 0 | 0 | 0.0028487 | 0 | 0 |
| 1636 | P885 | 0 | 0 | 0.00342632 | 0.00130787 | 0.00188782 |
| 1637 | P464 | 0 | 0 | 0.00310814 | 0 | 0.0019656 |
| 1638 | P890 | 0.0007583 | 0 | 0.00320873 | 0.00024475 | 0.00243969 |
| 1639 | P955.P957 | 0 | 0 | 0 | 0.0058326 | 0 |
| 1640 | P1045.P1047 | 0 | 0 | 0 | 0.00320912 | 0 |
| 1641 | P265 | 0 | 0 | 0 | 0.0032928 | 0 |
| 1642 | P8 | 0.0046792 | 0 | 0 | 0.00750418 | 0 |
| 1643 | P54 | 0.003055 | 0 | 0 | 0.00488379 | 0 |
| 1644 | P504 | 0.0021132 | 0 | 0.00110147 | 0.00588264 | 0 |
| 1645 | P573 | 0.0006667 | 0.00521614 | 0.00188172 | 0.00483074 | 0.000896192 |
| 1646 | U23 | 0.0014271 | 0.00549527 | 0.00099168 | 0.00378294 | 0.00251267 |
| 1647 | P563 | 0.0016118 | 0.00310348 | 0 | 0.00128196 | 0 |
| 1648 | P418 | 0.002713 | 0.00391814 | 0.00141436 | 0.00107919 | 0 |
| 1649 | P220 | 0 | 0.00368671 | 0.00133078 | 0.00101539 | 0 |
| 1650 | P964 | 0 | 0.00426506 | 0.00153907 | 0.00215245 | 0.00130036 |
| 1651 | P236 | 0.0039539 | 0.00507502 | 0.00366325 | 0.0045415 | 0.000580146 |
| 1652 | P274 | 0.0042994 | 0.00297954 | 0.00286699 | 0.00259702 | 0.00204407 |
| 1653 | P843 | 0.0024236 | 0.0032932 | 0.00326776 | 0.00351234 | 0.00357724 |
| 1654 | P1001 | 0.0039892 | 0.00341863 | 0.00411195 | 0.00316308 | 0.0036839 |
| 1655 | P1032 | 0.0004383 | 0.00253166 | 0.0036548 | 0.0017427 | 0.00173643 |
| 1656 | P167 | 0.0027486 | 0.0026455 | 0.0038183 | 0.0014563 | 0.00060498 |
| 1657 | P1077 | 0.0018397 | 0.00354252 | 0.00255741 | 0.00146347 | 0 |
| 1658 | P306 | 0.0013189 | 0.00380944 | 0.0027502 | 0.00209844 | 0.000870782 |
| 1659 | P1093 | 0.0021462 | 0.00309927 | 0.00447458 | 0.00426741 | 0 |
| 1660 | P426 | 0.0006648 | 0.00255975 | 0.00461841 | 0.00281844 | 0 |
| 1661 | P239 | 0 | 0.0026153 | 0.00471949 | 0.00396069 | 0.00119585 |
| 1662 | P1018 | 0.0006692 | 0.0038653 | 0.00418474 | 0.00425658 | 0.000441896 |
