# Supplementary material for: Dynamic Expression of Long Non-Coding RNAs (lncRNAs) in Adult Zebrafish
Source: PLoS One. 2013 Dec 31;8(12):e83616. doi: 10.1371/journal.pone.0083616 (PMC3877055; doi:10.1371/journal.pone.0083616)
Supplement: Table S4 — Comparison of lncRNA transcripts between the present study and previous studies (Ulitsky et al., 2011 and Pauli et al., 2012) generated by using ORF cut off set to 100 amino acids. (DOCX) [file pone.0083616.s005.docx]

**Table S4:** Comparison of lncRNA transcript between the present study and previous studies (Ulitsky et al., 2011 and Pauli et al., 2012) generated by using ORF cut off set to 100 amino acids.

|  | **Total lncRNA transcripts identified** | **Overlap with the present study (Kaushik et al)** |
| --- | --- | --- |
| **Adult zebrafish tissues (Kaushik et al)** | 6214 | NA |
| **Ulitsky et al 2011** | 691 | 176 |
| **Pauli et al 2012** | 1133 | 197 |
