# Supplementary material for: Dynamic Expression of Long Non-Coding RNAs (lncRNAs) in Adult Zebrafish
Source: PLoS One. 2013 Dec 31;8(12):e83616. doi: 10.1371/journal.pone.0083616 (PMC3877055; doi:10.1371/journal.pone.0083616)
Supplement: Table S5 — Genomic co-ordinates of the 442 lncRNA transcripts identified in this study. (DOCX) [file pone.0083616.s006.docx]

**Table S5**: Genomic co-ordinates of the 442 lncRNA transcripts identified in this study.

| S.No | LncRNA ID | Chromosome | Start | Stop |
| --- | --- | --- | --- | --- |
| 1 | lncBL_001 | chr21 | 42012553 | 42012894 |
| 2 | lncBL_002 | chr25 | 1546116 | 1546366 |
| 3 | lncBL_003 | chr14 | 1284531 | 1284834 |
| 4 | lncBL_004 | chr17 | 39911142 | 39911442 |
| 5 | lncBL_005 | chr6 | 26653300 | 26653711 |
| 6 | lncBL_006 | Zv9_scaffold3544 | 106800 | 107059 |
| 7 | lncBL_007 | chr24 | 40696950 | 40697182 |
| 8 | lncBL_008 | chr23 | 38022356 | 38022583 |
| 9 | lncBL_009 | chr25 | 8501722 | 8551311 |
| 10 | lncBL_010 | chr24 | 43114222 | 43156728 |
| 11 | lncBL_011 | chr3 | 2344066 | 2476421 |
| 12 | lncBL_012 | chr1 | 57119075 | 57153604 |
| 13 | lncBrBl_008 | chr19 | 25358094 | 25358345 |
| 14 | lncBr_001 | chr18 | 1420567 | 1421311 |
| 15 | lncBrBl_003 | chr9 | 41797321 | 41799741 |
| 16 | lncBr_002 | chr17 | 16743440 | 16744323 |
| 17 | lncBr_003 | chr24 | 38058933 | 38059726 |
| 18 | lncBr_004 | Zv9_scaffold3492 | 195012 | 195593 |
| 19 | lncBr_005 | chr18 | 4414892 | 4415541 |
| 20 | lncBr_006 | chr10 | 27818205 | 28373894 |
| 21 | lncBr_007 | chr9 | 2593641 | 2677305 |
| 22 | lncBr_008 | chr10 | 6857630 | 6858164 |
| 23 | lncBr_009 | chr10 | 6445831 | 6489847 |
| 24 | lncBrBl_007 | chr16 | 14084151 | 14084478 |
| 25 | lncBr_010 | chr14 | 12222002 | 12222584 |
| 26 | lncBr_011 | chr16 | 23228420 | 23228847 |
| 27 | lncBr_012 | chr8 | 100749 | 118742 |
| 28 | lncBr_013 | chr23 | 41507757 | 41508204 |
| 29 | lncBr_014 | chr16 | 54362191 | 54362786 |
| 30 | lncBrBl_005 | chr3 | 53270958 | 53271325 |
| 31 | lncBr_015 | chr23 | 34619051 | 34619427 |
| 32 | lncBrBl_009 | chr4 | 14506599 | 14507023 |
| 33 | lncBr_016 | chr3 | 56258865 | 56259284 |
| 34 | lncBr_017 | Zv9_NA731 | 3599 | 3971 |
| 35 | lncBr_018 | chr16 | 30232846 | 30233219 |
| 36 | lncBrBl_001 | chr5 | 9566270 | 9566482 |
| 37 | lncBr_019 | chr7 | 27279628 | 27279952 |
| 38 | lncBr_020 | chr13 | 11923289 | 11923610 |
| 39 | lncBr_021 | chr16 | 47910207 | 47910512 |
| 40 | lncBr_022 | chr11 | 9612217 | 9612552 |
| 41 | lncBr_023 | chr12 | 48802047 | 48846617 |
| 42 | lncBr_024 | Zv9_scaffold3486 | 36169 | 36492 |
| 43 | lncBr_025 | chr24 | 39093143 | 39093466 |
| 44 | lncBr_026 | chr18 | 16248863 | 16249249 |
| 45 | lncBr_027 | chr9 | 24519944 | 24520244 |
| 46 | lncBr_028 | chr19 | 7476861 | 7477158 |
| 47 | lncBr_029 | chr6 | 56827377 | 56827833 |
| 48 | lncBr_030 | chr13 | 11874003 | 11874265 |
| 49 | lncBr_031 | chr8 | 24105823 | 24106130 |
| 50 | lncBr_032 | chr7 | 60720954 | 60721276 |
| 51 | lncBrBl_006 | chr15 | 40192853 | 40193102 |
| 52 | lncBr_033 | chr4 | 22346388 | 22347001 |
| 53 | lncBr_034 | chr15 | 47399910 | 47400219 |
| 54 | lncBr_035 | chr4 | 2169549 | 2169796 |
| 55 | lncBr_036 | chr14 | 47989185 | 47989554 |
| 56 | lncBr_037 | chr22 | 20500910 | 20501228 |
| 57 | lncBr_038 | chr3 | 43798401 | 43862748 |
| 58 | lncBr_039 | chr11 | 31551197 | 31603385 |
| 59 | lncBr_040 | chr8 | 55562339 | 55562595 |
| 60 | lncBr_041 | chr17 | 51949622 | 51951160 |
| 61 | lncBr_042 | chr13 | 37930297 | 37932722 |
| 62 | lncBr_043 | Zv9_NA498 | 6975 | 7217 |
| 63 | lncBrBl_004 | chr10 | 5595067 | 5595338 |
| 64 | lncBr_044 | chr17 | 3053500 | 3053810 |
| 65 | lncBr_045 | chr2 | 14310010 | 14421231 |
| 66 | lncBr_046 | chr2 | 4511649 | 4511854 |
| 67 | lncBrBl_002 | chr25 | 29238151 | 29243699 |
| 68 | lncBr_047 | chr9 | 4162547 | 4162758 |
| 69 | lncBr_048 | chr6 | 49312314 | 49312522 |
| 70 | lncMBr_030 | chr10 | 43188665 | 43189330 |
| 71 | lncMBr_017 | chr3 | 61575398 | 61576045 |
| 72 | lncMBrBl_010 | chr2 | 58189115 | 58189742 |
| 73 | lncMBr_031 | Zv9_NA771 | 67035 | 67635 |
| 74 | lncMBrBl_008 | chr6 | 32415144 | 32531676 |
| 75 | lncMBr_037 | chr18 | 526859 | 527343 |
| 76 | lncMBr_033 | chr3 | 44573573 | 44574041 |
| 77 | lncMBrBl_006 | chr7 | 7753727 | 7755048 |
| 78 | lncMBr_009 | chr2 | 56720420 | 56720852 |
| 79 | lncMBr_023 | chr16 | 50395098 | 50395521 |
| 80 | lncMBr_014 | chr9 | 57543445 | 57543866 |
| 81 | lncMBr_040 | chr1 | 9653840 | 9654247 |
| 82 | lncMBr_011 | chr3 | 48293410 | 48293772 |
| 83 | lncMBr_034 | chr13 | 21829484 | 21829825 |
| 84 | lncMBr_025 | chr17 | 24363027 | 24363363 |
| 85 | lncMBr_022 | chr18 | 526438 | 526750 |
| 86 | lncMBr_035 | chr14 | 11299986 | 11300298 |
| 87 | lncMBrBl_009 | chr12 | 22029509 | 22029986 |
| 88 | lncMBrBl_007 | chr1 | 40506258 | 40506710 |
| 89 | lncMBr_020 | chr10 | 27818205 | 28373894 |
| 90 | lncMBr_027 | chr19 | 10100331 | 10100620 |
| 91 | lncMBrBl_003 | chr5 | 9992382 | 9992806 |
| 92 | lncMBr_001 | chr19 | 24899557 | 24900211 |
| 93 | lncMBr_039 | chr5 | 59640594 | 59640861 |
| 94 | lncMBr_005 | chr7 | 59500119 | 59500509 |
| 95 | lncMBr_028 | chr6 | 9829140 | 9851927 |
| 96 | lncMBr_041 | chr19 | 21143217 | 21143598 |
| 97 | lncMBr_026 | chr23 | 17522276 | 17522536 |
| 98 | lncMBr_002 | chr2 | 57705309 | 57705558 |
| 99 | lncMBr_003 | chr15 | 25949961 | 25950744 |
| 100 | lncMBr_019 | chr4 | 22347336 | 22347770 |
| 101 | lncMBr_036 | chr4 | 59767799 | 59768414 |
| 102 | lncMBl_001 | chr3 | 19324699 | 19324934 |
| 103 | lncMBrBl_005 | chr13 | 3835814 | 3846620 |
| 104 | lncMBr_016 | chr1 | 49800792 | 49801354 |
| 105 | lncMBl_003 | Zv9_scaffold3455 | 338975 | 339273 |
| 106 | lncMBrBl_002 | chr9 | 54571370 | 54572059 |
| 107 | lncMBr_024 | chr7 | 8140753 | 8144023 |
| 108 | lncMBr_008 | chr16 | 31452662 | 31452870 |
| 109 | lncMBr_021 | chr1 | 55033592 | 55034000 |
| 110 | lncMBr_004 | chr24 | 38076526 | 38076730 |
| 111 | lncMBr_038 | chr14 | 23594590 | 23594975 |
| 112 | lncMBr_010 | chr5 | 396217 | 396492 |
| 113 | lncMBr_029 | chr10 | 40521214 | 40521442 |
| 114 | lncMBrBl_004 | chr23 | 36849430 | 36850003 |
| 115 | lncMBrBl_001 | chr13 | 43904940 | 43905163 |
| 116 | lncMBl_002 | Zv9_NA178 | 1786 | 32445 |
| 117 | lncMBr_012 | Zv9_NA614 | 1878 | 2222 |
| 118 | lncMBr_006 | chr3 | 14876539 | 14876887 |
| 119 | lncMBl_004 | chr6 | 10988210 | 10988411 |
| 120 | lncMBr_018 | chr20 | 54528905 | 54529461 |
| 121 | lncMBr_032 | chr13 | 29660740 | 29660945 |
| 122 | lncMBr_007 | chr10 | 4816990 | 4817359 |
| 123 | lncMBr_013 | Zv9_NA584 | 69810 | 70011 |
| 124 | lncMBr_015 | chr22 | 18764625 | 18764949 |
| 125 | lncM_001 | chr9 | 44106248 | 44106489 |
| 126 | lncM_002 | chr1 | 13370487 | 13370748 |
| 127 | lncM_003 | chr5 | 65057608 | 65057854 |
| 128 | lncM_004 | chr15 | 33764665 | 33764880 |
| 129 | lncLMBrBl_001 | chr16 | 22401685 | 22402221 |
| 130 | lncLBr_004 | chr17 | 38947661 | 38948080 |
| 131 | lncLMBrBl_006 | chr19 | 213465 | 213843 |
| 132 | lncLMBr_003 | chr17 | 45414135 | 45421568 |
| 133 | lncLMBr_001 | chr20 | 53159638 | 53159972 |
| 134 | lncLBr_001 | chr13 | 9125535 | 9126051 |
| 135 | lncLMBrBl_005 | chr2 | 14459732 | 14460033 |
| 136 | lncLMBr_005 | chr12 | 40259482 | 40259775 |
| 137 | lncLBl_002 | Zv9_NA808 | 11476 | 11759 |
| 138 | lncLBr_002 | chr7 | 32304508 | 32476748 |
| 139 | lncLBr_006 | chr16 | 46594868 | 46595125 |
| 140 | lncLBr_005 | chr3 | 13103007 | 13103255 |
| 141 | lncLMBrBl_007 | chr16 | 54630044 | 54630624 |
| 142 | lncLMBrBl_004 | chr5 | 2104216 | 2104487 |
| 143 | lncLMBrBl_002 | chr8 | 53428207 | 53428729 |
| 144 | lncLBrBl_001 | chr21 | 1940022 | 1940441 |
| 145 | lncLMBrBl_003 | chr7 | 75757280 | 75765867 |
| 146 | lncLMBr_002 | chr18 | 9527268 | 9527710 |
| 147 | lncLBl_003 | chr11 | 41940027 | 41940424 |
| 148 | lncLBl_001 | chr9 | 8415781 | 8416051 |
| 149 | lncL_001 | chr7 | 28817211 | 28822970 |
| 150 | lncL_002 | chr17 | 42372064 | 42389977 |
| 151 | lncLBr_003 | chr5 | 40069517 | 40069926 |
| 152 | lncLMBr_004 | chr24 | 34724759 | 34725312 |
| 153 | lncHMBrBl_032 | chr11 | 8127307 | 8128027 |
| 154 | lncHMBr_013 | chr6 | 32660332 | 32661016 |
| 155 | lncHBrBl_020 | chr4 | 60109512 | 60110181 |
| 156 | lncHMBr_034 | chr14 | 2060779 | 2061431 |
| 157 | lncHLBrBl_006 | chr8 | 55389721 | 55406708 |
| 158 | lncHLMBrBl_057 | chr1 | 47992460 | 47992996 |
| 159 | lncHMBr_020 | chr16 | 30892808 | 30893306 |
| 160 | lncHBr_017 | chr11 | 30655810 | 30666861 |
| 161 | lncHMBrBl_027 | chr24 | 16612323 | 16612814 |
| 162 | lncHLMBrBl_064 | chr15 | 35715311 | 35715789 |
| 163 | lncHBr_009 | chr17 | 31706195 | 31706672 |
| 164 | lncHLMBrBl_028 | chr23 | 40237870 | 40238347 |
| 165 | lncHLMBr_009 | chr7 | 66652656 | 66653458 |
| 166 | lncHLMBl_001 | chr24 | 22669296 | 22669744 |
| 167 | lncHMBr_005 | chr4 | 21278074 | 21278518 |
| 168 | lncHLBr_001 | chr17 | 30879911 | 30880355 |
| 169 | lncHLMBr_015 | chr8 | 52478196 | 52478633 |
| 170 | lncHMBr_014 | chr13 | 48194020 | 48194456 |
| 171 | lncHMBr_027 | chr8 | 8028969 | 8029394 |
| 172 | lncHMBr_036 | chr12 | 49467725 | 49468425 |
| 173 | lncHLMBr_011 | chr11 | 1879309 | 1880273 |
| 174 | lncHLMBr_016 | chr7 | 23429070 | 23430003 |
| 175 | lncHBrBl_010 | chr6 | 1737793 | 1781413 |
| 176 | lncHMBl_005 | chr1 | 11246516 | 11246876 |
| 177 | lncHMBr_022 | chr17 | 49055356 | 49055711 |
| 178 | lncHMBr_028 | chr8 | 4909980 | 4910330 |
| 179 | lncHMBr_024 | chr5 | 14777 | 15121 |
| 180 | lncHMBrBl_015 | chr23 | 36537325 | 36537890 |
| 181 | lncHMBrBl_002 | chr2 | 56339517 | 56339841 |
| 182 | lncHMBrBl_013 | chr7 | 65988537 | 65988860 |
| 183 | lncHLMBr_014 | chr3 | 17170541 | 17171488 |
| 184 | lncHLMBr_006 | chr12 | 10102882 | 10103406 |
| 185 | lncHLMBr_010 | chr1 | 55032820 | 55033336 |
| 186 | lncHLMBrBl_032 | chr6 | 33567456 | 33568378 |
| 187 | lncHLBrBl_005 | chr20 | 36666997 | 36667914 |
| 188 | lncHMBrBl_005 | chr1 | 22351871 | 22363277 |
| 189 | lncHMBrBl_003 | chr9 | 18777929 | 18778223 |
| 190 | lncHMBr_029 | chr3 | 9145324 | 9146709 |
| 191 | lncHBr_018 | chr21 | 26681574 | 26681850 |
| 192 | lncHBr_015 | chr2 | 42692924 | 42701459 |
| 193 | lncHLBr_003 | chr20 | 9340589 | 9341186 |
| 194 | lncHMBr_038 | chr20 | 27227428 | 27228021 |
| 195 | lncHBl_001 | Zv9_NA384 | 35326 | 36080 |
| 196 | lncHMBrBl_001 | chr3 | 60947710 | 60947972 |
| 197 | lncHLMBrBl_042 | chr14 | 4825299 | 4826042 |
| 198 | lncHLMBrBl_054 | chr12 | 14665646 | 14666226 |
| 199 | lncHLBrBl_007 | chr20 | 2225805 | 2227091 |
| 200 | lncHLMBr_002 | chr5 | 18952138 | 18952672 |
| 201 | lncHMBrBl_020 | chr8 | 8832974 | 8833218 |
| 202 | lncHBl_003 | chr24 | 25844162 | 25855506 |
| 203 | lncHMBr_031 | chr20 | 15196488 | 15197371 |
| 204 | lncHLMBrBl_018 | chr2 | 41544214 | 41544572 |
| 205 | lncHBr_010 | chr17 | 20530257 | 20620014 |
| 206 | lncHBl_013 | chr10 | 8787964 | 8788188 |
| 207 | lncHLMBrBl_017 | chr14 | 51256835 | 51257305 |
| 208 | lncHMBr_032 | chr1 | 47458547 | 47459256 |
| 209 | lncHBr_013 | chr18 | 11807623 | 11807844 |
| 210 | lncHMBr_009 | chr13 | 52392350 | 52392689 |
| 211 | lncHMBr_006 | chr19 | 7185365 | 7185693 |
| 212 | lncHBrBl_001 | chr3 | 24930217 | 24930541 |
| 213 | lncHBrBl_014 | chr14 | 11502153 | 11502687 |
| 214 | lncHLMBr_005 | Zv9_NA738 | 4059 | 4266 |
| 215 | lncHBr_007 | chr23 | 32916482 | 32916687 |
| 216 | lncHBrBl_003 | chr10 | 20621566 | 20621969 |
| 217 | lncHLMBrBl_021 | chr6 | 17407042 | 17407816 |
| 218 | lncHMBr_011 | chr5 | 31537441 | 31537910 |
| 219 | lncHBl_011 | chr15 | 4965247 | 4965529 |
| 220 | lncHLMBrBl_026 | chr22 | 6690268 | 6690992 |
| 221 | lncHMBrBl_018 | chr1 | 49871704 | 49872161 |
| 222 | lncHBrBl_018 | chr19 | 5810 | 6086 |
| 223 | lncHBl_008 | chr17 | 7283782 | 7284055 |
| 224 | lncHLMBrBl_050 | chr7 | 72122127 | 72122791 |
| 225 | lncHLMBrBl_012 | chr8 | 45033786 | 45034047 |
| 226 | lncHBl_009 | chr3 | 6223964 | 6224384 |
| 227 | lncHBr_006 | chr10 | 403152 | 403568 |
| 228 | lncHLMBr_007 | chr10 | 24928898 | 24929154 |
| 229 | lncHBr_008 | chr6 | 10489071 | 10489398 |
| 230 | lncHBrBl_009 | chr19 | 7198035 | 7198436 |
| 231 | lncHMBl_010 | chr2 | 48379559 | 48379958 |
| 232 | lncHMBrBl_006 | chr15 | 6992474 | 6992947 |
| 233 | lncHMBr_025 | chr5 | 14375089 | 14375487 |
| 234 | lncHBrBl_016 | chr23 | 24418964 | 24419286 |
| 235 | lncHBl_017 | chr10 | 15111283 | 15111529 |
| 236 | lncHMBrBl_023 | chr1 | 34229667 | 34230057 |
| 237 | lncHMBr_019 | chr13 | 27597583 | 27598476 |
| 238 | lncHMBr_026 | chr2 | 34095638 | 34273788 |
| 239 | lncHLMBrBl_011 | chr1 | 55778354 | 55779082 |
| 240 | lncHBr_002 | chr10 | 41317934 | 41446076 |
| 241 | lncHBr_020 | chr18 | 11806935 | 11807528 |
| 242 | lncHMBl_015 | chr5 | 14820439 | 14820875 |
| 243 | lncHLBrBl_002 | chr7 | 21793862 | 21794628 |
| 244 | lncHMBrBl_011 | chr15 | 42915786 | 42916218 |
| 245 | lncHLM_001 | chr10 | 24928082 | 24928314 |
| 246 | lncHMBr_007 | chr1 | 6341850 | 6342407 |
| 247 | lncHMBr_021 | chr6 | 54346712 | 54381090 |
| 248 | lncHMBrBl_009 | chr16 | 32341461 | 32374902 |
| 249 | lncHLMBl_003 | chr9 | 1348559 | 1348913 |
| 250 | lncHLMBl_002 | chr12 | 26820404 | 26820630 |
| 251 | lncHBl_007 | chr16 | 19675399 | 19675685 |
| 252 | lncHBrBl_013 | chr5 | 45890488 | 45890712 |
| 253 | lncHLMBrBl_041 | chr19 | 32803104 | 32803812 |
| 254 | lncHLMBrBl_013 | chr19 | 2882408 | 2882873 |
| 255 | lncHLMBrBl_025 | chr19 | 18416545 | 18423495 |
| 256 | lncHLBl_003 | chr7 | 40142058 | 40142274 |
| 257 | lncHBr_003 | chr1 | 54529819 | 54530031 |
| 258 | lncHLMBrBl_063 | chr8 | 22903563 | 22904050 |
| 259 | lncHBr_011 | chr23 | 39168018 | 39168426 |
| 260 | lncHLMBrBl_067 | chr1 | 54677660 | 54749049 |
| 261 | lncHLMBr_012 | chr5 | 73587862 | 73588361 |
| 262 | lncHLMBrBl_046 | chr5 | 65837740 | 65838335 |
| 263 | lncHBr_012 | chr9 | 20691078 | 20691326 |
| 264 | lncHLMBr_001 | chr17 | 49805908 | 49806295 |
| 265 | lncHLMBrBl_034 | chr3 | 15224913 | 15225395 |
| 266 | lncHMBl_016 | chr15 | 30366152 | 30366770 |
| 267 | lncHMBr_040 | chr7 | 6735233 | 6735512 |
| 268 | lncHMBrBl_022 | chr4 | 59206892 | 59207169 |
| 269 | lncHLMBrBl_006 | chr4 | 58411719 | 58411995 |
| 270 | lncHLMBrBl_002 | chr4 | 27761625 | 27762294 |
| 271 | lncHMBr_035 | Zv9_NA237 | 3199 | 3517 |
| 272 | lncHMBrBl_010 | chr10 | 15103259 | 15103793 |
| 273 | lncHBrBl_017 | chr25 | 5565187 | 5565415 |
| 274 | lncHLMBrBl_045 | chr21 | 762833 | 763059 |
| 275 | lncHMBr_037 | chr23 | 42309944 | 42310540 |
| 276 | lncHLMBrBl_049 | chr23 | 43027147 | 43044297 |
| 277 | lncHBr_023 | chr22 | 8115144 | 8290526 |
| 278 | lncHLMBrBl_058 | chr18 | 2716231 | 2716644 |
| 279 | lncHMBl_011 | chr2 | 58907690 | 58907978 |
| 280 | lncHBr_022 | chr11 | 2957548 | 2957944 |
| 281 | lncHMBrBl_019 | chr23 | 13636091 | 13636376 |
| 282 | lncHMBr_010 | chr17 | 2222367 | 2222796 |
| 283 | lncHLBrBl_004 | chr21 | 34859370 | 34859828 |
| 284 | lncHLBl_001 | chr6 | 19855036 | 19855632 |
| 285 | lncHLMBrBl_065 | chr22 | 21099239 | 21099687 |
| 286 | lncHLMBrBl_030 | chr4 | 20893243 | 20893583 |
| 287 | lncHMBrBl_028 | chr2 | 29610566 | 29610938 |
| 288 | lncHBrBl_004 | chr12 | 572397 | 572699 |
| 289 | lncHLMBrBl_005 | chr6 | 20893001 | 20974061 |
| 290 | lncHLBr_004 | chr17 | 33080223 | 33080819 |
| 291 | lncHBr_016 | chr8 | 33360203 | 33363696 |
| 292 | lncHMBrBl_007 | chr6 | 29502098 | 29503755 |
| 293 | lncHLMBl_006 | chr13 | 45434521 | 45435126 |
| 294 | lncHLMBrBl_015 | chr2 | 37405826 | 37406242 |
| 295 | lncHLMBl_010 | chr1 | 37213180 | 37228029 |
| 296 | lncHMBrBl_012 | chr15 | 2805862 | 2806539 |
| 297 | lncHMBrBl_025 | chr5 | 50062485 | 50062766 |
| 298 | lncHMBr_008 | chr17 | 49051891 | 49052229 |
| 299 | lncHLMBrBl_019 | chr12 | 48061639 | 48151709 |
| 300 | lncHMBrBl_026 | Zv9_scaffold3487 | 171938 | 172291 |
| 301 | lncHBl_004 | chr3 | 4232992 | 4233393 |
| 302 | lncHLMBrBl_022 | chr6 | 17335007 | 17335435 |
| 303 | lncHLBl_002 | chr13 | 33682917 | 33683235 |
| 304 | lncHMBrBl_016 | Zv9_NA107 | 51669 | 52122 |
| 305 | lncHMBl_008 | chr13 | 38030697 | 38031022 |
| 306 | lncHLBl_004 | chr4 | 29035804 | 29036278 |
| 307 | lncHLMBrBl_033 | chr6 | 57348430 | 57349200 |
| 308 | lncHMBrBl_017 | chr10 | 17129289 | 17129824 |
| 309 | lncHLMBrBl_039 | chr11 | 27164195 | 27164832 |
| 310 | lncHMBr_015 | chr13 | 3991055 | 3991334 |
| 311 | lncHBl_010 | chr10 | 22287904 | 22288130 |
| 312 | lncH_001 | chr22 | 38857701 | 38860155 |
| 313 | lncHLMBrBl_056 | chr21 | 44455140 | 44456007 |
| 314 | lncHBrBl_008 | chr17 | 20769915 | 20770254 |
| 315 | lncHMBl_019 | chr5 | 22401701 | 22402060 |
| 316 | lncHMBrBl_033 | Zv9_NA930 | 17264 | 17645 |
| 317 | lncHLMBrBl_020 | chr25 | 22530836 | 22531169 |
| 318 | lncHMBrBl_029 | chr3 | 55651523 | 55651776 |
| 319 | lncH_002 | chr3 | 20126611 | 20126929 |
| 320 | lncHM_003 | chr22 | 2365124 | 2365602 |
| 321 | lncHLMBrBl_066 | chr24 | 43894933 | 43896147 |
| 322 | lncHL_001 | chr16 | 13852329 | 13923633 |
| 323 | lncHLMBrBl_016 | chr12 | 28112902 | 28113235 |
| 324 | lncHLMBrBl_027 | chr2 | 42729676 | 42730036 |
| 325 | lncHLMBrBl_029 | chr14 | 53353961 | 53354186 |
| 326 | lncHLMBrBl_055 | chr2 | 57918637 | 57919048 |
| 327 | lncHBrBl_012 | chr10 | 30609763 | 30610128 |
| 328 | lncHLMBrBl_044 | Zv9_scaffold3546 | 143886 | 144713 |
| 329 | lncHLBrBl_003 | chr19 | 25242262 | 25243732 |
| 330 | lncHBl_014 | chr1 | 45597370 | 45608211 |
| 331 | lncHMBrBl_021 | chr9 | 50113561 | 50114279 |
| 332 | lncHMBr_018 | chr20 | 8245441 | 8245774 |
| 333 | lncHLBrBl_008 | chr22 | 8974251 | 8974584 |
| 334 | lncHMBr_033 | chr11 | 11180124 | 11180417 |
| 335 | lncHBl_006 | chr16 | 19162666 | 19162977 |
| 336 | lncHBr_014 | chr8 | 46320276 | 46320698 |
| 337 | lncHLMBrBl_010 | chr25 | 13323701 | 13324133 |
| 338 | lncHMBrBl_030 | chr3 | 1609347 | 1609631 |
| 339 | lncH_003 | chr25 | 6381613 | 6381930 |
| 340 | lncHMBl_018 | chr19 | 16832825 | 16833087 |
| 341 | lncHLMBrBl_024 | chr15 | 40294275 | 40294572 |
| 342 | lncH_004 | chr8 | 23355789 | 23355996 |
| 343 | lncHMBr_030 | chr4 | 669148 | 670533 |
| 344 | lncHMBr_001 | chr6 | 33679283 | 33680139 |
| 345 | lncHMBrBl_008 | chr6 | 9485214 | 9500656 |
| 346 | lncHLMBrBl_014 | chr15 | 13777105 | 13777720 |
| 347 | lncHBl_016 | chr24 | 37800587 | 37800876 |
| 348 | lncHBrBl_021 | chr18 | 47827330 | 47827619 |
| 349 | lncHMBr_039 | chr15 | 2812212 | 2812518 |
| 350 | lncHMBl_014 | chr23 | 29308099 | 29308405 |
| 351 | lncHBrBl_002 | chr6 | 37260494 | 37260778 |
| 352 | lncHMBrBl_024 | chr15 | 30212954 | 30213238 |
| 353 | lncHBrBl_007 | chr5 | 73544889 | 73545197 |
| 354 | lncHLMBl_005 | chr2 | 42064729 | 42065004 |
| 355 | lncHBl_015 | chr9 | 40713 | 40971 |
| 356 | lncHMBl_003 | chr2 | 33858412 | 33858699 |
| 357 | lncHLMBr_003 | chr10 | 9280581 | 9282240 |
| 358 | lncHLMBrBl_048 | chr16 | 49570905 | 49571321 |
| 359 | lncHBl_002 | chr7 | 68126249 | 68126472 |
| 360 | lncHBl_005 | chr22 | 7557772 | 7558020 |
| 361 | lncHLBrBl_009 | chr10 | 27795856 | 27796157 |
| 362 | lncHLMBr_017 | chr12 | 29925912 | 29926145 |
| 363 | lncHLMBrBl_060 | chr5 | 7185695 | 7185941 |
| 364 | lncHLMBrBl_035 | chr15 | 22774914 | 22775198 |
| 365 | lncHMBr_023 | chr3 | 59662742 | 59663063 |
| 366 | lncH_005 | chr14 | 30674803 | 30675045 |
| 367 | lncH_006 | chr16 | 18887219 | 18887461 |
| 368 | lncHBl_018 | chr7 | 40864070 | 40864312 |
| 369 | lncH_007 | Zv9_scaffold3518 | 9774 | 10305 |
| 370 | lncHMBl_017 | chr11 | 41610456 | 41610696 |
| 371 | lncHBrBl_015 | chr6 | 59393010 | 59393270 |
| 372 | lncHLMBrBl_053 | chr16 | 17436110 | 17438715 |
| 373 | lncHMBrBl_014 | chr19 | 20126972 | 20127238 |
| 374 | lncHBrBl_011 | chr4 | 59172960 | 59173202 |
| 375 | lncHLMBrBl_051 | Zv9_NA17 | 56592 | 56902 |
| 376 | lncHLMBrBl_036 | chr16 | 12343048 | 12343530 |
| 377 | lncHLMBrBl_008 | chr17 | 14875443 | 14875704 |
| 378 | lncH_008 | chr8 | 145526 | 145764 |
| 379 | lncHLMBl_009 | chr3 | 45321508 | 45322139 |
| 380 | lncHMBl_002 | chr3 | 44215397 | 44215790 |
| 381 | lncHLMBrBl_023 | chr14 | 24079033 | 24079313 |
| 382 | lncHLMBl_011 | chr13 | 53782752 | 53782987 |
| 383 | lncHBr_005 | chr2 | 56254755 | 56254967 |
| 384 | lncHBrBl_006 | chr15 | 726771 | 727026 |
| 385 | lncHBr_004 | chr7 | 62867509 | 62867729 |
| 386 | lncHMBr_012 | chr4 | 3265237 | 3275995 |
| 387 | lncHMBr_004 | chr24 | 11224334 | 11224542 |
| 388 | lncH_009 | chr16 | 18905694 | 18906094 |
| 389 | lncHMBr_003 | chr8 | 46319920 | 46320221 |
| 390 | lncHLMBrBl_003 | chr3 | 56069083 | 56069667 |
| 391 | lncHLMBrBl_052 | chr15 | 13778506 | 13778754 |
| 392 | lncHBrBl_019 | chr9 | 1107426 | 1107631 |
| 393 | lncHMBr_041 | chr3 | 31340971 | 31341206 |
| 394 | lncHLMBr_013 | chr7 | 53201107 | 53204521 |
| 395 | lncHLMBrBl_047 | chr22 | 22533096 | 22533365 |
| 396 | lncHLMBrBl_007 | chr2 | 51371737 | 51371985 |
| 397 | lncHMBrBl_031 | chr9 | 53165944 | 53166441 |
| 398 | lncHBr_001 | chr5 | 25963841 | 25964130 |
| 399 | lncHMBl_013 | chr12 | 46188009 | 46236733 |
| 400 | lncHMBl_012 | chr24 | 40044817 | 40045037 |
| 401 | lncHMBl_007 | Zv9_scaffold3459 | 260879 | 261181 |
| 402 | lncH_010 | chr17 | 15286194 | 15286432 |
| 403 | lncHBr_019 | chr16 | 18902740 | 18902986 |
| 404 | lncHLMBl_004 | chr1 | 1684774 | 1685011 |
| 405 | lncHMBl_001 | chr25 | 13379805 | 13380093 |
| 406 | lncHLMBrBl_009 | chr23 | 36539154 | 36539565 |
| 407 | lncHLMBrBl_038 | chr14 | 17229008 | 17270814 |
| 408 | lncHMBr_002 | chr11 | 44715151 | 44715396 |
| 409 | lncHMBl_006 | chr10 | 22126924 | 22127168 |
| 410 | lncHLBrBl_001 | chr5 | 62740941 | 62741146 |
| 411 | lncHBrBl_005 | chr4 | 13060103 | 13060307 |
| 412 | lncHMBrBl_004 | chr7 | 19821550 | 19821857 |
| 413 | lncHLMBrBl_037 | chr20 | 43154350 | 43272081 |
| 414 | lncHM_002 | chr12 | 31856518 | 31856783 |
| 415 | lncHMBl_004 | chr10 | 42440854 | 42441143 |
| 416 | lncHBl_019 | chr22 | 33188909 | 33189189 |
| 417 | lncH_011 | chr18 | 33543268 | 33543469 |
| 418 | lncHBr_021 | chr9 | 52429590 | 52429791 |
| 419 | lncHL_002 | chr14 | 7493588 | 7495276 |
| 420 | lncHLMBrBl_059 | chr11 | 31207363 | 31207671 |
| 421 | lncHLBr_002 | chr14 | 7493588 | 7495276 |
| 422 | lncH_012 | chr19 | 48713075 | 48713368 |
| 423 | lncHLBrBl_010 | chr14 | 33975741 | 34170874 |
| 424 | lncHBl_012 | chr9 | 1922277 | 1922490 |
| 425 | lncH_013 | chr7 | 21496778 | 21496985 |
| 426 | lncHLMBrBl_043 | chr20 | 23068913 | 23070784 |
| 427 | lncHLMBrBl_031 | chr8 | 11281728 | 11281998 |
| 428 | lncHMBr_017 | chr3 | 25641791 | 25642046 |
| 429 | lncHBl_020 | chr6 | 36249396 | 36249660 |
| 430 | lncHLMBrBl_062 | chr13 | 44235210 | 44235581 |
| 431 | lncHLMBrBl_040 | chr5 | 250629 | 261036 |
| 432 | lncHLMBl_007 | chr10 | 4966139 | 4966644 |
| 433 | lncHMBl_009 | chr13 | 15188459 | 15188670 |
| 434 | lncHLMBl_008 | chr22 | 6411717 | 6412034 |
| 435 | lncHLMBrBl_001 | chr3 | 35005011 | 35066751 |
| 436 | lncHLMBr_004 | chr7 | 59131059 | 59168590 |
| 437 | lncHMBr_016 | chr3 | 13611301 | 13611542 |
| 438 | lncHLMBrBl_004 | chr2 | 43644045 | 43644376 |
| 439 | lncHM_001 | chr23 | 10014448 | 10014686 |
| 440 | lncHLMBr_008 | chr25 | 1619517 | 1619950 |
| 441 | lncHLMBrBl_061 | chr3 | 30399862 | 30400150 |
| 442 | lncHMBrBl_034 | chr3 | 3532157 | 3896406 |
