# Supplementary material for: Dynamic Expression of Long Non-Coding RNAs (lncRNAs) in Adult Zebrafish
Source: PLoS One. 2013 Dec 31;8(12):e83616. doi: 10.1371/journal.pone.0083616 (PMC3877055; doi:10.1371/journal.pone.0083616)
Supplement: Table S6 — List of oligo sequences used in the study. (DOCX) [file pone.0083616.s007.docx]

**Table S6:** List of oligo sequences used in the study.

| **S.No.** | **Gene name** | **Forward primer** | **Reverse primer** |
| --- | --- | --- | --- |
| 1 | *myl7-001* | GTTGACCAGGCTTTTGCAGTG | GGGTATTGTGTTGAAATGCAAC |
| 2 | *murcb* | CTGATAAACTGGGACTGGCAG | ACTGGTCGCCGTGTGCTCCTT |
| 3 | *tal1* | CCACGCCACCGAGCTAAAAAA | TGCACCATTCGAGTGTCGCTC |
| 4 | *mdka* | AGAAGAATAAGGGAGGTAAGG | CTCCAAATTCTTTCTTCCAGT |
| 5 | *tfr* | ATTGTTGGCTCTCGTTGGCAG | CTGAGTGTGTTCTCGATGCGC |
| 6 | *lncH_007* | CCCTGGTGTGCTTAAAATGTA | CTGCAATGCAGCCTGTTGAAT |
| 7 | *lncH_005* | CTCAAGGGAACCAGGGTTGTA | CTGTCAACAAAACTGTCACCTG |
| 8 | *lncLBr_003* | CCTTTTGTCGTTCCAATCCCC | ACGCAGGAGAGAGGATACAGG |
| 9 | *lncL_001* | GCTAGATACATTGTTCTCCATG | ATCCTAAACTGTGCTGCAGCT |
| 10 | *lncM_001* | CTGACCTACAATTTTATCCCA | AAAGGATTAGTAAAGCAGCGG |
| 11 | *lncM_003* | CCCCTGTCCGCTGTTTACTTT | ACTACTTTGATCCCCCTTGAC |
| 12 | *lncBrM_002* | CAGAAACTGACCTGAAACTGA | ATGTATTTGAAATAGCACGCGG |
| 13 | *lncBrM_028* | CTCTGGTGAAGAGACAGCCGT | GGTTAACTGCTCCTCTTCCAA |
| 14 | *lncBlH_017* | GAGTATTGCACATTTGTACAA | ATTCACCTATTCCTGTTTGTT |
| 15 | *lncHMBr_035* | CCAATCATGAGGCACTGGAAC | AGAGCCCGTCAGCTCACCCTTT |
